# Dosing and Safety Profile of Aficamten in Symptomatic Obstructive Hypertrophic Cardiomyopathy: Results From SEQUOIA‐HCM

**DOI:** 10.1161/JAHA.124.035993

**Published:** 2024-07-26

**Authors:** Caroline J. Coats, Ahmad Masri, Michael E. Nassif, Roberto Barriales‐Villa, Michael Arad, Nuno Cardim, Lubna Choudhury, Brian Claggett, Hans‐Dirk Düngen, Pablo Garcia‐Pavia, Albert A. Hagège, James L. Januzzi, Matthew M. Y. Lee, Gregory D. Lewis, Chang‐Sheng Ma, Martin S. Maron, Zi Michael Miao, Michelle Michels, Iacopo Olivotto, Artur Oreziak, Anjali T. Owens, John A. Spertus, Scott D. Solomon, Jacob Tfelt‐Hansen, Marion van Sinttruije, Josef Veselka, Hugh Watkins, Daniel L. Jacoby, Polina German, Stephen B. Heitner, Stuart Kupfer, Justin D. Lutz, Fady I. Malik, Lisa Meng, Amy Wohltman, Theodore P. Abraham, the SEQUOIA‐HCM Investigators, Yuhui Zhang, Haibo Yang, Chunli Shao, Zuyi Yuan, Qingchun Zeng, Xiaodong Li, Yushi Wang, Yan Shu, Mulei Chen, Ling Tao, Xinli Li, Jingfeng Wang, Zaixin Yu, Xiang Cheng, Kui Hong, David Zemanek, Henning Bundgaard, Jens Thune, Morten Jensen, Jens Mogensen, Gilbert Habib, Philippe Charron, Thibault Lhermusier, Jean‐Noël Trochu, Patricia Reant, Damien Logeart, Veselin Mitrovic, Tarek Bekfani, Frank Edelmann, Tim Seidler, Benjamin Meder, Paul Christian Schulze, Stephan Stoerk, Tienush Rassaf, Bela Merkely, Donna Zfat‐Zwas, Majdi Halabi, Offir Paz, Xavier Piltz, Marco Metra, Marco Canepa, Beatrice Musumeci, Michele Emdin, Ahmad Amin, Christian Knackstedt, Wojciech Wojakowski, Dariusz Dudek, Alexandra Toste, José Mesquita Bastos, Juan Ramón Gimeno Blanes, Rafael Jesus Hidalgo Urbano, Ana Garcia Alvarez, Luis Miguel Rincón Diaz, Tomas Vicente Ripoll Vera, Perry Elliott, NHS Greater Glasgow, Rob Cooper, Liverpool Heart, Masliza Mahmod, Antonis Pantazis, Maria Teresa Tome Esteban, Oregon Health, Ali Marian, David Owens, Frank McGrew, Richard Bach, Omar Wever‐Pinzon, Elias Collado, Aslan Turer, Bashar Hannawi, Jeffrey Geske, Penn Heart, John Symanski, Sanger Heart, Christopher Kramer, Nitasha Sarswat, Ferhaan Ahmad, Jeremy Markowitz, Neal Lakdawala, Sandeep Jani, Marshall Brinkley, Ozlem Bilen, Craig Asher, Sitaramesh Emani, Abhinav Sharma, David Fermin, Melissa Lyle, David Raymer, Andrew Darlington, Christopher Nielsen, Andrew Wang, Sherif Nagueh, Matthew Martinez, Milind Desai, Albree Tower‐Rader, Jacob Kelly, Alaska Heart, Florian Rader, Sounok Sen, Patrick Bering, Mathew Maurer, Sumeet Mitter, Mark Sherrid, Timothy Wong, Zainal Hussain, Sara Saberi, Srihari Naidu, Jorge Silva Enciso

**Affiliations:** ^1^ School of Cardiovascular and Metabolic Health University of Glasgow United Kingdom; ^2^ Oregon Health and Science University Portland OR; ^3^ University of Missouri Kansas City Healthcare Institute for Innovations in Quality and Saint Luke’s Mid America Heart Institute Kansas City MO; ^4^ Complexo Hospitalario Universitario A Coruña, INIBIC, CIBERCV‐ISCIII A Coruña Spain; ^5^ Leviev Heart Center, Sheba Medical Center Ramat‐Gan and Tel Aviv University Ramat‐Gan Israel; ^6^ Hospital CUF Descobertas Lisbon Portugal; ^7^ Northwestern University Feinberg School of Medicine Chicago IL; ^8^ Cardiovascular Division Brigham and Women’s Hospital, Harvard Medical School Boston MA; ^9^ Charité Campus Virchow‐Klinikum Berlin Germany; ^10^ Hospital Universitario Puerta de Hierro de Majadahonda, IDIPHISA, CIBERCV, and Centro Nacional de Investigaciones Cardiovasculares (CNIC) Madrid Spain; ^11^ Assistance Publique Hôpitaux de Paris, Département de Cardiologie, Hôpital Européen Georges‐Pompidou Paris France; ^12^ Division of Cardiology, Department of Medicine Massachusetts General Hospital, Harvard Medical School Boston MA; ^13^ Heart Failure and Biomarker Trials Baim Institute for Clinical Research Boston MA; ^14^ Beijing Anzhen Hospital, Capital Medical University Beijing China; ^15^ Lahey Hospital and Medical Center Burlington MA; ^16^ Department of Cardiology Erasmus Medical Center, Cardiovascular Institute, Thoraxcenter Rotterdam The Netherlands; ^17^ Meyer Children’s Hospital, Istituto di Ricovero e Cura a Carattere Scientifico (IRCCS) Florence Italy; ^18^ National Institute of Cardiololgy Warsaw Poland; ^19^ University of Pennsylvania Perelman School of Medicine Philadelphia PA; ^20^ Section of Forensic Genetics, Department of Forensic Medicine, Faculty of Health and Medical Sciences University of Copenhagen Denmark; ^21^ Department of Cardiology Copenhagen University Hospital Rigshospitalet Copenhagen Denmark; ^22^ Hypertrophic Cardiomyopathy Patient Author Zwolle The Netherlands; ^23^ JV Cardiology Prague Czech Republic; ^24^ Radcliffe Department of Medicine University of Oxford United Kingdom; ^25^ Cytokinetics, Incorporated South San Francisco CA; ^26^ University of California San Francisco San Francisco CA

**Keywords:** aficamten, cardiac myosin inhibitor, hypertrophic cardiomyopathy, Cardiomyopathy, Heart Failure, Hypertrophy

## Abstract

**Background:**

Aficamten, a novel cardiac myosin inhibitor, reversibly reduces cardiac hypercontractility in obstructive hypertrophic cardiomyopathy. We present a prespecified analysis of the pharmacokinetics, pharmacodynamics, and safety of aficamten in SEQUOIA‐HCM (Safety, Efficacy, and Quantitative Understanding of Obstruction Impact of Aficamten in HCM).

**Methods and Results:**

A total of 282 patients with obstructive hypertrophic cardiomyopathy were randomized 1:1 to daily aficamten (5–20 mg) or placebo between February 1, 2022, and May 15, 2023. Aficamten dosing targeted the lowest effective dose for achieving site‐interpreted Valsalva left ventricular outflow tract gradient <30 mm Hg with left ventricular ejection fraction (LVEF) ≥50%. End points were evaluated during titration (day 1 to week 8), maintenance (weeks 8–24), and washout (weeks 24–28), and included major adverse cardiac events, new‐onset atrial fibrillation, implantable cardioverter‐defibrillator discharges, LVEF <50%, and treatment‐emergent adverse events. At week 8, 3.6%, 12.9%, 35%, and 48.6% of patients achieved 5‐, 10‐, 15‐, and 20‐mg doses, respectively. Baseline characteristics were similar across groups. Aficamten concentration increased by dose and remained stable during maintenance. During the treatment period, LVEF decreased by −0.9% (95% CI, −1.3 to −0.6) per 100 ng/mL aficamten exposure. Seven (4.9%) patients taking aficamten underwent per‐protocol dose reduction for site‐interpreted LVEF <50%. There were no treatment interruptions or heart failure worsening for LVEF <50%. No major adverse cardiovascular events were associated with aficamten, and treatment‐emergent adverse events were similar between treatment groups, including atrial fibrillation.

**Conclusions:**

A site‐based dosing algorithm targeting the lowest effective aficamten dose reduced left ventricular outflow tract gradient with a favorable safety profile throughout SEQUOIA‐HCM.

**Registration:**

URL: https://www.clinicaltrials.gov; Unique Identifier: NCT05186818.

**Nonstandard Abbreviations and Acronyms**
AE: adverse event
CYP: cytochrome P450
EXPLORER‐HCM: A Phase 3, Randomized, Double‐Blind, Placebo‐Controlled Study to Evaluate the Efficacy and Safety of Mavacamten (MYK‐461) in Subjects With Symptomatic Obstructive Hypertrophic Cardiomyopathy
HCM: hypertrophic cardiomyopathy
LS: least squares
LVOT‐G: left ventricular outflow tract gradient
oHCM: obstructive hypertrophic cardiomyopathy
REDWOOD‐HCM: Randomized Evaluation of Dosing with CK‐3773274 in Obstructive Outflow Disease in HCM
SEQUOIA‐HCM: Safety, Efficacy, and Quantitative Understanding of Obstruction Impact of Aficamten in HCM

**Clinical Perspective**

**What Is New?**

- More than 80% of patients randomized to aficamten reached 1 of the 2 highest doses (15 or 20 mg) within the first 8 weeks of treatment.
- There were no major adverse cardiovascular events associated with aficamten treatment in SEQUOIA‐HCM (Safety, Efficacy, and Quantitative Understanding of Obstruction Impact of Aficamten in HCM).
- Over the 24‐week treatment period, reduced left ventricular ejection fraction <50% occurred in a small number of patients treated with aficamten; no instances were associated with adverse clinical consequences, and all instances were reversible and effectively managed by dose reduction without the need for treatment interruption.

**What Are the Clinical Implications?**

- Aficamten has a favorable pharmacological profile that enables up‐titration, as frequently as every 2 weeks, with clinical benefits evident at the lowest dose.
- While aficamten was effective at relieving obstruction by reducing cardiac contractility, its effects are rapidly reversible, which implies that if needed, the drug may be stopped with rapid reversal of pharmacodynamic effects, thereby minimizing the risk of prolonged reduction in left ventricular ejection fraction.

Cardiac myosin inhibitors are a new class of drugs that target left ventricular (LV) hypercontractility, which decreases LV outflow gradient in obstructive hypertrophic cardiomyopathy (oHCM) and improves patients' health status (symptoms, exercise capacity, and quality of life).^1^, ^2^ However, exposure to cardiac myosin inhibitors presents the potential for excessive reduction of left ventricular ejection fraction (LVEF) and symptomatic heart failure. This has been seen with mavacamten^3^, ^4^, ^5^ and resulted in the implementation of a risk evaluation and mitigation strategy by the US Food and Drug Administration, the components of which require patient and provider education and monitoring of concomitant medications, heart failure symptoms, and LV function by echocardiography.^6^

Aficamten is a next‐in‐class selective inhibitor of cardiac myosin that acts by binding directly to cardiac myosin at a distinct allosteric binding site, stabilizing a weakly bound state of myosin and decreasing the number of active actin–myosin cross‐bridges. Aficamten was engineered with the goal of achieving specific pharmacological properties, namely: (1) a half‐life that would enable dose escalation as frequently as every 2 weeks; (2) a shallow exposure–response relationship that would result in small reductions in LVEF for each dose increment; (3) rapid reversibility of the pharmacodynamic effect that would enable dose reduction without treatment interruption; and (4) low potential for drug–drug interactions, ensuring multiple elimination pathways that would lower the potential for drug–drug interactions. Desirable pharmacological properties were demonstrated in the phase 1 study of healthy volunteers whereby, after oral administration, aficamten was readily absorbed with maximum plasma concentrations occurring at ≈2 hours after dosing.^7^

Aficamten pharmacokinetics are generally linear with respect to dose and time over a wide range of single and multiple daily doses. Consistent with a terminal elimination half‐life of 75 to 85 hours, aficamten reaches steady‐state plasma exposures in most individuals after ≈2 weeks of daily administration. Aficamten elimination is primarily via metabolism by multiple cytochrome P450 (CYP) enzymes, including CYP2C9, CYP2D6, and CYP3A, thus mitigating the potential for clinically significant increases in aficamten exposure due to concomitantly administered CYP inhibitors or genetically mediated poor metabolizers.^7^, ^8^ These features were confirmed in patients with hypertrophic cardiomyopathy (HCM) who participated in REDWOOD‐HCM (Randomized Evaluation of Dosing with CK‐3773274 in Obstructive Outflow Disease in HCM; NCT04219826), the 10‐week phase 2 dose‐finding study of aficamten.^9^, ^10^

In the pivotal phase 3 trial, SEQUOIA‐HCM (Safety, Efficacy, and Quantitative Understanding of Obstruction Impact of Aficamten in HCM; NCT05186818), treatment with aficamten over 24 weeks improved exercise capacity, heart failure symptoms, and New York Heart Association functional class in patients with oHCM.^11^, ^12^

The dosing regimen in SEQUOIA‐HCM was designed to optimize both efficacy and safety by individually targeting the lowest effective dose in each patient to reduce provoked left ventricular outflow tract gradients (LVOT‐G) to <30 mm Hg, while maintaining LVEF ≥50%. This prespecified analysis from SEQUOIA‐HCM is designed to describe the patient characteristics, efficacy, and safety of aficamten at each dose used in the trial and by treatment phase within the trial, and plasma drug concentration and dose in relation to LVEF. Collectively, these analyses aim to test whether the pharmacological properties engineered into aficamten (half‐life of ≈3.4 days, shallow dose–response relationship, minimal drug interactions, and rapid reversibility) translate into clinical benefit while maintaining patient safety.

## Methods

### Data Availability

Qualified researchers may submit a request containing the research objectives, end points/outcomes of interest, statistical analysis plan, data requirements, publication plan, and qualifications of the researcher(s). Requests are reviewed by a committee of internal and external advisors. If approved, necessary statistical outputs will be provided to address the research question under the terms of a data‐sharing agreement. Requests will be considered after applications for marketing authorization in the United States and Europe have been reviewed and final decisions rendered. Requests may be submitted to medicalaffairs@cytokinetics.com.

### Study Organization and Oversight

SEQUOIA‐HCM was a multicenter, randomized, double‐blind, placebo‐controlled, phase 3 trial conducted at 26 sites in the United States, 12 in China, and 44 in Europe and Israel. The trial design has been described previously.^11^ Details of the SEQUOIA‐HCM investigators are provided in the appendix, and the study protocol and statistical analysis plan are available in Data S1 and S2, respectively. A diverse steering committee including representation from all enrolling countries, a patient representative, and with 20% female members, in collaboration with Cytokinetics, designed the trial, selected trial centers, and oversaw the conduct and monitoring of the trial (performed by a contract research organization, ICON, Dublin, Ireland). An institutional review board or independent ethics committee at each trial center approved the protocol. All patients provided written informed consent, and the study was carried out in accordance with the provisions of the Declaration of Helsinki and the International Council for Harmonization Guideline for Good Clinical Practice. An independent data monitoring committee had access to unblinded data for monitoring. Study personnel remained blinded to treatment assignments, dosing, and echocardiogram results through database lock. After database lock, a complete database copy was transferred to the Brigham and Women's Hospital Clinical Trials Outcomes Center, where statistical analyses were performed by an independent statistician. The authors, who had full access to the data, vouch for the accuracy and completeness of the data and data analyses.

### Study Population

The trial enrolled patients with symptomatic oHCM taking stable background HCM medical therapy that was individually optimized based on local practice. Key inclusion criteria were age 18 to 85 years, New York Heart Association class II or III, echocardiogram core laboratory–determined severe obstruction (resting and Valsalva LVOT‐G≥30 mm Hg and ≥50 mm Hg, respectively) and LVEF ≥60% at screening, and peak oxygen uptake ≤90% of age‐ and sex‐predicted with respiratory exchange ratio ≥1.05. Additional criteria are provided in Data S1. Steps taken to increase diversity among trial participants included inclusion of sites with high patient diversity, update of peak oxygen uptake inclusion criteria from <80 to ≤90% age‐ and sex‐predicted maximum criteria to reduce potential bias, and emphasis on diversity of patient population in communication with investigators.

### Study Drug Dosing Procedures

**Figure 1:**
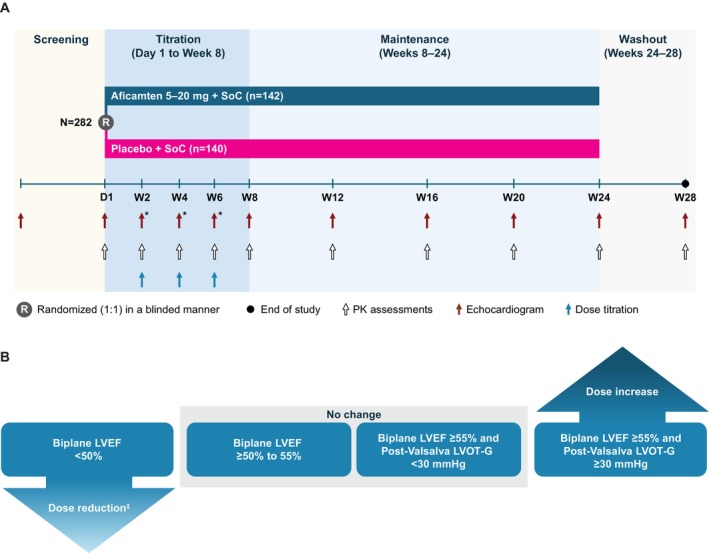
Study design illustrating (A) visit schedule* and (B) dose titration scheme based on echocardiographic criteria.^†^ *Focused echocardiogram. ^†^Patients receiving aficamten start at a dose of 5 mg once daily (dose 1) and may receive up to 4 escalating doses (10, 15, or 20 mg once daily) over the initial 6 weeks of the trial if they continue to meet the escalation criteria or will stop at their current dose when the escalation criteria are not met. ^‡^Dose escalation is not permitted in patients after an aficamten dose reduction. Patients taking aficamten 5 mg and with LVEF <50% will receive placebo. LVEF indicates left ventricular ejection fraction; LVOT‐G, left ventricular outflow tract gradient; PK, pharmacokinetic; SoC, standard of care; and W, week.

Screening, randomization, and patient disposition are included in Data S1 and S2, Figure S1, and were previously described.^12^ The study schema with the dose titration algorithm is shown in Figure 1. Randomization occurred 1:1 to either aficamten or placebo, which was initiated and administered once daily with or without food. Aficamten was started at 5 mg, with 3 opportunities (weeks 2, 4, and 6) for dose escalation in 5 mg increments to a maximum dose of 20 mg. Dose adjustment was based on site‐read echocardiographic LVEF and Valsalva LVOT‐G (Figure 1B). Values were input into an interactive web response system by an unmasked echocardiologist to preserve blinding of treatment assignments. Doses were increased only if the Valsalva LVOT‐G was ≥30 mm Hg and the LVEF was ≥55%. If the LVEF was <50%, the study drug dose was decreased, and no further dose escalation was permitted. If the LVEF was measured at the site as <40% at any period, the investigator and medical monitor were to be contacted and unmasked to the echocardiogram results, and the drug was to be interrupted. Other than in this setting, the site study team and sponsor remained masked to echocardiographic findings throughout the study. All echocardiograms were subsequently analyzed asynchronously in a blinded and standardized fashion by the echocardiography core laboratory, and these data were used for primary reporting purposes. Dosing decisions were based on the site‐read echocardiograms, and the pharmacodynamic effects of aficamten on LVEF were measured according to the core laboratory–measured variables. As such, dosing is reported using the site‐read echocardiograms, and efficacy is reported according to the core laboratory echocardiograms. Plasma concentrations of aficamten were obtained before dosing and 2 hours after dosing at study visits.

### Study End Points

Doses achieved, as well as safety and efficacy by dose, were divided into 3 distinct phases: the titration phase (day 1 to week 8), maintenance phase (week 8+1 day to week 24), and washout phase (week >24 to week 28). The treatment effect of aficamten was analyzed according to dose at week 8. Safety end points included incidence of major adverse cardiac events (cardiovascular death, cardiac arrest, nonfatal stroke, nonfatal myocardial infarction, cardiovascular hospitalization), new‐onset atrial fibrillation, appropriate implantable cardioverter‐defibrillator discharges, core laboratory–measured LVEF <50%, and treatment‐emergent adverse events (AEs). While core laboratory LVEF <50% was reported independently, the clinical consequences related to this finding were specifically evaluated and defined as a core laboratory LVEF <50% with either signs and symptoms of heart failure or >30% increase in NT‐proBNP (N‐terminal pro‐B‐type natriuretic peptide) from last assessment. Any core laboratory LVEF <40% was considered an important finding independent from the clinical or laboratory findings. Potential rebound during the washout phase was assessed in a blinded fashion and defined as the report of a cardiac AE in the presence of more severe LVOT obstruction, worsening symptoms (New York Heart Association ≥1 class increase or Kansas City Cardiomyopathy Questionnaire–Clinical Summary Score >15‐point decrease), and a ≥ 30% increase in NT‐proBNP relative to baseline.

### Statistical Analysis

Baseline characteristics were summarized using means and SDs or medians and interquartile ranges for continuous normal and right‐skewed variables, respectively, with categorical variables summarized using counts and percentages. Differences in baseline characteristics were compared using ANOVA, the Kruskal–Wallis test, and Pearson's χ^2^ test, respectively. Within‐group changes over time were summarized using paired *t* tests. Between‐group comparisons of changes over time, as well as associations between aficamten exposure and LVEF changes, were assessed using linear regression models, adjusted for baseline values as well as randomization stratification variables (ie, β‐blocker usage, cardiopulmonary exercise testing modality). Pairwise correlations were assessed using Pearson's correlation. No adjustments were made for multiple comparisons, and *P* values <0.05 were considered statistically significant. Analysis was performed by Brigham and Women's Hospital Clinical Trials Outcomes Center using Stata version 16.1 (StataCorp, College Station, TX).

## Results

The proportions of final titrated doses at week 8 were 3.6%, 12.9%, 35%, and 48.6% for 5, 10, 15, and 20 mg, respectively. Baseline characteristics were similar across dose groups, except for lower baseline LVOT‐G in patients requiring lower aficamten doses who were more frequently on ≥2 background medications (Table 1 and Table S1). There were no major adverse cardiovascular events associated with aficamten treatment in SEQUOIA‐HCM. Serious AEs occurred in 8 patients (5.6%) in the aficamten group and 13 patients (9.3%) in the placebo group, none of which were considered by the investigators to be related to the study drug.^12^

**Table 1:** Baseline Characteristics According to Final Dose of Aficamten at Week 8*

	Placebo (n=140)	5 mg (n=5)	10 mg (n=18)	15 mg (n=49)	20 mg (n=68)
Age, y	59.0±13.4	57.2±9.1	55.8±12.7	62.2±12.1	58.1±12.7
Female sex	59 (42.1)	2 (40.0)	6 (33.3)	23 (46.9)	25 (36.8)
Race					
Asian	25 (17.9)	2 (40.0)	4 (22.2)	10 (20.4)	12 (17.6)
Black	0 (0.0)	0 (0.0)	0 (0.0)	0 (0.0)	3 (4.4)
Other†	0 (0.0)	0 (0.0)	0 (0.0)	1 (2.0)	1 (1.5)
White	115 (82.1)	3 (60.0)	14 (77.8)	38 (77.6)	52 (76.5)
Geographic region					
China	22 (15.7)	1 (20.0)	1 (5.6)	10 (20.4)	11 (16.2)
North America	45 (32.1)	4 (80.0)	8 (44.4)	18 (36.7)	19 (27.9)
Rest of world	73 (52.1)	0 (0.0)	9 (50.0)	21 (42.9)	38 (55.9)
Medical history					
Hypertension	70 (50.0)	2 (40.0)	10 (55.6)	28 (57.1)	34 (50.0)
Known HCM‐causing gene mutation	25 (17.9)	2 (40.0)	3 (16.7)	8 (16.3)	11 (16.2)
Positive family history of HCM	34 (24.3)	1 (20.0)	5 (27.8)	17 (34.7)	17 (25.0)
Implantable cardioverter defibrillator	17 (12.1)	2 (40.0)	4 (22.2)	4 (8.2)	12 (17.6)
Permanent atrial fibrillation	1 (0.7)	0 (0.0)	0 (0.0)	1 (2.0)	1 (1.5)
Paroxysmal atrial fibrillation	20 (14.3)	0 (0.0)	3 (16.7)	12 (24.5)	6 (8.8)
Coronary artery disease	16 (11.4)	1 (20.0)	2 (11.1)	7 (14.3)	9 (13.2)
Diabetes	9 (6.4)	0 (0.0)	2 (11.1)	2 (4.1)	10 (14.7)
Vital signs at baseline					
SBP, mm Hg	126±16	131±20	120±15	126±16	125±16
DBP, mm Hg	74±11	73±15	74±11	74±9	76±11
Resting heart rate, beats/min	70.7±13.3	62.4±4.4	68.3±11.8	67.3±8.7	69.8±13.0
BMI, kg/m^2^	28.2±3.7	27.7±4.2	26.4±4.0	28.5±3.3	28.2±3.9
Background HCM therapy					
β blocker	87 (62.1)	5 (100.0)	10 (55.6)	31 (63.3)	40 (58.8)
Calcium channel blocker	36 (25.7)	1 (20.0)	3 (16.7)	17 (34.7)	24 (35.3)
Disopyramide	20 (14.3)	1 (20.0)	5 (27.8)	3 (6.1)	7 (10.3)
≥2 Background medications	31 (22.1)	3 (60.0)	5 (27.8)	7 (14.3)	
Baseline study assessments					
Baseline KCCQ Clinical Summary Score	74±18	68±26	75±19	77±20	75±17
Baseline NYHA Class II	106 (75.7)	3 (60.0)	16 (88.9)	33 (67.3)	54 (79.4)
Baseline NT‐proBNP, pg/mL	692 (335, 1795)	1133 (992, 1475)	338 (283, 674)	871 (428, 1505)	962 (511, 2085)
Baseline hs‐cTnI, ng/L	12 (8, 25)	12 (6, 234)	10 (5, 17)	13 (7, 24)	16 (8, 38)
Peak oxygen uptake, mL/kg per min	18.6±4.5	18.7±2.9	18.6±3.9	18.2±4.1	18.3±4.9
Echocardiographic parameters (core laboratory)					
LVEF at baseline, %	75±6	71±12	76±5	75±5	75±5
Peak LVOT‐G at rest	55±32	29±13	45±21	56±24	58±30
Peak LVOT‐G post‐Valsalva	83±33	51±24	71±29	84±26	88±35
LV maximal wall thickness, cm	2.10±0.30	2.42±0.74	1.94±0.22	2.04±0.26	2.11±0.28
Treatment‐emergent serious adverse events	13 (9.3)	0 (0.0)	1 (5.6)	1 (2.0)	5 (7.4)
Congenital, familial and genetic disorders	1 (0.7)	0 (0.0)	0 (0.0)	1 (2.0)	2 (2.9)
Hypertrophic cardiomyopathy	1 (0.7)	0 (0.0)	0 (0.0)	1 (2.0)	2 (2.9)
Cardiac disorders	6 (4.3)	0 (0.0)	1 (5.6)	0 (0.0)	1 (1.5)
Thalassemia	0 (0.0)	0 (0.0)	0 (0.0)	0 (0.0)	0 (0.0)
Arrhythmia supraventricular	0 (0.0)	0 (0.0)	0 (0.0)	0 (0.0)	1 (1.5)
Atrial fibrillation	1 (0.7)	0 (0.0)	1 (5.6)	0 (0.0)	0 (0.0)
Acute coronary syndrome	1 (0.7)	0 (0.0)	0 (0.0)	0 (0.0)	0 (0.0)
Acute myocardial infarction	1 (0.7)	0 (0.0)	0 (0.0)	0 (0.0)	0 (0.0)
Cardiac failure congestive	1 (0.7)	0 (0.0)	0 (0.0)	0 (0.0)	0 (0.0)
Sinoatrial block	1 (0.7)	0 (0.0)	0 (0.0)	0 (0.0)	0 (0.0)
Ventricular fibrillation	1 (0.7)	0 (0.0)	0 (0.0)	0 (0.0)	0 (0.0)
Safety events					
Any core LVEF <50%	1 (0.7)	1 (20.0)	0 (0.0)	2 (4.1)	2 (2.9)

Baseline characteristics are summarized using means and SDs, medians and IQRs, and counts and percentages, as appropriate. BMI indicates body mass index; DBP, diastolic blood pressure; HCM, hypertrophic cardiomyopathy; hs‐cTnI, high‐sensitivity cardiac troponin I; IQR, interquartile range; KCCQ, Kansas City Cardiomyopathy Questionnaire; LV, left ventricular; LVEF, left ventricular ejection fraction; LVOT‐G, left ventricular outflow tract gradient; NT‐proBNP, N‐terminal pro‐B‐type natriuretic peptide; NYHA, New York Heart Association; and SBP, systolic blood pressure.

* 2 patients withdrew from the aficamten group before completing week 8.

† “Other” category: 1 patient is "Hispanic" 15mg dose; 1 patient is "Unknown" 20mg dose group.

### Titration Phase

**Figure 2:**
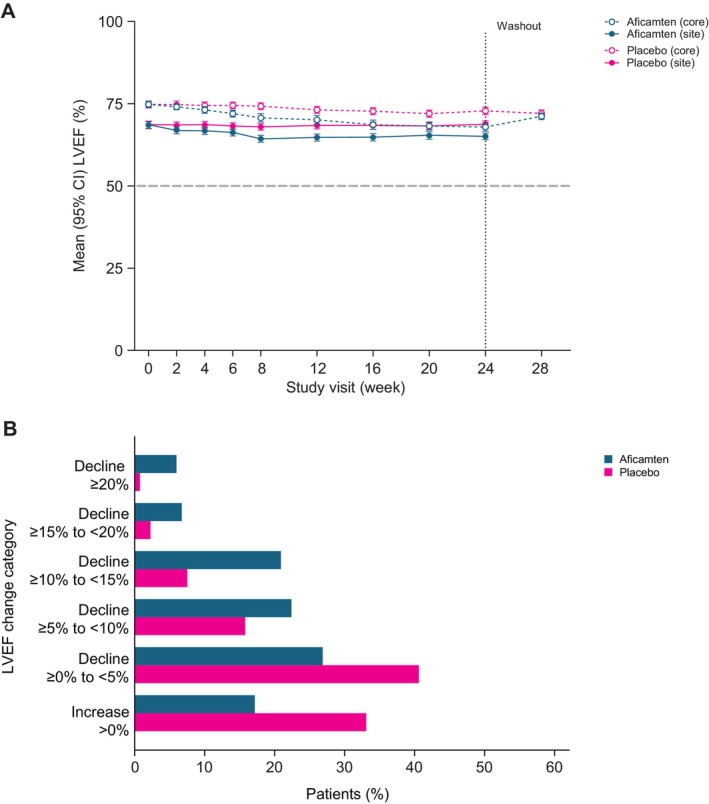
Effect of treatment on core laboratory‐ and site‐read measurements of LVEF over the study period* (A) and categorical changes in LVEF during the study phase (B). LVEF indicates left ventricular ejection fraction. *Data were analyzed using core‐read echocardiograms performed at each study visit.

Dose increases were paralleled by a modest and linear decrease in LVEF. The least squares (LS) mean reductions (95% CI) in LVEF versus placebo were − 0.7% (−1.8 to 0.4; nominal *P*=0.20) at week 2, −1.4% (−2.5 to −0.2; *P*=0.023) at week 4, −2.4% (−3.6 to −1.2; *P*<0.001) at week 6, and −3.5% (−4.9 to −2.2; *P*<0.001) at week 8 (Figure 2A). Efficacy, in terms of reduction in Valsalva LVOT‐G, was evident after 2 weeks compared with placebo, with steep reductions in the degree of obstruction as doses were escalated. At week 2 and week 8, the LS mean differences versus placebo were −21 mm Hg (−27 to −13; *P*<0.001) and −52 mm Hg (−58 to −45; *P*<0.001), respectively. Dose escalation during the titration phase was not required at 15.7% of visits due to achieving Valsalva LVOT‐G <30 mm Hg and at 1.3% of visits due to an LVEF <55% (Table S2).

### Maintenance Phase

**Figure 3:**
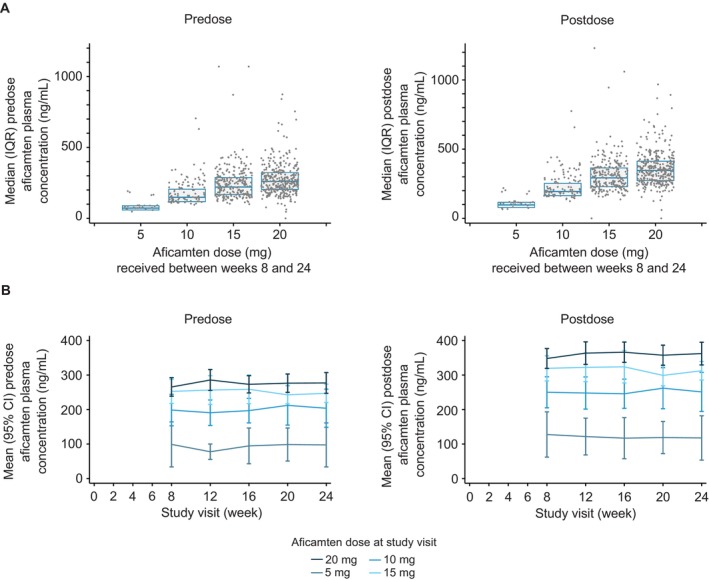
Predose and postdose plasma concentration of aficamten during the maintenance phase. (A) All available individual concentration measurements between weeks 8 and 24 independent of study week (median and IQR) and (B) mean (95% CI) by dose and study visit between weeks 8 and 24. IQR indicates interquartile range.

The final doses at week 24 were similar to those achieved at week 8 and were 5 mg in 5 (3.6%), 10 mg in 21 (15.3%), 15 mg in 48 (35.0%), and 20 mg in 63 (46.0%) patients. Each dose level was associated with incrementally higher plasma drug concentrations of aficamten in a dose‐dependent manner (median [interquartile range] predose concentrations for weeks 8–24 were 85 ng/mL [73–92] for 5 mg, 171 ng/mL [135–243] for 10 mg, 236 ng/mL [171–296] for 15 mg, and 260 ng/mL [203–324] for 20 mg; Figure 3A), and in each dose group, postdose and predose plasma drug concentrations remained stable over the duration of the maintenance phase and the postdose–predose ratio was small (≈1.3). At weeks 8 and 24, mean (±SD) plasma predose concentrations were as follows: 5 mg (99 ng/mL±52 and 98 ng/mL±51); 10 mg (199 ng/mL±89 and 204 ng/mL±118); 15 mg (253 ng/mL±122 and 247 ng/mL±91); and 20 mg (265 ng/mL±111 and 277 ng/mL±119), respectively (Figure 3B).

Between weeks 8 and 24, there was no significant change in core LVEF compared with placebo, −2.9% (95% CI, –4.2 to −1.5) for aficamten; −1.7% (95% CI, –2.8 to −0.5) for placebo; −1.2% (95% CI, –3.0 to 0.5; *P*=0.17) for LS mean difference. Overall, the LS mean change in LVEF from baseline to week 24 was −6.8% (95% CI, –8.1 to −5.5) for aficamten and −2.0% (95% CI –3.1 to −1.0) for placebo (LS mean difference, −4.8% [95% CI, –6.4 to −3.1]; *P*<0.001]). Corresponding categorical reductions in LVEF at the end of the treatment phase (week 24 echocardiogram compared with baseline) are shown in Figure 2B and reflect a normalization of LVEF from a hypercontractile state. Aficamten‐treated patients with the largest excursions in LVEF from baseline (≥20%) all had baseline LVEF >75% with no LVEF <50% events at any visit. According to the titration algorithm and site‐interpreted echocardiograms, 7 patients taking aficamten and 1 patient taking placebo met the criteria for a dose reduction during the maintenance phase: 1 at week 8, 1 at week 12, 4 at week 16, and 1 at week 20. Aficamten doses at the time of down‐titration were 15 mg in 4 patients and 20 mg in 3 patients.

### Washout Phase

After a 4‐week washout period, all pharmacodynamic measures returned toward baseline, and LVEF was not different from placebo (core laboratory LVEF LS mean difference, −0.8% [95% CI, −2.1 to 0.5]; *P*=0.21).

### Relationship Between Aficamten and LVEF

**Figure 4:**
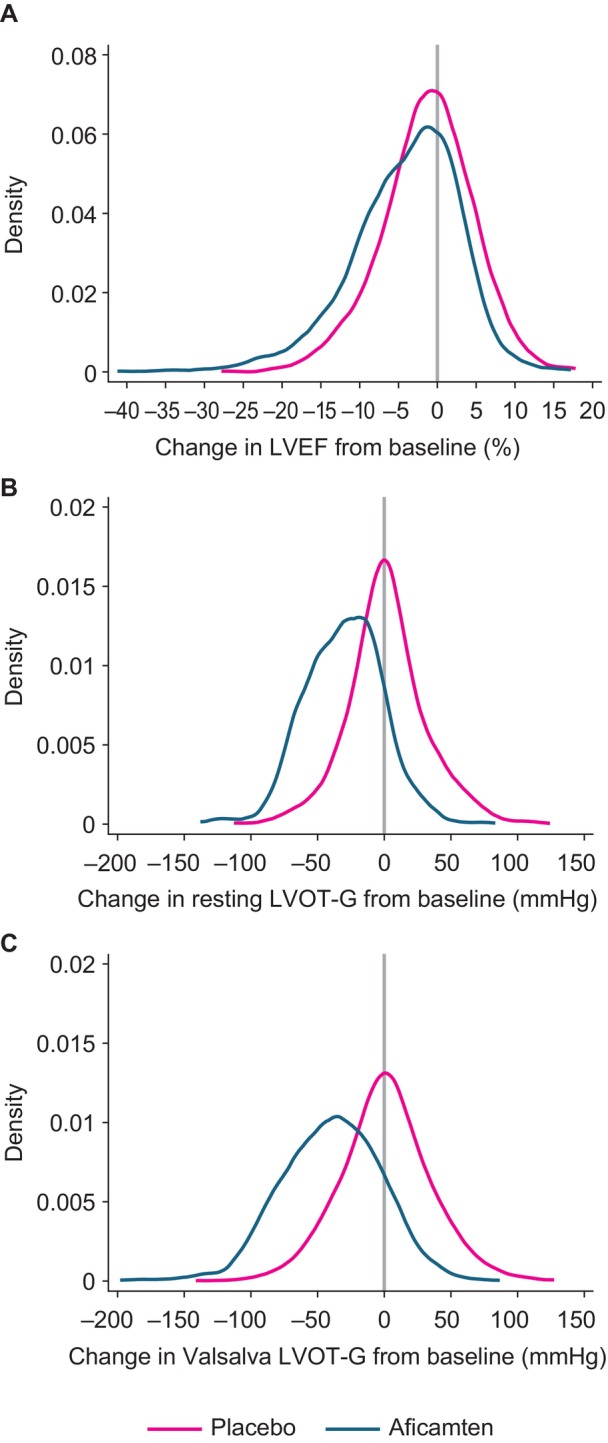
Kernel density estimate plots illustrating the distribution of change in echocardiographic parameters, (A) LVEF, (B) resting LVOT‐G, and (C) Valsalva LVOT‐G, from baseline at every study visit in the aficamten (blue) and placebo (pink) groups.* LVEF indicates left ventricular ejection fraction; and LVOT‐G, left ventricular outflow tract gradient. *Data were analyzed using all study visits between weeks 2 and 24 with core analysis of echocardiographic parameters. The mean (±SD) difference between the groups was −2.8% (±6.6) for LVEF (**A**), −13 (±33) mm Hg for resting LVOT‐G (**B**), and −18 (±40) mm Hg for Valsalva LVOT‐G (**C**). The gray vertical line denotes no change in echocardiographic parameter from baseline.

The distribution of changes in LVEF and LVOT‐G from day 1 is shown in Figure 4. Seven aficamten patients (4.9%) underwent a per‐protocol dose reduction because of site‐read LVEF <50%. Characteristics of patients with site‐ or core‐reported LVEF <50% are summarized in Table 2, with individual patient profiles available in Figure S2. In patients undergoing a dose reduction, 2 patients were receiving verapamil background therapy, and 5 were receiving β blockers (metoprolol [2], nadolol, propranolol, or bisoprolol). The placebo‐treated patient with overlapping site and core laboratory LVEF <50% was noted to have symptoms of heart failure and peripheral edema. One patient with an LVEF <40% by core laboratory measurement was interpreted by the site as having an LVEF between 40% and 50%; this patient was managed successfully with a per‐protocol dose reduction resulting in LVEF to >50% by the next visit and was asymptomatic with a Kansas City Cardiomyopathy Questionnaire–Clinical Summary Score of 98 at the time of LVEF <40%.

**Table 2:** Characteristics of Patients With Any Echocardiogram Reporting LVEF <50%

Core laboratory‐reported LVEF <50%														
Age and sex	Aficamten dose (mg/d) or placebo	Age at HCM diagnosis, y	Background treatment	Baseline MWT, cm	Baseline core LVEF, %	Study week with lowest core LVEF	Lowest core LVEF, %	Matching site LVEF, %	Site Valsalva LVOT‐G, mmHg	NYHA class	KCCQ‐CSS	Change from baseline NT‐proBNP, ng/dL	Down‐titration, mg	Next visit LVEF core
30M	20	11	BB	2.2	65	8	48	62	9	1	100	−535	N/A	56
57F	5	51	BB	2.0	56	24	46	60	23	2	74	−372	N/A	51
72F	15	65	CCB	1.8	80	20	48	52	5	1	100	−403	N/A	52
57F	20	57	None	2.3	84	16	43	59	6	1	100	−921	N/A	72
75F	Placebo	74	None	2.3	53	6	48	45	72	3	67	−291	N/A*	53
72F	15	71	CCB	2.4	63	16	34	49	8	2	98	111	15 to 10†	55

Site laboratory‐reported LVEF <50%														
Age and sex	Aficamten dose (mg/d) or placebo	Age at HCM diagnosis, y	Background treatment	Baseline MWT, cm	Baseline core LVEF, %	Study week with lowest site LVEF	Lowest site LVEF, %	Matching core LVEF, %	Site Valsalva LVOT‐G, mm Hg	NYHA class	KCCQ‐CSS	Change from BL in NT‐proBNP, ng/dL	Down‐titration, mg	Next visit LVEF core
41M	20	28	BB+DISO	1.9	70	16	47	59	24	2	75	−1597	20 to 15	54
52M	20	39	BB	2.2	69	16	46	51	4	1	100	−712	20 to 15	60
76F	15	75	BB	1.8	87	16	48	53	24	3	70	−44	15 to 10	52
59M	15	59	BB	2.2	77	12	48	70	7	2	98	−1482	15 to 10	60
54M	15	49	BB+DISO	1.5	76	8	49	72	8	1	89	−162	15 to 10	60
66M	20	64	CCB	2.0	76	20	45	53	9	3	44	−83	20 to 15	61

BB indicates β blocker, CCB, calcium channel blocker; DISO, disopyramide; HCM, hypertrophic cardiomyopathy; KCCQ‐CSS, Kansas City Cardiomyopathy Questionnaire–Clinical Summary Score; LVEF, left ventricular ejection fraction; LVOT‐G, left ventricular outflow tract‐gradient; MWT, maximal wall thickness; NT‐proBNP, N‐terminal pro‐B‐type natriuretic peptide; and NYHA, New York Heart Association.

* Patient had transient reduction in LVEF <50% on the basis of both core and site laboratory assessments.

^†^ COVID‐19 infection preceded LVEF <50% on the basis of both core and site laboratory assessments.

The exposure–response relationship was shallow, with an absolute LVEF reduction (between baseline and week 24) of −0.9% (95% CI, −1.3 to −0.6) per 100 ng/mL aficamten exposure. There was no threshold exposure over the range of doses explored in SEQUOIA‐HCM at which excessive pharmacodynamic effect was noted, and the 5 cases of core laboratory–detected LVEF <50%, including the patient with LVEF <40%, were not clustered at the higher end of the exposures.

Overall, there was modest correlation between site and core laboratory echocardiogram interpretation (*r*=0.34 [95% CI, 0.30–0.37]), with the sites interpreting the LVEF generally lower by 5.3% (95% CI, 5.0–5.6). Conversely, sites tended to measure Valsalva LVOT‐G higher than the core laboratory by 12.5 mm Hg (95% CI, 11.3–13.7), which had a stronger correlation with core laboratory values (Figure S3). Of the 8 patients with site‐determined LVEF <50% and 6 patients with core laboratory–measured LVEF <50%, 2 cases (1 placebo and 1 aficamten) overlapped (Table 2).

### Treatment‐Emergent AEs

Overall, treatment‐emergent AEs occurring in ≥2% of patients were similar in the aficamten and placebo groups (Table S3) except for hypertension and palpitations, which occurred in 11 (7.7%) versus 3 (2.1%) and 10 (7.0%) versus 4 (2.9%) patients in the aficamten and placebo groups, respectively. There were 3 AEs that resulted in treatment interruptions or discontinuations during the maintenance phase: 2 in patients taking placebo (loss of consciousness and acute lymphocytic leukemia) and 1 in a patient taking aficamten (paranoia). There were no AEs of cardiac failure associated with core laboratory LVEF <50% and none that required dose reduction or interruption. There were 10 reported AEs of HCM (signs and symptoms related to preexisting HCM): 6 in the aficamten group and 4 in the placebo group. All 6 occurring in the aficamten treatment group were reported during the washout phase, while the 4 events in the placebo group were reported during the treatment phase. There were 4 AEs of cardiac failure: 2 during the treatment phase (1 placebo and 1 aficamten) and 2 during the washout phase (both aficamten). Atrial fibrillation events were not associated with LVEF <50% events (χ^2^ *P*=0.67 core, *P*=0.62 site).

### Analysis for Potential Rebound During Washout Phase

Of the patients identified with cardiovascular AEs (23 [16.2%] who received aficamten and 9 [6.4%] who received placebo) during the washout phase, 4 (1.4%) met the prespecified criteria for potential rebound. Medical review was conducted in a blinded manner after database lock. Two of the 4 patients meeting the prespecified criteria for potential rebound were identified as having an alternative cause for the AE pathogenesis and likely contributory prespecified criteria (1 participant with a decline in hemoglobin of >2 g/dL and 1 with noncardiac syncope). After unblinding, the patient with syncope was identified as being treated with placebo. Two (0.7%) patients, both in the aficamten group, met the predefined criteria for potential rebound and had the following postdose AEs: HCM (of moderate severity) reported for 1 and dyspnea and palpitations (both of mild severity) reported for the other.

## Discussion

In SEQUOIA‐HCM, patients with oHCM treated with aficamten for 24 weeks achieved significant improvements in exercise capacity, as measured by the primary end point of change from baseline to week 24 in peak oxygen uptake: 1.8 mL/kg per min (95% CI, 1.2–2.3) increase for aficamten versus 0.0 mL/kg per min (95% CI, −0.5 to 0.5) for placebo (LS mean, 1.74 mL/kg per min [95% CI, 1.0–2.4]; *P*<0.001).^12^ Treatment with aficamten was also associated with significant improvements in all 10 secondary end point measures of outflow gradients, heart failure symptoms, and health status. Of note, with aficamten treatment: Kansas City Cardiomyopathy Questionnaire–Clinical Summary Score improved by 7 points (95% CI, 5–10), 58.5% of aficamten‐treated versus 24.3% of placebo‐treated patients had ≥1 New York Heart Association class improvement, Valsalva LVOT‐G decreased by 50 mm Hg (95% CI, 44–57), 49.3% of aficamten‐treated versus 3.6% of placebo‐treated patients achieved Valsalva LVOT‐G<30 mm Hg, NT‐proBNP decreased by 80%, and there was a substantial reduction in time spent eligible for septal reduction therapy (78 fewer days [95% CI, −100 to −56] compared with placebo).^12^ The dosing scheme employed in SEQUOIA‐HCM used local echocardiographic measurements in the absence of plasma drug concentration monitoring, consistent with the planned implementation in clinical practice. The current prespecified analysis demonstrated that aficamten dose escalations were associated with substantial decrements in LVOT‐G, predictable increments in plasma drug concentration, and low rates of LVEF excursions <50%. These findings provide evidence that echocardiographic‐guided dose titration targeting the lowest effective dose of aficamten to reduce the LVOT‐G is safe and effective in patients with oHCM and adds important insights not incorporated within the primary publication of SEQUOIA‐HCM.

An important finding in this analysis was the rapid reduction in LVOT‐G within 2 weeks of treatment initiation, and almost all patients treated with aficamten continued with subsequent stepwise dose escalation during the titration phase without the need for either discontinuation or interruption. Notably, patients who remained on the 5‐mg and 10‐mg doses had no per‐protocol dose reductions over the entire treatment period. Consistent with the results from REDWOOD‐HCM, the plasma concentration of aficamten increased with dose and was stable over time.^9^ The associated on‐target reduction in LVEF was modest, and when down‐titration was required, LVEF recovered rapidly and was not associated with either worsening heart failure symptoms or AEs of heart failure. At the end of treatment, aficamten cessation resulted in increased LVEF and LVOT‐G back to baseline values and was associated with a return of symptoms. This demonstrates reversibility within 4 weeks but was not associated with excessive rebound. Notably, assessment for potential rebound requires differentiation of normal disease variability from excessive rebound, which is inherently challenging in oHCM due to well‐described high baseline intrapatient variability of LVOT gradient and symptoms.^13^

In the era of targeted therapies for oHCM, maintenance of a normal LVEF >50% has developed into a key safety metric.^14^ In the mavacamten trials, 2.5% to 12.5% of patients experienced drug discontinuation due to reduction of LVEF <50%.^3^, ^4^, ^5^ It is important to note that the largest trial of mavacamten (EXPLORER‐HCM [A Phase 3, Randomized, Double‐Blind, Placebo‐Controlled Study to Evaluate the Efficacy and Safety of Mavacamten [MYK‐461] in Subjects With Symptomatic Obstructive Hypertrophic Cardiomyopathy; NCT03470545]) employed additional measures to prevent low LVEF events. First, plasma drug concentration monitoring was required and resulted in either treatment interruption or down‐titration independent of LVEF; second, dose adjustments were based on core laboratory–calculated LVEF and gradients; and third, doses were interrupted in the setting of any LVEF <50%. Mavacamten is available only through implementation of a risk evaluation and mitigation scheme program in the United States with elements to ensure safe use.^6^ In Europe, CYP genotyping of patients is required to determine the appropriate mavacamten dose because the elimination half‐life strongly depends on the CYP2C19 phenotype, with 2% of Europeans and 18% of Asians being slow metabolizers.^15^, ^16^ Data are emerging about the logistics and feasibility of mavacamten use in the real‐world setting.^17^

In contrast, SEQUOIA‐HCM was designed so that physicians at the site were responsible for echocardiographic interpretation and drug dosing without a need for plasma drug monitoring. Rather than treatment interruption for LVEF <50%, the half‐life of aficamten enabled a simple reduction to the next lower dose when LVEF remained >40%. This strategy was effective in all 7 patients undergoing dose reduction per site echocardiogram findings, with increases in site‐read LVEF to >50% at the time of THE next follow‐up. Interestingly, patients treated with aficamten who developed LVEF <50%, either by site or core laboratory measurements, continued to have improved symptoms relative to baseline at that time. Importantly, the reversibility of aficamten was demonstrated by the return to baseline of LVEF after washout. The absence of a measurable pharmacodynamic effect seen in SEQUOIA‐HCM at week 28 is supported by data from REDWOOD‐HCM, where plasma aficamten concentrations were low after just 2 weeks of washout (mean, 31.2 ng/mL±49.4).^9^

The dose‐selection algorithm and implementation protocol employed in SEQUOIA‐HCM were designed for translation into clinical practice, titrating to a treatment effect while maintaining a safety threshold. This approach is familiar to cardiologists and can be likened to other cardiovascular therapies (eg, adjusting hypertension treatments to blood pressure, warfarin dose to international normalized ratio, and heart failure medications to renal function and vital signs). There were no differences in most clinical characteristics of patients with oHCM according to titrated dose, except that the patients achieving the higher doses of 15 and 20 mg had higher resting and Valsalva LVOT‐G at baseline. Additionally, there were no prohibited medications or combination of medications, in SEQUOIA‐HCM, with participating patients receiving typical guideline‐recommended medical therapies for HCM, underlining the generalizability of these findings to the clinical environment. In the SEQUOIA‐HCM trial, differences between site‐ and core‐read echocardiograms impacting safety end points were reported in <1% of all echocardiograms. This suggests that real‐world implementation of aficamten is feasible and can be safely accomplished.

In conclusion, aficamten appeared safe and effective in the treatment of patients with oHCM in SEQUOIA‐HCM. The dosing algorithm employed was associated with rapid reduction of LVOT‐G, with a very low frequency of reversible asymptomatic occurrence of LVEF <50%.

## Sources of Funding

The SEQUOIA‐HCM study was funded by Cytokinetics, Inc.

## Disclosures

Dr Coats has received speaker fees from Alnylam and Roche, and advisory fees from Cytokinetics, Inc. Dr Masri has received consultant/advisor fees from Tenaya; Attralus; Cytokinetics, Inc.; Bristol Myers Squibb; and Ionis; and research grants from Ionis, Akcea, Pfizer, Ultromics, and Wheeler Foundation. Dr Nassif has received research and grant support from AstraZeneca and Cytokinetics, Inc.; and consulting/advisory fees from Vifor and Cytokinetics, Inc. Dr Barriales‐Villa has received consultant/advisor fees from MyoKardia/Bristol Myers Squibb. Dr Arad reports consultant and lecture fees from Bristol Myers Squibb. Dr Cardim has received consultant/advisor fees from Cytokinetics, Inc.; Bristol Myers Squibb; Pfizer; Menarini; Boehringer‐Ingelheim; and Bial. Dr Düngen reports grants from Novartis, CSL Behring, and Cytokinetics, Inc. Dr Garcia‐Pavia has received speakers' bureau fees from Pfizer, AstraZeneca, Novo Nordisk, Ionis, BridgeBio, BMS, Intellia, and Alnylam; consultant/advisor fees from Pfizer; Alnylam; MyoKardia/Bristol Myers Squibb; Cytokinetics, Inc.; Neuroimmune; Intellia; Ionis; BridgeBio; Lexeo; Rocket; Attralus; and AstraZeneca; and research/educational grants to his institution from Pfizer, AstraZeneca, Novo Nordisk, BridgeBio, and Alnylam. Dr Hagège has received consultant/advisor fees from Alnylam, Amicus Therapeutics, Bayer, MyoKardia/Bristol Myers Squibb, Pfizer, and Sanofi Genzyme; and steering committee fees for SEQUOIA‐HCM from Cytokinetics, Inc. Dr Januzzi is funded in part by the Hutter Family Professorship; is a Trustee of the American College of Cardiology; has served as a board member for Imbria Pharmaceuticals; has served as a director at Jana Care; has received grant support from Abbott, Applied Therapeutics, HeartFlow, Innolife, and Roche Diagnostics; has received consulting income from Abbott, Beckman, Bristol Myers Squibb, Boehringer Ingelheim, Janssen, Novartis, Pfizer, Merck, Roche Diagnostics, and Siemens; and has participated in clinical end point committees/data safety monitoring boards for Abbott, AbbVie, CVRx, Intercept, and Takeda. Dr Lee has received research grants through his institution from AstraZeneca, Boehringer Ingelheim, and Roche Diagnostics; and is a member of a clinical end points committee for Bayer, and a trial steering committee for Cytokinetics, Inc. Dr Lewis has received research funding from the National Institutes of Health (R01‐HL 151841, R01‐HL131029, and R01‐HL159514); American Heart Association (15GPSGC‐24 800 006); Amgen; Cytokinetics, Inc.; Applied Therapeutics; AstraZeneca; and SoniVie; honoraria for advisory boards from Pfizer; Merck; Boehringer Ingelheim; Novartis; American Regent; Cyclerion; Cytokinetics, Inc.; and Amgen; and royalties from UpToDate for scientific content authorship related to exercise physiology. Dr Maron has received consultant/advisor fees from Imbria; Edgewise; and BioMarin; and steering committee fees for SEQUOIA‐HCM from Cytokinetics, Inc. Dr Michels has received consultant/advisor fees from Bristol Myers Squibb; Cytokinetics, Inc.; and Pfizer; and research grant funding from Bristol Myers Squibb. Dr Olivotto has received speakers' bureau fees from Bristol Myers Squibb, Amicus, and Genzyme; consultant/advisor fees from Bristol Myers Squibb; Cytokinetics, Inc.; Sanofi Genzyme; Amicus; Bayer; Tenaya; Rocket Pharma; and Lexeo; and research grant funding from Bristol Myers Squibb; Cytokinetics, Inc.; Sanofi Genzyme; Amicus; Bayer; Menarini International; Chiesi; and Boston Scientific. Dr Oreziak has received investigator fees from Cytokinetics, Inc.; and MyoKardia/Bristol Myers Squibb. Dr Owens has received consultant/advisor fees from Cytokinetics, Inc.; Bristol Myers Squibb/MyoKardia; and Pfizer. Dr Spertus is the principal investigator of grants from the National Institutes of Health, Abbott Vascular, and the American College of Cardiology Foundation; is a consultant for Janssen, Novartis, Amgen, MyoKardia, AstraZeneca, Bayer, and Merck; serves on the scientific advisory board of United Healthcare and the board of directors for Blue Cross Blue Shield of Kansas City; owns the copyright to the KCCQ, SAQ, and PAQ; and has an equity interest in Health Outcomes Sciences. Dr Solomon has received consultant/advisor fees from Abbott; Action; Akros; Alnylam; Amgen; Arena; AstraZeneca; Bayer; Boehringer Ingelheim; Bristol Myers Squibb; Cardior; Cardurion; Corvia; Cytokinetics, Inc.; Daiichi‐Sankyo; GSK; Lilly; Merck; MyoKardia; Novartis; Roche; Theracos; Quantum Genomics; Cardurion; Janssen; Cardiac Dimensions; Tenaya; Sanofi‐Pasteur; DiNAQOR; Tremeau; CellProthera; Moderna; American Regent; Sarepta; Lexicon; AnaCardio; Akros; and PureTech Health; and research grants from Actelion; Alnylam; Amgen; AstraZeneca; Bellerophon; Bayer; Bristol Myers Squibb; Celladon; Cytokinetics, Inc.; Eidos; Gilead; GSK; Ionis; Lilly; Mesoblast; MyoKardia; National Institutes of Health/National Heart, Lung, and Blood Institute; NeuroTronik; Novartis; Novo Nordisk; Respicardia; Sanofi Pasteur; Theracos; and US2.AI. Dr Tfelt‐Hansen is a consultant for Leo Pharma, MicroPort, and Johnson and Johnson. M. van Sinttruije is a patient advisory committee member and a SEQUOIA‐HCM steering committee member for Cytokinetics, Inc. Dr Watkins has received consultant/advisor fees from Cytokinetics, Inc; BioMarin; and BridgeBio. Drs Jacoby, German, Heitner, Kupfer, J. D. Lutz, Dr Malik, and L. Meng and A. Wohltman are employees of and hold stock in Cytokinetics, Inc. The remaining authors have no disclosures to report.

## Appendix

### SEQUOIA‐HCM Investigators

Yuhui Zhang, Fuwai Hospital, CAMS & PUMC, China; Haibo Yang, The First Affiliated Hospital of Zhengzhou University, China; Chunli Shao, Peking University Third Hospital, China; Zuyi Yuan, The First Affiliated Hospital of Xi'an Jiaotong University, China; Qingchun Zeng, Nanfang Hospital, China; Xiaodong Li, Shengjing Hospital of China Medical University, China; Chang‐Sheng Ma, Beijing Anzhen Hospital, China; Yushi Wang, The First Hospital of Jinlin University, China; Yan Shu, Sichuan Provincial People's Hospital, China; Mulei Chen, Beijing Chao‐yang Hospital, Capital Medical University, China; Ling Tao, The First Affiliated Hospital of the Air Force Medical University, China; Xinli Li, Jiangsu Province Hospital, China; Jingfeng Wang, Sun Yat‐Sen Memorial Hospital, China; Zaixin Yu, Xiangya Hospital of Central South University, China; Xiang Cheng, Union Hospital affiliated to Tongji Medical College of Huazhong University of Science and Technology, China; Kui Hong, The Second Affiliated Hospital of Nanchang University, China; David Zemanek, Interni klinika kardiologie a angiologie 1. Lekarske fakulty a Vseobecne fakultni nemocnice v Praze, Czech Republic; Henning Bundgaard, Hjertecentret (The Heart Center)–Copenhagen University Hospital/Rigshosptalet, Denmark; Jens Thune, Bispebjerg Hospital, University of Copenhagen, Denmark; Morten Jensen, Aarhus Universitetshospital, Denmark; Jens Mogensen, Aalborg University Hospital, Aalborg Sygehus, Denmark; Albert A. Hagège, Groupement Hospitalier Universitaire Ouest—Hopital Europeen Georges‐Pompidou (HEGP), France; Gilbert Habib, Assistance Publique Hopitaux de Marseille (AP‐HM)—Hopital de La Timone, France; Philippe Charron, CHU Paris‐GH La Pitie Salpetriere‐Charles Foix—Hopital Pitie‐Salpetriere, France; Thibault Lhermusier, Centre Hospitalier Universitaire de Toulouse—Hopital Rangueil, France; Jean‐Noël Trochu, CHU de Nantes—Hopital Nord Laennec, France; Patricia Reant, Centre Hospitalier Universitaire (CHU) du Haut Leveque, France; Damien Logeart, Hopital Lariboisiere, Hospitalier Universitaire Nord, France; Veselin Mitrovic, Kerckhoff‐Klinik, Bad Nauheim, Germany; Tarek Bekfani, Universitatsklinikum Magdeburg, Germany; Frank Edelmann, Charite Universitatsmedizin Berlin, Germany; Tim Seidler, Klinikum der Georg‐August‐Universitaet Goettingen, Germany; Benjamin Meder, Universitatsklinikum Heidelberg, Germany; Paul Christian Schulze, Universitaetsklinikum Jena, Germany; Stephan Stoerk, University of Wurzburg, Comprehensive Heart Failure Center (CHFC), Germany; Tienush Rassaf, Universitaetsklinikum Essen, Germany; Bela Merkely, Semmelweis Egyetem, Varosmajori Sziv es Ergyogyaszati Klinika, Hungary; Donna Zfat‐Zwas, Hadassah Medical Center, Israel; Michael Arad, The Chaim Sheba Medical Center, Israel; Majdi Halabi, Ziv MC, Israel; Offir Paz, Kaplan Medical Center, Israel; Xavier Piltz, The Barzilai Medical Center, Faculty of Health Sciences, Ben Gurion University of the Negev, Israel; Iacopo Olivotto/Mattia Targetti, Careggi University Hospital, Italy; Marco Metra, Azienda Ospedaliera Spedali Civili di Brescia‐Universita degli Studi Di Brescia, Italy; Marco Canepa, Ospedale Policlinico San Martino—IRCCS, Italy; Beatrice Musumeci, Azienda Ospedaliera S'Andrea di Roma—UOC Cardiologia, Italy; Michele Emdin, Fondazione Toscana Gabriele Monasterio Per La Ricerca Medica E Di Sanita Pubblica (Ftgm), Italy; Michelle Michels, Erasmus Medisch Centrum 1, The Netherlands; Ahmad Amin, Amsterdam University Medical Center (Amsterdam UMC), Academic Medical Center (AMC), The Netherlands; Christian Knackstedt, Maastricht University Medical Center (MUMC), The Netherlands; Artur Oreziak, Narodowy Instytut Kardiologii Stefana kard, Wyszynskiego Panstwowy Instytut Badawczy, Poland; Wojciech Wojakowski, Kardio Brynow Sp. z o.o., Poland; Dariusz Dudek, Krakowskie Centrum Diagnostyczno‐Kliniczne, Poland; Alexandra Toste, Hospital Da Luz, Portugal; José Mesquita Bastos, CH Baixo Vouga—Aveiro, Portugal; Roberto Barriales‐Villa, Complejo Hospital Universitario A Coruna, Spain; Pablo Garcia Pavia, Hospital Universitario Puerta de Hierro de Majadahonda, Spain; Juan Ramón Gimeno Blanes, Hospital Universitario Virgen de la Arrixaca, Spain; Rafael Jesus Hidalgo Urbano, Hospital Universitario Virgen Macarena‐merge, Spain; Ana Garcia Alvarez, Hospital Clinic i Provincial de Barcelona, Spain; Luis Miguel Rincón Diaz, Hospital Universitario de Salamanca, Spain; Tomas Vicente Ripoll Vera, Hospital Son Llatzer, Spain; Perry Elliott, St Bartholomew's Hospital–Barts Health NHS Trust, United Kingdom; Caroline J. Coats, Gartnaval General Hospital, NHS Greater Glasgow and Clyde, United Kingdom; Rob Cooper, Liverpool Heart and Chest Hospital—Liverpool Heart and Chest Hospital NHS Foundation Trust, United Kingdom; Masliza Mahmod, John Radcliffe Hospital—Oxford University Hospitals NHS Foundation Trust, United Kingdom; William Bradlow, Queen Elizabeth Hospital Birmingham–University Hospitals Birmingham NHS Foundation Trust, United Kingdom; Antonis Pantazis, Harefield Hospital—Royal Brompton And Harefield NHS Foundation Trust, United Kingdom; Maria Teresa Tome Esteban, St George's Hospital—St George's University Hospitals NHS Foundation Trust, United Kingdom; Ahmad Masri, Oregon Health and Science University, United States; Michael E. Nassif, Saint Luke's Hospital of Kansas City, United States; Ali Marian, Baylor St. Luke's Medical Center, United States; David Owens, University of Washington Medical Center, United States; Matthew M. Y. Lee, Stanford University, United States; Frank McGrew, Baptist Memorial Health Care, United States; Richard Bach, Washington University School of Medicine, United States; Omar Wever‐Pinzon, University of Utah Health Sciences Center, United States; Elias Collado, Holy Cross Hospital, Fort Lauderdale, United States; Aslan Turer, University of Texas Southwestern Medical Center, United States; Bashar Hannawi, Henry Ford Medical Center, United States; Jeffrey Geske, Mayo Clinic, United States; Anjali T. Owens, Penn Heart and Vascular Center—Heart Failure, United States; John Symanski, Sanger Heart & Vascular Institute, Atrium Healthcare, United States; Christopher Kramer, University of Virginia Health System, United States; Nitasha Sarswat, University of Chicago Medicine, United States; Ferhaan Ahmad, University of Iowa Hospitals & Clinics, United States; Theodore P. Abraham, University of California, San Francisco Medical Center, United States; Jeremy Markowitz, University of Minnesota, United States; Neal Lakdawala, Harvard Medical School—Brigham and Women's Hospital (BWH)—The Schuster Family Transplantation Research Center (TRC), United States; Lubna Choudhury, Northwestern Memorial Hospital, United States; Sandeep Jani, MedStar Franklin Square Medical Center, United States; Marshall Brinkley, Vanderbilt University Medical Center (VUMC)—Vanderbilt University Hospital (VUH), United States; Ozlem Bilen, Emory University Hospital, The Emory Clinic, United States; Craig Asher, Cleveland Clinic Florida, United States; Sitaramesh Emani, Ohio State University Medical Center, United States; Abhinav Sharma, Heart and Vascular Center—Center for Advanced Care—Froedtert Hospital, United States; David Fermin, Spectrum Health Medical Group (SHMG)—Cardiology (West Michigan Heart P.C)—Grand Rapids, United States; Melissa Lyle, Mayo Clinic, United States; David Raymer, UCHealth Heart and Vascular Center—Anschutz Medical Campus, United States; Andrew Darlington, Piedmont Fayette Hospital, United States; Martin S. Maron, Lahey Hospital & Medical Center, United States; Christopher Nielsen, Medical University of South Carolina, United States; Andrew Wang, Duke University, United States; Sherif Nagueh, Houston Methodist Hospital, United States; Matthew Martinez, Morristown Medical Center, United States; Milind Desai, Cleveland Clinic—Taussig Cancer Institute, United States of America; Albree Tower‐Rader, Massachusetts General Hospital (MGH)—Interventional Cardiology Associates, United States; Jacob Kelly, Alaska Heart and Vascular Institute, United States; Florian Rader, Cedars‐Sinai Medical Center, United States; Sounok Sen, Yale School of Medicine, United States; Patrick Bering, MedStar Washington Hospital Center, United States; Mathew Maurer, New York‐Presbyterian/Columbia University Medical Center, United States; Sumeet Mitter, Mount Sinai Hospital, United States; Mark Sherrid, NYU Langone Medical Center, United States; Timothy Wong, University of Pittsburg Medical Center, United States; Zainal Hussain, Ascension St. John Clinical Research Institute, United States; Sara Saberi, University of Michigan Health System, United States; Srihari Naidu, Westchester Medical Center, United States; Jorge Silva Enciso, University of California—San Diego, United States.

## Supporting information

Data S1
Tables S1–S3
Figures S1–S3
