# Supplementary material for: Dosing and Safety Profile of Aficamten in Symptomatic Obstructive Hypertrophic Cardiomyopathy: Results From SEQUOIA‐HCM
Source: J Am Heart Assoc. 2024 Jul 26;13(15):e035993. doi: 10.1161/JAHA.124.035993 (PMC11964075; doi:10.1161/JAHA.124.035993)
Supplement: Supplementary file 1 — Data S1 Tables S1–S3 Figures S1–S3 [file JAH3-13-e035993-s001.pdf]

Supplemental Material

Supplemental Methods

Data S1. Study protocol

Protocol for: Coats CJ, Masri A, Nassif ME, et al. Dosing and Safety Profile of Aficamten in Symptomatic Obstructive Hypertrophic Cardiomyopathy: Results From SEQUOIA-HCM. Circulation 2024;XXX:XXX. DOI:

This trial protocol has been provided by the authors to give readers additional information about the work.

Original protocol

PROTOCOL CY 6031

A PHASE 3, MULTI-CENTER, RANDOMIZED, DOUBLE-BLIND, PLACEBO-CONTROLLED TRIAL TO EVALUATE THE EFFICACY AND SAFETY OF CK-3773274 IN ADULTS WITH SYMPTOMATIC HYPERTROPHIC CARDIOMYOPATHY AND LEFT VENTRICULAR OUTFLOW TRACT OBSTRUCTION

**Protocol Version and Date:**	26 July 2021
**Previous Version(s):**	Not applicable
**Product:**	CK-3773274 (aficamten)
**Regulatory Authority Identifier Number(s):**	IND 138814<br>EudraCT Number 2021-003536-92
**Sponsor:**	Cytokinetics, Inc.<br>280 East Grand Avenue<br>South San Francisco, CA 94080, USA

This document contains confidential information, which should not be copied, referred to, released or published without documented approval from Cytokinetics, Inc.

INVESTIGATOR SIGNATURE PAGE

Protocol Number:	CY 6031
Protocol Title:	A Phase 3, Multi-Center, Randomized, Double-blind, Placebo-controlled Trial to Evaluate the Efficacy and Safety of CK-3773274 in Adults with Symptomatic Hypertrophic Cardiomyopathy and Left Ventricular Outflow Tract Obstruction
Protocol Version and Date:	26 July 2021

Principal Investigator Commitment

I, the undersigned Principal Investigator, submit this statement of commitment as evidence that I understand my responsibilities pursuant to the Code of Federal Regulations (21 CFR § 312) and International Council for Harmonisation (ICH) E6(R2) Good Clinical Practice (GCP) guidelines, as well as with any and all applicable federal, state and/or local laws and regulations, and agree to conduct this trial in accordance with the protocol referenced herein.

Investigator Name: ______________________________ Date: ____________________

Investigator Signature: ____

PROTOCOL APPROVAL PAGE

Protocol Number:	CY 6031
Protocol Title:	A Phase 3, Multi-Center, Randomized, Double-blind, Placebo-controlled Trial to Evaluate the Efficacy and Safety of CK-3773274 in Adults with Symptomatic Hypertrophic Cardiomyopathy and Left Ventricular Outflow Tract Obstruction
Protocol Version and Date:	26 July 2021
Sponsor:	Cytokinetics, Inc.<br>280 East Grand Avenue<br>South San Francisco, CA 94080

27 JULY 2021

Laura Robertson, MD
Medical Director, Clinical Research, Cardiovascular

Date:

TABLE OF CONTENTS

INVESTIGATOR SIGNATURE PAGE 2
PROTOCOL APPROVAL PAGE 3
TABLE OF CONTENTS 4
LIST OF TABLES 8
LIST OF FIGURES 8
1. PROTOCOL SUMMARY 9
1.1. Synopsis 9
1.2. Schema 16
1.3. Schedule of Activities 17
1.4. Key Contacts 20
2. INTRODUCTION 21
2.1. Trial Rationale 21
2.2. Background 21
2.2.1. Hypertrophic Cardiomyopathy 21
2.2.2. CK-3773274 22
2.3. Benefit/Risk Assessment 22
2.3.1. Risk Assessment 22
2.3.1.1. Mitigation Strategy 23
2.3.2. CK-3773274 Benefit Assessment 23
3. OBJECTIVES AND ENDPOINTS 25
4. TRIAL DESIGN 27
4.1. Overall Design 27
4.1.1. Number of Sites 27
4.1.2. Number of Patients 27
4.1.3. Replacement of Patients 27
4.1.4. Trial Duration 27
4.1.5. CMR Imaging Sub-Study 28
4.2. Scientific Rationale for Trial Design 28
4.3. Justification for Dose 28
4.4. End of Study Definition 28
5. STUDY POPULATION 29

5.1. Inclusion Criteria 29
5.2. Exclusion Criteria 30
5.3. Lifestyle Considerations 32
5.4. Screen Failures....32
6. INVESTIGATIONAL PRODUCT 33
6.1. Investigational Product(s) Administered 33
6.2. Preparation/Handling/Storage/Accountability....33
6.3. Measures to Minimize Bias: Randomization and Blinding....34
6.4. Investigational Product Compliance....34
6.5. Concomitant Therapy 35
6.5.1. Drug-Drug Interactions....35
6.5.2. Rescue Medicine....36
6.6. Dose Modifications....36
6.6.1. Scheduled Dose Titrations....36
6.6.1.1. Week 2 Visit 36
6.6.1.2. Week 4 Visit 36
6.6.1.3. Week 6 Visit 36
6.6.1.4. Week 8 Visit 37
6.6.2. Dose Reductions 37
6.6.3. LVEF Safety Threshold....37
6.6.4. Hepatotoxicity Stopping and Rechallenge Rules 38
6.7. Access to Investigational Product after the End of the Study 38
7. TEMPORARY INTERRUPTION OF INVESTIGATIONAL PRODUCT, DISCONTINUATION OF INVESTIGATIONAL PRODUCT, AND PATIENT CONSENT WITHDRAWAL 39
7.1. Temporary IP Interruption....39
7.2. Permanent Discontinuation of IP....39
7.2.1. Management of Patients after Permanent Discontinuation of IP....40
7.3. Discontinuation from Trial Procedures 40
7.4 Patient Consent Withdrawal 41
7.5. Lost to Follow up....41
8. TRIAL ASSESSMENTS AND PROCEDURES....42
8.1. Visit Windows 42

8.2. Visit Schedule 42
8.2.1. Screening Visit....43
8.2.2. Day 1....43
8.2.3. Weeks 2 through 20 43
8.2.4. Week 24: End of Treatment Visit....43
8.2.5. Week 28: End of Study Visit....44
8.2.6. Early Discontinuation Visit 44
8.2.7. Unscheduled Visit....44
8.3. Efficacy Assessments 44
8.3.1. Cardiopulmonary Exercise Testing 44
8.3.2. Echocardiography....45
8.3.3. Cardiac Magnetic Resonance 46
8.3.4. New York Heart Association Functional Classification....46
8.3.5. Clinical Global Impression Scale 47
8.3.6. Patient-Reported Outcomes....47
8.4. Safety Assessments....47
8.4.1. Physical Examinations....47
8.4.2. Height and Weight....47
8.4.3. Vital Signs 47
8.4.4. Electrocardiograms....48
8.4.5. Laboratory Assessments 48
8.5. Adverse Events and Serious Adverse Events 49
8.5.1. Adverse Events....49
8.5.1.1. Definition of Adverse Event....49
8.5.1.2. Definition of Serious Adverse Event....49
8.5.1.3. Intensity of Adverse Events....50
8.5.1.4. Relationship to Investigational Product....51
8.5.1.5. Relationship to Trial Procedures 51
8.5.1.6. Reporting of AEs 51
8.5.1.7. Reporting Procedures for SAEs....51
8.5.1.8. Follow-up of AEs and SAEs....52
8.5.1.9. Regulatory Reporting....52
8.5.1.10. Pregnancy and Breastfeeding 53

8.6. Treatment of Overdose 54
8.7. Pharmacokinetics 54
8.8. Genetics 55
8.9. Serum for Biomarker Analysis 55
8.10. Serum Collection for Future Analyses 55
8.11. Immunogenicity Assessments 55
9. STATISTICAL CONSIDERATIONS 56
9.1. Statistical Hypotheses 56
9.2. Sample Size Determination 56
9.3. Populations for Analyses 56
9.4. Statistical Analyses 56
9.4.1. General Considerations 57
9.4.1.1. Multiplicity Adjustment 57
9.4.2. Primary Endpoint(s) 57
9.4.3. Secondary Endpoint(s) 58
9.4.4. Exploratory Endpoint(s) 59
9.4.5. Safety Analysis 60
9.4.5.1. Adverse Events 60
9.4.5.2. Serious Adverse Events 60
9.4.6. Pharmacokinetic Endpoints 60
9.4.7. Patient Disposition 61
9.4.8. Demographics and Other Baseline Characteristics 61
9.4.9. Investigational Product Exposure 61
9.4.10. Concomitant Medications 61
9.4.11. Clinical Laboratory Parameters 61
9.4.12. Vital Signs 61
9.4.13. Electrocardiogram 61
9.5. Data Monitoring Committee 61
10. SUPPORTING DOCUMENTATION AND OPERATIONAL CONSIDERATIONS 62
10.1. Appendix 1: Regulatory, Ethical, and Trial Oversight Considerations 62
10.1.1. Regulatory and Ethical Considerations 62
10.1.2. Financial Disclosure 62

10.1.3. Informed Consent Process 62
10.1.4. Data Protection 63
10.1.5. Committees Structure 63
10.1.6. Data Quality Assurance 64
10.1.7. Source Documents 64
10.1.8. Trial and Site Start and Closure 64
10.2. Appendix 2: Clinical Laboratory Tests 66
10.3. Appendix 3: Contraceptive Guidance 67
10.4. Appendix 4: Genetics 69
10.5. Appendix 5: Liver Safety: Actions and Follow-up Assessments 70
10.6. Appendix 6: Abbreviations 74
11. REFERENCES 77

LIST OF TABLES

Table 1: Trial Objectives and Endpoints 25
Table 2: Investigational Products 33
Table 3: Echocardiogram Criteria for Scheduled Dose Titrations 37
Table 4: CY 6031 Visit Windows 42
Table 5: CY 6031 Echocardiographic LV Parameters to be Measured 46
Table 6: Summary of PK Time Points 55
Table 7: Analysis Sets 56
Table 8: Protocol-Required Safety Laboratory Assessments 66
Table 9: List of Abbreviations 74

LIST OF FIGURES

Figure 1: Statistical Testing Hierarchy for Trial Endpoints 15
Figure 2: Trial Schema 16

1. PROTOCOL SUMMARY

1.1. Synopsis

**Name of Investigational Product(s) (IP):** CK-3773274
**Name of Active Ingredient(s):** CK-3773274
**Protocol Title:**<br>A Phase 3, Multi-Center, Randomized, Double-blind, Placebo-controlled Trial to Evaluate the Efficacy and Safety of CK-3773274 in Adults with Symptomatic Hypertrophic Cardiomyopathy and Left Ventricular Outflow Tract Obstruction
**Phase of Development:** Phase 3
**Rationale:**<br>Hypertrophic cardiomyopathy (HCM) is a disease of the cardiac sarcomere for which the fundamental pathophysiologic abnormality is myocardial hypercontractility leading to cardiac hypertrophy. In patients with obstructive HCM (oHCM), dynamic left ventricular outflow tract (LVOT) obstruction creates a high-pressure outflow tract gradient during systole. Patients with oHCM often develop signs and symptoms of heart failure.<br>CK-3773274 is a small molecule cardiac myosin inhibitor being developed as a chronic, oral treatment for patients with HCM. CK-3773274 is designed to reduce the hypercontractility that underlies the pathophysiology of HCM. Selective inhibition of cardiac myosin with CK-3773274 may yield potential advantages over current therapies for oHCM by directly reducing myocardial hypercontractility and addressing the fundamental cause of this sarcomeric disease.<br>In the Phase 2 trial, CY 6021 (REDWOOD-HCM), patients with oHCM received up to three doses of CK-3773274 or placebo (randomized 2:1) in a dose escalating manner using echocardiography to guide dose titration. Two cohorts of approximately 20 patients each were enrolled and treated for 10 weeks. Doses in the first cohort were 5, 10, 15 mg once daily; the second cohort studied 10, 20, and 30 mg once daily. In both cohorts, CK-3773274 significantly and substantially reduced the LVOT gradient (LVOT-G) in a dose and exposure dependent manner. There were no treatment interruptions or discontinuations, nor any treatment related serious adverse events. The results from this trial support progression of CK-3773274 to Phase 3 given the association between reductions in LVOT-G and improvements in patient symptoms and function.<br>This trial will evaluate the effects of treatment with CK-3773274 over a 24-week period on cardiopulmonary exercise capacity and health status in patients with symptomatic oHCM.
**Objectives and Endpoints:**

Objectives	***Endpoint(s)***
Primary	
To evaluate the effect of CK-3773274 on exercise capacity in patients with symptomatic oHCM	• Change in peak oxygen uptake (pVO_2) by cardiopulmonary exercise testing (CPET) from baseline to Week 24

Secondary	
To evaluate the effect of CK-3773274 on patient health status	• Change in Kansas City Cardiomyopathy Questionnaire – Clinical Summary Score (KCCQ-CSS) from baseline to Week 12 and Week 24
To evaluate the effect of CK-3773274 on New York Heart Association (NYHA) Functional Classification	• Proportion of patients with ≥1 class improvement in NYHA Functional Class from baseline to Week 12 and Week 24
To evaluate the effect of CK-3773274 on post-Valsalva left ventricular outflow tract gradients (LVOT-G)	• Change in post-Valsalva LVOT-G from baseline to Week 12 and Week 24<br>• Proportion of patients with post-Valsalva LVOT-G <30 mmHg at Week 12 and Week 24
To evaluate the effect of CK-3773274 on exercise capacity	• Change in total workload during CPET from baseline to Week 24
Safety	
To evaluate the safety and tolerability profile of CK-3773274 in patients with symptomatic oHCM	• Incidence of reported major adverse cardiac events (cardiovascular [CV] death, cardiac arrest, non-fatal stroke, non-fatal myocardial infarction, CV hospitalization)<br>• Incidence of new onset persistent atrial fibrillation<br>• Incidence of appropriate implantable cardiac defibrillator (ICD) discharges and aborted sudden cardiac death<br>• Incidence of left ventricular ejection fraction (LVEF) <50%<br>• Incidence of treatment emergent adverse events
Exploratory	
To evaluate the effect of CK-3773274 on exercise capacity and functional class	• Compared with baseline, number of patients at Week 24 achieving either:<br>– Change from baseline of ≥1.5 mL/kg/min in pVO_2 AND ≥1 class improvement in NYHA Functional Class<br>**OR**<br>– Change from baseline of ≥3.0 mL/kg/min in pVO_2 AND no worsening of NYHA Functional Class
To evaluate the effect of CK-3773274 on patient response over time	• Proportion of patients with improvement in KCCQ-CSS >5 points at Weeks 12 and 24<br>• Proportion of patients with resting LVOT-G <30 mmHg, post-Valsalva LVOT-G <50 mmHg, and NYHA Functional Class I at Weeks 12 and 24<br>• Proportion of patients with resting LVOT-G <30 mmHg, post-Valsalva LVOT-G <50 mmHg, and ≥ 1 class improvement in NYHA Functional Class at Weeks 12 and 24

To evaluate the effect of CK-3773274 on other CPET parameters	Change from baseline to Week 24 in:<br>• Ventilatory efficiency (VE/VCO_2 slope)<br>• Circulatory power (VO_2 × systolic BP)<br>• Ventilatory anaerobic threshold (VAT)
To evaluate the effect of CK-3773274 on health status and health-related quality of life as measured by PRO questionnaire	• Change from baseline to Week 24 in individual responses to the EuroQol 5-dimension 5-level instrument (EQ-5D-5L)
To evaluate the effect of CK-3773274 on cardiac function and structure	• Change from baseline to Week 24 in echocardiographic measurements of cardiac structure and of systolic function including:<br>– LVEF<br>– Left ventricular end-systolic and end-diastolic volumes (LVESV and LVEDV, respectively)<br>– Left atrial volume
To evaluate the effect of CK-3773274 on biomarker levels	• Change from baseline values in NT-pro-BNP, hs-cardiac-TnI and other biomarkers through Week 24
To evaluate the effect of CK-3773274 on left ventricular mass, function, and structure by cardiac magnetic resonance (CMR) imaging	• Change from baseline to Week 24 in CMR measurements of:<br>– Left ventricular (LV) mass index<br>– LVEF<br>– Septal and free wall thickness<br>– Left atrial volume index<br>– LVESV<br>– LVEDV
To assess the pharmacokinetics of CK-3773274 and its metabolites	• Pharmacokinetic parameters through Week 24

**Overall Design:**<br>This is a Phase 3 randomized, placebo-controlled, double-blind, multi-center trial in patients with symptomatic oHCM. Approximately 270 eligible patients will be randomized in a 1:1 ratio to receive CK-3773274 or placebo. Doses of 5, 10, 15, or 20 mg or matching placebo will be administered in an escalating manner using echocardiography to guide dose titration. Randomization will be stratified by use of beta-blockers and CPET exercise modality.<br>The trial will comprise three periods. The screening period will be up to 6 weeks in duration. The double-blind placebo-controlled treatment period will last 24 weeks. Following the final dose of investigational product (IP), there will be a 4-week safety follow-up period. IP will be administered orally once daily. During the initial six weeks of the treatment period, IP doses will be individually titrated at Weeks 2, 4, and 6 using echocardiography. Dose escalation at the Weeks 2, 4, and 6 visits will occur only if a patient has a post-Valsalva LVOT-G ≥30 mmHg and a biplane LVEF ≥55%. An echocardiogram will be performed at each subsequent visit during the trial and the dose down-titrated if necessary. The primary endpoint of pVO_2 will be measured by CPET at screening and at end of treatment (Week 24). If applicable, patients will continue taking background HCM medications consistent with regional clinical practice guidelines during the trial.<br>A CMR imaging sub-study will be open to approximately 40 patients who consent to participate.
**Trial Center(s):**<br>This trial will take place at approximately 80 sites worldwide.
**Number of Patients:**<br>Approximately 270 patients will be randomized to CK-3773274 or placebo.

Key Eligibility Criteria:

The key eligibility criteria are below. A full listing of eligibility criteria can be found in Section 5.

Inclusion Criteria

- Males and females between 18 and 85 years of age, inclusive, at screening.
- Body mass index <35 kg/m^2.
- Diagnosed with oHCM per the following criteria:
  - Has LV hypertrophy and non-dilated LV chamber in the absence of other cardiac disease and
  - Has an end-diastolic LV wall thickness as measured by the echocardiography core laboratory of ≥15 mm in one or more myocardial segments
- Has resting LVOT-G ≥30 mmHg and post-Valsalva LVOT G ≥50 mmHg during screening as determined by the echocardiography core laboratory.
- LVEF ≥60% at screening as determined by the echocardiography core laboratory.
- NYHA Functional Class II or III at screening.
- Hemoglobin ≥10g/dL at screening.
- Respiratory exchange ratio (RER) ≥1.05 and pVO2 <80% predicted on the screening CPET per the core laboratory.
- Patients on beta-blockers, verapamil, or diltiazem should have been on stable doses for >6 weeks prior to randomization and anticipate remaining on the same medication regimen during the trial.

Exclusion Criteria

Any of the following criteria will exclude potential patients from the trial:

- Known or suspected infiltrative, genetic or storage disorder causing cardiac hypertrophy that mimics oHCM (eg, Noonan syndrome, Fabry disease, amyloidosis).
- Significant valvular heart disease (per investigator judgment).
  - Moderate-severe valvular aortic stenosis.
  - Moderate-severe mitral regurgitation not due to systolic anterior motion of the mitral valve.
- History of LV systolic dysfunction (LVEF <45%) or stress cardiomyopathy at any time during their clinical course.
- Inability to exercise on a treadmill or bicycle (eg, orthopedic limitations).
- Has been treated with septal reduction therapy (surgical myectomy or percutaneous alcohol septal ablation) or has plans for either treatment during the trial period.
- Documented paroxysmal atrial fibrillation during the screening period.
- Paroxysmal or permanent atrial fibrillation requiring rhythm restoring treatment (eg, direct-current cardioversion, atrial fibrillation ablation procedure, or antiarrhythmic therapy) ≤6 months prior to screening. (This exclusion does not apply if atrial fibrillation has been treated with anticoagulation and adequately rate-controlled for >6 months.)
- History of syncope or sustained ventricular tachyarrhythmia with exercise within 6 months prior to screening.
- Has received prior treatment with CK-3773274 or mavacamten.

Exclusion Criteria for CMR sub-study

- Inability to tolerate CMR.
- Has an ICD.
- Has a cardiac pacemaker.

Data Monitoring Committee:

An independent Data Monitoring Committee (DMC) will be established for this trial to formally review the accumulating data periodically in order to assess risk to patients during the conduct of the trial. Details regarding the scope of responsibilities, meetings and communication procedures, as well as information requirements will be outlined in the DMC Charter. The DMC will have access to actual treatment assignments and patient-level data from the clinical trial database.

Statistical Methods:

Sample Size Calculation: assuming a difference in change from baseline in pVO_2 of 1.5 mL/kg/min for CK-3773274 compared to placebo, a standard deviation (SD) of 3.5 mL/kg/min, and 10% of patients missing change from baseline data of the primary endpoint, a sample size of 270 patients (approximately 135 randomized to CK-3773274 and 135 randomized to placebo) provides more than 90% power to detect the difference in pVO_2 change from baseline to Week 24 with a 2-sided type I error of 0.05.

Unless specified otherwise, efficacy analyses will be performed on the full analysis set (FAS), which includes all randomized patients who receive at least one dose of IP and have at least one post-baseline efficacy assessment. The primary analysis will test the null hypothesis that there is no treatment difference in the primary endpoint between patients randomized to placebo and those randomized to CK-3773274 in the FAS. Change from baseline in pVO_2 will be analyzed using an ANCOVA model with treatment group, randomization stratification factors, baseline pVO_2 and baseline weight as covariates.

For preservation of the overall type I error rate at two-sided 0.05 for the primary and secondary endpoints, the primary endpoint is tested first at two-sided 0.05. If the primary endpoint achieves statistical significance at two-sided $p \leq 0.05$, then a parallel gatekeeper method with two-sided 0.025 separately allocated to Week 12 and to Week 24 is applied for the first four secondary endpoints, with their testing being in the sequential order of KCCQ-CSS change from baseline, proportion of patients with ≥ 1 NYHA functional class improvement, post-Valsalva LVOT-G change from baseline and proportion of patients with post-Valsalva LVOT-G < 30 mm Hg. If all four of the first four secondary endpoints at Week 12 (or at Week 24) have two-sided $p \leq 0.025$, then there is recycling of the 0.025 for Week 12 (or Week 24) to Week 24 (or Week 12) so that 0.05 is applicable to the corresponding testing of the first four secondary endpoints. If $p \leq 0.025$ for the first four secondary endpoints at Week 12 (or at Week 24) and if $p \leq 0.05$ for the first four secondary endpoints at Week 24 (or at Week 12), then the fifth secondary endpoint of change from baseline to Week 24 in total workload is tested at two-sided $p \leq 0.05$. The multiple testing procedure is illustrated in Figure 1 below. The testing sequence of secondary endpoints of KCCQ-CSS, NYHA and post-Valsalva LVOT-G for Week 12 and Week 24, as well as that for change in total workload at Week 24, will be detailed in the Statistical Analysis Plan.

Figure 1: Statistical Testing Hierarchy for Trial Endpoints

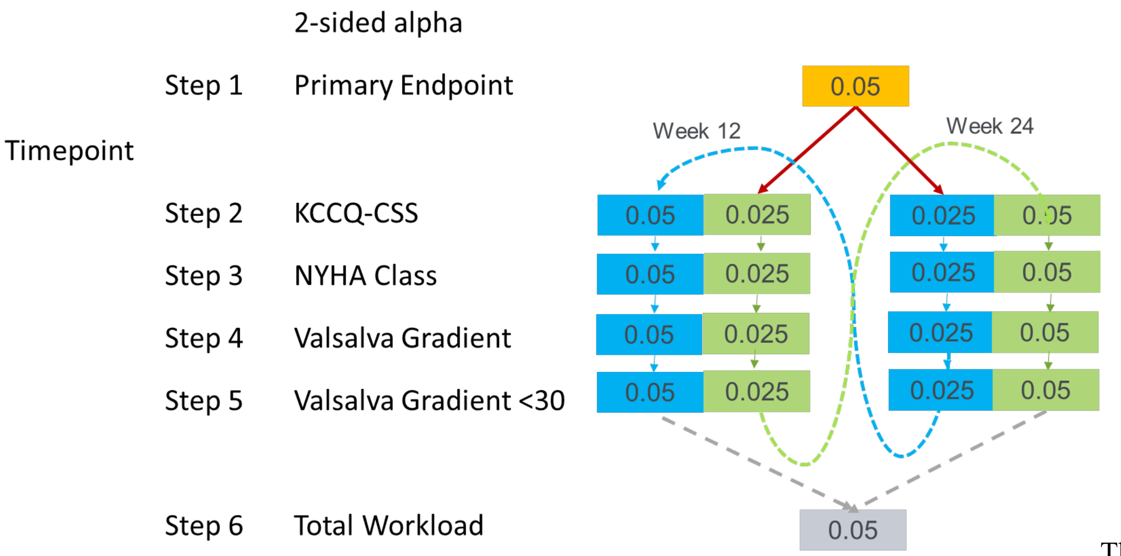

The proportion of responders in various exploratory endpoints will be analyzed using Cochran–Mantel–Haenszel (CMH) test stratified by randomization factors. The p-value and 95% confidence interval (CI) will be obtained using exact method. Other change from baseline endpoints will be analyzed using mixed measures repeated model with treatment, visit, randomization stratification factors, treatment by visit, baseline by visit interaction as fixed effect and baseline assessment as covariate.

Safety analyses will be performed on the safety analysis set (SAS) which includes all patients who received at least one dose of IP. The pharmacokinetics analysis set (PKS) will consist of patients who have at least one measurable plasma concentration of CK-3773274.

The number and percentage of patients reporting any treatment-emergent AEs will be coded using the MedDRA dictionary and be tabulated by system organ class and preferred term.

Analyses will be further detailed in the Statistical Analysis Plan.

1.2. Schema

Figure 2: Trial Schema

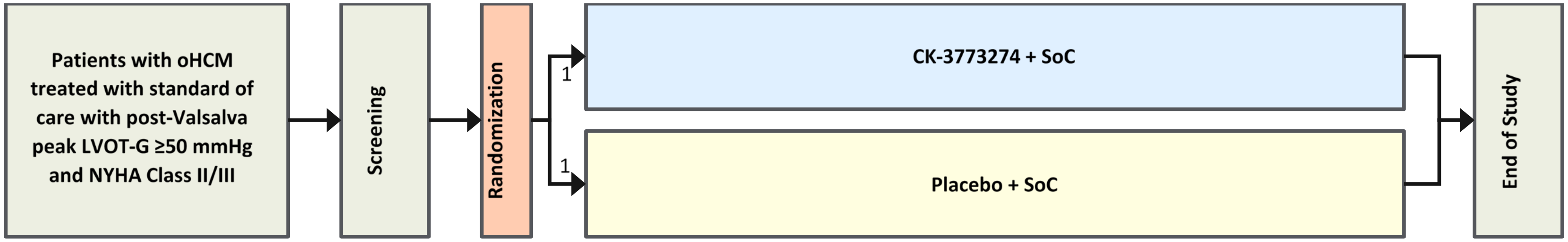

Study Visits

	Screen	D1	W2	W4	W6	W8	W12	W16	W20	W24	W28
Echocardiogram	↑	↑	↑*	↑*	↑*	↑*	↑	↑	↑	↑	↑
CPET	↑									↑	
KCCQ		↑	↑	↑	↑	↑	↑	↑	↑	↑	↑
NYHA	↑	↑	↑	↑	↑	↑	↑	↑	↑	↑	↑
Dose Titration			↑	↑	↑						

* Focused echocardiogram

1.3. Schedule of Activities

Trial Procedure	Screening[a] (≤ 42 days)	Day 1	Week 2	Week 4	Week 6	Week 8	Week 12	Week 16	Week 20	EOT[b] (Week 24)	EOS[c] (Week 28)	ED[d]
**GENERAL PROCEDURES AND SAFETY ASSESSMENTS**												
Informed consent	X											
Enrollment in IWRS	X											
Inclusion/exclusion criteria	X											
Medical/Surgical history	X	X										
Demographics	X											
Height/weight[e]	X									X		
Vital signs[f]	X	X	X	X	X	X	X	X	X	X	X	X
Physical examination	X									X		X
Adverse events/serious adverse events[g]	X	X	X	X	X	X	X	X	X	X	X	X
Concomitant medications	X	X	X	X	X	X	X	X	X	X	X	X
12-lead ECG (triplicate)	X	X	X	X	X	X	X	X	X	X	X	X
CPET	X									X		
CMR imaging sub-study[h]	X									X		
Focused Echo (LVOT-G + LVEF)[i]			X	X	X	X						
Full Echocardiogram[i]	X	X					X	X	X	X	X	
Randomization		X										
**CENTRAL LABORATORY ASSESSMENTS**												
Laboratory assessments	X	X					X			X	X	X
CMR substudy Hematocrit[j]	X									X		
Pregnancy test (WOCBP only)[k]	X	X		X		X	X	X	X	X	X	X
NT-pro-BNP		X	X	X	X	X	X	X	X	X	X	X
hs-cTnI		X	X	X	X	X	X	X	X	X	X	X
PK samples[l]		X	X	X	X	X	X	X	X	X		X

Trial Procedure	Screening[a] (≤ 42 days)	Day 1	Week 2	Week 4	Week 6	Week 8	Week 12	Week 16	Week 20	EOT[b] (Week 24)	EOS[c] (Week 28)	ED[d]
Other biomarker samples[m]		X					X			X		
Serum Collection for Future Analyses		X					X			X		
Genotype sample[n]		X										
**PATIENT-REPORTED OUTCOMES AND FUNCTIONING ASSESSMENTS**												
NYHA Functional Classification	X	X	X	X	X	X	X	X	X	X	X	X
KCCQ[o]		X	X	X	X	X	X	X	X	X	X	X
EQ-5D-5L[o]		X								X		X
CGI		X								X	X	X
PGI-C[o]		X								X	X	X
SAQ-7[o]		X		X		X	X	X	X	X	X	X
**INVESTIGATIONAL PRODUCT**												
IP dose administration at site[p]		X	X	X	X	X	X	X	X	X		
IP dispensation		X	X	X	X	X	X	X	X			
IP dose titration[q]			X	X	X							
IP dose adjustment[r]						X	X	X	X			

CGI = Clinical Global Impression scale; CMR = cardiac magnetic resonance imaging; CPET = cardiopulmonary exercise testing; ECG = electrocardiogram; Echo = echocardiogram; ED = early discontinuation; EOS = end of study; EOT = end of treatment; EQ-5D-5L = EuroQol 5-dimension 5-level instrument; hs-cTnI = high sensitivity cardiac troponin I; IP = investigational product; IWRS = interactive web response system; KCCQ = Kansas City Cardiomyopathy Questionnaire; NT-proBNP = n-terminal prohormone brain natriuretic peptide; NYHA = New York Heart Association; SAQ-7 = Seattle Angina Questionnaire-7, PGI-C = Patient Global Impression of Change scale; PK = pharmacokinetic; WOCBP = women of childbearing potential

[a] The CPET must be completed within three weeks but not less than one week prior to randomization.

[b] If a patient is temporarily unable to exercise on the treadmill or bicycle (whichever modality was used at baseline) due to an adverse event (eg, ankle sprain, upper respiratory infection, migraine), but not due to HCM symptoms, or if the site is unable to perform CPET (eg, equipment malfunction), then the Week 24 visit may be postponed by up to 4 weeks. The patient should continue to receive IP until the visit. Sites should contact patients shortly before the Week 24 visit and confirm their ability to perform CPET.

[c] The EOS visit will occur at Week 28 or 4 weeks after a postponed Week 24 visit. It is not required for patients who discontinue IP > 4 weeks prior to the Week 24 visit.

[d] Patients who withdraw from the trial IP should complete an early discontinuation visit as soon as possible if they no longer wish to be part of the study. A safety contact (eg, phone call) should occur 4 weeks following the last dose of IP to assess adverse events and vital status.

[e] Height is measured at the Screening visit only.

[f] Vital signs include heart rate, respiratory rate, and blood pressure. Oxygen Saturation only done at screening.

[g] Only adverse events considered related to trial procedures are collected during the screening period until initiation of IP (Day 1). Any medical occurrence not related to a trial procedure during this period should be collected as medical history.
[h] A CMR imaging sub-study will be open to approximately 40 patients who consent to participate. The baseline CMR imaging should be done prior to randomization.
[i] Echocardiograms will be done prior to dosing on Day 1 and 2 hours after dosing in the clinic at other time points.
[j] Must be done within 24 hours of CMR.
[k] Only for WOCBP. Serum pregnancy test at Screening visit. A urine pregnancy test may be performed locally at all other required timepoints.
[l] When PK collection and an echocardiogram are scheduled for the same time point, PK collection should be completed prior to the echocardiogram.
[m] Other biomarkers are referenced in Table 8.
[n] A genotype sample will be collected on Day 1 from patients who provide consent.
[o] All PROs, KCCQ, ED-5D-5L, SAQ-7, and PGI-C, should be done prior to other assessments.
[p] On clinic visit days after Day 1, patients should take IP after the blood draw. At the Week 24 visit, patients should take their final dose of IP before the CPET.
[q] See Section 6.6.
[r] See Section 7.1.

1.4. Key Contacts

**Sponsor's Trial Contact:**	Edward Robbie<br>Manager, Clinical Operations<br>Email: CY6031ClinicalOperations@cytokinetics.com<br>Phone: +1 (650) 624-3046<br>Fax: +1 (650) 624-3225
**Sponsor's Medical Monitor:**	Laura Robertson, MD<br>Medical Director, Clinical Research, Cardiovascular<br>Email: CY6031MedicalMonitor@cytokinetics.com<br>Mobile: +1 (415) 290-5630<br>Fax: +1 (650) 624-3225
**Serious Adverse Event Reporting:**	Cytokinetics Drug Safety<br>Email: CY6031DrugSafety@cytokinetics.com<br>Fax: +1 (650) 243-4199

2. INTRODUCTION

This is a Phase 3 trial of CK-3773274, a small molecule, allosteric inhibitor of cardiac myosin being developed as a chronic oral treatment for patients with hypertrophic cardiomyopathy (HCM).

2.1. Trial Rationale

The development of a targeted therapeutic drug that directly reduces myocardial contractility in the sarcomere may yield potential advantages over current therapies for obstructive hypertrophic cardiomyopathy (oHCM) because it potentially addresses the underlying pathophysiology of HCM. CK-3773274 is a cardiac myosin inhibitor with potential to reduce left ventricular outflow tract (LVOT) obstruction and improve symptoms in patients with hyperdynamic ventricular contractility in oHCM.

This trial is intended to establish the efficacy and safety of CK-3773274 with respect to improvements in exercise capacity and patient symptoms, as well as reduction in left ventricular outflow tract gradient (LVOT-G) in patients with oHCM.

2.2. Background

2.2.1. Hypertrophic Cardiomyopathy

HCM results from pathogenic genetic mutations, often affecting the genes encoding the proteins of the cardiac sarcomere, such as myosin (Maron, B. J. 2018). Histologic features include myofibrillar disarray, myocyte hypertrophy and interstitial fibrosis. Clinically, HCM is characterized by left ventricular (LV) hypertrophy unexplained by loading conditions and a nondilated LV with preserved or increased ejection fraction (Gersh 2011). Imaging studies of patients with HCM show hypertrophied LV walls, enhanced ventricular contractility, normal end-diastolic LV volume, reduced end-systolic volume, impaired diastolic compliance and often left atrial enlargement (Marian 2017). From population-based insurance claims and national health system data, the prevalence of clinically identified individuals with HCM in the US and EU is approximately 1:2000 and 1:3195 (Maron, M. S. 2016; Husser 2018; Magnusson 2017; Pujades-Rodriguez 2018).

Approximately 70% of patients with phenotypic HCM will demonstrate an element of LVOT obstruction (Maron, M. S. 2006). The mechanisms for developing obstruction are well defined and involve a complex interplay between alterations in ventricular flow between asymmetric septal hypertrophy and the mitral valve leaflets. The result is abnormal systolic contact with the mitral valve leaflets (most commonly the anterior leaflet) and the development of an LVOT gradient (LVOT-G). By nature, oHCM is a dynamic condition with variable systolic gradients. In the setting of reduced afterload or reduced preload, symptoms change depending on the gradient and often worsen during exertion. Additional clinical manifestations of HCM include an elevated risk for ventricular fibrillation and sudden cardiac death; heart failure syndrome due to diastolic dysfunction; chest pain due to microvascular ischemia; palpitations and stroke due to atrial fibrillation; syncope and presyncope due to either ventricular arrhythmias or an abnormal blood pressure response to exercise; and, in a minority of patients, progression to systolic heart failure.

Contemporary management strategies for oHCM have resulted in the majority of patients achieving normal or near-normal longevity and improved morbidity; however, there has been little progress with the development of novel pharmacotherapies. Current medical treatment consists of beta-blockers, verapamil, diltiazem and disopyramide as recommended in the 2014 European Society of Cardiology and in the 2020 American College of Cardiology Foundation / American Heart Association guidelines for the diagnosis and management of HCM. For patients with advanced symptomatic disease unresponsive to medications, septal reduction therapies (surgical myectomy or percutaneous alcohol ablation of the septum) can provide effective LVOT-G reduction (Elliott 2014; Gersh 2011; Ponikowski 2016; Ommen 2020). A subgroup of patients, who have been resuscitated from sudden cardiac death or who are at risk of sudden cardiac death, may undergo placement of an implantable cardioverter defibrillator (ICD) (Kristensen 2014). For those patients with HCM with end-stage disease who have both significant systolic impairment and diastolic dysfunction, cardiac transplantation may be the only treatment option (Gersh 2011). Disease-related mortality is most often attributable to sudden cardiac death, heart failure, and embolic stroke.

Mutations in over a dozen genes encoding sarcomere-associated proteins cause HCM. MYH7 and MYBPC3, encoding β-myosin heavy chain and myosin-binding protein C, respectively, are the two most common genes involved, together accounting for approximately 50% of the HCM families (Elliott 2014). Mechanistically, mutations in HCM appear to increase the net power generation in the sarcomere in vitro (Chuan 2012; Sommese 2013; Spudich 2016; Toepfer 2019). The findings in these studies are consistent with the underlying myocardial pathophysiology of the LV in patients with HCM being hypercontractile with diminished compliance (Wilson 1967).

These nonclinical investigations have enhanced our understanding of the molecular pathogenesis of HCM and have stimulated efforts designed to identify cardiac myosin modulators that can target the underlying mechanism of hypercontractility in oHCM.

2.2.2. CK-3773274

CK-3773274, a small molecule allosteric inhibitor of cardiac myosin, is being developed as a chronic oral treatment for patients with HCM. CK-3773274 is designed to reduce the hypercontractility that underlies the pathophysiology of HCM in the cardiac sarcomere. The intended pharmacologic effect is reduction in force produced by the cardiac sarcomere resulting in less LVOT obstruction and improved diastolic function in patients with oHCM.

CK-3773274 has been studied in a Phase 1 study of healthy adult participants and a Phase 2 study of patients with oHCM. This Phase 3 trial will assess the efficacy and safety of CK-3773274 in patients with oHCM.

Please refer to the Investigator's Brochure for detailed information on the nonclinical and clinical studies of CK-3773274.

2.3. Benefit/Risk Assessment

2.3.1. Risk Assessment

Excessive exposures to CK-3773274 may result in an excess of the intended pharmacodynamic (PD) effect, namely a decrease in LV systolic function, resulting in decreases in stroke volume and cardiac output with compensatory increases in heart rate. In nonclinical toxicology studies,

sustained depression of cardiac function led to increases in heart weight and dilatation of the cardiac chambers. These adverse cardiac effects are consistent with the anticipated physiological response to an excessive PD effect of CK-3773274.

In the first-in-human CY 6011 study, short-term decreases in cardiac function produced no changes in the vital signs or electrocardiograms (ECGs) of the participants. The effect of CK-3773274 on left ventricular ejection fraction (LVEF) reversed within 24-48 hours (single ascending dose and multiple ascending dose cohorts) of discontinuation of dosing. The participants who had decreases in LVEF to <50% remained asymptomatic until their cardiac function returned to the normal LVEF range.

In the Phase 2 trial, CY 6021 (REDWOOD-HCM), one patient with an LVEF at baseline of 58% experienced a transient reduction in LVEF to the range of 40-50% after titrating up from 10 mg to 20 mg, requiring down titration with return of LVEF to above 50% after two weeks. No interruptions or discontinuations of treatment with CK-3773274 occurred in any patients in the study and, on average, LVEF returned to baseline within two weeks after the end of treatment.

Together these findings indicate that the treatment effect of CK-3773274 is well tolerated and readily reversible with either a reduction of dose or discontinuation of treatment.

2.3.1.1. Mitigation Strategy

The main mitigation strategy will be facilitated by an individualized dose titration scheme based on each patient’s PD response to CK-3773274 with application of prespecified echocardiographic criteria, including LVEF thresholds for dose escalation, down-titration, and drug discontinuation.

Patients enrolled in this trial will be required to have an LVEF ≥60% prior to randomization, as confirmed by the central echocardiography laboratory. A low starting dose of 5 mg and a maximum dose of 20 mg were chosen as these were found to be well-tolerated in the Phase 2 study (CY 6021) of patients with oHCM and effective at reducing the LVOT-G without adversely impacting overall LVEF. Dose escalation will be performed on an individualized basis only if the following criteria are met: both post-Valsalva LVOT-G ≥30 mmHg and biplane LVEF ≥55%. Importantly, in contrast to CY 6021, the lower limit of LVEF for dose escalations will be increased from 50% to 55% to provide a safety margin from the threshold of LVEF (<50%) that will trigger dose reduction. If the LVEF is <50% at any time, the dose of CK-3773274 will be down-titrated, and if the LVEF is <40% at any time, CK-3773274 will be temporarily interrupted.

An independent Data Monitoring Committee (DMC) will be established for this trial to formally review the accumulating data periodically in order to assess the risk to patients during the conduct of the trial. DMC members will include echocardiologists and HCM experts and will have access to treatment assignments and patient-level data in support of safety oversight.

2.3.2. CK-3773274 Benefit Assessment

The development of a targeted therapeutic drug that directly reduces myocardial contractility in the sarcomere may yield potential clinical benefit for patients with oHCM by trying to address the underlying pathophysiology of HCM. CK-3773274 is a cardiac myosin inhibitor with the

potential to reduce LVOT obstruction and thereby reduce symptoms in patients with hyperdynamic ventricular contractility in oHCM.

In the Phase 2 trial, CY 6021 (REDWOOD-HCM), patients with oHCM received up to three doses of CK-3773274 or placebo (randomized 2:1) in a dose escalating manner using echocardiography to guide dose titration. Two cohorts of approximately 20 patients each were enrolled and treated for 10 weeks. Doses in the first cohort were 5, 10, 15 mg once daily; the second cohort studied 10, 20, and 30 mg once daily. In both cohorts, CK-3773274 significantly and substantially reduced the LVOT gradient (LVOT-G) in a dose and exposure dependent manner. The majority of patients treated with CK-3773274 (78.6% in Cohort 1 and 92.9% in Cohort 2) achieved the target goal of treatment, defined as resting gradient <30 mmHg and post-Valsalva gradient <50 mmHg at Week 10 compared to placebo (7.7%).

Given that the LVOT-G is the primary cause of symptoms in patients with oHCM, participation in this trial may afford those randomized to CK-3773274 symptom reduction and increased exercise capacity. Patient contributions to the performance of this trial may yield a new therapeutic modality for the treatment of their disease.

3. OBJECTIVES AND ENDPOINTS

Table 1: Trial Objectives and Endpoints

**Objectives**	**Endpoint(s)**
Primary	
To evaluate the effect of CK-3773274 on exercise capacity in patients with symptomatic oHCM	• Change in peak oxygen uptake (pVO_2) by cardiopulmonary exercise testing (CPET) from baseline to Week 24
Secondary	
To evaluate the effect of CK-3773274 on patient health status	• Change in Kansas City Cardiomyopathy Questionnaire – Clinical Summary Score (KCCQ-CSS) from baseline to Week 12 and Week 24
To evaluate the effect of CK-3773274 on New York Heart Association (NYHA) Functional Classification	• Proportion of patients with ≥1 class improvement in NYHA Functional Class from baseline to Week 12 and Week 24
To evaluate the effect of CK-3773274 on post-Valsalva left ventricular outflow tract gradients (LVOT-G)	• Change in post-Valsalva LVOT-G from baseline to Week 12 and Week 24<br>• Proportion of patients with post-Valsalva LVOT-G <30 mmHg
To evaluate the effect of CK-3773274 on exercise capacity	• Change in total workload during CPET from baseline to Week 24
Safety	
To evaluate the safety and tolerability profile of CK-3773274 in patients with symptomatic oHCM	• Incidence of reported major adverse cardiac events (cardiovascular [CV] death, cardiac arrest, non-fatal stroke, non-fatal myocardial infarction, CV hospitalization)<br>• Incidence of new onset persistent atrial fibrillation<br>• Incidence of appropriate implantable cardiac defibrillator (ICD) discharges and aborted sudden cardiac death<br>• Incidence of left ventricular ejection fraction (LVEF) <50%<br>• Incidence of treatment emergent adverse events
Exploratory	
To evaluate the effect of CK-3773274 on exercise capacity and functional class	• Compared with baseline, number of patients at Week 24 achieving either:<br>– Change from baseline of ≥1.5 mL/kg/min in pVO_2 AND ≥1 class improvement in NYHA Functional Class<br>**OR**<br>– Change from baseline of ≥3.0 mL/kg/min in pVO_2 AND no worsening of NYHA Functional Class

Table 1: Trial Objectives and Endpoints (Continued)

Objectives	Endpoint(s)
To evaluate the effect of CK-3773274 on patient response over time	• Proportion of patients with improvement in KCCQ-CSS >5 points at Weeks 12 and 24<br>• Proportion of patients with resting LVOT-G <30 mmHg, post-Valsalva LVOT-G <50 mmHg, and NYHA Functional Class I at Weeks 12 and 24<br>• Proportion of patients with resting LVOT-G <30 mmHg, post-Valsalva LVOT-G <50 mmHg, and ≥ 1 class improvement in NYHA Functional Class at Weeks 12 and 26
To evaluate the effect of CK-3773274 on other CPET parameters	Change from baseline to Week 24 in:<br>• Ventilatory efficiency (VE/VCO_2 slope)<br>• Circulatory power (VO_2 × systolic BP)<br>• Ventilatory anaerobic threshold (VAT)
To evaluate the effect of CK-3773274 on health status and health-related quality of life as measured by PRO questionnaire	• Change from baseline to Week 24 in individual responses to the EuroQol 5-dimension 5-level instrument (EQ-5D-5L)
To evaluate the effect of CK-3773274 on health status and quality of life related to chest pain-like angina	Change from baseline to Week 24 in summary and domain scores for the Seattle Angina Questionnaire-7 (SAQ-7)
To evaluate the effect of CK-3773274 on cardiac function and structure	• Change from baseline to Week 24 in echocardiographic measurements of cardiac structure and of systolic function including:<br>– LVEF<br>– Left ventricular end-systolic and end-diastolic volumes (LVESV and LVEDV, respectively)<br>– Left atrial volume
To evaluate the effect of CK-3773274 on biomarker levels	• Change from baseline values in NT-pro-BNP, hs-cardiac-TnI and other biomarkers through Week 24
To evaluate the effect of CK-3773274 on left ventricular mass, function, and structure by cardiac magnetic resonance (CMR) imaging	• Change from baseline to Week 24 in CMR measurements of:<br>– Left ventricular (LV) mass index<br>– LVEF<br>– Septal and free wall thickness<br>– Left atrial volume index<br>– LVESV<br>– LVEDV
To assess the pharmacokinetics of CK-3773274 and its metabolites	• Change from baseline in pharmacokinetic parameters through Week 24

4. TRIAL DESIGN

4.1. Overall Design

This is a Phase 3, randomized, placebo-controlled, double-blind, multi-center trial in patients with symptomatic oHCM. Approximately 270 eligible patients will be randomized in a 1:1 ratio to receive CK-3773274 or placebo. Randomization will be stratified by use of beta-blockers (yes or no) and CPET exercise modality (treadmill or bicycle) and implemented in the Interactive Web Response System (IWRS). A cap on the number of patients taking beta-blockers and will not exceed approximately 70% of total enrollment. The number of patients with persistent atrial fibrillation at screening will also be capped at approximately 15%, and the number of patients using the bicycle CPET exercise modality will be capped at approximately 50% as well.

IP will be administered orally once daily with or without food. During the initial six weeks of the treatment period, IP doses will be individually titrated at Weeks 2, 4, and 6 using echocardiography. Dose escalation at Weeks 2, 4, and 6 will occur only if a patient has a post-Valsalva LVOT-G ≥30 mmHg and a biplane LVEF ≥55%. Echocardiograms will be performed at each subsequent visit during the trial and the dose down titrated if necessary. The primary endpoint of pVO_2 will be measured by CPET at screening and at end of treatment (Week 24). If applicable, patients will continue taking background HCM medications consistent with regional clinical practice guidelines during the trial.

All patients will be followed according to the Schedule of Activities (SoA) from randomization through the date of their final visit irrespective of whether the patient is continuing to receive IP, unless the patient has discontinued prematurely from the trial or withdrawn consent. An early discontinuation visit will be performed for patients that discontinue prematurely from the trial.

The overall study design is described by a trial schema in Figure 2.

The trial endpoints and objectives are defined in Table 1.

4.1.1. Number of Sites

Approximately 80 investigative sites worldwide will participate in this trial.

4.1.2. Number of Patients

Approximately 270 patients will be randomized in the trial.

4.1.3. Replacement of Patients

Patients who are withdrawn or removed from treatment or the trial will not be replaced.

4.1.4. Trial Duration

The trial will comprise three periods. After signing the informed consent form, patients will complete assessments to determine trial eligibility during a screening period of up to 6 weeks in duration. The double-blind placebo-controlled treatment period will last 24 weeks. Following the final dose of IP, there will be a 4-week safety follow-up period.

4.1.5. CMR Imaging Sub-Study

A CMR imaging sub-study will be open to approximately 40 patients who consent to participate.

4.2. Scientific Rationale for Trial Design

This trial is designed to provide data supporting the clinical efficacy and safety of CK-3773274 in patients with symptomatic oHCM and an LVOT-G >50 mmHg post-Valsalva. Reduction of the LVOT-G is expected to correlate with improvement in the patients' symptoms, health status and exercise capacity.

Since patient characteristics vary substantially in this disease, individualized dose titration to a PD response (reduction of the post-Valsalva LVOT-G to <30 mmHg with preservation of LVEF ≥55%) is being employed to maximize efficacy and safety. The eligibility criteria are designed to enable enrollment of a patient population representative of the general population of patients with oHCM while ensuring the safety of the patients in this trial.

A placebo control and double-blinded approach are being employed in this trial to avoid bias in data collection, including the safety assessments and PD measures that comprise the primary and secondary endpoints.

4.3. Justification for Dose

The doses of CK-3773274 are summarized in Table 2. A starting dose of 5 mg and a maximum dose of 20 mg were chosen as these were found to be well-tolerated in the Phase 2 study of patients with oHCM and effective at reducing the LVOT-G without adversely impacting overall LVEF. Within-patient dose escalation will only occur when the patient's current dose is well-tolerated, and the patient meets the criteria described in Section 6.6.

4.4. End of Study Definition

The end of the study is defined as the date of the last visit of the last patient in the trial.

5. STUDY POPULATION

Before patients begin any trial-specific activities/procedures, Cytokinetics requires a copy of the site's institutional review board/independent ethics committee (IRB/IEC) approval of the protocol, informed consent form (ICF), and all other patient information and/or recruitment material, if applicable. A signed ICF must be obtained from each patient before commencement of any trial-specific activities/procedures.

A patient's participation in the trial begins after signing the informed consent. After confirming the patient has met all eligibility criteria, randomization should then occur before the first dose on Day 1 is administered. The site is to document the informed consent signature and randomization dates in the patient's medical record and in/on the case report form (CRF).

Prospective approval of protocol deviations to recruitment and enrollment criteria, also known as protocol waivers or exceptions, is not permitted.

5.1. Inclusion Criteria

Patients are eligible to be included in the trial only if all the following criteria apply:

101. Able to comprehend and willing to sign an ICF and willing to comply with all trial procedures and restrictions for the duration specified in the Schedule of Activities (SoA; Section 1.3).
102. Males and females between 18 and 85 years of age, inclusive, at screening.
103. Body mass index <35 kg/m^2.
104. Diagnosed with oHCM per the following criteria:
    a. Has LV hypertrophy and non-dilated LV chamber in the absence of other cardiac disease and
    b. Has an end-diastolic LV wall thickness as measured by the echocardiography core laboratory of ≥15 mm in one or more myocardial segments
105. Has resting LVOT-G ≥30 mmHg and post-Valsalva LVOT-G ≥50 mmHg during screening as determined by the echocardiography core laboratory
106. LVEF ≥60% at screening as determined by the echocardiography core laboratory.
107. New York Heart Association (NYHA) Functional Class II or III at screening
108. Hemoglobin ≥10 g/dL at screening.
109. Respiratory exchange ratio (RER) ≥1.05 and pVO_2 <80% predicted on the screening CPET per the core laboratory.
110. Patients on beta-blockers, verapamil, or diltiazem should have been on a stable regimen for >6 weeks prior to randomization and anticipate remaining on the same medication regimen during the trial.
111. Male patients are eligible to participate if they agree to the following during the trial and for at least 4 weeks after the last dose of IP:
    a. Refrain from donating sperm

Plus either:

b. Be abstinent from heterosexual intercourse as their preferred and usual lifestyle (abstinent on a long term and persistent basis) and agree to remain abstinent

OR

Must agree to use a male condom and, when his female partner is a woman of childbearing potential, have his female partner use a highly effective method of contraception (as described in Appendix 3 [Section 10.3])

112. A female patient is eligible to participate if she is not pregnant, breastfeeding or planning to donate eggs, and at least one of the following conditions applies:

a. Is not a woman of childbearing potential (WOCBP; as described in Appendix 3 [Section 10.3])

OR

Is a WOCBP and using a highly effective method of contraception (as described in Appendix 3 [Section 10.3]) and male partner agrees to use a condom, during the trial and for at least 4 weeks after the last dose of IP.

b. A WOCBP must have a negative pregnancy test (urine or serum as required by local regulations) at Day 1, prior to the first dose of study IP.

Note: The investigator is responsible for review of medical history, menstrual history, and recent sexual activity to decrease the risk for inclusion of a woman with an early undetected pregnancy.

Contraceptive use by men or WOCBPs should be consistent with the guidance in Appendix 3 (Section 10.3) and local regulations regarding the methods of contraception for those participating in clinical studies.

113. Able to complete all screening procedures.

5.2. Exclusion Criteria

Patients will be excluded from the trial if any of the following criteria apply:

201. Significant valvular heart disease (per investigator judgment).

a. Moderate-severe valvular aortic stenosis and/or regurgitation.
b. Moderate-severe mitral regurgitation not due to systolic anterior motion of the mitral valve

202. Documented history of current obstructive coronary artery disease (>70% stenosis in one or more epicardial coronary arteries) or documented history of myocardial infarction.

203. Known or suspected infiltrative, genetic or storage disorder causing cardiac hypertrophy that mimics oHCM (eg, Noonan syndrome, Fabry disease, amyloidosis).

204. Prior treatment with cardiotoxic agents such as doxorubicin or similar.

205. History of LV systolic dysfunction (LVEF <45%) or stress cardiomyopathy at any time during their clinical course.
206. Has any ECG abnormality considered by the investigator to pose a risk to patient safety (eg, second degree atrioventricular block type II).
207. Documented paroxysmal atrial fibrillation during the screening period.
208. Paroxysmal or permanent atrial fibrillation requiring rhythm restoring treatment (eg, direct-current cardioversion, atrial fibrillation ablation procedure, or antiarrhythmic therapy) ≤6 months prior to screening. (This exclusion does not apply if atrial fibrillation has been treated with anticoagulation and adequately rate-controlled for >6 months.)
209. History of syncope or sustained ventricular tachyarrhythmia with exercise within 6 months prior to screening.
210. ICD placement within 3 months prior to screening or planned ICD placement during the trial.
211. History of appropriate ICD discharge for life-threatening ventricular arrhythmia within 6 months prior to screening.
212. Has been treated with septal reduction therapy (surgical myectomy or percutaneous alcohol septal ablation) or has plans for either treatment during the trial period.
213. Inability to exercise on a treadmill or bicycle (eg, orthopedic limitations).
214. Documented room air oxygen saturation reading >90% at screening.
215. Hepatic impairment defined by a total bilirubin (TBL) ≥1.5 × the upper limit of normal (ULN), or alanine aminotransferase (ALT) or aspartate aminotransferase (AST) ≥3 × ULN at screening. Patients with documented Gilbert syndrome and TBL ≥1.5 × ULN due to unconjugated hyperbilirubinemia, without other hepatic impairment, are permitted.
216. Recipient of a major organ transplant (eg, heart, lung, liver, bone marrow, renal) or anticipated transplantation within 12 months from randomization.
217. History or evidence of any other clinically significant disorder, malignancy, active infection, other condition, or disease that, in the opinion of the investigator or the Medical Monitor, would pose a risk to patient safety or interfere with the trial evaluation, procedures, or completion.
218. Estimated glomerular filtration rate (eGFR) <30 mL/min/1.73 m^2 (by the modified Modification of Diet in Renal Disease equation) at screening.
219. Currently participating in another investigational device or drug trial or received an investigational device or drug <1 month (or 5 half-lives for drugs, whichever is longer) prior to screening. Other investigational procedures while participating in this trial are not permitted.
220. Has received prior treatment with CK-3773274 or mavacamten.
221. Any known hypersensitivity to excipients in study drug tablets

Criteria for CMR sub-study

222. Inability to tolerate CMR.
223. Has an ICD.
224. Has a cardiac pacemaker.

5.3. Lifestyle Considerations

Patients will abstain from strenuous exercise for 24 hours before each blood collection for clinical laboratory tests.

5.4. Screen Failures

Screen failures are defined as patients who consent to participate in the clinical trial but are not subsequently randomized to IP. A minimal set of screen failure information is required to ensure transparent reporting of screen failure patients to meet the Consolidated Standards of Reporting Trials publishing requirements and to respond to queries from regulatory authorities. Minimal information includes demographics, reason for screen failure, eligibility criteria, and any SAEs related to trial-related procedures.

The screening eligibility period is 42 days as defined in Section 4.1. An individual who does not meet the criteria for participation in this trial is referred to as a screen failure. Patients may be rescreened one time after initial screening when the reason for screen failure is resolved or expected to be resolved.

Patients must re-sign an informed consent before they are rescreened. At rescreening, they must meet all inclusion/exclusion criteria at the time of rescreening and have all elements of the screening visit performed again to be eligible.

If an element of the screening visit could not be performed for logistical or technical reasons (eg, trained evaluator was out sick, equipment malfunction) the patient can return within the screening eligibility period to complete the visit.

Retesting for abnormal lab work within the original screening period may also be performed if there is reason to believe the repeat labs may improve and not be clinically significant and the patient is otherwise eligible to participate. Patients cannot be retested for abnormal bilirubin laboratory results. Patients who are retested during the original screening eligibility period do not need to re-sign an informed consent. The screening window is established by the date of the original screening visit and not by the date lab retesting takes place.

No waivers will be granted regarding inclusion/exclusion criteria.

6. INVESTIGATIONAL PRODUCT

This section describes any IP, marketed product(s), or placebo intended to be administered to a trial patient according to the study protocol.

6.1. Investigational Product(s) Administered

Table 2: Investigational Products

Arm Name	Active	Placebo
**IP/Product Name**	CK-3773274	Placebo
**Type**	Drug	Drug
**Dose Formulation**	Tablet	Tablet
**Unit Dose Strength(s)**	5mg	Matching placebo
**Dosage Level(s)**	5mg, 10mg, 15mg, 20mg	
**Route of Administration**	Oral	Oral
**Use**	Experimental	Placebo
**IMP and NIMP**	IMP	IMP
**Sourcing**	Patheon Inc.<br>Toronto Regional Operations (TRO)<br>2100 Syntex Court<br>Mississauga, Ontario L5N 7K9<br>Canada	Patheon Inc.<br>Toronto Regional Operations (TRO)<br>2100 Syntex Court<br>Mississauga, Ontario L5N 7K9<br>Canada
**Excipients**	Microcrystalline Cellulose<br>Mannitol<br>Croscarmellose Sodium<br>Hydroxypropyl Cellulose<br>Sodium Lauryl Sulfate<br>Magnesium Stearate<br>Opadry QX White	Microcrystalline Cellulose<br>Lactose Monohydrate<br>Croscarmellose Sodium<br>Magnesium Stearate<br>Opadry QX White
**Packaging and Labeling**	IP will be provided in blister packs which will be labeled as required per country requirement	IP will be provided in blister packs which will be labeled as required per country requirement

IMP = investigational medicinal product; NIMP = non-investigational medicinal product

6.2. Preparation/Handling/Storage/Accountability

The investigator or designee must confirm appropriate temperature conditions have been maintained during transit for all IP received and any discrepancies are reported and resolved before use of the IP.

Only patients randomized in the trial may receive IP and only authorized site staff may supply or administer IP. All IP must be stored in a secure, environmentally controlled, and monitored

(manual or automated) area in accordance with the labeled storage conditions with access limited to the investigator and authorized site staff.

The investigator, institution, or the head of the medical institution (where applicable) is responsible for IP accountability, chain of custody, reconciliation, and record maintenance (ie, receipt, reconciliation, and final disposition records).

Further guidance and information regarding IP storage condition, dispensation, packaging, labeling, and accounting procedures are provided in the Pharmacy Manual.

IP should be stored at or below 25°C.

6.3. Measures to Minimize Bias: Randomization and Blinding

All eligible patients will be centrally assigned to randomized IP using the IWRS. Before the trial is initiated, the login information & directions for the IWRS will be provided to each site.

Because viewing echocardiogram results could potentially compromise the blinded investigator and blinded study coordinator, specified unblinded study staff will perform and read the echocardiograms.

An unblinded sonographer at the site will perform the echocardiograms. An unblinded cardiologist, who is not the investigator and is called the Echo Cardiologist, will read the echocardiograms, measure the LVOT-G and LVEF and enter the echocardiogram results in the IWRS for dose titrations and adjustments. Therefore, the investigator and site staff will remain blinded to the echocardiogram images and results.

IP will be dispensed at the trial visits summarized in the SoA (Section 1.3). Returned IP should not be re-dispensed to the patients.

Patients randomized to placebo will receive placebo throughout the study and will perform all protocol procedures in order to maintain the blind for the treatment group allocation and IP dose. Patients should continue to take IP through the morning of the Week 24 Visit.

The IWRS will be programmed with blind-breaking instructions and the unblinding procedure is documented in the study manual. In case of an emergency, the investigator has the sole responsibility for determining if unblinding of a patient's intervention assignment is warranted. Patient safety must always be the first consideration in making such a determination. If the investigator decides that unblinding is warranted, the investigator is encouraged to contact Cytokinetics prior to unblinding a patient's intervention assignment unless this could delay emergency treatment of the patient. If a patient's intervention assignment is unblinded, Cytokinetics must be notified within 24 hours after breaking the blind. The date and reason that the blind was broken must be recorded in the source documentation and CRF, as applicable.

6.4. Investigational Product Compliance

When patients are dosed at the site, the date and time of the dose administered in the clinic will be recorded in the source documents and recorded in the CRF.

When patients self-administer IP at home, compliance with IP will be assessed at each site visit. Compliance will be assessed by counting returned tablets during the site visits and documented

in the source documents and CRF. Deviation(s) from the prescribed dosage regimen should be recorded in the CRF.

For IP accountability, a record of the following should be documented in the CRF at every visit and reconciled with IP and compliance records:

- the date and kits dispensed
- date and tablets returned

IP dosing first and last dates, including dates for dosing interruptions will also be recorded in the CRF.

6.5. Concomitant Therapy

Any medication, including over-the-counter or prescription medicines, vitamins, and/or herbal supplements, that the patient is receiving at the time of enrollment or receives during the trial must be recorded along with:

- Reason for use
- Dates of administration including start and end dates
- Dosage information including dose and frequency

Patients on beta-blockers, verapamil, or diltiazem should have been on stable doses for >6 weeks prior to randomization and anticipate remaining on the same medication regimen during the trial.

The Medical Monitor should be contacted if there are any questions regarding concomitant or prior therapy.

Patients may continue to take prescription medications, which in the opinion of the investigator and the Medical Monitor, will not interfere with the trial.

While medications and doses should remain stable whenever appropriate during the trial, investigators may prescribe or adjust any concomitant medication or treatment deemed necessary to provide adequate supportive care.

6.5.1. Drug-Drug Interactions

In vitro studies showed that CK-3773274 was metabolized by CYP2D6 and CYP3A with potential contributions from CYPs 2C9 and 2C19. Since CYP2D6 was identified in vitro as a metabolizing enzyme of CK-3773274, the degree of dependence of its metabolism on CYP2D6 was explored in the first-in-human study, CY 6011, in participants with a poor metabolizing CYP2D6 genotype. Compared to participants with an extensive metabolizing CYP2D6 genotype, the pharmacokinetics of CK-3773274 in participants with a poor metabolizing genotype were not different, suggesting that CYP2D6-mediated interactions are unlikely. In the absence of clinical data, the use of strong CYP3A inhibitors or inducers should be used with caution and careful monitoring.

Caution may be needed when administering CK-3773274 with known substrates of P-gp and OAT3 until drug-drug interaction studies are completed. Contact the Medical Monitor to determine if a potentially meaningful drug-drug interaction may exist.

6.5.2. Rescue Medicine

The use of rescue medications in the event of a low cardiac output state (ie, dobutamine) is allowable at any time during the trial (Section 8.6). The date and time of rescue medication administration as well as the name and dosage regimen of the rescue medication must be recorded.

6.6. Dose Modifications

6.6.1. Scheduled Dose Titrations

Patients randomized to CK-3773274 may receive up to four escalating doses of IP over the initial 6 weeks of the trial as outlined below in Table 3. Patients receiving CK-3773274 start at a dose of 5 mg once daily (Dose 1) and may escalate through doses of 10, 15, and 20 mg once daily if they continue to meet the escalation criteria or will stop at their current dose when escalation criteria are not met.

6.6.1.1. Week 2 Visit

After randomization each patient will receive Dose 1 once daily for two weeks. At the Week 2 visit, the patient will have an echocardiogram 2 hours following administration of their dose of IP. Patients will up-titrate to Dose 2 if the following conditions are met on echocardiography:

- Post-Valsalva LVOT-G ≥30 mmHg, and the biplane LVEF ≥55%

Otherwise, the patient will remain on Dose 1.

If LVEF is <50% at Week 2, the IWRS will assign the patient to placebo.

6.6.1.2. Week 4 Visit

After two more weeks on the assigned dose, at the Week 4 visit each patient will have an echocardiogram 2 hours following administration of their dose of IP. Patients will up-titrate to the next higher dose if the following conditions are met on echocardiography:

- Post-Valsalva LVOT-G ≥30 mmHg, and the biplane LVEF ≥55%

Otherwise, the patient will remain on the same dose.

If LVEF is <50% at Week 4, the IWRS will assign the patient to the prior dose level or to placebo if the patient was on Dose 1.

6.6.1.3. Week 6 Visit

After 2 more weeks on the assigned dose, at the Week 6 visit each patient will have an echocardiogram 2 hours following administration of their dose of IP. Patients will up-titrate to the next higher dose if the following conditions are met on echocardiography:

- Post-Valsalva LVOT-G ≥30 mmHg, and the biplane LVEF ≥55%

Otherwise, the patient will remain on the same dose.

If LVEF is <50% at Week 6, the IWRS will assign the patient to the prior dose level or to placebo if the patient was on Dose 1.

Table 3: Echocardiogram Criteria for Scheduled Dose Titrations

Echocardiogram Criteria for Scheduled Dose Titrations in Weeks 2, 4, and 6

Biplane LVEF		Post-Valsalva LVOT-G	Action
<50%			Reduce Dose[a]
≥50% - 55%			No Dose Change
≥55%	and	<30 mmHg	No Dose Change
≥55%	and	≥30 mmHg	Increase Dose

[a] Once a patient's IP dose is down titrated, no further escalation is permitted. If LVEF <50% on 5 mg, the patient will receive placebo.

6.6.1.4. Week 8 Visit

After two additional weeks on the assigned dose, at the Week 8 visit each patient will have an echocardiogram 2 hours following administration of their dose of IP to ensure the LVEF is ≥50%.

If the LVEF is <50% at Week 8, the IWRS will assign the patient to the next lower dose or to placebo if the patient was on Dose 1.

6.6.2. Dose Reductions

After Week 6, no further dose escalations may occur. During the course of the study, for safety reasons, dose reductions may occur at scheduled or unscheduled visits. Dose reductions will be determined by the IWRS system based on echocardiography results. After Week 8, dose reductions will be based on echocardiogram results from the initial scheduled or unscheduled visits. If the LVEF is <50%, then the IWRS will assign the patient to the next lower dose or to placebo if the patient was on Dose 1. The IWRS will not further reduce the dose for at least seven days after the previous reduction.

6.6.3. LVEF Safety Threshold

If the unblinded echocardiologist observes that the LVEF has crossed the defined safety threshold of <40% or feels the patient requires urgent medical attention, the unblinded echocardiologist will enter the LVEF value in the IWRS and discuss the results with the blinded investigator or qualified designee. The Medical Monitor will be informed in these cases.

If a patient's LVEF is <40% at any time, the following steps should occur after consultation with the Medical Monitor:

- IP should be stopped and held for at least 7 days.
- Repeat echocardiograms should be performed per investigator judgment until a normal LVEF (≥55%) has been documented at which point the patient can be re-started on IP after being down-titrated.

- Document dose interruption in the eCRF and include the reason for interruption, the date of the last dose, and the restart date (Section 7.1).

6.6.4. Hepatotoxicity Stopping and Rechallenge Rules

Patients with abnormal hepatic laboratory values (ie, alkaline phosphatase [ALP], AST, ALT, TBL) and/or international normalized ratio (INR) and/or signs/symptoms of hepatitis may meet the criteria for withholding or permanent discontinuation of IP or other protocol-required therapies as specified in the FDA Guidance for Industry Drug-Induced Liver Injury: Premarketing Clinical Evaluation, July 2009. See Appendix 5 (Section 10.5) for guidance on the assessment and management of abnormal hepatic laboratory values.

6.7. Access to Investigational Product after the End of the Study

Patients who complete CY 6031 and meet eligibility criteria will be offered participation in an open-label extension trial. Participation in the open-label extension trial is at the discretion of the patient and not a condition of participation in CY 6031. The commitment to the conduct of an open-label extension trial will be at Cytokinetics's discretion.

7. TEMPORARY INTERRUPTION OF INVESTIGATIONAL PRODUCT, DISCONTINUATION OF INVESTIGATIONAL PRODUCT, AND PATIENT CONSENT WITHDRAWAL

Emergent safety concerns should be discussed with the Medical Monitor immediately upon occurrence or awareness to determine if the patient should continue, interrupt or permanently discontinue IP.

Unless a safety concern arises, the investigator should make every effort to keep a patient on the IP for as long as possible during the trial. The degree to which a patient withdraws from the trial varies. There are three types of discontinuation: temporary IP interruption, permanent IP discontinuation and patient withdrawal of consent.

7.1. Temporary IP Interruption

Initially, any IP interruption should be considered temporary unless permanent IP discontinuation is mandated by the protocol.

A temporary IP interruption:

- Will be implemented when a predefined safety threshold has been met (Section 6.6)
- May be considered by the investigator in the case of an AE/SAE or for another reason

If a temporary IP interruption occurred because a safety threshold was met, blinded treatment will be resumed at least 7 days later, either at a lower dose or with a permanent switch to placebo if the patient was at 5mg, as determined by the IWRS (Section 6.6.3).

If the IP was temporarily interrupted because of an AE/SAE, the investigator should make the best effort to resume IP as soon as practically possible, assuming there are no remaining safety concerns.

If dosing is interrupted for more than 3 consecutive days in the first 6 weeks and more than 7 consecutive days thereafter, the investigator should contact the Medical Monitor to discuss the patient.

All temporary IP interruptions of greater than 3 days should be recorded in the eCRF (stop and start dates and reason for interruption) and the Medical Monitor should be notified.

7.2. Permanent Discontinuation of IP

In all cases, patients should be encouraged to discuss stopping IP with the investigator or the investigator’s designee. Best efforts should be made to address the patient’s questions, adjust concomitant medical therapies if needed and arrange follow-up safety assessments. Refer to Section 7.2.1. for management of patients that permanently discontinue IP.

Any permanent discontinuation of IP should be recorded in the eCRF including the reason for permanent discontinuation.

Reasons for permanent IP discontinuation may include any of the following:

- Patient request
- Pregnancy

- All criteria for possible drug-induced liver injury (DILI) are met (Appendix 5 [Section 10.5])
- The investigator judges that continued administration of IP would be detrimental to the patient's safety or well-being.
- Protocol violation
- Lost to follow-up
- Any breaking of the trial blind requested by the investigator
- Death
- The Sponsor requests that the patient permanently discontinue IP

7.2.1. Management of Patients after Permanent Discontinuation of IP

If IP is permanently discontinued, the patient should be encouraged to remain in the trial to continue to obtain outcome measures and safety data (see Section 7.2).

There are several options for a patient after permanently discontinuing IP:

- Patient agrees to continue to return to clinic for all remaining trial visits.
- Patient agrees to complete the early discontinuation (ED) visit as soon as possible after the decision is made and complete an EOS visit.
- Patient only agrees to complete the early discontinuation (ED) visit.
- Patient agrees to be contacted by phone to obtain patient trial data.
- Patient withdraws consent (see Section 7.4) and does not agree to any further trial procedures or visits.
- For those patients that have not withdrawn consent and have difficulty returning for all remaining trial visits, they can be contacted by phone to obtain patient trial data.

7.3. Discontinuation from Trial Procedures

Patients can decline to continue receiving IP and/or other protocol-required therapies or procedures at any time during the trial but continue participation in the trial. If this occurs, the investigator is to discuss with the patient the appropriate processes for discontinuation from IP or other protocol-required therapies and must discuss with the patient the options for continuation of the SoA (Section 1.3) including different options of follow-up (eg, in person, by phone/mail, through family/friends, in correspondence/communication with other treatment physicians, from the review of medical records) and collection of data, including endpoints and AEs. Patients who have discontinued IP and/or protocol required therapies or procedures should not be automatically removed from the trial. Whenever safe and feasible it is imperative that patients remain on-trial to ensure safety surveillance and/or collection of outcome data. The investigator must document the change to the SoA (Section 1.3) and the level of follow-up that is agreed to by the patient (eg, in person, by telephone/mail, through family/friends, in correspondence/communication with other physicians, from review of the medical records).

7.4 Patient Consent Withdrawal

Patients have the right to withdraw consent and no longer participate in the trial at any time and for any reason without prejudice to their future medical care by the physician or at the institution. Consent withdrawal means the patient no longer wishes to undergo any follow-up visits, trial procedures, investigator contact, and non-patient contact follow-up (eg, medical records check).

- Discontinuing IP should be distinguished from consent withdrawal for follow-up since the patient may agree to undergo trial procedures or still be contacted even though they have stopped taking IP (see Section 7.3).
- Consent withdrawal should be accompanied by documentation of the reason for withdrawal. Patients requesting consent withdrawal for any follow-up should be informed that it may limit the public health value of the trial.

Patients who withdraw consent should be asked explicitly about the contribution of possible AEs to their decision to withdraw consent, and any AE information elicited should be documented.

Preferably, the patient should withdraw consent in writing and, if the patient or the patient's representative refuses or is physically unable, the site should document and sign the reason for the patient's failure to withdraw consent in writing. The ICF for the trial may note that although a patient is completely free to leave the trial and stop taking IP, the investigators hope the patient will remain for follow-up status evaluations.

For patients who have withdrawn consent for further follow-up, investigators may review public records as permitted by applicable law to determine vital status of the patient before or at the end of the trial.

7.5. Lost to Follow up

A patient will be considered lost to follow-up if he or she repeatedly fails to return for scheduled visits and is unable to be contacted by the site.

The following actions must be taken if a patient fails to return to the clinic for a required trial visit:

- The site must attempt to contact the patient or the patient's family and reschedule the missed visit as soon as possible and counsel the patient on the importance of maintaining the assigned visit schedule and ascertain whether the patient wishes to and/or should continue in the trial.
- Before a patient is deemed lost to follow up, the investigator or designee must make every effort to regain contact with the patient or the patient's family (where possible, 3 telephone calls and, if necessary, a certified letter to the patient's last known mailing address or local equivalent methods). These contact attempts should be documented in the patient's medical record.
- Should the patient continue to be unreachable, he/she will be considered to have discontinued from the trial and is lost to follow-up.

Closing of specific sites or discontinuation of the trial are handled as part of Appendix 1 (Section 10.1.8).

8. TRIAL ASSESSMENTS AND PROCEDURES

Trial assessments and procedures and their timing are summarized in the SoA (Section 1.3). Protocol waivers or exceptions are not allowed. Adherence to the trial design requirements, including those specified in the SoA, is essential and required for trial conduct.

8.1. Visit Windows

There will be a total of 10 in-person trial visits per patient, with windows to aid in scheduling as shown in Table 4. If a patient visit must be scheduled outside the visit window, the Medical Monitor should be contacted.

Table 4: CY 6031 Visit Windows

Visit	Visit Window
Screening	Up to 42 days prior to Day 1 visit
Day 1 (First Dosing Day)	N/A
Week 2[a]	+3 days
Week 4[a]	+3 days
Week 6[a]	+3 days
Week 8[a]	+3 days
Week 12	±3 days
Week 16	±7 days
Week 20	±7 days
Week 24 (EOT)	±7 days
Week 28 (EOS)	+7 days

[a] Visit should occur at least 14 days but less than 17 days from the previous visit.

8.2. Visit Schedule

Activities will be completed during clinic visits as described in this section. General guidance for the preferred order of assessments and procedures are outlined below:

- On visit days, patients should wait to take their daily IP dose at the clinic.
- Patient-reported outcomes questionnaires should be completed by patients prior to any other activities. (ie, KCCQ (administered first), EQ-5D-5L, SAQ-7, Patient Global Impression of Change [PGI-C])
- IP should be administered after completion of vital signs, ECG, and blood draws.
- ECGs and vital signs must be performed prior to blood draws or other invasive procedures.
- Echocardiograms should be done prior to IP dosing on Day 1 and 2 hours after IP dosing in the clinic at other time points.

- CPET should be performed after other visit activities including IP administration.

Please refer to the study manual for additional details.

8.2.1. Screening Visit

All screening evaluations must be completed and reviewed to confirm that potential patients meet all eligibility criteria. The investigator will maintain a screening log to record details of all patients screened and to confirm eligibility or record reasons for screening failure, as applicable.

Laboratory values obtained at screening and reported through the central laboratory will be used to determine patient eligibility.

The screening period will be up to 6 weeks in duration to allow greater flexibility for visit scheduling and the potential for retesting.

The CPET should not be completed until the patient is otherwise deemed eligible. The CPET used for eligibility should be completed within three weeks but not less than one week prior to randomization.

8.2.2. Day 1

Patients who meet all eligibility criteria including confirmation from the CPET core laboratory at the end of the screening period will return to the site for randomization and Day 1 activities as defined in the SoA.

8.2.3. Weeks 2 through 20

Please refer to the SoA (Section 1.3) for Weeks 2-20.

8.2.4. Week 24: End of Treatment Visit

Week 24 is defined as the EOT visit and includes assessments and procedures critical for analysis of the trial’s endpoints. These assessments are outlined in the Schedule of Assessments. Additionally, the following should be considered in advance of Week 24:

- Contact patients shortly before the Week 24 EOT visit to confirm their ability to perform the CPET, and schedule the procedure to ensure it is completed within the protocol-defined window
- For patients temporarily unable to exercise on the treadmill or bicycle (whichever modality was used at baseline) due to an adverse event (eg, ankle sprain, upper respiratory infection, migraine), but not due to HCM symptoms, or if the site is unable to perform the CPET (eg, equipment malfunction), then the Week 24 visit may be postponed by up to 4 weeks and those patients should continue to receive IP until the visit
- Schedule CMR imaging for sub-study (if applicable)
    - For patients who provided consent and completed baseline imaging at screening, schedule the CMR imaging to ensure it is completed within the protocol-defined window

8.2.5. Week 28: End of Study Visit

All patients should complete an end of study (EOS) visit:

- For subjects who complete all study visits, the EOS visit will occur at Week 28 (or 4 weeks after a delayed Week 24 visit).
- For subjects who early-terminate IP more than 4 weeks before Week 24 and continue to stay on-study for all follow-up assessments, the Week 24 visit can be considered the EOS visit (and the EOS visit does not need to be performed).
- For subjects who withdraw consent and do not wish to continue the participation in follow-up assessments, and EOS visit should be performed 4 weeks after their final IP dose if possible.

To ensure a 4-week safety follow-up (to assess any Adverse Events), the EOS Exit Date must correspond to either 4 weeks after last dose, or at their last study visit (ie., Week 24)—whichever is later.

8.2.6. Early Discontinuation Visit

For patients who discontinue the trial prematurely, the activities outlined in the SoA will be completed during an Early Discontinuation visit as soon as possible.

8.2.7. Unscheduled Visit

Assessments may be completed at the investigator's discretion during an Unscheduled visit. In addition, assessments performed at a scheduled visit that are not defined in the SOA will be considered unscheduled assessments.

8.3. Efficacy Assessments

8.3.1. Cardiopulmonary Exercise Testing

All patients will undergo CPET with gas-exchange analysis and the methodology will be standardized across all participating sites, as described in the CPET manual. Testing will include continuous ECG monitoring by trained personnel and be performed in an area that is equipped for cardiopulmonary resuscitation. Treadmill is the preferred modality for exercise testing. For CPET laboratories that do not perform treadmill testing, cycle ergometry is an acceptable alternative. Exercise protocols for both modalities will be provided in the CPET manual. Patients must use the same testing modality for all exercise tests during the trial. Whenever possible, CPET should be administered by the same trial personnel using the same equipment and performed after the other trial procedures on that visit day (including echocardiogram, KCCQ, EQ-5D-5L, CGI, PGI-C, NYHA class, SAQ-7, vital signs, ECG, blood sampling, IP administration). Patients naïve to exercise protocols will be familiarized with the technique during screening.

All CPET testing will be symptom-limited and patients will be strongly encouraged to achieve maximal exertion and an RER $\geq$1.05. The reason(s) for termination of sub-maximal exercise tests will be documented. A test will be identified as being maximal effort if the RER is $\geq$1.05.

The Week 24 CPET should be performed at approximately the same time of day (eg, morning, mid-day, afternoon) as the baseline CPET at screening, at a consistent time after the last dose of beta-blocker and IP. Whenever possible, patients should perform exercise testing between three and ten hours after taking beta blocking agents.

If a life-threatening arrhythmia, early ischemia, severe hypotension or other serious finding is identified by the investigator during CPET, the patient will be asked to stop the exercise test, and his/her physicians will be notified of the results. If the patient is performing the screening test, s/he will not be randomized to the trial. Enrolled patients who have a non-life-threatening event or finding that stops the test can resume testing when it is safe to do so and after appropriate treatment, per the investigator.

All sites must be qualified by the CPET core laboratory prior to the initiation of screening. To qualify, sites will perform an exercise test on two healthy adults and submit them for core laboratory review. Sites may be required to submit additional normal exercise tests during the conduct of the trial for review by the CPET core laboratory in order to confirm proper function of testing equipment. Sites may be qualified based on exercise tests recently reviewed by the CPET core laboratory during the conduct of other trials.

8.3.2. Echocardiography

Echocardiography will be done during screening and prior to dosing on Day 1. Echocardiography will also be performed 2 hours after dosing in the clinic on Weeks 2, 4, 6, 8, 12, 16, 20, 24, and 28.

Certified sonographers will perform echocardiography using standard high-quality, high-fidelity machines approved by Cytokinetics. Whenever possible, the same sonographer will perform all studies for a single patient. Echocardiograms will be performed after the patient has been resting in a supine position for at least 10 minutes and in accordance with the echocardiography manual. Instructions for the performance of the Valsalva maneuver and imaging the LVOT-G will also be included in the echocardiography manual.

When echocardiograms are scheduled at the same time as blood draws, vital signs, and/or ECGs, the order of evaluation will be vital signs, ECGs, blood draw and echocardiogram. The blood draw should be obtained at the scheduled time point and the echocardiograms will follow.

Echocardiographic parameters to be measured will at least include the left ventricular parameters in Table 5 in addition to right heart function metrics detailed in the echocardiography protocol.

Table 5: CY 6031 Echocardiographic LV Parameters to be Measured

Resting LVOT-G	LVEDV	IVST
Post-Valsalva LVOT-G	LVESD	IVCT
LVEF	LVESV	IVRT
LVFS	LVCO	E/E' ratio (septal and lateral)
GLS	LV Stroke Volume	LAV
LVEDD	LVOT VTI	

GLS = global longitudinal strain; IVCT = isovolumic contraction time; IVRT = isovolumic relaxation time; IVST = interventricular septum thickness; LAV = left atrial volume; LVCO = left ventricular cardiac output; LVEDD = left ventricular end diastolic diameter; LVEDV = left ventricular end diastolic volume; LVESD = left ventricular end systolic diameter; LVESV = left ventricular end systolic volume; VTI = velocity time integral.

Unscheduled echocardiograms may be obtained when clinically indicated, for example to assess an AE or follow-up a clinically significant change in a prior echocardiogram, as determined by the investigator. Results will be interpreted by the unblinded Echo Cardiologist at the investigational site.

All echocardiograms (including unscheduled) will be sent to the core laboratory for interpretation. On-site interpretation of LVEF and LVOT-G will be used for dose escalation and reduction decisions via IWRS. The core laboratory quantification of the echocardiograms will be used for all statistical analyses.

8.3.3. Cardiac Magnetic Resonance

A CMR imaging sub-study will assess the effects of administration of CK-3773274 dosing on cardiac morphology, function, and fibrosis in approximately 40 oHCM patients who are eligible and consent to participate. CMR will be performed during screening period and Week 24.

Patients with eGFR <30 mL/min/1.73 m^2 or an allergy to gadolinium may have a non-contrast CMR.

8.3.4. New York Heart Association Functional Classification

After interviewing the patient, the investigator (or qualified designee) will record the NYHA Functional Classification in the CRF (Criteria Committee of the New York Heart 1994). The NYHA classification is as follows:

- Class I - No symptoms and no limitation in ordinary physical activity (eg, shortness of breath when walking, climbing stairs)
- Class II - Mild symptoms (eg, mild shortness of breath and/or angina) and slight limitation during ordinary activity.
- Class III - Marked limitation in activity due to symptoms, even during less-than-ordinary activity (eg, walking short distances [20-100 m]). Comfortable only at rest.
- Class IV - Severe limitations. Experiences symptoms even while at rest. Mostly bedbound patients.

8.3.5. Clinical Global Impression Scale

The investigator (or qualified designee) will record the CGI scale assessment of the patient's global functioning in the CRF at the time points outlined in the SoA.

8.3.6. Patient-Reported Outcomes

The following questionnaires will be completed at trial visits specified in the SoA (Section 1.3):

- KCCQ
- EQ-5D-5L
- PGI-C
- SAQ-7

Patients will be asked to complete the KCCQ, EQ-5D-5L, SAQ-7 and PGI-C questionnaires in a quiet place prior to the medical consultation and prior to undergoing any tests and procedures to avoid biasing their responses.

Site staff will verify the questionnaires for completeness before the patients leave the clinic or hospital.

8.4. Safety Assessments

Planned time points for all safety assessments are provided in the SoA (Section 1.3).

8.4.1. Physical Examinations

A complete physical examination will include, at a minimum, assessments of the cardiovascular, respiratory, and neurological systems. Breast, genital, and rectal examinations are not required unless specific evaluation is warranted.

Investigators should pay special attention to clinical signs related to previous serious illnesses.

Physical examinations may be conducted at any time during the treatment period if clinically indicated.

8.4.2. Height and Weight

Height and weight will be measured while patient is fully clothed with shoes removed. Height will be measured at screening only.

8.4.3. Vital Signs

At the Screening visit, a room air oxygen saturation will be assessed.

At all visits, heart rate, respiratory rate, and blood pressure will be assessed.

Blood pressure and heart rate measurements will be assessed with the patient in a supine or sitting position. Blood pressure and heart rate measurements should be performed with an automated oscillometer after the patient has rested for at least 5 minutes in a quiet setting without distractions (eg, television, cell phones). The position selected for a patient should be the same that is used throughout the trial.

Vital signs (to be taken before blood collection for laboratory tests) will consist of 1 heart rate reading and 3 consecutive blood pressure readings recorded at intervals of at least 1 minute. The average of the 3 blood pressure readings will be recorded in the CRF.

8.4.4. Electrocardiograms

Triplicate 12-lead ECGs will be obtained as outlined in the SoA (Section 1.3) using an ECG machine that automatically calculates the heart rate and measures PR, QRS, QT, and QTc intervals.

At each time point at which triplicate ECGs are performed, three individual ECG tracings should be obtained as closely as possible in succession, but no more than 2 minutes apart.

Patients should be sitting or supine in a rested and calm state for at least 5 minutes prior to the ECG. The investigator may perform additional ECG recordings as needed for the care of the patient.

A patient will be withdrawn from the trial by the investigator or designee if, in their medical judgment, ECG findings are present which make continued trial participation not in the patient's best interest.

For safety monitoring purposes, the investigator or designee must review, sign, and date all ECG tracings.

Unscheduled ECGs may be collected at additional time points, for example in case of an AE or based on vital signs, PK results or PD results, as determined by the investigator or the Medical Monitor.

All ECG tracings will be kept as part of the patient's permanent trial file at the site. Digital recordings will be analyzed and stored at a central ECG laboratory.

8.4.5. Laboratory Assessments

See Appendix 2 (Section 10.2) for the list of clinical laboratory tests to be performed and see the SoA (Section 1.3) for the timing and frequency.

Repeat or unscheduled samples may be taken for safety reasons or for technical issues with the samples.

The investigator must review the laboratory report, document this review, and record any clinically significant changes occurring during the trial in the AE section of the CRF. Clinically significant abnormal laboratory findings are those which are not associated with the underlying disease, unless judged by the investigator to be more severe than expected for the patient's condition.

All protocol-required laboratory assessments, as defined in Appendix 2 (Section 10.2), must be conducted in accordance with the laboratory manual and the SoA (Section 1.3).

8.5. Adverse Events and Serious Adverse Events

8.5.1. Adverse Events

8.5.1.1. Definition of Adverse Event

An **adverse event (AE)** is defined as any untoward medical occurrence in a patient or clinical investigation patient administered a pharmaceutical product and which does not necessarily have a causal relationship with this treatment. An AE can therefore be any unfavorable and unintended sign (including an abnormal laboratory finding), symptom, or disease temporally associated with the use of IP, whether or not related to the IP.

Adverse events include:

- Exacerbation of a chronic or intermittent pre-existing condition including either an increase in frequency and/or intensity of the condition.
- New conditions detected or diagnosed after IP administration even though it may have been present before the start of the trial.
- Abnormal assessments, eg, change on physical examination, ECG findings, if they represent a clinically significant finding that was not present at trial start or worsened during the course of the trial.
- Laboratory test abnormalities if they represent a clinically significant finding, symptomatic or not, which was not present at trial start or worsened during the course of the trial, require treatment or led to dose reduction, interruption or permanent discontinuation of IP. In general, abnormal laboratory findings without clinical significance (based on the investigator's judgment) are not to be recorded as adverse events. Where applicable, clinical sequelae (not the laboratory abnormality) are to be recorded as the adverse event.
- Signs, symptoms, or the clinical sequelae of a suspected drug-drug interaction.
- Signs, symptoms, or the clinical sequelae of a suspected overdose of either IP or a concomitant medication. Overdose per se will not be reported as an AE/SAE unless it is an intentional overdose taken with possible suicidal/self-harming intent. Such overdoses should be reported regardless of sequelae.

8.5.1.2. Definition of Serious Adverse Event

A **serious adverse event** (SAE) is defined as any untoward medical occurrence that at any dose:

- results in death,
- Is life threatening, NOTE: The term "life-threatening" in the definition of "serious" refers to an event in which the patient was at risk of death at the time of the event; it does not refer to an event which hypothetically might have caused death if it were more severe
- Requires inpatient hospitalization or prolongation of existing hospitalization
- Results in persistent or significant disability/incapacity, or

- Is a congenital anomaly/birth defect
- Important medical event

Medical and scientific judgement should be exercised in deciding whether expedited reporting is appropriate in other situations, such as important medical events that may not be immediately life-threatening or result in death or hospitalization but may jeopardize the patient or may require intervention to prevent one of the other outcomes listed in the definition above. These should also usually be considered serious. Examples of such events include invasive or malignant cancers, intensive treatment in an emergency room or at home for allergic bronchospasm; blood dyscrasias or convulsions that do not result in hospitalization; or development of drug dependency or drug abuse.

An adverse event would meet the criterion of "requires hospitalization," if the event necessitated an admission to a health care facility (eg, overnight stay).

The following reasons for hospitalization are exempted from being reported:

- Hospitalization for cosmetic elective surgery, or social and/or convenience reasons.
- Hospitalization for pre-planned (ie, planned prior to signing informed consent) surgery or standard monitoring of a pre-existing disease or medical condition that did not worsen, eg, hospitalization for coronary angiography in a patient with stable angina pectoris.

However, complications that occur during an exempted hospitalization are AEs or SAEs (for example if a complication prolongs a pre-planned hospitalization).

8.5.1.3. Intensity of Adverse Events

The investigator must assess the intensity for each AE and SAE reported during the trial according to a three-point scale: mild, moderate, severe.

If the intensity of an AE worsens during IP administration, only the worst intensity should be reported on the AE page. If the AE lessens in intensity, no change in the severity is required.

The three categories of intensity are defined as follows:

**Mild**	The event is noticeable to the patient. It does not influence daily activities, and does not require intervention.
**Moderate**	The event makes the patient uncomfortable. Performance of daily activities are influenced, and intervention is needed.
**Severe**	The event causes noticeable discomfort, and interferes with daily activities. The patient may not be able to continue in the trial, and treatment or intervention is needed.

A mild, moderate, or severe AE may or may not be serious. Medical judgment should be used on a case-by-case basis.

Seriousness, rather than severity assessment, determines the regulatory reporting obligations.

8.5.1.4. Relationship to Investigational Product

Each AE must be assessed by the investigator, based on clinical judgment, as to whether or not there is a reasonable possibility of causal relationship to the IP and reported as either related or unrelated.

- A "reasonable possibility" of a relationship conveys that there are facts, evidence, and/or arguments to suggest a causal relationship, rather than a relationship cannot be ruled out.
- Alternative causes, such as underlying disease(s), concomitant therapy, and other risk factors, as well as the temporal relationship of the event to IP administration will be considered and investigated.
- For each AE/SAE, the investigator must document in the medical notes that he/she has reviewed the AE/SAE and has provided an assessment of causality.
- There may be situations in which an SAE has occurred and the investigator has minimal information to include in the initial report to Cytokinetics. However, it is very important that the investigator always assesses causality for every event before the initial transmission of the SAE data to Cytokinetics.
- The investigator may change his/her opinion of causality considering follow-up information and send an SAE follow-up report with the updated causality assessment.

8.5.1.5. Relationship to Trial Procedures

An AE is defined as related to trial procedures if it appears to have a reasonable possibility of a causal relationship to protocol-required procedures.

8.5.1.6. Reporting of AEs

The investigator is responsible for ensuring that all adverse events observed by the investigator or reported by the patient that occur after starting the IP through study exit are recorded in the AE eCRF.

Only adverse events considered related to trial procedures are reported after signing of the informed consent until IP administration.

Medical occurrences that are not associated with trial procedures and that begin before the start of IP but after signing of the ICF will be recorded on the Medical History/Current Medical Conditions CRF, not the AE CRF.

8.5.1.7. Reporting Procedures for SAEs

Prompt notification by the investigator to Cytokinetics of an SAE is essential so that legal obligations and ethical responsibilities towards the safety of patients and the safety of an IP under clinical investigation are met.

The investigator is responsible for ensuring that all SAEs observed by the investigator or reported by the patient that occur after informed consent through end of study, or 4 weeks after the last administration of IP, whichever is later, are reported to Cytokinetics on an SAE Report Form within 24 hours following the investigator's knowledge of the event and recorded in the

AE eCRF. These events must be reported regardless of the investigator-attributed causal relationship with IP or protocol-related procedures.

The SAE Report forms must be emailed or faxed to Cytokinetics Drug Safety (contact details are provided on the SAE Report form):

Email: **CY6031DrugSafety@cytokinetics.com**

Facsimile: **+1 (650) 243-4199**

The investigator must attempt to establish a diagnosis of the event based on signs, symptoms, and/or other clinical information. Whenever possible, the diagnosis (not the individual signs/symptoms) will be documented as the AE/SAE.

The investigator must complete the SAE Report form in English and must assess the causal relationship of the event to IP.

If the patient is hospitalized in a hospital other than the trial site, it is the investigator's responsibility to contact this hospital to obtain all SAE relevant information and documentation.

New information relating to a previously reported SAE must be reported to Cytokinetics within 24 hours following knowledge of the new information. Cytokinetics Drug Safety may contact the investigator to obtain further information.

8.5.1.8. Follow-up of AEs and SAEs

The investigator is obligated to perform or arrange for the conduct of supplemental measurements and/or evaluations as medically indicated or as requested by Cytokinetics to elucidate the nature and/or causality of the AE or SAE as fully as possible. This may include additional laboratory tests or investigations, histopathological examinations, or consultation with other health care professionals.

If a patient dies during participation in the trial or during a protocol-defined follow-up period, the investigator will provide Cytokinetics with a copy of any post-mortem findings including histopathology if it has been performed.

Non-serious adverse events must be followed until they resolve or until the patient completes the trial, whichever comes first.

Serious adverse events still ongoing at the end of study must be followed up until resolution or stabilization, or until the event outcome is provided, eg, death. Reporting after study exit to Drug Safety may continue after the EOS visit.

New SAEs occurring after the 4-week follow-up period must be reported to the Cytokinetics drug safety department within 24 hours of the investigator's knowledge of the event, **only** if considered by the investigator to be causally related to previous exposure to the IP.

8.5.1.9. Regulatory Reporting

The reference safety document used for the assessment of expectedness of a suspected serious adverse reaction for the purpose of expedited reporting to Health Authorities, IRBs/IECs, and investigators is the reference safety information section of the Investigator's Brochure [CK-3773274 IB].

Cytokinetics will report SAEs and/or suspected unexpected serious adverse reactions as required to regulatory authorities, investigators/institutions, and IRBs/IECs in compliance with all reporting requirements according to local regulations and good clinical practice.

The investigator is to notify the appropriate IRB/IEC of SAEs occurring at the site and other adverse event reports received from Cytokinetics, in accordance with local procedures and statutes.

8.5.1.10. Pregnancy and Breastfeeding

If a woman becomes pregnant while on IP, IP must be discontinued. The investigator must counsel the patient and discuss the risks of continuing with the pregnancy and the possible effects on the fetus.

Please refer to Appendix 3 (Section 10.3) regarding contraceptive guidance.

Irrespective of the treatment received by the patient, any pregnancy occurring in a female patient, or female partner of a male patient, after starting the IP up to 4 weeks following IP discontinuation must be reported to Cytokinetics within 24 hours of the investigator's knowledge of the event.

Pregnancies must be recorded in the eCRF and reported on the Cytokinetics Pregnancy form, which is emailed or faxed to Cytokinetics Drug Safety (contact details are provided on the Pregnancy Report form):

Email: **CY6031DrugSafety@cytokinetics.com**

Facsimile: **+1 (650) 243-4199**

Details of all pregnancies in female patients and female partners of male patients will be collected after the start of IP and until the conclusion of the pregnancy.

Any pregnancy complication or elective termination of a pregnancy for medical reasons must be reported as an AE or SAE.

Abnormal pregnancy outcomes (eg, spontaneous abortion, fetal death, stillbirth, congenital anomalies, ectopic pregnancy) are considered SAEs.

Any post-trial pregnancy-related SAE considered reasonably related to the IP by the investigator will be reported to Cytokinetics as described in Section 8. While the investigator is not obligated to actively seek this information in former trial patients, he or she may learn of an SAE through spontaneous reporting.

Male Patients with Partners Who Become Pregnant

If the partner of a male subject becomes pregnant while on study drug, he may continue receiving treatment; however, he must use barrier method (ie, condom) during sexual intercourse to avoid further fetal exposure.

The investigator will attempt to collect pregnancy information on any male patient's female partner who becomes pregnant while the male patient is in this trial.

After obtaining the necessary signed ICF from the pregnant female partner directly, the investigator must complete the Pregnancy Report Form and submit it to Cytokinetics within

24 hours of receipt of the partner's consent. The female partner will also be followed to determine the outcome of the pregnancy. Information on the status of the mother and child will be forwarded to Cytokinetics.

Female Patients Who Breastfeed

If a female patient breastfeeds while on study drug, study drug will be discontinued.

The investigator will collect breastfeeding information on any female patient who breastfeeds while taking the IP through 14 days after the end of study drug treatment. The mother and infant health information will be recorded on the Pregnancy Report Form and submitted to Cytokinetics immediately and no later than 24 hours following the investigator's knowledge of event.

8.6. Treatment of Overdose

For this trial, any dose of IP that exceeds the protocol-specified dose or dosing frequency will be considered an overdose.

There is no established treatment for an overdose. In the event of overdose, monitor for signs and symptoms including but not limited to hypotension, cardiac dysrhythmia, tachycardia, tachypnea, peripheral and pulmonary edema, decrease renal function, dizziness, dyspnea, palpitation, fatigue. The use of rescue medications (eg, dobutamine) to treat a low cardiac output state is recommended if necessary.

If a patient experiences low cardiac output due to systolic dysfunction, the investigator should follow appropriate regional heart failure treatment guidelines.

The date and time of rescue medication administration as well as the name and dosage regimen of the rescue medication must be recorded.

In the event of an overdose, the investigator should:

- Contact the Medical Monitor immediately who may recommend:
  a. Close monitoring of the patient for any AEs/SAEs and laboratory abnormalities.
  b. Obtaining a plasma sample for PK analysis as soon as practical and note the date of the last dose of IP.
- Document the quantity of the excess dose as well as the duration of the overdose in the CRF.

Decisions regarding dose interruptions or modifications will be made by the investigator in consultation with the Medical Monitor based on the clinical evaluation of the patient.

8.7. Pharmacokinetics

Seventeen blood samples of approximately 4 mL will be collected for measurement of plasma concentrations of CK-3773274 as specified in the SoA (Section 1.3) and Table 6 below. Samples will be used to evaluate the PK of CK-3773274 and potentially its metabolites. Instructions for the collection and handling of biological samples will be provided in the laboratory manual.

The actual date and time (24-hour clock time) of each sample will be recorded. The time of administration of IP on the day of PK sampling will be recorded in the CRF. It is important to

provide instructions to patients that they should not take their dose on the day of their clinic visit until in the clinic.

See Table 6 for a summary of PK sampling time points. All samples should be drawn within ±10 minutes of the scheduled time point. Drug concentration information that would unblind the trial will not be reported to investigative sites or blinded personnel until the trial has been unblinded.

Table 6: Summary of PK Time Points

Visit	PK Time Point
Day 1	Pre-dose, 2 hr
Week 2	Pre-dose, 2 hr
Week 4	Pre-dose, 2 hr
Week 6	Pre-dose, 2 hr
Week 8	Pre-dose, 2 hr
Week 12	Pre-dose, 2 hr
Week 16	Pre-dose, 2 hr
Week 20	Pre-dose, 2 hr
Week 24 (EOT)	Pre-dose, 2 hr
Early Discontinuation	untimed

8.8. Genetics

As HCM is a genetic disease, blood and/or DNA from patients who consent may be analyzed through the use of whole genome sequencing, whole exome sequencing, next-generation sequencing, and/or other method to identify genetic variants and mutations that are predictive of patient phenotype, response to IP, resistance to IP, metabolism of IP, susceptibility to developing AEs, or to increase the knowledge and understanding of cardiovascular, muscle and disease biology. In the event of DNA extraction failure, a replacement genetic blood sample may be requested from the patient.

See Appendix 4 (Section 10.4) for information regarding genetic research. Details on processes for collection and shipment and destruction of these samples can be found in the laboratory manual.

8.9. Serum for Biomarker Analysis

Blood will be collected for analysis of serum biomarkers. See Section 10.2 for list of biomarkers.

8.10. Serum Collection for Future Analyses

Serum will be collected and banked in this trial for future research on biomarkers. Serum will be stored in a long-term storage facility designated by Cytokinetics for up to 20 years.

8.11. Immunogenicity Assessments

No immunogenicity assessments will be done for this trial.

9. STATISTICAL CONSIDERATIONS

9.1. Statistical Hypotheses

The analyses evaluating treatment effect on the primary and secondary efficacy endpoints will test the null hypothesis that there is no treatment difference between patients receiving placebo and those receiving CK-3773274 in the full analysis set (FAS). Adjustments for multiplicity will be specified in Section 9.4.1.1.

9.2. Sample Size Determination

Assuming a difference in change from baseline in pVO_2 of 1.5 mL/kg/min for CK-3773274 compared to placebo, a standard deviation (SD) of 3.5 mL/kg/min, accounting for limiting beta-blocker use (less than ~70%), limiting exercise modality of bicycle (less than ~50%) and 10% of patients missing change from baseline data of the primary endpoint, a sample size of 270 patients at randomization ratio of 1:1 (approximately 135 randomized to CK-3773274 and 135 randomized to placebo) provides more than 90% power to detect the difference in pVO_2 change from baseline to Week 24 with a 2-sided type I error of 0.05.

9.3. Populations for Analyses

The analysis populations are defined in Table 7.

Table 7: Analysis Sets

Analysis Set	Description
All Randomized Set	All randomized patients.
Full Analysis Set	All randomized patients who receive at least one dose of randomized IP and have at least one post-baseline efficacy measurement. Patients will be analyzed according to their randomized treatment group assignment. Efficacy endpoints will be analyzed based on the FAS.
Safety Analysis Set	All randomized patients who received at least one dose of IP, CK-3773274 or placebo. Patients will be analyzed by their randomized treatment group assignment. If a patient receives treatment throughout the study that is different than the randomized treatment group assignment, then this patient will be grouped by the actual treatment group
Pharmacokinetics Analysis Set (PKS)	All randomized patients who have at least one evaluable plasma concentration of CK-3773274, provided they have no major protocol violations deviations that could affect the PK of CK-3773274.

9.4. Statistical Analyses

The Statistical Analysis Plan (SAP) will be finalized prior to database lock and it will include a more technical and detailed description of the statistical analyses described in this section. This

section is a summary of the planned statistical analyses of the most important endpoints including primary and key secondary endpoints.

9.4.1. General Considerations

Summary tables will present descriptive statistics such as number of patients, mean, median, standard deviation, minimum and maximum for continuous variables, and number of patients and the percentage for categorical variables, overall and by treatment in the planned analysis sets. For model-based analysis, least squares means, difference of least squares means between treatments, their standard errors and 95% confidence intervals (CI), and two-sided p-values for the relative statistical inferences will be presented. Baseline is defined as the last available measurement taken before the first dose of randomized IP unless otherwise specified. Listings will include patient ID, demographics, treatment assigned and other relevant items, and sorted by treatment assignment, patient ID and date of assessment. Unless specified otherwise, efficacy, safety and pharmacokinetics analyses will be performed on the full analysis set, safety analysis set and pharmacokinetics analysis set, respectively. Statistical analysis methods will be detailed in the SAP.

9.4.1.1. Multiplicity Adjustment

The null hypothesis for the primary and secondary efficacy variables in the FAS will be tested in the pre-specified order using a closed testing procedure.

For preservation of the overall type I error rate at two-sided 0.05 for the primary and secondary endpoints, the primary endpoint is tested first at two-sided 0.05. If the primary endpoint achieves statistical significance at two-sided $p \leq 0.05$, then a parallel gatekeeper method with two-sided 0.025 separately allocated to Week 12 and to Week 24 is applied for the first four secondary endpoints, with their testing being in the sequential order of KCCQ-CSS change from baseline, proportion of patients with ≥1 NYHA functional class improvement, post-Valsalva LVOT-G change from baseline and proportion of patients with post-Valsalva LVOT-G < 30 mm Hg. If all four of the first four secondary endpoints at Week 12 (or at Week 24) have two-sided $p \leq 0.025$, then there is recycling of the 0.025 for Week 12 (or Week 24) to Week 24 (or Week 12) so that 0.05 is applicable to the corresponding testing of the first four secondary endpoints. If $p \leq 0.025$ for the first four secondary endpoints at Week 12 (or at Week 24) and if $p \leq 0.05$ for the first four secondary endpoints at Week 24 (or at Week 12), then the fifth secondary endpoint of change from baseline to Week 24 in total workload is tested at two-sided $p \leq 0.05$. The multiple testing procedure is illustrated in Figure 1. The testing sequence of secondary endpoints of KCCQ-CSS, NYHA Functional Class and post-Valsalva LVOT-G for Week 12 and Week 24, as well as that for change in total workload at Week 24, will be detailed in the SAP.

9.4.2. Primary Endpoint(s)

The primary endpoint of the study is change in pVO_2 by CPET from baseline to Week 24.

The primary estimand is the difference in means of the change from baseline to Week 24 in pVO_2 between CK-3773274 and placebo for the target population of potentially treatable CK-3773274 patients despite intercurrent events after a first dose. Subjects without a dose of IP will be excluded from the FAS as they are anticipated to not represent patients from a treatable population defined as patients who would meet the eligibility requirements of this study and are

capable and willing to be dosed. Missing data will be imputed using multiple imputation method under the missing at random (MAR) assumption. The distribution of missing CPET data at Week 24 and the reasons for the missing data will be tabulated in the FAS. The primary analysis of the primary endpoint will use an ANCOVA model with treatment group, randomization stratification factors, baseline pVO_2 and baseline weight as covariates in the FAS. Sensitivity analyses will be performed by repeating the primary analysis examining assumptions that data are not missing at random: missing pVO_2 from patients who discontinued CK-3773274 treatment or missing pVO_2 from patients from the placebo arm will be imputed based on the model that is constructed using observed pVO_2 data from the placebo arm. Missing pVO_2 from patients who remained on CK-3773274 treatment will be imputed based on the model that is constructed using observed pVO_2 data from the CK-3773274 arm. Least squares means (LSM), LSM treatment difference and the standard error from each imputed dataset will be combined using Rubin's rules to produce an overall LSM estimate of the treatment difference, its 95% confidence interval, and p-value.

Other sensitivity analyses and the details will be included in the SAP.

9.4.3. Secondary Endpoint(s)

The secondary endpoint(s) of the study are:

- Change in KCCQ-CSS from baseline to Week 12 and Week 24
- Proportion of patients with ≥1 class improvement in NYHA Functional Class from baseline to Week 12 and Week 24
- Change in post-Valsalva LVOT-G from baseline to Week 12 and Week 24
- Proportion of patients with post-Valsalva LVOT-G < 30 mmHg at Week 12 and Week 24
- Change in total workload during CPET from baseline to Week 24

Change in KCCQ-CSS and change in post-Valsalva LVOT-G from baseline to Week 12 and Week 24 will be analyzed using a MMRM (mixed model repeated measures) model with baseline as covariate, randomization stratification factors, visit, treatment group, and interaction terms of treatment by visit and baseline by visit. An unstructured covariance matrix will be specified.

Proportion of patients with ≥1 class improvement in NYHA Functional Class from baseline to Week 12 and Week 24 or proportion of patients with post-Valsalva LVOT-G < 30 mmHg at Week 12 and Week 24 will be analyzed using Cochran– Mantel–Haenszel (CMH) test stratified by randomization factors. Patient's Week 20 NYHA Functional Class will be used when Week 24 NYHA Functional Class is not available. Patients who do not have Week 12 and/or Week 24 NYHA Functional Class will be treated as non-responders. The p-value and 95% confidence interval (CI) will be obtained using exact method. Adjustment of multiplicity of the primary and secondary endpoints is specified in Section 9.4.1.1.

9.4.4. Exploratory Endpoint(s)

The exploratory endpoints of the study are:

- Compared with baseline, number of patients at Week 24 achieving either:
  - Change from baseline of ≥1.5 mL/kg/min in pVO_2 AND ≥1 class improvement in NYHA Functional Class

  OR

  - Change of ≥3.0 mL/kg/min from baseline in pVO_2 AND no worsening of NYHA Functional Class
- Proportion of patients with improvement in KCCQ-CSS >5 points at Week 12 and Week 24
- Proportion of patients with resting LVOT-G <30 mmHg, post-Valsalva LVOT-G <50 mmHg, and NYHA Functional Class I at Week 12 and Week 24
- Proportion of patients with resting LVOT-G <30 mmHg, post-Valsalva LVOT-G <50 mmHg, and ≥1 class improvement in NYHA Functional Class at Week 12 and Week 24
- Change from baseline to Week 24 in:
  - VE/VCO_2 slope
  - VO_2 × systolic BP
  - VAT
- Change from baseline to Week 24 in individual responses to the EQ-5D-5L
- Change from baseline to Week 24 in summary and individual domain scores for the SAQ-7
- Change from baseline to Week 24 in echocardiographic measurements of cardiac structure and of systolic function including:
  - LVEF
  - LVESV and LVEDV
  - Left atrial volume
- Change from baseline values in NT-pro-BNP, hs-cardiac-TnI and other biomarkers through Week 24
- Change from baseline to Week 24 in CMR measurements of:
  - LV mass index
  - LVEF
  - Septal and free wall thickness
  - Left atrial volume index

- LVESV
- LVEDV

Proportion of responders will be analyzed using Cochran– Mantel–Haenszel (CMH) test stratified by randomization factors. Change from baseline in continuous echocardiography parameters will be analyzed using MMRM model with baseline as covariate, randomization stratification factors, visit, treatment group and treatment group by visit as interaction. An unstructured covariance matrix will be specified. Change from baseline in other CPET parameters will be analyzed using the same primary model for the primary endpoint. Change from baseline in parameters of CMR measurements and 5Q-5D-5L will be analyzed using an ANCOVA model with baseline as covariate, randomization stratification factors and treatment group as fixed effects. Median and median difference of NT-pro-BNP between treatment group and 95% confidence of the median difference will be presented. Log transformed NT-pro-BNP may be performed and analyzed using MMRM model with log baseline as covariate, visit, randomization stratification factors, treatment group as fixed effects and treatment group by visit interaction. The same model for KCCQ-CSS will be used to analyze the change from baseline in SAQ-7.

9.4.5. Safety Analysis

Safety analyses will be performed on the safety analysis set.

9.4.5.1. Adverse Events

A treatment-emergent AE is an AE with an onset after initiation of IP, or an AE present at initiation of IP dosing that worsens in severity during the treatment. AEs will be coded using MedDRA dictionary and summarized by preferred terms and system organ class. The version of the MedDRA dictionary will be specified in the clinical study report. AEs will be classified according to severity. The number and percentage of patients reporting AEs will be tabulated.

Only treatment-emergent AEs with an onset from the first dose until 4 weeks after last dose of IP will be summarized. All AEs will be included in patient listings.

Patient incidence of reported major adverse cardiac events (CV death, cardiac arrest, non-fatal stroke, non-fatal myocardial infarction, CV hospitalization) will be summarized by treatment group and event type. Patient incidence of new onset persistent atrial fibrillation, patient incidence of appropriate ICD discharges and aborted sudden cardiac death, patient incidence of LVEF <50% will be summarized by treatment group.

9.4.5.2. Serious Adverse Events

Summaries of SAEs (by preferred term and system organ class) and SAE severity will be presented.

The safety follow-up is defined as 4 weeks following the last dose of IP.

9.4.6. Pharmacokinetic Endpoints

Plasma concentrations of CK-3773274 and PK parameter C_{max} and C_{trough} will be summarized using descriptive statistics including mean, standard deviation, geometric mean, coefficient of

variation, median, and range. Geometric mean concentrations over time will be graphically displayed.

9.4.7. Patient Disposition

The number of patients who are randomized, who complete the planned treatment, and who prematurely discontinue from the planned treatment and/or the study will be presented by treatment group and overall. Reasons for premature discontinuation as recorded on the termination page of the CRF will also be summarized.

9.4.8. Demographics and Other Baseline Characteristics

Patient demographics and other baseline characteristics will be summarized descriptively by treatment group.

9.4.9. Investigational Product Exposure

IP exposure will be summarized, including the total number of doses administered, total amount of drug administered, and the total duration of IP administration, defined as the date of the last dose minus the date of first dose + 1.

9.4.10. Concomitant Medications

Concomitant medications will be summarized and classified by drug class and preferred term using the World Health Organization (WHO) Drug Dictionary. The version of the WHO Drug Dictionary will be specified in the clinical study report.

9.4.11. Clinical Laboratory Parameters

Descriptive statistics for clinical laboratory values and changes from baseline at each protocol specified assessment time point will be presented.

9.4.12. Vital Signs

Descriptive statistics for vital signs and changes from baseline at each protocol specified assessment time point will be presented.

9.4.13. Electrocardiogram

Descriptive statistics for ECG parameters (eg, heart rate, PR interval, QRS interval, QT interval, and QTc interval [both Bazett's and Fridericia's corrections]) and changes from baseline at each protocol specified assessment time point will be presented. Select ECG parameters will be analyzed using a repeated measures analyses with dose and time points as factors and baseline ECG parameter as a covariate. Dose-response trend will be estimated.

9.5. Data Monitoring Committee

An unblinded DMC will regularly review the emerging data for safety monitoring purpose. The DMC or Cytokinetics can require an ad hoc DMC meeting at any time. No study activities will be suspended during the safety review. For details on the DMC, refer to Appendix 1 (Section 10.1.5).

10. SUPPORTING DOCUMENTATION AND OPERATIONAL CONSIDERATIONS

10.1. Appendix 1: Regulatory, Ethical, and Trial Oversight Considerations

10.1.1. Regulatory and Ethical Considerations

This trial will be conducted in accordance with the protocol and with the following:

- Consensus ethical principles derived from international guidelines including the Declaration of Helsinki and Council for International Organizations of Medical Sciences (CIOMS) International Ethical Guidelines
- Applicable ICH Good Clinical Practice (GCP) Guidelines
- Applicable laws and regulations

The protocol, protocol amendments, ICF, Investigator's Brochure, and other relevant documents (eg, advertisements) must be submitted to an IRB/IEC by the investigator and reviewed and approved by the IRB/IEC before the trial is initiated.

Any amendments to the protocol will require IRB/IEC approval before implementation of changes made to the trial design, except for changes necessary to eliminate an immediate hazard to trial patients.

The investigator will be responsible for the following:

- Providing summaries of the status of the trial to the IRB/IEC annually or more frequently in accordance with the requirements, policies, and procedures established by the IRB/IEC
- Notifying the IRB/IEC of SAEs or other significant safety findings as required by IRB/IEC procedures
- Providing oversight of the conduct of the trial at the site and adherence to requirements of 21 CFR, ICH guidelines, the IRB/IEC, European regulation 536/2014 for clinical studies (if applicable), and all other applicable local regulations

10.1.2. Financial Disclosure

Investigators and sub-investigators will provide Cytokinetics with sufficient, accurate financial information as requested to allow Cytokinetics to submit complete and accurate financial certification or disclosure statements to the appropriate regulatory authorities. Investigators are responsible for providing information on financial interests during the course of the trial and for 1 year after completion of the trial.

10.1.3. Informed Consent Process

The investigator or his/her representative will explain the nature of the trial to the patient or his/her legally authorized representative and answer all questions regarding the trial.

Patients must be informed that their participation is voluntary. Patients must be able to comprehend and be willing to sign a statement of informed consent that meets the requirements

of 21 CFR 50, local regulations, ICH guidelines, Health Insurance Portability and Accountability Act (HIPAA) requirements, where applicable, and the IRB/IEC or trial center.

The medical record must include a statement that informed consent was obtained before any trial-specific activities/procedures were performed and the date the consent was obtained. The authorized person obtaining the informed consent must also sign the ICF.

Patients must be re-consented to the most current version of the ICF(s) during their participation in the trial.

A copy of the ICF(s) must be provided to the patient or the patient's legally authorized representative.

Patients are not required to sign a new ICF if they are retested during the initial screening window.

10.1.4. Data Protection

Patients will be assigned a unique identifier by Cytokinetics. Any patient records or datasets that are transferred to Cytokinetics will contain the identifier only; patient names or any information which would make the patient identifiable will not be transferred.

The patient must be informed that his/her personal trial-related data will be used by Cytokinetics in accordance with local data protection law. The level of disclosure must also be explained to the patient who will be required to give consent for their data to be used as described in the ICF.

The patient must be informed that his/her medical records may be examined by Clinical Quality Assurance auditors or other authorized personnel appointed by Cytokinetics, by appropriate IRB/IEC members, and by inspectors from regulatory authorities.

10.1.5. Committees Structure

The trial organization will include an Executive Committee (EC), Steering Committee (SC) and DMC.

The EC will contribute to trial design, implementation, data analysis, and communication of trial results and will consist of experts external to Cytokinetics who are qualified by their medical and scientific expertise and experience, one of the trial investigators, and a Cytokinetics representative. The responsibilities of the EC will be described in an EC charter.

The SC will contribute to implementation of the trial, data analysis, and communication of trial results. They will be HCM experts external to Cytokinetics and represent the different geographies the trial will be conducted in. The responsibilities of the SC will be described in a SC charter.

An independent DMC will be established for this trial to formally review the accumulating data periodically in order to assess risk to patients during the conduct of the trial. The DMC will include an external cardiologist with relevant expertise and other designated members with relevant expertise, eg, representing clinical science, clinical pharmacology, and biostatistics. The independent DMC membership will exclude the individuals from Cytokinetics or the contract research organization (CRO) trial team involved in trial conduct. The DMC members will have access to treatment assignments and patient level data from the clinical trial database. DMC

membership, responsibilities, relationship with Cytokinetics and the CRO, and the purpose and timing of the meetings will be further described in the DMC charter.

10.1.6. Data Quality Assurance

All patient data relating to the trial will be recorded on printed or electronic CRF unless transmitted to Cytokinetics or designee electronically (eg, laboratory data). The investigator is responsible for verifying that data entries are accurate and correct by physically or electronically signing the CRF.

The investigator must maintain accurate documentation (source data) that supports the information entered in the CRF.

The investigator must permit trial-related monitoring, audits, IRB/IEC review, and regulatory authority inspections and provide direct access to source data documents.

Monitoring details describing strategy (eg, risk-based initiatives in operations and quality such as Risk Management and Mitigation Strategies and Analytical Risk-Based Monitoring), methods, responsibilities and requirements, including handling of non-compliance issues and monitoring techniques (central, remote, or on-site monitoring) are provided in the Monitoring Plan.

Cytokinetics or designee is responsible for the data management of this trial including quality checking of the data.

Cytokinetics assumes accountability for actions delegated to other individuals (eg, CROs).

Trial monitors will perform ongoing source data verification to confirm that data entered into the CRF by authorized site personnel are accurate, complete, and verifiable from source documents; that the safety and rights of patients are being protected; and that the trial is being conducted in accordance with the currently approved protocol and any other trial agreements, ICH GCP, and all applicable regulatory requirements.

Records and documents, including signed ICFs, pertaining to the conduct of this trial must be retained by the investigator for 5 years after trial completion unless local regulations or institutional policies require a longer retention period. No records may be destroyed during the retention period without the approval of Cytokinetics. No records may be transferred to another location or party without notification to Cytokinetics.

10.1.7. Source Documents

Source documents provide evidence for the existence of the patient and substantiate the integrity of the data collected. Source documents are filed at the investigator's site.

Data reported in the CRF or entered in the CRF that are transcribed from source documents must be consistent with the source documents or the discrepancies must be explained. The investigator may need to request previous medical records or transfer records, depending on the trial. Also, current medical records must be available.

10.1.8. Trial and Site Start and Closure

The trial start date is the date on which the clinical trial will be open for recruitment of patients.

The first act of recruitment is the first site activated.

Cytokinetics or designee reserves the right to close the trial site or terminate the trial at any time for any reason at the sole discretion of Cytokinetics. Trial sites will be closed upon trial completion. A trial site is considered closed when all required documents and trial supplies have been collected and a site closure visit has been performed.

The investigator may initiate trial-site closure at any time, provided there is reasonable cause and sufficient notice is given in advance of the intended termination.

Reasons for the early closure of a trial site by Cytokinetics or investigator may include but are not limited to:

- Failure of the investigator to comply with the protocol, the requirements of the IRB/IEC or local regulatory authorities, Cytokinetics's procedures, or GCP guidelines
- Inadequate recruitment of patients by the investigator
- Discontinuation of further IP development

If the trial is prematurely terminated or suspended, Cytokinetics shall promptly inform the investigators, the IECs/IRBs, the regulatory authorities, and any CRO(s) used in the trial of the reason for termination or suspension, as specified by the applicable regulatory requirements. The investigator shall promptly inform the patient and should assure appropriate patient therapy and/or follow-up.

10.2. Appendix 2: Clinical Laboratory Tests

The tests detailed in Table 8 will be performed by the central laboratory. Pregnancy testing for WOCBP at time points after screening may be performed locally.

Local laboratory results are only required if the central laboratory results are not available in time for either IP administration and/or response evaluation. If a local sample is required, it is important that the sample for central analysis is obtained at the same time if feasible. Additionally, if local laboratory results are used for a response evaluation, the results must be entered in the CRF.

Protocol-specific requirements for inclusion or exclusion of patients are detailed in Section 5 of the protocol.

Additional tests may be performed at any time during the trial as determined necessary by the investigator or required by local regulations.

Table 8: Protocol-Required Safety Laboratory Assessments

Chemistry		Urinalysis	Hematology	Other Assessments
Sodium	Total bilirubin	Specific gravity	Hemoglobin	CK-3773274 plasma concentration
Potassium	Direct bilirubin	pH	Hematocrit	
Chloride	CK	Blood	RBC	Pregnancy test[a]
Calcium	ALP	Protein	RDW	FSH[a]
Magnesium	LDH	Glucose	MCV	NT-proBNP[b]
Phosphorus	AST (SGOT)	Bilirubin	MCH	hs-cTnI
Urea	ALT (SGPT)		MCHC	Other biomarkers including: Galectin-3, PINP, PIIINP, TIMP-1, CITP and Soluble ST2
Creatinine	GGT		WBC	
Iron	TIBC		Platelets	
Glucose	Ferritin			
Total protein	Bicarbonate			

ALP = alkaline phosphatase; CK = creatine kinase; FSH = follicle-stimulating hormone; GGT = gamma-glutamyl transferase LDH = lactic acid dehydrogenase; MCH = mean corpuscular hemoglobin; MCHC = mean corpuscular hemoglobin concentration; MCV = mean corpuscular volume; NT-proBNP = N-terminal pro-B-type natriuretic peptide; RBC = red blood cell; RDW = red cell distributions width; SGOT = serum glutamic-oxaloacetic transaminase; SGPT = serum glutamic-pyruvic transaminase; TIBC= total iron binding protein; WBC = white blood cell

[a] A pregnancy test is required for WOCBP; FSH only at screening if needed.

[b] NT-proBNP results will be masked after randomization

Investigators must document their review of each laboratory report.

Laboratory results that could unblind the trial will not be reported to investigative sites or other blinded personnel until the trial has been unblinded.

10.3. Appendix 3: Contraceptive Guidance

Definitions:

Woman of Childbearing Potential

A woman is considered fertile following menarche and until becoming post-menopausal unless permanently sterile (see below).

If fertility is unclear (eg, amenorrhea in adolescents or athletes) and a menstrual cycle cannot be confirmed before first dose of IP, additional evaluation should be considered.

Women in the following categories are not considered WOCBP:

1. Premenarchal
2. Premenopausal female with 1 of the following:
   - Documented hysterectomy
   - Documented bilateral salpingectomy
   - Documented bilateral oophorectomy

   For individuals with permanent infertility due to an alternate medical cause other than the above (eg, Mullerian agenesis, androgen insensitivity), investigator discretion should be applied to determining trial entry.

   Note: Documentation can come from the site personnel's review of the patient's medical records, medical examination, or medical history interview.
3. Postmenopausal female
   - A postmenopausal state is defined as no menses for 12 months without an alternative medical cause.
     - A high follicle stimulating hormone (FSH) level in the postmenopausal range may be used to confirm a postmenopausal state in women not using hormonal contraception or hormone replacement therapy. However, in the absence of 12 months of amenorrhea, confirmation with more than one FSH measurement (>40 IU/L or mIU/mL) is required.
   - Females on hormone replacement therapy and whose menopausal status is in doubt will be required to use one of the non-estrogen hormonal highly effective contraception methods if they wish to continue their hormone replacement therapy during the trial. Otherwise, they must discontinue hormone replacement therapy to allow confirmation of postmenopausal status before trial enrollment.

Highly Effective Method of Contraception

A highly effective method of contraception is one that has a failure rate of <1% per year when used consistently and correctly.

Examples of highly effective contraception that have low user dependency are:

- Implantable progestogen-only hormone contraception associated with inhibition of ovulation
- Intrauterine device (IUD)
- Intrauterine hormone-releasing system (IUS)
- Bilateral tubal occlusion
- Vasectomized partner, only when the absence of sperm has been confirmed and vasectomized partner is the sole sexual partner of the female patient

Examples of highly effective contraception that are user-dependent are:

- Combined hormonal methods of birth control include oral, intravaginal, transdermal, injectable, or implantable
- Oral or injectable progestogen-only hormone contraception associated with the inhibition of ovulation
- Sexual abstinence

  Note: Sexual abstinence is considered a highly effective method only if defined as refraining from heterosexual intercourse during the entire period of risk associated with the trial intervention. The reliability of sexual abstinence needs to be evaluated in relation to the duration of the trial and the preferred and usual lifestyle of the patient. Periodic abstinence (calendar, symptothermal, post-ovulation methods), withdrawal (coitus interruptus), spermicides only, and lactational amenorrhoea method (LAM) are not acceptable methods of contraception.

Contraception Guidance:

Women of Childbearing Potential

WOCBP must use at least one highly effective method of birth control. If hormonal contraception is used as a highly effective method of birth control, a male condom must also be used. Male condom and female condom should not be used together (due to risk of failure with friction).

If additional medications are given during treatment, the investigator is to review the prescribing information/summary of product characteristics for all concomitant therapy, as they may alter the contraceptive requirements. These additional medications may require an increase in the number of contraceptive methods and/or length of time that contraception is to be utilized after the last dose of protocol-required therapies. The investigator is to discuss these changes with the trial patient.

10.4. Appendix 4: Genetics

Use/Analysis of DNA

Genetic variation may impact a patient's response to IP, susceptibility to, and severity and progression of disease. Variable response to IP may be due to genetic determinants that impact drug absorption, distribution, metabolism, and excretion; mechanism of action of the drug; disease etiology; and/or molecular subtype of the disease being treated. Therefore, where local regulations and IRB/IEC allow, a blood sample will be collected for DNA analysis from patients consenting to provide the sample.

DNA samples will be used for research related to this trial may consist of the analysis of one or more candidate genes or the analysis of genetic markers throughout the genome (as appropriate).

The results of genetic analyses may be reported in the clinical study report or in a separate trial summary.

Cytokinetics will store the DNA samples in a secure storage space with adequate measures to protect confidentiality.

10.5. Appendix 5: Liver Safety: Actions and Follow-up Assessments

Drug-induced Liver Injury Reporting & Additional Assessments

To facilitate appropriate monitoring for signals of DILI, cases of concurrent aspartate aminotransferase (AST) or alanine aminotransferase (ALT) and total bilirubin (TBL) and/or international normalized ratio (INR) elevation require the following:

- The event is to be reported to Cytokinetics as an SAE within 24 hours of discovery or notification of the event (ie, before additional etiologic investigations have been concluded)
- The AE CRF that captures information necessary to facilitate the evaluation of treatment-emergent liver abnormalities is to be completed and sent to Cytokinetics.

Other events of hepatotoxicity and potential DILI are to be reported as SAEs if they meet the criteria for an SAE defined in Section 8.5.1.2.

Criteria for Permanent Discontinuation of Investigational Product and Other Protocol-required Therapies due to Potential Hepatotoxicity

CK-3773274 **must** be discontinued permanently and the patient should be followed according to the following recommendations for possible DILI, if ALL of the criteria below are met:

- TBL >2 × ULN or INR >1.5

AND

- increased AST or ALT from the relevant baseline value as specified below:

Baseline AST or ALT Value	AST or ALT Elevation
<ULN	>3 × ULN

AND

- no other cause for the combination of the above laboratory abnormalities is apparent; important alternative causes for elevated AST/ALT and TBL values include, but are not limited to:
  - hepatobiliary tract disease
  - viral hepatitis (eg, Hepatitis A/B/C/D/E, Epstein-Barr Virus, cytomegalovirus, Herpes Simplex Virus, Varicella, toxoplasmosis, and Parvovirus)
  - right sided heart failure, hypotension, or any cause of hypoxia to the liver causing ischemia
  - exposure to hepatotoxic agents/drugs or hepatotoxins, including herbal and dietary supplements, plants and mushrooms
  - heritable disorders causing impaired glucuronidation (eg, Gilbert's Syndrome, Crigler-Najjar syndrome) and drugs that inhibit bilirubin glucuronidation (eg, indinavir, atazanavir)

- alpha-one antitrypsin deficiency
- alcoholic hepatitis
- autoimmune hepatitis
- Wilson's disease and hemochromatosis
- nonalcoholic fatty liver disease including steatohepatitis
- nonhepatic causes (eg, rhabdomyolysis, hemolysis)

Criteria for Conditional Interruption of Investigational Product and Other Protocol-required Therapies due to Potential Hepatotoxicity

For patients who do not meet the criteria for permanent discontinuation of IP outlined above and have no underlying liver disease, the following rules are recommended for interruption of IP and other protocol required therapies:

- Elevation of either AST or ALT according to the following schedule:

Baseline AST or ALT Value	AST or ALT Elevation
Any	>8 × ULN at any time
Any	>5 × ULN but <8 × ULN for ≥2 weeks
Any	>5 × ULN but <8 × ULN and unable to adhere to enhanced monitoring schedule
Any	>3 × ULN with clinical signs or symptoms that are consistent with hepatitis (such as right upper quadrant pain/tenderness, fever, nausea, vomiting, jaundice)

OR

- TBL >3 × ULN at any time

IP and other protocol-required therapies, as appropriate must be withheld pending investigation into alternative causes of DILI. If IP is withheld, the patient is to be followed according to recommendations in this section for possible DILI. Rechallenge may be considered if an alternative cause for impaired liver tests (ALT, AST, ALP) and/or elevated TBL, is discovered and the laboratory abnormalities resolve to normal or baseline.

Rechallenge of Investigational Product and Other Protocol-required Therapies After Potential Hepatotoxicity

The decision to rechallenge the patient must be discussed and agreed upon unanimously by the patient, investigator, and Medical Monitor. Patients reinitiating IP after withholding for potential hepatotoxicity will restart IP, according to initial randomized allocation, on the same IP dose as established before the event and will not further titrate the dose.

If signs or symptoms recur with rechallenge, then IP must be permanently discontinued. Patients who clearly meet the criteria for permanent discontinuation must never be rechallenged.

Additional Clinical Assessments and Observation

All patients in whom IP(s) or protocol-required therapies is/are withheld (either permanently or conditionally) due to potential DILI or who experience AST or ALT elevations >3 × ULN are to undergo a repeat test and a period of "close observation" until abnormalities have stabilized, returned to normal, or returned to the patient's baseline levels. Recommended assessments and testing frequency that are to be performed during this period include:

- Repeat AST, ALT, ALP, bilirubin (total and direct), and INR within 48 hours of receiving results with repeat testing until stabilized
- In cases of TBL >2 × ULN or INR >1.5, retesting of liver tests, bilirubin (total and direct), and INR should be performed within 48 hours of receiving results with repeat testing 2-3 times per week until stabilized

Testing frequency of the above laboratory tests may decrease if the abnormalities stabilize or the IP(s) or protocol-required therapies has/have been discontinued AND the patient is asymptomatic.

- Initiate investigation of alternative causes for elevated AST or ALT and/or elevated TBL:
  - Obtain complete blood count (CBC) with differential to assess for eosinophilia
  - Obtain serum total immunoglobulin IgG, Anti-nuclear antibody (ANA), Anti Smooth Muscle Antibody, and Liver Kidney Microsomal antibody 1 (LKM1) to assess for autoimmune hepatitis
  - Obtain serum acetaminophen (paracetamol) levels
  - Obtain a more detailed history of:
    - Prior and/or concurrent diseases or illness
    - Exposure to environmental and/or industrial chemical agents
    - Symptoms (if applicable) including right upper quadrant pain, hypersensitivity type reactions, fatigue, nausea, vomiting and fever
    - Prior and/or concurrent use of alcohol, recreational drugs and special diets
    - Concomitant use of medications (including non-prescription medicines and herbal and dietary supplements), plants, and mushrooms
  - Obtain viral serologies
  - Obtain creatine phosphokinase, haptoglobin, lactate dehydrogenase, and peripheral blood smear
  - Perform appropriate liver imaging if clinically indicated
- Obtain appropriate blood sampling for PK analysis if this has not already been collected
- Obtain hepatology consult (liver biopsy may be considered in consultation with an hepatologist)

Follow the patient and the laboratory tests (ALT, AST, TBL, INR) until all laboratory abnormalities return to baseline or normal. The "close observation period" is to continue for a minimum of 4 weeks after discontinuation of all IP(s) and protocol required therapies.

The potential DILI event and additional information such as medical history, concomitant medications, and laboratory results must be captured in corresponding CRFs.

10.6. Appendix 6: Abbreviations

Table 9: List of Abbreviations

Abbreviation/Term	Explanation
AE	Adverse event
ALP	Alkaline phosphatase
ALT	Alanine aminotransferase
ANA	Anti-nuclear antibody
AST	Aspartate aminotransferase
CBC	Complete blood count
CGI	Clinical Global Impression scale
CI	Confidence interval
CIOMS	Council for International Organizations of Medical Sciences
C_{max}	Maximum plasma concentration observed
C_{trough}	Trough plasma concentration observed
CMH	Cochran–Mantel–Haenszel
CMR	Cardiac magnetic resonance
CPET	Cardiopulmonary exercise testing
CRF	Case report form
CRO	Contract research organization
CV	Cardiovascular
CYP	Cytochrome P450
DILI	Drug induced liver injury
DMC	Data monitoring committee
EC	Executive Committee
ECG	Electrocardiogra(m/phy)
ED	Early discontinuation
EOS	End of study
EOT	End of treatment
EQ-5D-5L	EuroQol 5-dimension 5-level instrument
FAS	Full analysis set
FSH	Follicle-stimulating hormone
FU	Follow up
GCP	Good Clinical Practice

Abbreviation/Term	Explanation
GLP	Good Laboratory Practice
HCM	Hypertrophic cardiomyopathy
HIPAA	Health Insurance Portability and Accountability Act
hs-cTnI	High sensitivity cardiac troponin I
IB	Investigator’s Brochure
ICD	Implantable cardioverter defibrillators
ICF	Informed consent form
ICH	International Council for Harmonisation
IEC	Independent ethics committee
IgG	Immunoglobulin G
IMP	Investigational medicinal product
INR	International normalized ratio
IP	Investigational product
IRB	Institutional review board
IUD	Intrauterine device
IUS	Intrauterine hormone-releasing system
IWRS	Interactive web response system
KCCQ	Kansas City Cardiomyopathy Questionnaire
LAM	Lactational amenorrhoea method
LKM1	Liver Kidney Microsomal antibody 1
LV	Left ventricle(ular)
LVEDV	Left ventricular end-diastolic volume
LVEF	Left ventricular ejection fraction
LVESV	Left ventricular end-systolic volume
LVFS	Left ventricular fractional shortening
LVOT	Left ventricular outflow tract
LVOT-G	Left ventricular outflow tract gradient
MedDRA	Medical Dictionary for Regulatory Activities
NIMP	Non-investigational medicinal product
NT-proBNP	n-terminal prohormone brain natriuretic peptide
NYHA	New York Heart Association
oHCM	Obstructive hypertrophic cardiomyopathy

Abbreviation/Term	Explanation
PD	Pharmacodynamics
PDS	Pharmacodynamics analysis set
PGI-C	Patient Global Impression of Change scale
PK	Pharmacokinetics
PKS	Pharmacokinetics analysis set
PRO	Patient-reported outcomes
pVO_2	Peak oxygen uptake
RBC	Red blood cell
RER	Respiratory exchange ratio
SAE	Serious adverse event
SAS	Safety analysis set
SAQ-7	Seattle Angina Questionnaire -7
SC	Steering Committee
SD	Standard deviation
SoA	Schedule of activities
SoC	Standard of care
TBL	Total bilirubin
ULN	Upper limit of normal
VAT	Ventilatory anaerobic threshold
WOCBP	Women of childbearing potential

11. REFERENCES

Chuan, P., Sivaramakrishnan, S., Ashley, E. A. and Spudich, J. A. (2012). "Cell-intrinsic functional effects of the α-cardiac myosin arg-403-gln mutation in familial hypertrophic cardiomyopathy." Biophysical journal 102(12): 2782-2790.

Criteria Committee of the New York Heart, A. (1994). Nomenclature and criteria for diagnosis of diseases of the heart and great vessels. Boston: Little, Brown & Co.

Elliott, P. M., Anastasakis, A., Borger, M. A., Borggrefe, M., Cecchi, F., Charron, P., et al. (2014). "2014 esc guidelines on diagnosis and management of hypertrophic cardiomyopathy: The task force for the diagnosis and management of hypertrophic cardiomyopathy of the european society of cardiology (esc)." European heart journal 35(39): 2733-2779.

Gersh, B. J., Maron, B. J., Bonow, R. O., Dearani, J. A., Fifer, M. A., Link, M. S., et al. (2011). "2011 accf/aha guideline for the diagnosis and treatment of hypertrophic cardiomyopathy." Journal of the American College of Cardiology 58(25): e212-e260.

Husser, D., Ueberham, L., Jacob, J., Heuer, D., Riedel-Heller, S., Walker, J., et al. (2018). "Prevalence of clinically apparent hypertrophic cardiomyopathy in germany-an analysis of over 5 million patients." PloS one 13(5): e0196612-e0196612.

Kristensen, S. D., Knuuti, J., Saraste, A., Anker, S., BÃ¸tker, H. E., Hert, S. D., et al. (2014). "2014 esc/esa guidelines on non-cardiac surgery: Cardiovascular assessment and management: The joint task force on non-cardiac surgery: Cardiovascular assessment and management of the european society of cardiology (esc) and the european society of anaesthesiology (esa)." European heart journal 35(35): 2383-2431.

Magnusson, P., Palm, A., Branden, E. and Morner, S. (2017). "Misclassification of hypertrophic cardiomyopathy: Validation of diagnostic codes." Clinical epidemiology 9: 403-410.

Marian, A. J. and Braunwald, E. (2017). "Hypertrophic cardiomyopathy: Genetics, pathogenesis, clinical manifestations, diagnosis, and therapy." Circulation research 121(7): 749-770.

Maron, B. J. (2018). "Clinical course and management of hypertrophic cardiomyopathy." New England Journal of Medicine 379(20): 655-668.

Maron, M. S., Iacopo, O., Andrey, G. Z., Mark, S. L., Natesa, G. P., Jeffery, T. K., et al. (2006). "Hypertrophic cardiomyopathy is predominantly a disease of left ventricular outflow tract obstruction." Circulation 114(21): 2232-2239.

Maron, M. S., Rowin, E. J., Olivotto, I., Casey, S. A., Arretini, A., Tomberli, B., et al. (2016). "Contemporary natural history and management of nonobstructive hypertrophic cardiomyopathy." Journal of the American College of Cardiology 67(12): 1399-1409.

Ommen, S. R., Mital, S., Burke, M. A., Day, S. M., Deswal, A., Elliott, P., et al. (2020). "2020 aha/acc guideline for the diagnosis and treatment of patients with hypertrophic cardiomyopathy." Circulation 142(25): e558-e631.

Ponikowski, P., Voors, A. A., Anker, S. D., Bueno, H., Cleland, J. G. F., Coats, A. J. S., et al. (2016). "2016 esc guidelines for the diagnosis and treatment of acute and chronic heart failure: The task force for the diagnosis and treatment of acute and chronic heart failure of the european society of cardiology (esc)developed with the special contribution of the heart failure association (hfa) of the esc." European heart journal 37(27): 2129-2200.

Pujades-Rodriguez, M., Guttmann, O. P., Gonzalez-Izquierdo, A., Duyx, B., O'Mahony, C., Elliott, P., et al. (2018). "Identifying unmet clinical need in hypertrophic cardiomyopathy using national electronic health records." PloS one 13(1): e0191214-e0191214.

Sommese, R. F., Sung, J., Nag, S., Sutton, S., Deacon, J. C., Choe, E., et al. (2013). "Molecular consequences of the r453c hypertrophic cardiomyopathy mutation on human β-cardiac myosin motor function." Proceedings of the National Academy of Sciences 110(31): 12607-12612.

Spudich, J. A., Aksel, T., Bartholomew, S. R., Nag, S., Kawana, M., Yu, E. C., et al. (2016). "Effects of hypertrophic and dilated cardiomyopathy mutations on power output by human β-cardiac myosin." The Journal of Experimental Biology 219(2): 161-167.

Toepfer, C. N., Wakimoto, H., Garfinkel, A. C., McDonough, B., Liao, D., Jiang, J., et al. (2019). "Hypertrophic cardiomyopathy mutations in mybpc3 dysregulate myosin." Science Translational Medicine 11(476): eaat1199.

Wilson, W. S., Criley, J. M. and Ross, R. S. (1967). "Dynamics of left ventricular emptying in hypertrophic subaortic stenosis: A cineangiographic and hemodynamic study." American Heart Journal 73(1): 4-16.

Final version of

the protocol

PROTOCOL CY 6031

A PHASE 3, MULTI-CENTER, RANDOMIZED, DOUBLE-BLIND, PLACEBO-CONTROLLED TRIAL TO EVALUATE THE EFFICACY AND SAFETY OF CK-3773274 IN ADULTS WITH SYMPTOMATIC HYPERTROPHIC CARDIOMYOPATHY AND LEFT VENTRICULAR OUTFLOW TRACT OBSTRUCTION

**Protocol Version and Date:**	Amendment 04 dated 08 December 2023
**Previous Version(s):**	Amendment 03 dated 03 January 2023<br>Amendment 02 dated 10 December 2021<br>Amendment 01 dated 17 August 2021<br>Original Protocol 26 July 2021
**Product:**	CK-3773274 (aficamten)
**Regulatory Authority Identifier Number(s):**	IND 138814<br>EudraCT Number 2021-003536-92
**Sponsor:**	Cytokinetics, Inc.<br>350 Oyster Point Blvd.<br>South San Francisco, CA 94080, USA

This document contains confidential information, which should not be copied, referred to, released or published without documented approval from Cytokinetics, Inc.

INVESTIGATOR SIGNATURE PAGE

Protocol Number:	CY 6031
Protocol Title:	A Phase 3, Multi-Center, Randomized, Double-blind, Placebo-controlled Trial to Evaluate the Efficacy and Safety of CK-3773274 in Adults with Symptomatic Hypertrophic Cardiomyopathy and Left Ventricular Outflow Tract Obstruction
Protocol Version and Date:	Amendment 04 dated 08 December 2023

Principal Investigator Commitment

I, the undersigned Principal Investigator, submit this statement of commitment as evidence that I understand my responsibilities pursuant to the Code of Federal Regulations (21 CFR § 312) and International Council for Harmonisation (ICH) E6(R2) Good Clinical Practice (GCP) guidelines, as well as with any and all applicable federal, state and/or local laws and regulations, and agree to conduct this trial in accordance with the protocol referenced herein.

Investigator Name: ______________________ Date: ______________

Investigator Signature: ______________________________

PROTOCOL APPROVAL PAGE

Protocol Number:	CY 6031
Protocol Title:	A Phase 3, Multi-Center, Randomized, Double-blind, Placebo-controlled Trial to Evaluate the Efficacy and Safety of CK-3773274 in Adults with Symptomatic Hypertrophic Cardiomyopathy and Left Ventricular Outflow Tract Obstruction
Protocol Version and Date:	Amendment 04 dated 08 December 2023
Sponsor:	Cytokinetics, Inc.<br>350 Oyster Point Blvd.<br>South San Francisco, CA 94080

DocuSigned by:

Signer Name: Daniel Jacoby
Signing Reason: I approve this document
Signing Time: Dec-11-2023 | 15:31:15 PST
6897282076D24D538A113264D87AE5FC

Dec-11-2023 | 15:31:18 PST

Daniel Jacoby, MD
Senior Medical Director, Clinical Research, Cardiovascular

Date:

PROTOCOL AMENDMENT SUMMARY OF CHANGES

DOCUMENT HISTORY	
**Document**	**Date**
Amendment 04	08 December 2023
Amendment 03	03 January 2023
Amendment 02	10 December 2021
Amendment 01	17 August 2021
Original Protocol	26 July 2021

Amendment 04 (08 December 2023)

The main purpose of this amendment is as follows:

- To add a safety endpoint that contextualizes observations of LVEF <50% with clinically relevant associated findings.
- To update the definition of full analysis set.
- To update the testing hierarchy to use a closed testing procedure with pre-specified testing order to test the secondary endpoints once the primary endpoint achieves statistical significance.

Table 1: Protocol Amendment 04 Summary of Changes

Section # and Name	Description of Change	Brief Rationale
1.1 Synopsis<br>3. Objectives and Endpoints	Added the safety endpoint of incidence of LVEF <50% with signs and symptoms of heart failure (concomitant adverse event of heart failure or dyspnea) and/or increase in NT-proBNP from baseline	This additional safety endpoint contextualizes an observation of LVEF <50% with clinically relevant associated findings
1.1 Synopsis<br>9.3 Populations For Analyses	Updated full analysis set definition to include all randomized patients	Per FDA's recommendation

Table 1: Protocol Amendment 04 Summary of Changes (Continued)

Section # and Name	Description of Change	Brief Rationale
1.1 Synopsis<br>9.4.1.1 Multiplicity Adjustment	Revised testing hierarchy; and made editorial updates	The testing hierarchy was simplified from a parallel gatekeeping method to a closed testing procedure. This adjustment allows for the examination of secondary endpoints at two-sided alpha level of 0.05 once the primary endpoint achieves statistical significance at the prespecified sequential testing order

TABLE OF CONTENTS

INVESTIGATOR SIGNATURE PAGE 2
PROTOCOL APPROVAL PAGE 3
PROTOCOL AMENDMENT SUMMARY OF CHANGES 4
TABLE OF CONTENTS 6
LIST OF TABLES 10
LIST OF FIGURES 10
1. PROTOCOL SUMMARY 11
1.1. Synopsis 11
1.2. Schema 19
1.3. Schedule of Activities 20
1.4. Key Contacts 23
2. INTRODUCTION 24
2.1. Trial Rationale 24
2.2. Background 24
2.2.1. Hypertrophic Cardiomyopathy 24
2.2.2. CK-3773274 25
2.3. Benefit/Risk Assessment 25
2.3.1. Risk Assessment 25
2.3.1.1. Mitigation Strategy 26
2.3.2. CK-3773274 Benefit Assessment 27
3. OBJECTIVES AND ENDPOINTS 28
4. TRIAL DESIGN 31
4.1. Overall Design 31
4.1.1. Number of Sites 31
4.1.2. Number of Patients 31
4.1.3. Replacement of Patients 31
4.1.4. Trial Duration 31
4.1.5. CMR Imaging Sub-Study 32
4.2. Scientific Rationale for Trial Design 32
4.3. Justification for Dose 32
4.4. End of Study Definition 32

5. STUDY POPULATION 33
5.1. Inclusion Criteria 33
5.2. Exclusion Criteria 34
5.3. Lifestyle Considerations 36
5.4. Screen Failures....36
6. INVESTIGATIONAL PRODUCT 38
6.1. Investigational Product(s) Administered 38
6.2. Preparation/Handling/Storage/Accountability....38
6.3. Measures to Minimize Bias: Randomization and Blinding....39
6.4. Investigational Product Compliance....40
6.5. Concomitant Therapy 40
6.5.1. Drug-Drug Interactions....40
6.5.2. Rescue Medicine....41
6.6. Dose Modifications....41
6.6.1. Scheduled Dose Titrations....41
6.6.1.1. Week 2 Visit 41
6.6.1.2. Week 4 Visit 41
6.6.1.3. Week 6 Visit 42
6.6.1.4. Week 8 Visit 42
6.6.2. Dose Reductions 42
6.6.3. LVEF Safety Threshold....42
6.6.4. Hepatotoxicity Stopping and Rechallenge Rules 43
6.7. Access to Investigational Product after the End of the Study 43
7. TEMPORARY INTERRUPTION OF INVESTIGATIONAL PRODUCT, DISCONTINUATION OF INVESTIGATIONAL PRODUCT, AND PATIENT CONSENT WITHDRAWAL 44
7.1. Temporary IP Interruption....44
7.2. Permanent Discontinuation of IP....44
7.2.1. Management of Patients after Permanent Discontinuation of IP....45
7.3. Discontinuation from Trial Procedures 45
7.4. Patient Consent Withdrawal 46
7.5. Lost to Follow up....46
8. TRIAL ASSESSMENTS AND PROCEDURES 47

8.1. Visit Schedule 47
8.1.1. Screening Visit....48
8.1.2. Day 1....48
8.1.3. Weeks 2 through 20 48
8.1.4. Week 24: End of Treatment Visit....48
8.1.5. Week 28: End of Study Visit....49
8.1.6. Early Discontinuation Visit 49
8.1.7. Unscheduled Visit....49
8.2. Efficacy Assessments 49
8.2.1. Cardiopulmonary Exercise Testing 49
8.2.2. Echocardiography....50
8.2.3. Cardiac Magnetic Resonance 51
8.2.4. New York Heart Association Functional Classification....51
8.2.5. Clinical Global Impression Scale 52
8.2.6. Patient-Reported Outcomes....52
8.3. Safety Assessments....52
8.3.1. Physical Examinations....52
8.3.2. Height and Weight....52
8.3.3. Vital Signs 53
8.3.4. Electrocardiograms....53
8.3.5. Laboratory Assessments....53
8.4. Adverse Events and Serious Adverse Events....54
8.4.1. Adverse Events....54
8.4.1.1. Definition of Adverse Event....54
8.4.1.2. Definition of Serious Adverse Event....54
8.4.1.3. Intensity of Adverse Events....55
8.4.1.4. Relationship to Investigational Product....56
8.4.1.5. Relationship to Trial Procedures 56
8.4.1.6. Reporting of AEs....56
8.4.1.7. Reporting Procedures for SAEs....57
8.4.1.8. Follow-up of AEs and SAEs....57
8.4.1.9. Regulatory Reporting....58
8.4.1.10. Pregnancy and Breastfeeding 58

8.5. Treatment of Overdose 59
8.6. Pharmacokinetics 60
8.7. Genetics 60
8.8. Serum for Biomarker Analysis 61
8.9. Serum and Plasma Collection for Future Analyses 61
8.10. Immunogenicity Assessments 61
9. STATISTICAL CONSIDERATIONS 62
9.1. Statistical Hypotheses 62
9.2. Sample Size Determination 62
9.3. Populations for Analyses 62
9.4. Statistical Analyses 63
9.4.1. General Considerations 63
9.4.1.1. Multiplicity Adjustment 63
9.4.2. Primary Endpoint(s) 63
9.4.3. Secondary Endpoint(s) 64
9.4.4. Exploratory Endpoint(s) 65
9.4.5. Safety Analysis 66
9.4.5.1. Adverse Events 66
9.4.5.2. Serious Adverse Events 66
9.4.6. Pharmacokinetic Endpoints 67
9.4.7. Patient Disposition 67
9.4.8. Demographics and Other Baseline Characteristics 67
9.4.9. Investigational Product Exposure 67
9.4.10. Concomitant Medications 67
9.4.11. Clinical Laboratory Parameters 67
9.4.12. Vital Signs 67
9.4.13. Electrocardiogram 67
9.5. Data Monitoring Committee 68
10. SUPPORTING DOCUMENTATION AND OPERATIONAL CONSIDERATIONS 69
10.1. Appendix 1: Regulatory, Ethical, and Trial Oversight Considerations 69
10.1.1. Regulatory and Ethical Considerations 69
10.1.2. Financial Disclosure 69

10.1.3. Informed Consent Process 69
10.1.4. Data Protection 70
10.1.5. Committees Structure 70
10.1.6. Data Quality Assurance 71
10.1.7. Source Documents 71
10.1.8. Trial and Site Start and Closure 71
10.2. Appendix 2: Clinical Laboratory Tests 73
10.3. Appendix 3: Contraceptive Guidance 74
10.4. Appendix 4: Genetics 76
10.5. Appendix 5: Liver Safety: Actions and Follow-up Assessments 77
10.6. Appendix 6: Abbreviations 81
10.7. Appendix 7: Protocol Amendment History 84
11. REFERENCES 85

LIST OF TABLES

Table 1: Protocol Amendment 04 Summary of Changes 4
Table 2: Trial Objectives and Endpoints 28
Table 3: Investigational Products 38
Table 4: Echocardiogram Criteria for Scheduled Dose Titrations 42
Table 5: CY 6031 Visit Windows 47
Table 6: CY 6031 Echocardiographic LV Parameters to be Measured 51
Table 7: Summary of PK Time Points 60
Table 8: Analysis Sets 62
Table 9: Protocol-Required Safety Laboratory Assessments 73
Table 10: List of Abbreviations 81

LIST OF FIGURES

Figure 1: Statistical Testing Hierarchy for Trial Endpoints 18
Figure 2: Trial Schema 19

1. PROTOCOL SUMMARY

1.1. Synopsis

**Name of Investigational Product(s) (IP):** CK-3773274
**Name of Active Ingredient(s):** CK-3773274
**Protocol Title:**<br>A Phase 3, Multi-Center, Randomized, Double-blind, Placebo-controlled Trial to Evaluate the Efficacy and Safety of CK-3773274 in Adults with Symptomatic Hypertrophic Cardiomyopathy and Left Ventricular Outflow Tract Obstruction
**Phase of Development:** Phase 3
**Rationale:**<br>Hypertrophic cardiomyopathy (HCM) is a disease of the cardiac sarcomere for which the fundamental pathophysiologic abnormality is myocardial hypercontractility leading to cardiac hypertrophy. In patients with obstructive HCM (oHCM), dynamic left ventricular outflow tract (LVOT) obstruction creates a high-pressure outflow tract gradient during systole. Patients with oHCM often develop signs and symptoms of heart failure.<br>CK-3773274 is a small molecule cardiac myosin inhibitor being developed as a chronic, oral treatment for patients with HCM. CK-3773274 is designed to reduce the hypercontractility that underlies the pathophysiology of HCM. Selective inhibition of cardiac myosin with CK-3773274 may yield potential advantages over current therapies for oHCM by directly reducing myocardial hypercontractility and addressing the fundamental cause of this sarcomeric disease.<br>In the Phase 2 trial, CY 6021 (REDWOOD-HCM), patients with oHCM received up to three doses of CK-3773274 or placebo (randomized 2:1) in a dose escalating manner using echocardiography to guide dose titration. Two cohorts of approximately 20 patients each were enrolled and treated for 10 weeks. Doses in the first cohort were 5, 10, 15 mg once daily; the second cohort studied 10, 20, and 30 mg once daily. In both cohorts, CK-3773274 significantly and substantially reduced the LVOT gradient (LVOT-G) in a dose and exposure dependent manner. A third cohort of 13 patients with symptomatic oHCM taking standard of care therapy plus disopyramide were enrolled and treated for 10 weeks. Doses were 5, 10, 15mg once daily. CK3773274 again significantly substantially reduced LVOT-G in a dose and exposure dependent manner. There were no treatment interruptions or discontinuations, nor any treatment related serious adverse events. The results from this trial support progression of CK-3773274 to Phase 3 given the association between reductions in LVOT-G and improvements in patient symptoms and function.<br>This trial will evaluate the effects of treatment with CK-3773274 over a 24-week period on cardiopulmonary exercise capacity and health status in patients with symptomatic oHCM.
**Objectives and Endpoints:**

Objectives	***Endpoint(s)***
Primary	
To evaluate the effect of CK-3773274 on exercise capacity in patients with symptomatic oHCM	• Change in peak oxygen uptake (pVO_2) by cardiopulmonary exercise testing (CPET) from baseline to Week 24

Secondary	
To evaluate the effect of CK-3773274 on patient health status	• Change in Kansas City Cardiomyopathy Questionnaire – Clinical Summary Score (KCCQ-CSS) from baseline to Week 12 and Week 24
To evaluate the effect of CK-3773274 on New York Heart Association (NYHA) Functional Classification	• Proportion of patients with ≥1 class improvement in NYHA Functional Class from baseline to Week 12 and Week 24
To evaluate the effect of CK-3773274 on post-Valsalva left ventricular outflow tract gradients (LVOT-G)	• Change in post-Valsalva LVOT-G from baseline to Week 12 and Week 24<br>• Proportion of patients with post-Valsalva LVOT-G <30 mmHg at Week 12 and Week 24
To evaluate the effect of CK-3773274 on duration of eligibility for septal reduction therapy	• Duration of eligibility for septal reduction therapy (SRT) during the 24-week treatment period in patients who were eligible for SRT at baseline.
To evaluate the effect of CK-3773274 on exercise capacity	• Change in total workload during CPET from baseline to Week 24
Safety	
To evaluate the safety and tolerability profile of CK-3773274 in patients with symptomatic oHCM	• Incidence of reported major adverse cardiac events (cardiovascular [CV] death, cardiac arrest, non-fatal stroke, non-fatal myocardial infarction, CV hospitalization)<br>• Incidence of new onset persistent atrial fibrillation<br>• Incidence of appropriate implantable cardiac defibrillator (ICD) discharges and aborted sudden cardiac death<br>• Incidence of left ventricular ejection fraction (LVEF) <50%<br>• Incidence of LVEF <50% with at least one of the following:<br>o Signs and symptoms of heart failure (concomitant adverse event of heart failure or dyspnea), AND/OR<br>o Increase in NT-proBNP (≥30% increase), relative to results from the most recent previous visit and above the upper limit of normal, at the time of LVEF assessment <50%<br>• Incidence of treatment emergent adverse events

**Exploratory**	
To evaluate the effect of CK-3773274 on exercise capacity and functional class	• Compared with baseline, proportion of patients at Week 24 achieving either:<br>– Change from baseline of ≥1.5 mL/kg/min in pVO_2 AND ≥1 class improvement in NYHA Functional Class<br>**OR**<br>– Change from baseline of ≥3.0 mL/kg/min in pVO_2 AND no worsening of NYHA Functional Class
To evaluate the effect of CK-3773274 on patient response over time	• Proportion of patients with improvement in KCCQ-CSS ≥5 points at Weeks 12 and 24<br>• Proportion of patients with resting LVOT-G <30 mmHg, post-Valsalva LVOT-G <50 mmHg, and NYHA Functional Class I at Weeks 12 and 24<br>• Proportion of patients with resting LVOT-G <30 mmHg, post-Valsalva LVOT-G <50 mmHg, and ≥1 class improvement in NYHA Functional Class at Weeks 12 and 24
To evaluate the effect of CK-3773274 on septal reduction therapy eligibility	• Proportion of patients who are eligible for septal reduction therapy at Week 24 among patients who were eligible for septal reduction therapy at baseline
To evaluate the effect of CK-3773274 on other CPET parameters	Change from baseline to Week 24 in:<br>• Ventilatory efficiency (VE/VCO_2 slope)<br>• Circulatory power (VO_2 × systolic BP)<br>• Ventilatory anaerobic threshold (VAT)
To evaluate the effect of CK-3773274 on health status and health-related quality of life as measured by PRO questionnaire	• Change from baseline to Week 24 in individual responses to the EuroQol 5-dimension 5-level instrument (EQ-5D-5L)
To evaluate the effect of CK-3773274 on health status and quality of life related to chest pain-like angina	• Change from baseline to Week 24 in summary and domain scores for the Seattle Angina Questionnaire-7 (SAQ-7)
To evaluate the effect of CK-3773274 on cardiac function and structure	• Change from baseline to Week 24 in echocardiographic measurements of cardiac structure and of systolic function including:<br>– LVEF<br>– Left ventricular end-systolic and end-diastolic volumes (LVESV and LVEDV, respectively)<br>– Left atrial volume
To evaluate the effect of CK-3773274 on biomarker levels	• Change from baseline values in NT-pro-BNP, hs-cardiac-TnI and other biomarkers through Week 24
To evaluate the effect of CK-3773274 on left ventricular mass, function, and	• Change from baseline to Week 24 in CMR measurements of:

structure by cardiac magnetic resonance (CMR) imaging	– Left ventricular (LV) mass index<br>– LVEF<br>– Septal, free wall, and maximal wall thickness<br>– Left atrial volume index<br>– LVESV<br>– LVEDV
To assess the pharmacokinetics of CK-3773274 and its metabolites	• Pharmacokinetic parameters through Week 24

Overall Design:

This is a Phase 3 randomized, placebo-controlled, double-blind, multi-center trial in patients with symptomatic oHCM. Approximately 270 eligible patients will be randomized in a 1:1 ratio to receive CK-3773274 or placebo. Doses of 5, 10, 15, or 20 mg or matching placebo will be administered in an escalating manner using echocardiography to guide dose titration. Randomization will be stratified by use of beta-blockers and CPET exercise modality.

The trial will comprise three periods. The screening period will be up to 6 weeks in duration. The double-blind placebo-controlled treatment period will last 24 weeks. Following the final dose of investigational product (IP), there will be a 4-week safety follow-up period. IP will be administered orally once daily. During the initial six weeks of the treatment period, IP doses will be individually titrated at Weeks 2, 4, and 6 using echocardiography. Dose escalation at the Weeks 2, 4, and 6 visits will occur only if a patient has a post-Valsalva LVOT-G ≥30 mmHg and a biplane LVEF ≥55%. An echocardiogram will be performed at each subsequent visit during the trial and the dose down-titrated if necessary. The primary endpoint of pVO_2 will be measured by CPET at screening and at end of treatment (Week 24). Patients background oHCM therapy will be individually optimized according to local practice.

A CMR imaging sub-study will be open to approximately 100 patients who consent to participate.

Trial Center(s):

This trial will take place at approximately 120 sites worldwide.

Number of Patients:

Approximately 270 patients will be randomized to CK-3773274 or placebo.

Key Eligibility Criteria:

The key eligibility criteria are below. A full listing of eligibility criteria can be found in Section 5.

Inclusion Criteria

- Males and females between 18 and 85 years of age, inclusive, at screening.
- Body mass index <35 kg/m^2.
- Diagnosed with HCM per the following criteria:
  - Has LV hypertrophy and non-dilated LV chamber in the absence of other cardiac disease and
  - Has an end-diastolic LV wall thickness as measured by the echocardiography core laboratory of:
    a. ≥15 mm in one or more myocardial segments OR
    b. ≥13 mm in one or more wall segments *and* a known-disease-causing gene mutation or positive family history of HCM
- Has resting LVOT-G ≥30 mmHg and post-Valsalva LVOT G ≥50 mmHg during screening as determined by the echocardiography core laboratory.
- LVEF ≥60% at screening as determined by the echocardiography core laboratory.
- NYHA Functional Class II or III at screening.
- Hemoglobin ≥10g/dL at screening.
- Respiratory exchange ratio (RER) ≥1.05 and pVO2 ≤90% predicted on the screening CPET per the core laboratory.

- Patients on beta-blockers, verapamil, diltiazem, or disopyramide should have been on stable doses for >6 weeks prior to randomization and anticipate remaining on the same medication regimen during the trial. Patients treated with disopyramide must also be concomitantly treated with a beta blocker and/or calcium channel blocker.

Exclusion Criteria

Any of the following criteria will exclude potential patients from the trial:

- Known or suspected infiltrative, genetic or storage disorder causing cardiac hypertrophy that mimics oHCM (eg, Noonan syndrome, Fabry disease, amyloidosis).
- Significant valvular heart disease (per investigator judgment).
  - Moderate-severe valvular aortic stenosis.
  - Moderate-severe mitral regurgitation not due to systolic anterior motion of the mitral valve.
- History of LV systolic dysfunction (LVEF <45%) or stress cardiomyopathy at any time during their clinical course.
- Inability to exercise on a treadmill or bicycle (eg, orthopedic limitations).
- Has been treated with septal reduction therapy (surgical myectomy or percutaneous alcohol septal ablation) or has plans for either treatment during the trial period.
- Documented paroxysmal atrial fibrillation during the screening period.
- Paroxysmal or permanent atrial fibrillation is only excluded **IF**:
  - rhythm restoring treatment (eg, direct-current cardioversion, atrial fibrillation ablation procedure, or antiarrhythmic therapy) has been required ≤6 months prior to screening
  - rate control and anticoagulation have not been achieved for at least 6 months prior to screening.
- History of syncope or sustained ventricular tachyarrhythmia with exercise within 6 months prior to screening.
- Has received prior treatment with CK-3773274 or mavacamten.

<u>*Exclusion Criteria for CMR sub-study*</u>

- Inability to tolerate CMR.
- Has an implantable cardioverter-defibrillator (ICD).
- Has a cardiac pacemaker.

Data Monitoring Committee:

An independent Data Monitoring Committee (DMC) will be established for this trial to formally review the accumulating data periodically in order to assess risk to patients during the conduct of the trial. Details regarding the scope of responsibilities, meetings and communication procedures, as well as information requirements will be outlined in the DMC Charter. The DMC will have access to actual treatment assignments and patient-level data from the clinical trial database.

Statistical Methods:

Sample Size Calculation: assuming a difference in change from baseline in pVO_2 of 1.5 mL/kg/min for CK-3773274 compared to placebo, a standard deviation (SD) of 3.5 mL/kg/min, and 10% of patients missing change from baseline data of the primary endpoint, a sample size of 270 patients (approximately 135 randomized to CK-3773274 and 135 randomized to placebo) provides more than 90% power to detect the difference in pVO_2 change from baseline to Week 24 with a 2-sided type I error of 0.05.

During the study, the aggregate pooled missing data rate and overall pooled SD for the change from baseline in pVO_2 at Week 24 will be monitored periodically in a blinded fashion. If the pooled SD is larger than expected, Cytokinetics may consider increasing the sample size once in order to maintain the intended power.

Unless specified otherwise, efficacy analyses will be performed on the full analysis set (FAS), which includes all randomized patients. The primary analysis will test the null hypothesis that there is no treatment difference in the primary endpoint between patients randomized to placebo and those randomized to CK-3773274 in the FAS. Change from baseline in pVO_2 will be analyzed using an ANCOVA model with treatment group, randomization stratification factors, baseline pVO_2 and baseline weight as covariates.

For preservation of the overall type I error rate at two-sided 0.05 for the primary and secondary endpoints will be tested in the following specified order using a closed testing procedure. First, the primary endpoint is tested first at two-sided 0.05. If the primary endpoint achieves statistical significance at two-sided $p \le 0.05$, then the secondary endpoints will be tested at two-sided 0.05, with their testing being in the sequential order of KCCQ-CSS change from baseline, proportion of patients with ≥1 NYHA functional class improvement, post-Valsalva LVOT-G change from baseline, proportion of patients with post-Valsalva LVOT-G < 30 mm Hg, and duration of SRT eligibility for participants who are SRT eligible at baseline, for each after 24 weeks of treatment; then KCCQ-CSS change from baseline, proportion of patients with ≥1 NYHA functional class improvement, post-Valsalva LVOT-G change from baseline, and proportion of patients with post-Valsalva LVOT-G < 30 mmHg, for each after 12 weeks of treatment; and lastly change from baseline to Week 24 in total workload. SRT eligibility is defined as resting or post-Valsalva LVOT-G ≥ 50 mmHg AND NYHA Functional Class ≥ 3. See Figure 1 for illustration of the testing order. The detailed description of testing sequence will be provided in the Statistical Analysis Plan.

Figure 1: Statistical Testing Hierarchy for Trial Endpoints

Primary Endpoint		Significance Level
Step 1	pVO_2	0.05
Secondary Endpoints		↓
Step 2	KCCQ-CSS (24 wk)	0.05
		↓
Step 3	NYHA Class (24 wk)	0.05
		↓
Step 4	Valsalva Gradient (24 wk)	0.05
		↓
Step 5	%Valsalva Gradient (24 wk)	0.05
		↓
Step 6	Duration SRT Eligible (24 wk)	0.05
		↓
Step 7	KCCQ-CSS (12 wk)	0.05
		↓
Step 8	NYHA Class (12 wk)	0.05
		↓
Step 9	Valsalva Gradient (12 wk)	0.05
		↓
Step 10	%Valsalva Gradient (12 wk)	0.05
		↓
Step 11	Total workload (24 wk)	0.05

The proportion of responders in various exploratory endpoints will be analyzed using Cochran–Mantel–Haenszel (CMH) test stratified by randomization factors in FAS. The p-value and 95% confidence interval (CI) will be obtained using exact method. For the subgroup of patients who are SRT-eligible at baseline, total duration SRT eligible during the 24-week treatment period will be analyzed using an ANCOVA model with treatment group and randomization stratification factor beta blocker use/no use as fixed effects adjusting for significant baseline characteristics. Other change from baseline endpoints will be analyzed using mixed measures repeated model with treatment, visit, randomization stratification factors, treatment by visit, baseline by visit interaction as fixed effect and baseline assessment as covariate.

Safety analyses will be performed on the safety analysis set (SAS) which includes all patients who received at least one dose of IP. The pharmacokinetics analysis set (PKS) will consist of patients who have at least one measurable plasma concentration of CK-3773274.

The number and percentage of patients reporting any treatment-emergent AEs will be coded using the MedDRA dictionary and be tabulated by system organ class and preferred term.

Analyses will be further detailed in the Statistical Analysis Plan.

1.2. Schema

Figure 2: Trial Schema

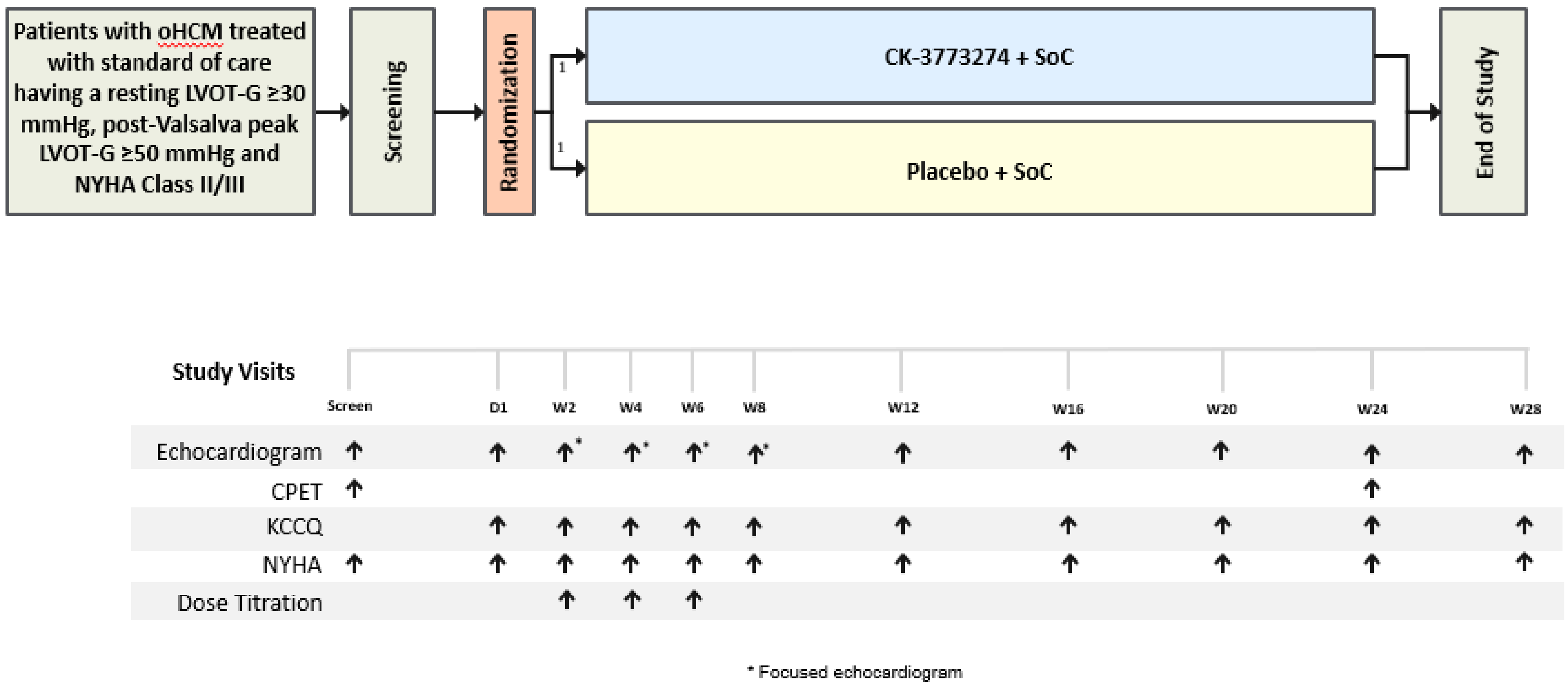

1.3. Schedule of Activities

Trial Procedure	Screening[a] (≤ 42 days)	Day 1	Week 2	Week 4	Week 6	Week 8	Week 12	Week 16	Week 20	EOT[b] (Week 24)	EOS[c] (Week 28)	ED[d]
**Visit Window**	**Up to 42 days prior to Day 1**	**N/A**	**+ 3 days**	**+ 3 days**	**+ 3 days**	**+ 3 days**	**± 3 days**	**± 7 days**	**± 7 days**	**± 7 days**	**+ 7 days**	**As soon as possible after withdrawal**
**GENERAL PROCEDURES AND SAFETY ASSESSMENTS**												
Informed consent	X											
Enrollment in IWRS	X											
Inclusion/Exclusion criteria	X											
Medical/Surgical history	X											
Demographics	X											
Height/weight[e]	X									X		
Vital signs[f]	X	X	X	X	X	X	X	X	X	X	X	X
Physical examination	X									X		X
Adverse events/serious adverse events[g]	X	X	X	X	X	X	X	X	X	X	X	X
Concomitant medications	X	X	X	X	X	X	X	X	X	X	X	X
12-lead ECG (triplicate)	X	X	X	X	X	X	X	X	X	X	X	X
CPET	X									X		
CMR imaging sub-study[h]	X									X		
Focused Echo (LVOT-G + LVEF)[i]			X	X	X	X						
Full Echocardiogram[i]	X	X					X	X	X	X	X	X
Randomization		X										
**CENTRAL LABORATORY ASSESSMENTS**												
Laboratory assessments	X	X					X			X	X	X
CMR substudy Hematocrit[j]	X									X		
Pregnancy test (WOCBP only)[k]	X	X	X	X	X	X	X	X	X	X	X	X
NT-pro-BNP		X	X	X	X	X	X	X	X	X	X	X

Trial Procedure	Screening[a] (≤ 42 days)	Day 1	Week 2	Week 4	Week 6	Week 8	Week 12	Week 16	Week 20	EOT[b] (Week 24)	EOS[c] (Week 28)	ED[d]
**Visit Window**	**Up to 42 days prior to Day 1**	**N/A**	**+ 3 days**	**+ 3 days**	**+ 3 days**	**+ 3 days**	**± 3 days**	**± 7 days**	**± 7 days**	**± 7 days**	**+ 7 days**	**As soon as possible after withdrawal**
hs-cTnI		X	X	X	X	X	X	X	X	X	X	X
PK samples[l]		X	X	X	X	X	X	X	X	X		X
Other biomarker samples[m]		X					X			X		
Serum and Plasma Collection for Future Analyses		X					X			X		
Genotype sample[n]		X										
PT-INR[o]	X											
**PATIENT-REPORTED OUTCOMES AND FUNCTIONING ASSESSMENTS**												
NYHA Functional Classification	X	X	X	X	X	X	X	X	X	X	X	X
KCCQ[p]		X	X	X	X	X	X	X	X	X	X	X
EQ-5D-5L[p]		X	X	X	X	X	X	X	X	X	X	X
CGI										X	X	X
PGI-C[p]										X		X
SAQ-7[p]		X		X		X	X	X	X	X	X	X
**INVESTIGATIONAL PRODUCT**												
IP dose administration at site[q]		X	X	X	X	X	X	X	X	X		
IP dispensation		X	X	X	X	X	X	X	X			
IP dose titration[r]			X	X	X							
IP dose adjustment[s]						X	X	X	X			

CGI = Clinical Global Impression scale; CMR = cardiac magnetic resonance imaging; CPET = cardiopulmonary exercise testing; ECG = electrocardiogram; Echo = echocardiogram; ED = early discontinuation; EOS = end of study; EOT = end of treatment; EQ-5D-5L = EuroQol 5-dimension 5-level instrument; hs-cTnI = high sensitivity cardiac troponin I; IP = investigational product; IWRS = interactive web response system; KCCQ = Kansas City Cardiomyopathy Questionnaire; NT-proBNP = n-terminal prohormone brain natriuretic peptide; NYHA = New York Heart Association; SAQ-7 = Seattle Angina Questionnaire-7, PGI-C = Patient Global Impression of Change scale; PK = pharmacokinetic; WOCBP = women of childbearing potential; PT-INR = prothrombin time/international normalized ratio; UNS DILI = unscheduled drug-induced liver injury

[a] The CPET must be completed within four weeks but not less than one week prior to randomization.

[b] If a patient is temporarily unable to exercise on the treadmill or bicycle (whichever modality was used at baseline) due to an adverse event (eg, ankle sprain, upper respiratory infection, migraine), but not due to HCM symptoms, or if the site is unable to perform CPET (eg, equipment malfunction), then the Week 24 visit may be postponed by up to 4 weeks. The patient should continue to receive IP until the visit. Sites should contact patients shortly before the Week 24 visit and confirm their ability to perform CPET. If necessary, the Week 24 visit may be split across two consecutive days within the visit window. If the visit is split, all assessments, except the CPET, should occur on the first day of the split visit. The CPET should occur on the second day of the split visit. Dosing will occur on site on both split visit days.

[c] The EOS visit (Week 28) will occur 4 weeks after last dose. It is not required for patients who discontinue IP >4 weeks prior to the Week 24.

[d] Patients who withdraw from the trial IP should complete an early discontinuation visit as soon as possible if they no longer wish to be part of the study. An EOS visit should be performed 4 weeks after their final IP dose if possible.

[e] Height is measured at the Screening visit only.

[f] Vital signs include heart rate, respiratory rate, and blood pressure. Oxygen Saturation only done at Screening.

[g] Only SAEs and non- serious AEs considered related to trial procedures are collected during the screening period until initiation of IP (Day 1). Any medical occurrence not related to a trial procedure during this period should be collected as medical history.

[h] A CMR imaging sub-study will be open to approximately 100 patients who consent to participate. The baseline CMR imaging should be done prior to randomization. The EOT CMR should be performed after the Week 20 visit, but prior to the last dose of IP at Week 24.CMR should occur after CPET if the assessments are performed on the same day.

i Echocardiograms will be done prior to dosing on Day 1 and 2 hours after dosing in the clinic at other time points.

[j] Must be done within 24 hours of CMR.

[k] Only for WOCBP. Serum pregnancy test at Screening visit. A urine pregnancy test may be performed locally at all other required timepoints. If a urine pregnancy test is positive, a serum pregnancy test should be performed.

[l] When PK collection and an echocardiogram are scheduled for the same time point, PK collection should be completed prior to the echocardiogram.

[m] Other biomarkers are referenced in Table 9.

[n] A genotype sample will be collected on Day 1 from patients who provide consent.

[o] PT-INR should also be taken at UNS DILI visits in addition to the screening visit.

[p] All PROs, KCCQ, ED-5D-5L, SAQ-7, and PGI-C, should be done prior to other assessments.

[q] On clinic visit days after Day 1, patients should take IP after the blood draw. At the Week 24 visit, patients should take their final dose of IP before the CPET.

[r] See Section 6.6.

[s] See Section 7.1.

1.4. Key Contacts

**Sponsor's Trial Contact:**	Edward Robbie<br>Manager, Clinical Operations<br>Email: CY6031ClinicalOperations@cytokinetics.com<br>Phone: +1 (650) 624-3046<br>Fax: +1 (650) 624-3225
**Sponsor's Medical Monitor:**	Daniel Jacoby, MD<br>Senior Medical Director, Clinical Research, Cardiovascular<br>Email: CY6031MedicalMonitor@cytokinetics.com<br>Mobile: +1 (650) 410-0096<br>Fax: +1 (650) 624-3225
**Serious Adverse Event Reporting:**	Cytokinetics Drug Safety<br>Email: CY6031DrugSafety@cytokinetics.com<br>Fax: +1 (650) 243-4199

2. INTRODUCTION

This is a Phase 3 trial of CK-3773274, a small molecule, allosteric inhibitor of cardiac myosin being developed as a chronic oral treatment for patients with hypertrophic cardiomyopathy (HCM).

2.1. Trial Rationale

The development of a targeted therapeutic drug that directly reduces myocardial contractility in the sarcomere may yield potential advantages over current therapies for obstructive hypertrophic cardiomyopathy (oHCM) because it potentially addresses the underlying pathophysiology of HCM. CK-3773274 is a cardiac myosin inhibitor with potential to reduce left ventricular outflow tract (LVOT) obstruction and improve symptoms in patients with hyperdynamic ventricular contractility in oHCM.

This trial is intended to establish the efficacy and safety of CK-3773274 with respect to improvements in exercise capacity and patient symptoms, as well as reduction in left ventricular outflow tract gradient (LVOT-G) in patients with oHCM.

2.2. Background

2.2.1. Hypertrophic Cardiomyopathy

HCM results from pathogenic genetic mutations, often affecting the genes encoding the proteins of the cardiac sarcomere, such as myosin (Maron, B. J. 2018). Histologic features include myofibrillar disarray, myocyte hypertrophy and interstitial fibrosis. Clinically, HCM is characterized by left ventricular (LV) hypertrophy unexplained by loading conditions and a nondilated LV with preserved or increased ejection fraction (Gersh 2011). Imaging studies of patients with HCM show hypertrophied LV walls, enhanced ventricular contractility, normal end-diastolic LV volume, reduced end-systolic volume, impaired diastolic compliance and often left atrial enlargement (Marian 2017). From population-based insurance claims and national health system data, the prevalence of clinically identified individuals with HCM in the US and EU is approximately 1:2000 and 1:3195 (Maron, M. S. 2016; Husser 2018; Magnusson 2017; Pujades-Rodriguez 2018).

Approximately 70% of patients with phenotypic HCM will demonstrate an element of LVOT obstruction (Maron, M. S. 2006). The mechanisms for developing obstruction are well defined and involve a complex interplay between alterations in ventricular flow between asymmetric septal hypertrophy and the mitral valve leaflets. The result is abnormal systolic contact with the mitral valve leaflets (most commonly the anterior leaflet) and the development of an LVOT gradient (LVOT-G). By nature, oHCM is a dynamic condition with variable systolic gradients. In the setting of reduced afterload or reduced preload, symptoms change depending on the gradient and often worsen during exertion. Additional clinical manifestations of HCM include an elevated risk for ventricular fibrillation and sudden cardiac death; heart failure syndrome due to diastolic dysfunction; chest pain due to microvascular ischemia; palpitations and stroke due to atrial fibrillation; syncope and presyncope due to either ventricular arrhythmias or an abnormal blood pressure response to exercise; and, in a minority of patients, progression to systolic heart failure.

Contemporary management strategies for oHCM have resulted in the majority of patients achieving normal or near-normal longevity and improved morbidity; however, there has been little progress with the development of novel pharmacotherapies. Current medical treatment consists of beta-blockers, verapamil, diltiazem and disopyramide as recommended in the 2014 European Society of Cardiology and in the 2020 American College of Cardiology Foundation / American Heart Association guidelines for the diagnosis and management of HCM. For patients with advanced symptomatic disease unresponsive to medications, septal reduction therapies (surgical myectomy or percutaneous alcohol ablation of the septum) can provide effective LVOT-G reduction (Elliott 2014; Gersh 2011; Ponikowski 2016; Ommen 2020). A subgroup of patients, who have been resuscitated from sudden cardiac death or who are at risk of sudden cardiac death, may undergo placement of an implantable cardioverter defibrillator (ICD) (Kristensen 2014). For those patients with HCM with end-stage disease who have both significant systolic impairment and diastolic dysfunction, cardiac transplantation may be the only treatment option (Gersh 2011). Disease-related mortality is most often attributable to sudden cardiac death, heart failure, and embolic stroke.

Mutations in over a dozen genes encoding sarcomere-associated proteins cause HCM. MYH7 and MYBPC3, encoding β-myosin heavy chain and myosin-binding protein C, respectively, are the two most common genes involved, together accounting for approximately 50% of the HCM families (Elliott 2014). Mechanistically, mutations in HCM appear to increase the net power generation in the sarcomere in vitro (Chuan 2012; Sommese 2013; Spudich 2016; Toepfer 2019). The findings in these studies are consistent with the underlying myocardial pathophysiology of the LV in patients with HCM being hypercontractile with diminished compliance (Wilson 1967).

These nonclinical investigations have enhanced our understanding of the molecular pathogenesis of HCM and have stimulated efforts designed to identify cardiac myosin modulators that can target the underlying mechanism of hypercontractility in oHCM.

2.2.2. CK-3773274

CK-3773274, a small molecule allosteric inhibitor of cardiac myosin, is being developed as a chronic oral treatment for patients with HCM. CK-3773274 is designed to reduce the hypercontractility that underlies the pathophysiology of HCM in the cardiac sarcomere. The intended pharmacologic effect is reduction in force produced by the cardiac sarcomere resulting in less LVOT obstruction and improved diastolic function in patients with oHCM.

CK-3773274 has been studied in a Phase 1 study of healthy adult participants and a Phase 2 study of patients with oHCM. This Phase 3 trial will assess the efficacy and safety of CK-3773274 in patients with oHCM.

Please refer to the Investigator’s Brochure for detailed information on the nonclinical and clinical studies of CK-3773274.

2.3. Benefit/Risk Assessment

2.3.1. Risk Assessment

Excessive exposures to CK-3773274 may result in an excess of the intended pharmacodynamic (PD) effect, namely a decrease in LV systolic function, resulting in decreases in stroke volume and cardiac output with compensatory increases in heart rate. In nonclinical toxicology studies,

sustained depression of cardiac function led to increases in heart weight and dilatation of the cardiac chambers (Section 2.2.2). These adverse cardiac effects are consistent with the anticipated physiological response to an excessive PD effect of CK-3773274.

In the first-in-human CY 6011 study, short-term decreases in cardiac function produced no changes in the vital signs or electrocardiograms (ECGs) of the participants. The effect of CK-3773274 on left ventricular ejection fraction (LVEF) reversed within 24-48 hours (single ascending dose and multiple ascending dose cohorts) of discontinuation of dosing. The participants who had decreases in LVEF to <50% remained asymptomatic until their cardiac function returned to the normal LVEF range.

In the first three cohorts of the Phase 2 trial, CY 6021 (REDWOOD-HCM), there were no adverse events of decreased LVEF reported, however, a decline in LVEF < 50% per core laboratory evaluation was reported for 2 of 41 participants in the aficamten group, and no participants in the placebo group. The decline in LVEF was asymptomatic in both patients and returned to above 50% at the next observed time point 2 weeks later. One of these participants underwent per-protocol dose reduction from 20 mg to 10 mg daily and completed the treatment period without dose interruption. The second participant who completed study treatment was noted to have an LVEF of 49.3% at Week 10 (end of treatment visit). There were no reports of post-baseline LVEF < 40%.

Together these findings indicate that the treatment effect of CK-3773274 is well tolerated and readily reversible with either a reduction of dose or discontinuation of treatment.

2.3.1.1. Mitigation Strategy

The main mitigation strategy will be facilitated by an individualized dose titration scheme based on each patient’s PD response to CK-3773274 with application of prespecified echocardiographic criteria, including LVEF thresholds for dose escalation, down-titration, and drug discontinuation.

Patients enrolled in this trial will be required to have an LVEF ≥60% prior to randomization, as confirmed by the central echocardiography laboratory. A low starting dose of 5 mg and a maximum dose of 20 mg were chosen as these were found to be well-tolerated in the Phase 2 study (CY 6021) of patients with oHCM and effective at reducing the LVOT-G without adversely impacting overall LVEF. Dose escalation will be performed on an individualized basis only if the following criteria are met: both post-Valsalva LVOT-G ≥30 mmHg and biplane LVEF ≥55%. Importantly, in contrast to CY 6021, the lower limit of LVEF for dose escalations will be increased from 50% to 55% to provide a safety margin from the threshold of LVEF (<50%) that will trigger dose reduction. If the LVEF is <50% at any time, the dose of CK-3773274 will be down-titrated, and if the LVEF is <40% at any time, CK-3773274 will be temporarily interrupted.

An independent Data Monitoring Committee (DMC) will be established for this trial to formally review the accumulating data periodically in order to assess the risk to patients during the conduct of the trial. DMC members will include echocardiologists and HCM experts and will have access to treatment assignments and patient-level data in support of safety oversight.

2.3.2. CK-3773274 Benefit Assessment

The development of a targeted therapeutic drug that directly reduces myocardial contractility in the sarcomere may yield potential clinical benefit for patients with oHCM by trying to address the underlying pathophysiology of HCM. CK-3773274 is a cardiac myosin inhibitor with the potential to reduce LVOT obstruction and thereby reduce symptoms in patients with hyperdynamic ventricular contractility in oHCM.

In the Phase 2 trial, CY 6021 (REDWOOD-HCM), patients with oHCM received up to three doses of CK-3773274 or placebo (randomized 2:1) in a dose escalating manner using echocardiography to guide dose titration. Two cohorts of 21 (14 CK-3773274 and 7 placebo) and 20 patients (14 CK-3773274 and 6 placebo) each were enrolled and treated for 10 weeks. Doses in the first cohort were 5, 10, 15 mg once daily; the second cohort studied 10, 20, and 30 mg once daily. In both cohorts, CK-3773274 significantly and substantially reduced the LVOT gradient (LVOT-G) in a dose and exposure dependent manner. The majority of patients treated with CK-3773274 (78.6% in Cohort 1 and 92.9% in Cohort 2) achieved the target goal of treatment, defined as resting gradient <30 mmHg and post-Valsalva gradient <50 mmHg at Week 10 compared to placebo (7.7%). A third cohort of 13 patients taking disopyramide plus either a calcium-channel blocker or beta-blocker were enrolled and treated for 10 weeks. Doses were 5, 10, 15 mg once daily. CK-3773274 significantly and substantially reduced LVOT-G in dose and exposure dependent manner. Ten patients (77%) achieved complete or partial LVOT-G response at Week 10. Complete response was defined as rest and post-Valsalva LVOT-G <30 mmHg and <50 mmHG respectively. Partial response was defined as either rest LVOT-G ≥30mmHg with Valsalva LVOT-G <50 mmHG, or rest LVOT-G <30 mmHg with post-Valsalva LVOT-G ≥50 mmHg . Eleven of 13 (85%) achieved improvement in NYHA class by ≥1 class.

Given that the LVOT-G is the primary cause of symptoms in patients with oHCM, participation in this trial may afford those randomized to CK-3773274 symptom reduction and increased exercise capacity. Patient contributions to the performance of this trial may yield a new therapeutic modality for the treatment of their disease.

3. OBJECTIVES AND ENDPOINTS

Table 2: Trial Objectives and Endpoints

Objectives	Endpoint(s)
Primary	
To evaluate the effect of CK-3773274 on exercise capacity in patients with symptomatic oHCM	• Change in peak oxygen uptake (pVO_2) by cardiopulmonary exercise testing (CPET) from baseline to Week 24
Secondary	
To evaluate the effect of CK-3773274 on patient health status	• Change in Kansas City Cardiomyopathy Questionnaire – Clinical Summary Score (KCCQ-CSS) from baseline to Week 12 and Week 24
To evaluate the effect of CK-3773274 on New York Heart Association (NYHA) Functional Classification	• Proportion of patients with ≥1 class improvement in NYHA Functional Class from baseline to Week 12 and Week 24
To evaluate the effect of CK-3773274 on post-Valsalva left ventricular outflow tract gradients (LVOT-G)	• Change in post-Valsalva LVOT-G from baseline to Week 12 and Week 24<br>• Proportion of patients with post-Valsalva LVOT-G <30 mmHg at Week 12 and Week 24
To evaluate the effect of CK-3773274 on duration of eligibility for septal reduction therapy	• Duration of eligibility for septal reduction therapy (SRT) during the 24-week treatment period in patients who were eligible for SRT at baseline.
To evaluate the effect of CK-3773274 on exercise capacity	• Change in total workload during CPET from baseline to Week 24
Safety	
To evaluate the safety and tolerability profile of CK-3773274 in patients with symptomatic oHCM	• Incidence of reported major adverse cardiac events (cardiovascular [CV] death, cardiac arrest, non-fatal stroke, non-fatal myocardial infarction, CV hospitalization)<br>• Incidence of new onset persistent atrial fibrillation<br>• Incidence of appropriate implantable cardiac defibrillator (ICD) discharges and aborted sudden cardiac death<br>• Incidence of left ventricular ejection fraction (LVEF) <50%<br>• Incidence of LVEF <50% with at least one of the following:<br>o Signs and symptoms of heart failure (concomitant adverse event of heart failure or dyspnea), AND/OR<br>o Increase in NT-proBNP (≥30% increase), relative to results from the most recent previous visit and above the upper limit of normal, at the time of LVEF assessment <50%<br>• Incidence of treatment emergent adverse events

Table 2: Trial Objectives and Endpoints (Continued)

Objectives	Endpoint(s)
Exploratory	
To evaluate the effect of CK-3773274 on exercise capacity and functional class	• Compared with baseline, proportion of patients at Week 24 achieving either:<br>– Change from baseline of ≥1.5 mL/kg/min in pVO_2 AND ≥1 class improvement in NYHA Functional Class<br>**OR**<br>– Change from baseline of ≥3.0 mL/kg/min in pVO_2 AND no worsening of NYHA Functional Class
To evaluate the effect of CK-3773274 on patient response over time	• Proportion of patients with improvement in KCCQ-CSS ≥5 points at Weeks 12 and 24<br>• Proportion of patients with resting LVOT-G <30 mmHg, post-Valsalva LVOT-G <50 mmHg, and NYHA Functional Class I at Weeks 12 and 24<br>• Proportion of patients with resting LVOT-G <30 mmHg, post-Valsalva LVOT-G <50 mmHg, and ≥1 class improvement in NYHA Functional Class at Weeks 12 and 24
To evaluate the effect of CK-3773274 on septal reduction therapy eligibility	• Proportion of patients who are eligible for septal reduction therapy at Week 24 among patients who were eligible for septal reduction therapy at baseline
To evaluate the effect of CK-3773274 on other CPET parameters	Change from baseline to Week 24 in:<br>• Ventilatory efficiency (VE/VCO_2 slope)<br>• Circulatory power (VO_2 × systolic BP)<br>• Ventilatory anaerobic threshold (VAT)
To evaluate the effect of CK-3773274 on health status and health-related quality of life as measured by PRO questionnaire	• Change from baseline to Week 24 in individual responses to the EuroQol 5-dimension 5-level instrument (EQ-5D-5L)
To evaluate the effect of CK-3773274 on health status and quality of life related to chest pain-like angina	Change from baseline to Week 24 in summary and domain scores for the Seattle Angina Questionnaire-7 (SAQ-7)
To evaluate the effect of CK-3773274 on cardiac function and structure	• Change from baseline to Week 24 in echocardiographic measurements of cardiac structure and of systolic function including:<br>– LVEF<br>– Left ventricular end-systolic and end-diastolic volumes (LVESV and LVEDV, respectively)<br>– Left atrial volume

Table 2: Trial Objectives and Endpoints (Continued)

Objectives	Endpoint(s)
To evaluate the effect of CK-3773274 on biomarker levels	• Change from baseline values in NT-pro-BNP, hs-cardiac-TnI and other biomarkers through Week 24
To evaluate the effect of CK-3773274 on left ventricular mass, function, and structure by cardiac magnetic resonance (CMR) imaging	• Change from baseline to Week 24 in CMR measurements of:<br>– Left ventricular (LV) mass index<br>– LVEF<br>– Septal, free wall, and maximal wall thickness<br>– Left atrial volume index<br>– LVESV<br>– LVEDV
To assess the pharmacokinetics of CK-3773274 and its metabolites	• Pharmacokinetic parameters through Week 24

4. TRIAL DESIGN

4.1. Overall Design

This is a Phase 3, randomized, placebo-controlled, double-blind, multi-center trial in patients with symptomatic oHCM. Approximately 270 eligible patients will be randomized in a 1:1 ratio to receive CK-3773274 or placebo. Randomization will be stratified by use of beta-blockers (yes or no) and CPET exercise modality (treadmill or bicycle) and implemented in the Interactive Web Response System (IWRS). A cap on the number of patients taking beta-blockers and will not exceed approximately 70% of total enrollment. The number of patients taking disopyramide will be capped at approximately 10% of total enrollment. The number of patients with persistent atrial fibrillation at screening will also be capped at approximately 15%, and the number of patients using the bicycle CPET exercise modality will be capped at approximately 50% as well.

IP will be administered orally once daily with or without food. During the initial six weeks of the treatment period, IP doses will be individually titrated at Weeks 2, 4, and 6 using echocardiography. Dose escalation at Weeks 2, 4, and 6 will occur only if a patient has a post-Valsalva LVOT-G ≥30 mmHg and a biplane LVEF ≥55%. Echocardiograms will be performed at each subsequent visit during the trial and the dose down titrated if necessary. The primary endpoint of pVO_2 will be measured by CPET at screening and at end of treatment (Week 24). Patients' background HCM therapy should be individually optimized according to the local practice.

All patients will be followed according to the Schedule of Activities (SoA) from randomization through the date of their final visit irrespective of whether the patient is continuing to receive IP, unless the patient has discontinued prematurely from the trial or withdrawn consent. An early discontinuation visit will be performed for patients who discontinue prematurely from the trial.

The overall study design is described by a trial schema in Figure 2.

The trial endpoints and objectives are defined in Table 2.

4.1.1. Number of Sites

Approximately 120 investigative sites worldwide will participate in this trial.

4.1.2. Number of Patients

Approximately 270 patients will be randomized in the trial.

4.1.3. Replacement of Patients

Patients who are withdrawn or removed from treatment or the trial will not be replaced.

4.1.4. Trial Duration

The trial will comprise three periods. After signing the informed consent form, patients will complete assessments to determine trial eligibility during a screening period of up to 6 weeks in duration. The double-blind placebo-controlled treatment period will last 24 weeks. Following the final dose of IP, there will be a 4-week safety follow-up period.

4.1.5. CMR Imaging Sub-Study

A CMR imaging sub-study will be open to approximately 100 patients who consent to participate.

4.2. Scientific Rationale for Trial Design

This trial is designed to provide data supporting the clinical efficacy and safety of CK-3773274 in patients with symptomatic oHCM and an LVOT-G >50 mmHg post-Valsalva. Reduction of the LVOT-G is expected to correlate with improvement in the patients' symptoms, health status and exercise capacity.

Since patient characteristics vary substantially in this disease, individualized dose titration to a PD response (reduction of the post-Valsalva LVOT-G to <30 mmHg with preservation of LVEF ≥55%) is being employed to maximize efficacy and safety. The eligibility criteria are designed to enable enrollment of a patient population representative of the general population of patients with oHCM while ensuring the safety of the patients in this trial.

A placebo control and double-blinded approach are being employed in this trial to avoid bias in data collection, including the safety assessments and PD measures that comprise the primary and secondary endpoints.

4.3. Justification for Dose

The doses of CK-3773274 are summarized in Table 3. A starting dose of 5 mg and a maximum dose of 20 mg were chosen as these were found to be well-tolerated in the Phase 2 study of patients with oHCM and effective at reducing the LVOT-G without adversely impacting overall LVEF. Within-patient dose escalation will only occur when the patient's current dose is well-tolerated, and the patient meets the criteria described in Section 6.6.

4.4. End of Study Definition

The end of the study is defined as the date of the last visit of the last patient in the trial.

5. STUDY POPULATION

Before patients begin any trial-specific activities/procedures, Cytokinetics requires a copy of the site's institutional review board/independent ethics committee (IRB/IEC) approval of the protocol, informed consent form (ICF), and all other patient information and/or recruitment material, if applicable. A signed ICF must be obtained from each patient before commencement of any trial-specific activities/procedures.

A patient's participation in the trial begins after signing the informed consent. After confirming the patient has met all eligibility criteria, randomization should then occur before the first dose on Day 1 is administered. The site is to document the informed consent signature and randomization dates in the patient's medical record and in/on the case report form (CRF).

Prospective approval of protocol deviations to recruitment and enrollment criteria, also known as protocol waivers or exceptions, is not permitted.

5.1. Inclusion Criteria

Patients are eligible to be included in the trial only if all the following criteria apply:

101. Able to comprehend and willing to sign an ICF and willing to comply with all trial procedures and restrictions for the duration specified in the Schedule of Activities (SoA; Section 1.3).
102. Males and females between 18 and 85 years of age, inclusive, at screening.
103. Body mass index <35 kg/m^2.
104. Diagnosed with HCM per the following criteria:
    a. Has LV hypertrophy and non-dilated LV chamber in the absence of other cardiac disease and
    b. Has an end-diastolic LV wall thickness as measured by the echocardiography core laboratory of:
        - ≥15 mm in one or more myocardial segments OR
        - ≥13 mm in one or more wall segments *and* a known-disease-causing gene mutation or positive family history of HCM
105. Has resting LVOT-G ≥30 mmHg and post-Valsalva LVOT-G ≥50 mmHg during screening as determined by the echocardiography core laboratory
106. LVEF ≥60% at screening as determined by the echocardiography core laboratory.
107. New York Heart Association (NYHA) Functional Class II or III at screening
108. Hemoglobin ≥10 g/dL at screening.
109. Respiratory exchange ratio (RER) ≥1.05 and pVO_2 ≤90% predicted on the screening CPET per the core laboratory.
110. Patients on beta-blockers, verapamil, diltiazem, or disopyramide should have been on a stable regimen for >6 weeks prior to randomization and anticipate remaining on the same medication regimen during the trial. Patients treated with disopyramide must also be concomitantly treated with a beta blocker and/or calcium channel blocker.

111. Male patients are eligible to participate if they agree to the following:

a) Refrain from donating sperm during the trial plus at least 10 weeks after the last dose of IP

AND

b) During the trial plus 4 weeks after the last dose of IP either:

1. Be abstinent from heterosexual intercourse as their preferred and usual lifestyle (abstinent on a long term and persistent basis) and agree to remain abstinent in writing

OR

2. Must agree to use a male condom when his female partner is a woman of childbearing potential, and have his female partner use a highly effective method of contraception (as described in Appendix 3 [Section 10.3])

112. A female patient is eligible to participate if she is not pregnant, breastfeeding or planning to donate eggs, and at least one of the following conditions applies:

a. Is not a woman of childbearing potential (WOCBP; as described in Appendix 3 [Section 10.3])

OR

Is a WOCBP and using a highly effective method of contraception (as described in Appendix 3 [Section 10.3]) and male partner agrees to use a condom, during the trial and for at least 4 weeks after the last dose of IP.

b. A WOCBP must have a negative pregnancy test (urine or serum as required by local regulations) at Day 1, prior to the first dose of study IP.

Note: The investigator is responsible for review of medical history, menstrual history, and recent sexual activity to decrease the risk for inclusion of a woman with an early undetected pregnancy.

Contraceptive use by men or WOCBPs should be consistent with the guidance in Appendix 3 (Section 10.3) and local regulations regarding the methods of contraception for those participating in clinical studies.

113. Willing and able to complete all screening procedures.

5.2. Exclusion Criteria

Patients will be excluded from the trial if any of the following criteria apply:

201. Significant valvular heart disease (per investigator judgment).

a. Moderate-severe valvular aortic stenosis and/or regurgitation.
b. Moderate-severe mitral regurgitation not due to systolic anterior motion of the mitral valve

202. Documented history of current obstructive coronary artery disease (>70% stenosis in one or more epicardial coronary arteries) or documented history of myocardial infarction.

203. Known or suspected infiltrative, genetic or storage disorder causing cardiac hypertrophy that mimics oHCM (eg, Noonan syndrome, Fabry disease, amyloidosis).

204. Prior treatment with cardiotoxic agents such as doxorubicin or similar.

205. History of LV systolic dysfunction (LVEF <45%) or stress cardiomyopathy at any time during their clinical course.

206. Has any ECG abnormality considered by the investigator to pose a risk to patient safety (eg, second degree atrioventricular block type II).

207. Documented paroxysmal atrial fibrillation during the screening period.

208. Paroxysmal or permanent atrial fibrillation is only excluded IF:
    - rhythm restoring treatment (eg, direct-current cardioversion, atrial fibrillation ablation procedure, or antiarrhythmic therapy) has been required ≤6 months prior to screening
    - rate control and anticoagulation have not been achieved for at least 6 months prior to screening

209. History of syncope or sustained ventricular tachyarrhythmia with exercise within 6 months prior to screening.

210. ICD placement within 3 months prior to screening or planned ICD placement during the trial.

211. History of appropriate ICD discharge for life-threatening ventricular arrhythmia within 6 months prior to screening.

212. Has been treated with septal reduction therapy (surgical myectomy or percutaneous alcohol septal ablation) or cannot postpone plans for septal reduction therapy until after the trial period.

213. Inability to exercise on a treadmill or bicycle (eg, orthopedic limitations).

214. Documented room air oxygen saturation reading <90% at screening.

215. Hepatic impairment defined by a total bilirubin (TBL) ≥1.5 × the upper limit of normal (ULN), or alanine aminotransferase (ALT) or aspartate aminotransferase (AST) ≥3 × ULN at screening. Patients with documented Gilbert syndrome and TBL ≥1.5 × ULN due to unconjugated hyperbilirubinemia, without other hepatic impairment, are permitted.

216. Recipient of a major organ transplant (eg, heart, lung, liver, bone marrow, renal) or anticipated transplantation within 12 months from randomization.

217. History or evidence of any other clinically significant disorder, malignancy, active infection, other condition, or disease that, in the opinion of the investigator or the Medical Monitor, would pose a risk to patient safety or interfere with the trial evaluation, procedures, or completion.

218. Estimated glomerular filtration rate (eGFR) <30 mL/min/1.73 m^2 (by the modified Modification of Diet in Renal Disease equation) at screening.
219. Currently participating in another investigational device or drug trial or received an investigational device or drug <1 month (or 5 half-lives for drugs, whichever is longer) prior to screening. Other investigational procedures while participating in this trial are not permitted.

    Note: At the EOS visit for CY 6031, screening assessments for entry into an open-label extension study of aficamten are permitted.
220. Has received prior treatment with CK-3773274 or mavacamten.
221. Any known hypersensitivity to excipients in study drug tablets

Exclusion Criteria for CMR sub-study

222. Inability to tolerate CMR.
223. Has an ICD.
224. Has a cardiac pacemaker.
225. Does not consent to participate in CMR sub-study

5.3. Lifestyle Considerations

Patients will abstain from strenuous exercise for 24 hours before each blood collection for clinical laboratory tests and before CPET. Patients should not exercise at all for 12 hours prior to CPET. Patients should also fast for at least 4 hours prior to CPET. Any medications that may cause drowsiness should be avoided for 8 hours prior CPET.

5.4. Screen Failures

Screen failures are defined as patients who consent to participate in the clinical trial but are not subsequently randomized to IP. A minimal set of screen failure information is required to ensure transparent reporting of screen failure patients to meet the Consolidated Standards of Reporting Trials publishing requirements and to respond to queries from regulatory authorities. Minimal information includes demographics, reason for screen failure, eligibility criteria, and any SAEs related to trial-related procedures.

The screening eligibility period is 42 days as defined in Section 4.1. An individual who does not meet the criteria for participation in this trial is referred to as a screen failure. Patients may be rescreened one time after initial screening when the reason for screen failure is resolved or expected to be resolved.

Patients must re-sign an informed consent before they are rescreened. At rescreening, they must meet all inclusion/exclusion criteria at the time of rescreening and have all elements of the screening visit performed again to be eligible. Patients who screen fail and rescreen do not need a repeat CMR.

If an element of the screening visit could not be performed for logistical or technical reasons (eg, trained evaluator was out sick, equipment malfunction) the patient can return within the

screening eligibility period to complete the visit. Echocardiograms and CPETs failed due to technical insufficiencies can be repeated.

Retesting for abnormal laboratory results within the original screening period may also be performed if there is reason to believe the repeat laboratory results may improve and not be clinically significant and the patient is otherwise eligible to participate. Patients cannot be retested for abnormal bilirubin laboratory results. Patients who are retested during the original screening eligibility period do not need to re-sign an informed consent. The screening window is established by the date of the original screening visit and not by the date that laboratory retesting takes place. Patients who repeat echocardiograms and CPETs due to technical insufficiencies do not need to re-sign an informed consent.

No waivers will be granted regarding inclusion/exclusion criteria.

6. INVESTIGATIONAL PRODUCT

This section describes any IP, marketed product(s), or placebo intended to be administered to a trial patient according to the study protocol.

6.1. Investigational Product(s) Administered

Table 3: Investigational Products

Arm Name	Active	Placebo
**IP/Product Name**	CK-3773274	Placebo
**Type**	Drug	Drug
**Dose Formulation**	Tablet	Tablet
**Unit Dose Strength(s)**	5mg	Matching placebo
**Dosage Level(s)**	5mg, 10mg, 15mg, 20mg	
**Route of Administration**	Oral	Oral
**Use**	Experimental	Placebo
**IMP and NIMP**	IMP	IMP
**Sourcing**	Patheon Inc.<br>Toronto Regional Operations (TRO)<br>2100 Syntex Court<br>Mississauga, Ontario L5N 7K9<br>Canada	Patheon Inc.<br>Toronto Regional Operations (TRO)<br>2100 Syntex Court<br>Mississauga, Ontario L5N 7K9<br>Canada
**Excipients**	Microcrystalline Cellulose<br>Mannitol<br>Croscarmellose Sodium<br>Hydroxypropyl Cellulose<br>Sodium Lauryl Sulfate<br>Magnesium Stearate<br>Opadry QX White	Microcrystalline Cellulose<br>Lactose Monohydrate<br>Croscarmellose Sodium<br>Magnesium Stearate<br>Opadry QX White
**Packaging and Labeling**	IP will be provided in blister packs which will be labeled as required per country requirement	IP will be provided in blister packs which will be labeled as required per country requirement

IMP = investigational medicinal product; NIMP = non-investigational medicinal product

6.2. Preparation/Handling/Storage/Accountability

The investigator or designee must confirm appropriate temperature conditions have been maintained during transit for all IP received and any discrepancies are reported and resolved before use of the IP.

Only patients randomized in the trial may receive IP and only authorized site staff may supply or administer IP. All IP must be stored in a secure, environmentally controlled, and monitored

(manual or automated) area in accordance with the labeled storage conditions with access limited to the investigator and authorized site staff.

The investigator, institution, or the head of the medical institution (where applicable) is responsible for IP accountability, chain of custody, reconciliation, and record maintenance (ie, receipt, reconciliation, and final disposition records).

Patients should be instructed to take four tablets each day from one row on the blister pack. Further guidance and information regarding IP storage condition, dispensation, packaging, labeling, and accounting procedures are provided in the Pharmacy Manual.

IP should be stored at or below 25°C.

6.3. Measures to Minimize Bias: Randomization and Blinding

All eligible patients will be centrally assigned to randomized IP using the IWRS. Before the trial is initiated, the login information & directions for the IWRS will be provided to each site.

Because viewing echocardiogram results could potentially compromise the blinded investigator and blinded study coordinator, specified unmasked study staff will perform and read the echocardiograms.

An unmasked sonographer at the site will perform the echocardiograms. An unmasked cardiologist, who is not the investigator and is called the unmasked echocardiologist, will read the echocardiograms, measure the LVOT-G and LVEF and enter the echocardiogram results in the IWRS for dose titrations and adjustments. An unmasked designee, who is also not involved in other aspects of the study visits, may be delegated to enter data into IWRS on the unmasked echocardiologist's behalf. All site staff, including the unmasked echocardiologist and the unmasked designee, will be blinded to randomized treatment assignments. Neither the unmasked echocardiologist nor the unmasked data entry designee will reveal echocardiogram results to the rest of the study team, except in the event of a critical safety issue (eg, LVEF < 40%). Therefore, the investigator and site staff will remain blinded to the echocardiogram images and results.

IP will be dispensed at the trial visits summarized in the SoA (Section 1.3). Returned IP should not be re-dispensed to the patients.

Patients randomized to placebo will receive placebo throughout the study and will perform all protocol procedures in order to maintain the blind for the treatment group allocation and IP dose. Patients should continue to take IP through the morning of the Week 24 Visit.

The IWRS will be programmed with blind-breaking instructions and the unblinding procedure is documented in the study manual. In case of an emergency, the investigator has the sole responsibility for determining if unblinding of a patient's intervention assignment is warranted. Patient safety must always be the first consideration in making such a determination. If the investigator decides that unblinding is warranted, the investigator is encouraged to contact Cytokinetics prior to unblinding a patient's intervention assignment unless this could delay emergency treatment of the patient. If a patient's intervention assignment is unblinded, Cytokinetics must be notified within 24 hours after breaking the blind. The date and reason that the blind was broken must be recorded in the source documentation and CRF, as applicable.

6.4. Investigational Product Compliance

When patients are dosed at the site, the date and time of the dose administered in the clinic will be recorded in the source documents and recorded in the CRF.

When patients self-administer IP at home, compliance with IP will be assessed at each site visit. Compliance will be assessed by counting returned tablets during the site visits and documented in the source documents and CRF. Deviation(s) from the prescribed dosage regimen should be recorded in the CRF.

For IP accountability, a record of the following should be documented in the CRF at every visit and reconciled with IP and compliance records:

- the date and kits dispensed
- date and tablets returned

IP dosing first and last dates, including dates for dosing interruptions will also be recorded in the CRF.

6.5. Concomitant Therapy

Any medication, including over-the-counter or prescription medicines, vitamins, and/or herbal supplements, that the patient is receiving at the time of enrollment or receives during the trial must be recorded along with:

- Reason for use
- Dates of administration including start and end dates
- Dosage information including dose and frequency

Patients on beta-blockers, verapamil, diltiazem, or disopyramide should have been on stable doses for >6 weeks prior to randomization and anticipate remaining on the same medication regimen during the trial. Patients treated with disopyramide must also be concomitantly treated with a beta blocker and/or calcium channel blocker. Patients' background HCM therapy should be individually optimized according to the local practice.

The Medical Monitor should be contacted if there are any questions regarding concomitant or prior therapy.

Patients may continue to take prescription medications, which in the opinion of the investigator and the Medical Monitor, will not interfere with the trial.

While medications and doses should remain stable whenever appropriate during the trial, investigators may prescribe or adjust any concomitant medication or treatment deemed necessary to provide adequate supportive care.

6.5.1. Drug-Drug Interactions

In vitro studies showed that CK-3773274 was metabolized by CYP2D6 and CYP3A with potential contributions from CYPs 2C9 and 2C19. Since CYP2D6 was identified in vitro as a metabolizing enzyme of CK-3773274, the degree of dependence of its metabolism on CYP2D6 was explored in the first-in-human study, CY 6011, in participants with a poor metabolizing

CYP2D6 genotype. Compared to participants with an extensive metabolizing CYP2D6 genotype, the pharmacokinetics of CK-3773274 in participants with a poor metabolizing genotype were not different, suggesting that CYP2D6-mediated interactions are unlikely. In the absence of clinical data, the use of strong CYP3A inhibitors or inducers should be used with caution and careful monitoring.

Caution may be needed when administering CK-3773274 with known substrates of P-gp until drug-drug interaction studies are completed. Contact the Medical Monitor to determine if a potentially meaningful drug-drug interaction may exist.

6.5.2. Rescue Medicine

The use of rescue medications in the event of a low cardiac output state (eg, dobutamine) is allowable at any time during the trial (Section 8.5). The date and time of rescue medication administration as well as the name and dosage regimen of the rescue medication must be recorded.

6.6. Dose Modifications

6.6.1. Scheduled Dose Titrations

Patients randomized to CK-3773274 may receive up to four escalating doses of IP over the initial 6 weeks of the trial as outlined below in Table 4. Patients receiving CK-3773274 start at a dose of 5 mg once daily (Dose 1) and may escalate through doses of 10, 15, and 20 mg once daily if they continue to meet the escalation criteria or will stop at their current dose when escalation criteria are not met.

6.6.1.1. Week 2 Visit

After randomization each patient will receive Dose 1 once daily for two weeks. At the Week 2 visit, the patient will have an echocardiogram 2 hours following administration of their dose of IP. Patients will up-titrate to Dose 2 if the following conditions are met on echocardiography:

- Post-Valsalva LVOT-G ≥30 mmHg, and the biplane LVEF ≥55%

Otherwise, the patient will remain on Dose 1.

If LVEF is <50% at Week 2, the IWRS will assign the patient to placebo.

6.6.1.2. Week 4 Visit

After two more weeks on the assigned dose, at the Week 4 visit each patient will have an echocardiogram 2 hours following administration of their dose of IP. Patients will up-titrate to the next higher dose if the following conditions are met on echocardiography:

- Post-Valsalva LVOT-G ≥30 mmHg, and the biplane LVEF ≥55%

Otherwise, the patient will remain on the same dose.

If LVEF is <50% at Week 4, the IWRS will assign the patient to the prior dose level or to placebo if the patient was on Dose 1.

6.6.1.3. Week 6 Visit

After 2 more weeks on the assigned dose, at the Week 6 visit each patient will have an echocardiogram 2 hours following administration of their dose of IP. Patients will up-titrate to the next higher dose if the following conditions are met on echocardiography:

- Post-Valsalva LVOT-G ≥30 mmHg, and the biplane LVEF ≥55%

Otherwise, the patient will remain on the same dose.

If LVEF is <50% at Week 6, the IWRS will assign the patient to the prior dose level or to placebo if the patient was on Dose 1.

Table 4: Echocardiogram Criteria for Scheduled Dose Titrations

Echocardiogram Criteria for Scheduled Dose Titrations in Weeks 2, 4, and 6

Biplane LVEF		Post-Valsalva LVOT-G	Action
<50%			Reduce Dose[a]
≥50% - 55%			No Dose Change
≥55%	and	<30 mmHg	No Dose Change
≥55%	and	≥30 mmHg	Increase Dose

[a] Once a patient's IP dose is down titrated, no further escalation is permitted. If LVEF <50% on 5 mg, the patient will receive placebo.

6.6.1.4. Week 8 Visit

After two additional weeks on the assigned dose, at the Week 8 visit each patient will have an echocardiogram 2 hours following administration of their dose of IP to ensure the LVEF is ≥50%.

If the LVEF is <50% at Week 8, the IWRS will assign the patient to the next lower dose or to placebo if the patient was on Dose 1.

6.6.2. Dose Reductions

After Week 6, no further dose escalations may occur. During the course of the study, for safety reasons, dose reductions may occur at scheduled or unscheduled visits. Dose reductions will be determined by the IWRS system based on echocardiography results. After Week 8, dose reductions will be based on echocardiogram results from the initial scheduled or unscheduled visits. If the LVEF is <50%, then the IWRS will assign the patient to the next lower dose or to placebo if the patient was on Dose 1. The IWRS will not further reduce the dose for at least seven days after the previous reduction.

6.6.3. LVEF Safety Threshold

If the unmasked echocardiologist observes that the LVEF has crossed the defined safety threshold of <40% or feels the patient requires urgent medical attention, the unmasked

echocardiologist will enter the LVEF value in the IWRS and discuss the results with the blinded investigator or qualified designee. The Medical Monitor will be informed in these cases.

If a patient's LVEF is <40% at any time, the following steps should occur after consultation with the Medical Monitor:

- IP should be stopped and held for at least 7 days.
- Repeat echocardiograms should be performed per investigator judgment until a normal LVEF (≥55%) has been documented at which point the patient can be re-started on IP after being down-titrated.
- Document dose interruption in the eCRF and include the reason for interruption, the date of the last dose, and the restart date (Section 7.1).

6.6.4. Hepatotoxicity Stopping and Rechallenge Rules

Patients with abnormal hepatic laboratory values (ie, alkaline phosphatase [ALP], AST, ALT, TBL) and/or international normalized ratio (INR) and/or signs/symptoms of hepatitis may meet the criteria for withholding or permanent discontinuation of IP or other protocol-required therapies as specified in the FDA Guidance for Industry Drug-Induced Liver Injury: Premarketing Clinical Evaluation, July 2009. See Appendix 5 (Section 10.5) for guidance on the assessment and management of abnormal hepatic laboratory values.

6.7. Access to Investigational Product after the End of the Study

Patients who complete CY 6031 and meet eligibility criteria will be offered participation in an open-label extension study of aficamten. Participation in the open-label extension study is at the discretion of the patient and not a condition of participation in CY 6031. The commitment to the conduct of an open-label extension trial will be at Cytokinetics's discretion.

7. TEMPORARY INTERRUPTION OF INVESTIGATIONAL PRODUCT, DISCONTINUATION OF INVESTIGATIONAL PRODUCT, AND PATIENT CONSENT WITHDRAWAL

Emergent safety concerns should be discussed with the Medical Monitor immediately upon occurrence or awareness to determine if the patient should continue, interrupt or permanently discontinue IP.

Unless a safety concern arises, the investigator should make every effort to keep a patient on the IP for as long as possible during the trial. The degree to which a patient withdraws from the trial varies. There are three types of discontinuation: temporary IP interruption, permanent IP discontinuation and patient withdrawal of consent.

7.1. Temporary IP Interruption

Initially, any IP interruption should be considered temporary unless permanent IP discontinuation is mandated by the protocol.

A temporary IP interruption:

- Will be implemented when a predefined safety threshold has been met (Section 6.6)
- May be considered by the investigator in the case of an AE/SAE or for another reason

If a temporary IP interruption occurred because a safety threshold was met, blinded treatment will be resumed at least 7 days later, either at a lower dose or with a permanent switch to placebo if the patient was at 5mg, as determined by the IWRS (Section 6.6.3).

If the IP was temporarily interrupted because of an AE/SAE, the investigator should make the best effort to resume IP as soon as practically possible, assuming there are no remaining safety concerns.

If dosing is interrupted for more than 3 consecutive days in the first 6 weeks and more than 7 consecutive days thereafter, the investigator should contact the Medical Monitor to discuss the patient.

All temporary IP interruptions of greater than 3 days should be recorded in the eCRF (stop and start dates and reason for interruption) and the Medical Monitor should be notified.

7.2. Permanent Discontinuation of IP

In all cases, patients should be encouraged to discuss stopping IP with the investigator or the investigator's designee. Best efforts should be made to address the patient's questions, adjust concomitant medical therapies if needed and arrange follow-up safety assessments. Refer to Section 7.2.1. for management of patients who permanently discontinue IP.

Any permanent discontinuation of IP should be recorded in the eCRF including the reason for permanent discontinuation.

Reasons for permanent IP discontinuation may include any of the following:

- Patient request
- Pregnancy

- All criteria for possible drug-induced liver injury (DILI) are met (Appendix 5 [Section 10.5])
- The investigator judges that continued administration of IP would be detrimental to the patient's safety or well-being.
- Protocol violation
- Lost to follow-up
- Any breaking of the trial blind requested by the investigator
- Death
- The Sponsor requests that the patient permanently discontinue IP

7.2.1. Management of Patients after Permanent Discontinuation of IP

If IP is permanently discontinued, the patient should be encouraged to remain in the trial to continue to obtain outcome measures and safety data (see Section 7.2).

There are several options for a patient after permanently discontinuing IP:

- Patient agrees to continue to return to clinic for all remaining trial visits.
- Patient agrees to complete the early discontinuation (ED) visit as soon as possible after the decision is made and complete an EOS visit.
- Patient only agrees to complete the ED visit.
- Patient agrees to be contacted by phone to obtain patient trial data.
- Patient withdraws consent (see Section 7.4) and does not agree to any further trial procedures or visits.
- For those patients who have not withdrawn consent and have difficulty returning for all remaining trial visits, they can be contacted by phone to obtain patient trial data.

7.3. Discontinuation from Trial Procedures

Patients can decline to continue receiving IP and/or other protocol-required therapies or procedures at any time during the trial but continue participation in the trial. If this occurs, the investigator is to discuss with the patient the appropriate processes for discontinuation from IP or other protocol-required therapies and must discuss with the patient the options for continuation of the SoA (Section 1.3) including different options of follow-up (eg, in person, by phone/mail, through family/friends, in correspondence/communication with other treatment physicians, from the review of medical records) and collection of data, including endpoints and AEs. Patients who have discontinued IP and/or protocol required therapies or procedures should not be automatically removed from the trial. Whenever safe and feasible it is imperative that patients remain on-trial to ensure safety surveillance and/or collection of outcome data. The investigator must document the change to the SoA (Section 1.3) and the level of follow-up that is agreed to by the patient (eg, in person, by telephone/mail, through family/friends, in correspondence/communication with other physicians, from review of the medical records).

7.4. Patient Consent Withdrawal

Patients have the right to withdraw consent and no longer participate in the trial at any time and for any reason without prejudice to their future medical care by the physician or at the institution. Consent withdrawal means the patient no longer wishes to undergo any follow-up visits, trial procedures, investigator contact, and non-patient contact follow-up (eg, medical records check).

- Discontinuing IP should be distinguished from consent withdrawal for follow-up since the patient may agree to undergo trial procedures or still be contacted even though they have stopped taking IP (see Section 7.3).
- Consent withdrawal should be accompanied by documentation of the reason for withdrawal. Patients requesting consent withdrawal for any follow-up should be informed that it may limit the public health value of the trial.

Patients who withdraw consent should be asked explicitly about the contribution of possible AEs to their decision to withdraw consent, and any AE information elicited should be documented.

Preferably, the patient should withdraw consent in writing and, if the patient or the patient's representative refuses or is physically unable, the site should document and sign the reason for the patient's failure to withdraw consent in writing. The ICF for the trial may note that although a patient is completely free to leave the trial and stop taking IP, the investigators hope the patient will remain for follow-up status evaluations.

For patients who have withdrawn consent for further follow-up, investigators may review public records as permitted by applicable law to determine vital status of the patient before or at the end of the trial.

7.5. Lost to Follow up

A patient will be considered lost to follow-up if he or she repeatedly fails to return for scheduled visits and is unable to be contacted by the site.

The following actions must be taken if a patient fails to return to the clinic for a required trial visit:

- The site must attempt to contact the patient or the patient's family and reschedule the missed visit as soon as possible and counsel the patient on the importance of maintaining the assigned visit schedule and ascertain whether the patient wishes to and/or should continue in the trial.
- Before a patient is deemed lost to follow up, the investigator or designee must make every effort to regain contact with the patient or the patient's family (where possible, 3 telephone calls and, if necessary, a certified letter to the patient's last known mailing address or local equivalent methods). These contact attempts should be documented in the patient's medical record.
- Should the patient continue to be unreachable, he/she will be considered to have discontinued from the trial and is lost to follow-up.

Closing of specific sites or discontinuation of the trial are handled as part of Appendix 1 (Section 10.1.8).

8. TRIAL ASSESSMENTS AND PROCEDURES

Trial assessments and procedures and their timing are summarized in the SoA (Section 1.3). Protocol waivers or exceptions are not allowed. Adherence to the trial design requirements, including those specified in the SoA, is essential and required for trial conduct.

There will be a total of 11 in-person trial visits per patient, with windows to aid in scheduling as shown in Table 5. If a patient visit must be scheduled outside the visit window, the Medical Monitor should be contacted.

Table 5: CY 6031 Visit Windows

Visit	Visit Window
Screening	Up to 42 days prior to Day 1 visit
Day 1 (First Dosing Day)	N/A
Week 2	+3 days
Week 4	+3 days
Week 6	+3 days
Week 8	+3 days
Week 12	±3 days
Week 16	±7 days
Week 20	±7 days
Week 24 (EOT)	±7 days
Week 28 (EOS)	+7 days

All visits should be scheduled based on Day 1

8.1. Visit Schedule

Activities will be completed during clinic visits as described in this section. General guidance for the preferred order of assessments and procedures are outlined below:

- On visit days, patients should wait to take their daily IP dose at the clinic.
- Patient-reported outcomes questionnaires should be completed by patients prior to any other activities. (ie, KCCQ (administered first), EQ-5D-5L, SAQ-7, Patient Global Impression of Change [PGI-C])
- IP should be administered after completion of vital signs, ECG, and blood draws.
- ECGs and vital signs must be performed prior to blood draws or other invasive procedures.
- Echocardiograms should be done prior to IP dosing on Day 1 and 2 hours after IP dosing in the clinic at other time points.
- CPET should be performed after other visit activities including IP administration. CMR should occur after CPET if the assessments are performed on the same day.

Please refer to the study manuals for additional details.

8.1.1. Screening Visit

All screening evaluations must be completed and reviewed to confirm that potential patients meet all eligibility criteria. The investigator will maintain a screening log to record details of all patients screened and to confirm eligibility or record reasons for screening failure, as applicable.

Laboratory values obtained at screening and reported through the central laboratory will be used to determine patient eligibility.

The screening period will be up to 6 weeks in duration to allow greater flexibility for visit scheduling and the potential for retesting.

The CPET should not be completed until the patient is otherwise deemed eligible. The CPET used for eligibility should be completed within four weeks but not less than one week prior to randomization.

8.1.2. Day 1

Patients who meet all eligibility criteria including confirmation from the CPET core laboratory at the end of the screening period will return to the site for randomization and Day 1 activities as defined in the SoA.

8.1.3. Weeks 2 through 20

Please refer to the SoA (Section 1.3) for Weeks 2-20.

8.1.4. Week 24: End of Treatment Visit

Week 24 is defined as the EOT visit and includes assessments and procedures critical for analysis of the trial's endpoints. These assessments are outlined in the Schedule of Activities. Additionally, the following should be considered in advance of Week 24:

- Contact patients shortly before the Week 24 EOT visit to confirm their ability to perform the CPET, and schedule the procedure to ensure it is completed within the protocol-defined window
- For patients temporarily unable to exercise on the treadmill or bicycle (whichever modality was used at baseline) due to an adverse event (eg, ankle sprain, upper respiratory infection, migraine), but not due to HCM symptoms, or if the site is unable to perform the CPET (eg, equipment malfunction), then the Week 24 visit may be postponed by up to 4 weeks and those patients should continue to receive IP until the visit
- Schedule CMR imaging for sub-study (if applicable)
  - For patients who provided consent and completed baseline imaging at screening, schedule the CMR imaging to ensure it is completed within the protocol-defined window. The baseline CMR should be performed within the 6-week screening period prior to randomization.

  - The EOT CMR should be performed after the Week 20 visit, but prior to the last dose of IP at Week 24.

- If necessary, the Week 24 visit may be split across two consecutive days within the visit window. If the visit is split, all assessments, except the CPET, should occur on the first day of the split visit. The CPET should occur on the second day of the split visit. Dosing will occur on site on both split visit days.

8.1.5. Week 28: End of Study Visit

All patients should complete an end of study (EOS) visit:

- For subjects who complete all study visits, the EOS visit will occur at Week 28 (or 4 weeks after a delayed Week 24 visit).
- For subjects who early-terminate IP more than 4 weeks before Week 24 and continue to stay on-study for all follow-up assessments, the Week 24 visit can be considered the EOS visit (and the EOS visit does not need to be performed).
- For subjects who withdraw consent and do not wish to continue the participation in follow-up assessments, an EOS visit should be performed 4 weeks after their final IP dose if possible.

To ensure a 4-week safety follow-up (to assess any Adverse Events), the EOS Exit Date must correspond to either 4 weeks after last dose, or at their last study visit (ie, Week 24), whichever is later.

Screening assessments for entry into an open-label extension study of aficamten are permitted at the EOS visit. CY 6031 EOS visit assessments must occur before the open-label screening assessments. Assessments for each visit must be done in the order defined by the protocol.

8.1.6. Early Discontinuation Visit

For patients who discontinue the trial prematurely, the activities outlined in the SoA will be completed during an Early Discontinuation visit as soon as possible.

8.1.7. Unscheduled Visit

Assessments may be completed at the investigator’s discretion during an Unscheduled visit. In addition, assessments performed at a scheduled visit that are not defined in the SOA will be considered unscheduled assessments.

8.2. Efficacy Assessments

8.2.1. Cardiopulmonary Exercise Testing

All patients will undergo CPET with gas-exchange analysis and the methodology will be standardized across all participating sites, as described in the CPET manual. Testing will include continuous ECG monitoring by trained personnel and be performed in an area that is equipped for cardiopulmonary resuscitation. Treadmill is the preferred modality for exercise testing. For CPET laboratories that do not perform treadmill testing, cycle ergometry is an acceptable alternative. Exercise protocols for both modalities will be provided in the CPET manual. Patients

must use the same testing modality for all exercise tests during the trial. Whenever possible, CPET should be administered by the same trial personnel using the same equipment and performed after the other trial procedures on that visit day (including echocardiogram, KCCQ, EQ-5D-5L, CGI, PGI-C, NYHA class, SAQ-7, vital signs, ECG, blood sampling, IP administration). Patients naïve to exercise protocols will be familiarized with the technique during screening.

All CPET testing will be symptom-limited and patients will be strongly encouraged to achieve maximal exertion and an RER ≥1.05. The reason(s) for termination of sub-maximal exercise tests will be documented. A test will be identified as being maximal effort if the RER is ≥1.05.

Patients should not engage in strenuous exercise for 24 hours prior to the CPET, and patients should not exercise at all within 12 hours prior to the test. Patients should fast for at least 4 hours prior to CPET. All regularly scheduled medications should be taken as normal. Patients should avoid taking medications that cause drowsiness within 8 hours prior to the test. Weight should be collected immediately prior to each CPET.

The Week 24 CPET should be performed at approximately the same time of day (eg, morning, mid-day, afternoon) as the baseline CPET at screening, at a consistent time after the last dose of beta-blocker and IP. Whenever possible, patients should perform exercise testing between three and ten hours after taking beta blocking agents.

If a life-threatening arrhythmia, early ischemia, severe hypotension or other serious finding is identified by the investigator during CPET, the patient will be asked to stop the exercise test, and his/her physicians will be notified of the results. If the patient is performing the screening test, s/he will not be randomized to the trial. Enrolled patients who have a non-life-threatening event or finding that stops the test can resume testing when it is safe to do so and after appropriate treatment, per the investigator.

All sites must be qualified by the CPET core laboratory prior to the initiation of screening. To qualify, sites will perform an exercise test on two healthy adults and submit them for core laboratory review. Sites may be required to submit additional normal exercise tests during the conduct of the trial for review by the CPET core laboratory in order to confirm proper function of testing equipment. Sites may be qualified based on exercise tests recently reviewed by the CPET core laboratory during the conduct of other trials.

8.2.2. Echocardiography

Echocardiography will be done during screening and prior to dosing on Day 1. Echocardiography will be performed 2 hours (±30 min) after dosing in the clinic on Weeks 2, 4, 6, 8, 12, 16, 20, and 24. Echocardiography will also be performed at Week 28.

Certified sonographers will perform echocardiography using standard high-quality, high-fidelity machines approved by Cytokinetics. Whenever possible, the same sonographer will perform all studies for a single patient. Echocardiograms will be performed after the patient has been resting in a supine position for at least 10 minutes and in accordance with the echocardiography manual. Instructions for the performance of the Valsalva maneuver and imaging the LVOT-G will also be included in the echocardiography manual.

When echocardiograms are scheduled at the same time as blood draws, vital signs, and/or ECGs, the order of evaluation will be vital signs, ECGs, blood draw and echocardiogram. The blood draw should be obtained at the scheduled time point and the echocardiograms will follow.

Echocardiographic parameters to be measured will at least include the left ventricular parameters in Table 6 in addition to right heart function metrics detailed in the echocardiography protocol.

Table 6: CY 6031 Echocardiographic LV Parameters to be Measured

Resting LVOT-G	LVEDV	IVST
Post-Valsalva LVOT-G	LVESD	IVCT
LVEF	LVESV	IVRT
LVFS	LVCO	E/E' ratio (septal and lateral)
GLS	LV Stroke Volume	LAV
LVEDD	LVOT VTI	

GLS = global longitudinal strain; IVCT = isovolumic contraction time; IVRT = isovolumic relaxation time; IVST = interventricular septum thickness; LAV = left atrial volume; LVCO = left ventricular cardiac output; LVEDD = left ventricular end diastolic diameter; LVEDV = left ventricular end diastolic volume; LVESD = left ventricular end systolic diameter; LVESV = left ventricular end systolic volume; VTI = velocity time integral.

Unscheduled echocardiograms may be obtained when clinically indicated, for example to assess an AE or follow-up a clinically significant change in a prior echocardiogram, as determined by the investigator. Results will be interpreted by the unmasked echocardiologist at the investigational site.

All echocardiograms (including unscheduled) will be sent to the core laboratory for interpretation. On-site interpretation of LVEF and LVOT-G will be used for dose escalation and reduction decisions via IWRS. The core laboratory quantification of the echocardiograms will be used for all statistical analyses.

8.2.3. Cardiac Magnetic Resonance

A CMR imaging sub-study will assess the effects of administration of CK-3773274 dosing on cardiac morphology, function, and fibrosis in approximately 100 oHCM patients who are eligible and consent to participate. CMR will be performed during screening period and Week 24. CMR should occur after CPET if the assessments are performed on the same day.

Patients with eGFR <30 mL/min/1.73 m^2 or an allergy to gadolinium may have a non-contrast CMR.

Patients who screen fail and rescreen do not need a repeat CMR.

8.2.4. New York Heart Association Functional Classification

After interviewing the patient, the investigator (or qualified designee) will record the NYHA Functional Classification in the CRF (Criteria Committee of the New York Heart 1994). The NYHA classification is as follows:

- Class I - No symptoms and no limitation in ordinary physical activity (eg, shortness of breath when walking, climbing stairs)

- Class II - Mild symptoms (eg, mild shortness of breath and/or angina) and slight limitation during ordinary activity.
- Class III - Marked limitation in activity due to symptoms, even during less-than-ordinary activity (eg, walking short distances [20-100 m]). Comfortable only at rest.
- Class IV - Severe limitations. Experiences symptoms even while at rest. Mostly bedbound patients.

8.2.5. Clinical Global Impression Scale

The investigator (or qualified designee) will record the CGI scale assessment of the patient's global functioning in the CRF at the time points outlined in the SoA.

8.2.6. Patient-Reported Outcomes

The following questionnaires will be completed at trial visits specified in the SoA (Section 1.3):

- KCCQ
- EQ-5D-5L
- PGI-C
- SAQ-7

Patients will be asked to complete the KCCQ, EQ-5D-5L, SAQ-7 and PGI-C questionnaires in a quiet place prior to the medical consultation and prior to undergoing any tests and procedures to avoid biasing their responses.

Site staff will verify the questionnaires for completeness before the patients leave the clinic or hospital.

8.3. Safety Assessments

Planned time points for all safety assessments are provided in the SoA (Section 1.3).

8.3.1. Physical Examinations

A complete physical examination will include, at a minimum, assessments of the cardiovascular, respiratory, and neurological systems. Breast, genital, and rectal examinations are not required unless specific evaluation is warranted.

Investigators should pay special attention to clinical signs related to previous serious illnesses.

Physical examinations may be conducted at any time during the treatment period if clinically indicated.

8.3.2. Height and Weight

Height and weight will be measured while patient is fully clothed with shoes removed. Height will be measured at screening only.

8.3.3. Vital Signs

At the Screening visit, a room air oxygen saturation will be assessed.

At all visits, heart rate, respiratory rate, and blood pressure will be assessed.

Blood pressure and heart rate measurements will be assessed with the patient in a supine or sitting position. Blood pressure and heart rate measurements should be performed with an automated oscillometer after the patient has rested for at least 5 minutes in a quiet setting without distractions (eg, television, cell phones). The position selected for a patient should be the same that is used throughout the trial.

Vital signs (to be taken before blood collection for laboratory tests) will consist of 1 heart rate reading and 3 consecutive blood pressure readings recorded at intervals of at least 1 minute. The average of the 3 blood pressure readings will be recorded in the CRF.

8.3.4. Electrocardiograms

Triplicate 12-lead ECGs will be obtained as outlined in the SoA (Section 1.3) using an ECG machine that automatically calculates the heart rate and measures PR, QRS, QT, and QTc intervals.

At each time point at which triplicate ECGs are performed, three individual ECG tracings should be obtained as closely as possible in succession, but no more than 2 minutes apart.

Patients should be sitting or supine in a rested and calm state for at least 5 minutes prior to the ECG. The investigator may perform additional ECG recordings as needed for the care of the patient.

A patient will be withdrawn from the trial by the investigator or designee if, in their medical judgment, ECG findings are present which make continued trial participation not in the patient's best interest.

For safety monitoring purposes, the investigator or designee must review, sign, and date all ECG tracings.

Unscheduled ECGs may be collected at additional time points, for example in case of an AE or based on vital signs, PK results or PD results, as determined by the investigator or the Medical Monitor.

All ECG tracings will be kept as part of the patient's permanent trial file at the site. Digital recordings will be analyzed and stored at a central ECG laboratory.

8.3.5. Laboratory Assessments

See Appendix 2 (Section 10.2) for the list of clinical laboratory tests to be performed and see the SoA (Section 1.3) for the timing and frequency.

Repeat or unscheduled samples may be taken for safety reasons or for technical issues with the samples.

The investigator must review the laboratory report, document this review, and record any clinically significant changes occurring during the trial in the AE section of the CRF. Clinically significant abnormal laboratory findings are those which are not associated with the underlying

disease, unless judged by the investigator to be more severe than expected for the patient's condition.

All protocol-required laboratory assessments, as defined in Appendix 2 (Section 10.2), must be conducted in accordance with the laboratory manual and the SoA (Section 1.3).

8.4. Adverse Events and Serious Adverse Events

8.4.1. Adverse Events

8.4.1.1. Definition of Adverse Event

An **adverse event (AE)** is defined as any untoward medical occurrence in a patient or clinical investigation patient administered a pharmaceutical product and which does not necessarily have a causal relationship with this treatment. An AE can therefore be any unfavorable and unintended sign (including an abnormal laboratory finding), symptom, or disease temporally associated with the use of IP, whether or not related to the IP.

Adverse events include:

- Exacerbation of a chronic or intermittent pre-existing condition including either an increase in frequency and/or intensity of the condition.
- New conditions detected or diagnosed after IP administration even though it may have been present before the start of the trial.
- Abnormal assessments, eg, change on physical examination, ECG findings, if they represent a clinically significant finding that was not present at trial start or worsened during the course of the trial.
- Laboratory test abnormalities if they represent a clinically significant finding, symptomatic or not, which was not present at trial start or worsened during the course of the trial, require treatment or led to dose reduction, interruption or permanent discontinuation of IP. In general, abnormal laboratory findings without clinical significance (based on the investigator's judgment) are not to be recorded as adverse events. Where applicable, clinical sequelae (not the laboratory abnormality) are to be recorded as the adverse event.
- Signs, symptoms, or the clinical sequelae of a suspected drug-drug interaction.
- Signs, symptoms, or the clinical sequelae of a suspected overdose of either IP or a concomitant medication. Overdose per se will not be reported as an AE/SAE unless it is an intentional overdose taken with possible suicidal/self-harming intent. Such overdoses should be reported regardless of sequelae.

8.4.1.2. Definition of Serious Adverse Event

A **serious adverse event** (SAE) is defined as any untoward medical occurrence that at any dose:

- results in death,
- Is life threatening, NOTE: The term "life-threatening" in the definition of "serious" refers to an event in which the patient was at risk of death at the time of the event; it

does not refer to an event which hypothetically might have caused death if it were more severe

- Requires inpatient hospitalization or prolongation of existing hospitalization
- Results in persistent or significant disability/incapacity, or
- Is a congenital anomaly/birth defect
- Important medical event

Medical and scientific judgement should be exercised in deciding whether expedited reporting is appropriate in other situations, such as important medical events that may not be immediately life-threatening or result in death or hospitalization but may jeopardize the patient or may require intervention to prevent one of the other outcomes listed in the definition above. These should also usually be considered serious. Examples of such events include invasive or malignant cancers, intensive treatment in an emergency room or at home for allergic bronchospasm; blood dyscrasias or convulsions that do not result in hospitalization; or development of drug dependency or drug abuse.

An adverse event would meet the criterion of “requires hospitalization,” if the event necessitated an admission to a health care facility (eg, overnight stay).

The following reasons for hospitalization are exempted from being reported:

- Hospitalization for cosmetic elective surgery, or social and/or convenience reasons.
- Hospitalization for pre-planned (ie, planned prior to signing informed consent) surgery or standard monitoring of a pre-existing disease or medical condition that did not worsen, eg, hospitalization for coronary angiography in a patient with stable angina pectoris.

However, complications that occur during an exempted hospitalization are AEs or SAEs (for example if a complication prolongs a pre-planned hospitalization).

8.4.1.3. Intensity of Adverse Events

The investigator must assess the intensity for each AE and SAE reported during the trial according to a three-point scale: mild, moderate, severe.

If the intensity of an AE worsens during IP administration, only the worst intensity should be reported on the AE page. If the AE lessens in intensity, no change in the severity is required.

The three categories of intensity are defined as follows:

**Mild**	The event is noticeable to the patient. It does not influence daily activities and does not require intervention.
**Moderate**	The event makes the patient uncomfortable. Performance of daily activities are influenced, and intervention is needed.

**Severe**	The event causes noticeable discomfort and interferes with daily activities. The patient may not be able to continue in the trial, and treatment or intervention is needed.

A mild, moderate, or severe AE may or may not be serious. Medical judgment should be used on a case-by-case basis.

Seriousness, rather than severity assessment, determines the regulatory reporting obligations.

8.4.1.4. Relationship to Investigational Product

Each AE must be assessed by the investigator, based on clinical judgment, as to whether or not there is a reasonable possibility of causal relationship to the IP and reported as either related or unrelated.

- A "reasonable possibility" of a relationship conveys that there are facts, evidence, and/or arguments to suggest a causal relationship, rather than a relationship cannot be ruled out.
- Alternative causes, such as underlying disease(s), concomitant therapy, and other risk factors, as well as the temporal relationship of the event to IP administration will be considered and investigated.
- For each AE/SAE, the investigator must document in the medical notes that he/she has reviewed the AE/SAE and has provided an assessment of causality.
- There may be situations in which an SAE has occurred and the investigator has minimal information to include in the initial report to Cytokinetics. However, it is very important that the investigator always assesses causality for every event before the initial transmission of the SAE data to Cytokinetics.
- The investigator may change his/her opinion of causality considering follow-up information and send an SAE follow-up report with the updated causality assessment.

8.4.1.5. Relationship to Trial Procedures

An AE is defined as related to trial procedures if it appears to have a reasonable possibility of a causal relationship to protocol-required procedures.

8.4.1.6. Reporting of AEs

The investigator is responsible for ensuring that all SAEs and non-serious AEs observed by the investigator or reported by the patient that occur after starting the IP through study exit are recorded in the AE eCRF.

Only SAEs and non-serious AEs considered related to trial procedures are reported after signing of the informed consent until IP administration.

Medical occurrences that are not associated with trial procedures and that begin before the start of IP but after signing of the ICF will be recorded on the Medical History/Current Medical Conditions CRF, not the AE CRF.

8.4.1.7. Reporting Procedures for SAEs

Prompt notification by the investigator to Cytokinetics of an SAE is essential so that legal obligations and ethical responsibilities towards the safety of patients and the safety of an IP under clinical investigation are met.

The investigator is responsible for ensuring that all SAEs observed by the investigator or reported by the patient that occur after starting the IP through end of study, or 4 weeks after the last administration of IP, whichever is later, are reported to Cytokinetics on an SAE Report Form within 24 hours following the investigator's knowledge of the event and recorded in the AE eCRF. These events must be reported regardless of the investigator-attributed causal relationship with IP.

The SAE Report forms must be emailed or faxed to Cytokinetics Drug Safety (contact details are provided on the SAE Report form):

Email: CY6031DrugSafety@cytokinetics.com

Facsimile: +1 (650) 243-4199

The investigator must attempt to establish a diagnosis of the event based on signs, symptoms, and/or other clinical information. Whenever possible, the diagnosis (not the individual signs/symptoms) will be documented as the AE/SAE.

The investigator must complete the SAE Report form in English and must assess the causal relationship of the event to IP.

If the patient is hospitalized in a hospital other than the trial site, it is the investigator's responsibility to contact this hospital to obtain all SAE relevant information and documentation.

New information relating to a previously reported SAE must be reported to Cytokinetics within 24 hours following knowledge of the new information. Cytokinetics Drug Safety may contact the investigator to obtain further information.

8.4.1.8. Follow-up of AEs and SAEs

The investigator is obligated to perform or arrange for the conduct of supplemental measurements and/or evaluations as medically indicated or as requested by Cytokinetics to elucidate the nature and/or causality of the AE or SAE as fully as possible. This may include additional laboratory tests or investigations, histopathological examinations, or consultation with other health care professionals.

If a patient dies during participation in the trial or during a protocol-defined follow-up period, the investigator will provide Cytokinetics with a copy of any post-mortem findings including histopathology if it has been performed.

Non-serious AEs must be followed until they resolve or until the patient completes the trial, whichever comes first.

Serious AEs still ongoing at the end of study must be followed up until resolution or stabilization, or until the event outcome is provided, eg, death. Reporting after study exit to Drug Safety may continue after the EOS visit.

New SAEs occurring after the 4-week follow-up period must be reported to the Cytokinetics drug safety department within 24 hours of the investigator's knowledge of the event, **only** if considered by the investigator to be causally related to previous exposure to the IP.

8.4.1.9. Regulatory Reporting

The reference safety document used for the assessment of expectedness of a suspected serious adverse reaction for the purpose of expedited reporting to Health Authorities, IRBs/IECs, and investigators is the reference safety information section of the Investigator's Brochure [CK-3773274 IB].

Cytokinetics will report SAEs and/or suspected unexpected serious adverse reactions as required to regulatory authorities, investigators/institutions, and IRBs/IECs in compliance with all reporting requirements according to local regulations and good clinical practice.

The investigator is to notify the appropriate IRB/IEC of SAEs occurring at the site and other AE reports received from Cytokinetics, in accordance with local procedures and statutes.

8.4.1.10. Pregnancy and Breastfeeding

If a woman becomes pregnant while on IP, IP must be discontinued. The investigator must counsel the patient and discuss the risks of continuing with the pregnancy and the possible effects on the fetus.

Please refer to Appendix 3 (Section 10.3) regarding contraceptive guidance.

Irrespective of the treatment received by the patient, any pregnancy occurring in a female patient, or female partner of a male patient, after starting the IP up to 4 weeks following IP discontinuation must be reported to Cytokinetics within 24 hours of the investigator's knowledge of the event.

Pregnancies must be recorded in the eCRF and reported on the Cytokinetics Pregnancy form, which is emailed or faxed to Cytokinetics Drug Safety (contact details are provided on the Pregnancy Report form):

Email: **CY6031DrugSafety@cytokinetics.com**

Facsimile: **+1 (650) 243-4199**

Details of all pregnancies in female patients and female partners of male patients will be collected after the start of IP and until the conclusion of the pregnancy. The follow-up of an infant (if applicable) will be conducted up to 12 months after the birth of the child.

Any pregnancy complication or elective termination of a pregnancy for medical reasons must be reported as an AE or SAE.

Abnormal pregnancy outcomes (eg, spontaneous abortion, fetal death, stillbirth, congenital anomalies, ectopic pregnancy) are considered SAEs.

Any post-trial pregnancy-related SAE considered reasonably related to the IP by the investigator will be reported to Cytokinetics as described in Section 8. While the investigator is not obligated to actively seek this information in former trial patients, he or she may learn of an SAE through spontaneous reporting.

Male Patients with Partners Who Become Pregnant

If the partner of a male subject becomes pregnant while on study drug, he may continue receiving treatment; however, he must use barrier method (ie, condom) during sexual intercourse to avoid further fetal exposure.

The investigator will attempt to collect pregnancy information on any male patient's female partner who becomes pregnant while the male patient is in this trial.

After obtaining the necessary signed ICF from the pregnant female partner directly, the investigator must complete the Pregnancy Report Form and submit it to Cytokinetics within 24 hours of receipt of the partner's consent. The female partner will also be followed to determine the outcome of the pregnancy. Information on the status of the mother and child will be forwarded to Cytokinetics.

Female Patients Who Breastfeed

If a female patient breastfeeds while on study drug, study drug will be discontinued.

The investigator will collect breastfeeding information on any female patient who breastfeeds while taking the IP through one month after the end of study drug treatment. The mother and infant health information will be recorded on the Pregnancy Report Form and submitted to Cytokinetics immediately and no later than 24 hours following the investigator's knowledge of event.

8.5. Treatment of Overdose

For this trial, any dose of IP that exceeds the protocol-specified dose or dosing frequency will be considered an overdose.

There is no established treatment for an overdose. In the event of overdose, monitor for signs and symptoms including but not limited to hypotension, cardiac dysrhythmia, tachycardia, tachypnea, peripheral and pulmonary edema, decrease renal function, dizziness, dyspnea, palpitation, fatigue. The use of rescue medications (eg, dobutamine) to treat a low cardiac output state is recommended if necessary.

If a patient experiences low cardiac output due to systolic dysfunction, the investigator should follow appropriate regional heart failure treatment guidelines.

The date and time of rescue medication administration as well as the name and dosage regimen of the rescue medication must be recorded.

In the event of an overdose, the investigator should:

- Contact the Medical Monitor immediately who may recommend:
    a. Close monitoring of the patient for any AEs/SAEs and laboratory abnormalities.
    b. Obtaining a plasma sample for PK analysis as soon as practical and note the date of the last dose of IP.
- Document the quantity of the excess dose as well as the duration of the overdose in the CRF.

Decisions regarding dose interruptions or modifications will be made by the investigator in consultation with the Medical Monitor based on the clinical evaluation of the patient.

8.6. Pharmacokinetics

Eighteen blood samples of approximately 4 mL will be collected for measurement of plasma concentrations of CK-3773274 as specified in the SoA (Section 1.3) and Table 7 below. Samples will be used to evaluate the PK of CK-3773274 and potentially its metabolites. Instructions for the collection and handling of biological samples will be provided in the laboratory manual.

The actual date and time (24-hour clock time) of each sample will be recorded. The time of administration of IP on the day of PK sampling will be recorded in the CRF. It is important to provide instructions to patients that they should not take their dose on the day of their clinic visit until in the clinic.

See Table 7 for a summary of PK sampling time points. All samples should be drawn within ±10 minutes of the scheduled time point. Drug concentration information that would unblind the trial will not be reported to investigative sites or blinded personnel until the trial has been unblinded.

Table 7: Summary of PK Time Points

Visit	PK Time Point
Day 1	Pre-dose and 2 hours post-dose
Week 2	Pre-dose and 2 hours post-dose
Week 4	Pre-dose and 2 hours post-dose
Week 6	Pre-dose and 2 hours post-dose
Week 8	Pre-dose and 2 hours post-dose
Week 12	Pre-dose and 2 hours post-dose
Week 16	Pre-dose and 2 hours post-dose
Week 20	Pre-dose and 2 hours post-dose
Week 24 (EOT)	Pre-dose and 2 hours post-dose
Early Discontinuation	untimed

8.7. Genetics

As HCM is a genetic disease, blood and/or DNA from patients who consent may be analyzed through the use of both clinically reportable testing (Clinical Laboratory Improvement Amendments (CLIA) certified laboratory), and non-clinically reportable (non-CLIA certified laboratory) whole genome sequencing, whole exome sequencing, next-generation sequencing, and/or other method to identify genetic variants and mutations that are predictive of patient phenotype, response to IP, resistance to IP, metabolism of IP, susceptibility to developing AEs, or to increase the knowledge and understanding of cardiovascular, muscle and disease biology.

If a participant consents, and where local regulations and IRB/IEC allow, a blood sample will be collected for DNA analysis from participants consenting to provide the sample. In the event of

DNA extraction failure, a replacement genetic blood sample may be requested from the patient. CLIA certified genetic test results may be made available to the investigator.

See Appendix 4 (Section 10.4) for information regarding genetic research. Details on processes for collection and shipment and destruction of these samples can be found in the laboratory manual.

8.8. Serum for Biomarker Analysis

Blood will be collected for analysis of serum biomarkers. See Section 10.2 for list of biomarkers.

8.9. Serum and Plasma Collection for Future Analyses

For participants who provide consent, serum, plasma, and DNA samples (as described in Section 8.7) will be collected and banked in this trial for future research on biomarkers and genetics. Serum, plasma, and DNA samples will be stored in a long-term storage facility designated by Cytokinetics for up to 20 years.

8.10. Immunogenicity Assessments

No immunogenicity assessments will be done for this trial.

9. STATISTICAL CONSIDERATIONS

9.1. Statistical Hypotheses

The analyses evaluating treatment effect on the primary and secondary efficacy endpoints will test the null hypothesis that there is no treatment difference between patients receiving placebo and those receiving CK-3773274 in the full analysis set (FAS). Adjustments for multiplicity will be specified in Section 9.4.1.1.

9.2. Sample Size Determination

Assuming a difference in change from baseline in pVO_2 of 1.5 mL/kg/min for CK-3773274 compared to placebo, a standard deviation (SD) of 3.5 mL/kg/min, accounting for limiting beta-blocker use (less than ~70%), limiting exercise modality of bicycle (less than ~50%) and 10% of patients missing change from baseline data of the primary endpoint, a sample size of 270 patients at randomization ratio of 1:1 (approximately 135 randomized to CK-3773274 and 135 randomized to placebo) provides more than 90% power to detect the difference in pVO_2 change from baseline to Week 24 with a 2-sided type I error of 0.05.

During the study, the aggregate pooled missing data rate and overall pooled SD for the change from baseline in pVO_2 at Week 24 will be monitored periodically in a blinded fashion. If the pooled SD is larger than expected, Cytokinetics may consider increasing the sample size once in order to maintain the intended power.

9.3. Populations for Analyses

The analysis populations are defined in Table 8.

Table 8: Analysis Sets

Analysis Set	Description
All Randomized Set	All randomized patients.
Full Analysis Set	All randomized patients. Patients will be analyzed according to their randomized treatment group assignment. Efficacy endpoints will be analyzed based on the FAS.
Safety Analysis Set	All randomized patients who received at least one dose of IP, CK-3773274 or placebo. Patients will be analyzed by their randomized treatment group assignment. If a patient receives treatment throughout the study that is different than the randomized treatment group assignment, then this patient will be grouped by the actual treatment group
Pharmacokinetics Analysis Set (PKS)	All randomized patients who have at least one evaluable plasma concentration of CK-3773274, provided they have no major protocol violations deviations that could affect the PK of CK-3773274.

9.4. Statistical Analyses

The Statistical Analysis Plan (SAP) will be finalized prior to database lock and it will include a more technical and detailed description of the statistical analyses described in this section. This section is a summary of the planned statistical analyses of the most important endpoints including primary and key secondary endpoints.

9.4.1. General Considerations

Summary tables will present descriptive statistics such as number of patients, mean, median, standard deviation, minimum and maximum for continuous variables, and number of patients and the percentage for categorical variables, overall and by treatment in the planned analysis sets. For model-based analysis, least squares means (LSMs), difference of LSMs between treatments, their standard errors and 95% confidence intervals (CI), and two-sided p-values for the relative statistical inferences will be presented. Baseline is defined as the last available measurement taken before the first dose of randomized IP unless otherwise specified. Listings will include patient ID, demographics, treatment assigned and other relevant items, and sorted by treatment assignment, patient ID and date of assessment. Unless specified otherwise, efficacy, safety and pharmacokinetics analyses will be performed on the full analysis set, safety analysis set and pharmacokinetics analysis set, respectively. Statistical analysis methods will be detailed in the SAP.

9.4.1.1. Multiplicity Adjustment

The null hypothesis for the primary and secondary efficacy variables in the FAS will be tested in the pre-specified order using a closed testing procedure.

For preservation of the overall type I error rate at two-sided 0.05 for the primary and secondary endpoints will be tested in the following specified order using a closed testing procedure . If the primary endpoint achieves statistical significance at two-sided $p \leq 0.05$, then secondary endpoints will be tested with two-sided 0.05, with their testing being in the sequential order of KCCQ-CSS change from baseline, proportion of patients with ≥1 NYHA functional class improvement, post-Valsalva LVOT-G change from baseline, proportion of patients with post-Valsalva LVOT-G < 30 mm Hg, and duration of SRT eligibility for participants who are SRT eligible at baseline, for each after 24 weeks of treatment; then KCCQ-CSS change from baseline, proportion of patients with ≥1 NYHA functional class improvement, post-Valsalva LVOT-G change from baseline, and proportion of patients with post-Valsalva LVOT-G < 30 mmHg, for each after 12 weeks of treatment; and lastly change from baseline to Week 24 in total workload. SRT eligibility is defined as resting or post-Valsalva LVOT-G ≥ 50 mmHg AND NYHA Functional Class ≥ 3. The multiple testing procedure is illustrated in Figure 1. The description of testing sequence will be detailed in the SAP.

9.4.2. Primary Endpoint(s)

The primary endpoint of the study is change in pVO_2 by CPET from baseline to Week 24.

The primary estimand is the difference in means of the change from baseline to Week 24 in pVO_2 between CK-3773274 and placebo for the target population of potentially treatable CK-3773274 patients despite intercurrent events after a first dose. Missing data will be imputed using multiple imputation method under the missing at random (MAR) assumption. The

distribution of missing CPET data at Week 24 and the reasons for the missing data will be tabulated in the FAS. The primary analysis of the primary endpoint will use an ANCOVA model with treatment group, randomization stratification factors, baseline pVO_2 and baseline weight as covariates in the FAS. Sensitivity analyses will be performed by repeating the primary analysis examining assumptions that data are not missing at random: missing pVO_2 from patients who discontinued CK-3773274 treatment or missing pVO_2 from patients from the placebo arm will be imputed based on the model that is constructed using observed pVO_2 data from the placebo arm. Missing pVO_2 from patients who remained on CK-3773274 treatment will be imputed based on the model that is constructed using observed pVO_2 data from the CK-3773274 arm. The LSMs, LSM treatment difference, and the standard error from each imputed dataset will be combined using Rubin's rules to produce an overall LSM estimate of the treatment difference, its 95% confidence interval, and p-value.

Other sensitivity analyses and the details will be included in the SAP.

9.4.3. Secondary Endpoint(s)

The secondary endpoint(s) of the study are:

- Change in KCCQ-CSS from baseline to Week 12 and Week 24
- Proportion of patients with ≥1 class improvement in NYHA Functional Class from baseline to Week 12 and Week 24
- Change in post-Valsalva LVOT-G from baseline to Week 12 and Week 24
- Proportion of patients with post-Valsalva LVOT-G <30 mmHg at Week 12 and Week 24
- Duration of eligibility for septal reduction therapy (SRT) during the 24-week treatment period in patients who were eligible for SRT at baseline. Participants are classified as being SRT eligible if they have NYHA Class ≥3 AND resting or Valsalva LVOT-G ≥50 mmHg.
- Change in total workload during CPET from baseline to Week 24

Change in KCCQ-CSS and change in post-Valsalva LVOT-G from baseline to Week 12 and Week 24 will be analyzed using a mixed model repeated measures (MMRM) model with baseline as covariate, randomization stratification factors, visit, treatment group, and interaction terms of treatment by visit and baseline by visit. An unstructured covariance matrix will be specified.

Proportion of patients with ≥1 class improvement in NYHA Functional Class from baseline to Week 12 and Week 24 or proportion of patients with post-Valsalva LVOT-G <30 mmHg at Week 12 and Week 24 will be analyzed using Cochran– Mantel–Haenszel (CMH) test stratified by randomization factors. Patient's Week 20 NYHA Functional Class will be used when Week 24 NYHA Functional Class is not available. Total duration of SRT eligibility (NYHA Class ≥3 AND resting or Valsalva LVOT-G ≥50 mmHg) during the 24-week treatment period will be analyzed using an ANCOVA model with treatment group and randomization stratification factor beta blocker use/no use as fixed effects adjusting for significant baseline characteristics. The p-value and 95% CI will be obtained using exact method. Adjustment of multiplicity of the primary and secondary endpoints is specified in Section 9.4.1.1.

9.4.4. Exploratory Endpoint(s)

The exploratory endpoints of the study are:

- Compared with baseline, proportion of patients at Week 24 achieving either:
  - Change from baseline of ≥1.5 mL/kg/min in pVO_2 AND ≥1 class improvement in NYHA Functional Class

  OR

  - Change of ≥3.0 mL/kg/min from baseline in pVO_2 AND no worsening of NYHA Functional Class
- Proportion of patients with improvement in KCCQ-CSS ≥5 points at Week 12 and Week 24
- Proportion of patients with resting LVOTG <30 mmHg, post-Valsalva LVOTG <50 mmHg, and NYHA Functional Class I at Week 12 and Week 24
- Proportion of patients with resting LVOTG <30 mmHg, post-Valsalva LVOTG <50 mmHg, and ≥1 class improvement in NYHA Functional Class at Week 12 and Week 24
- Proportion of patients who are eligible for SRT at Week 24 among patients who were eligible for SRT at baseline
- Change from baseline to Week 24 in:
  - VE/VCO_2 slope
  - VO_2 × systolic BP
  - VAT
- Change from baseline to Week 24 in individual responses to the EQ-5D-5L
- Change from baseline to Week 24 in summary and individual domain scores for the SAQ-7
- Change from baseline to Week 24 in echocardiographic measurements of cardiac structure and of systolic function including:
  - LVEF
  - LVESV and LVEDV
  - Left atrial volume
- Change from baseline values in NT-pro-BNP, hs-cardiac-TnI and other biomarkers through Week 24
- Change from baseline to Week 24 in CMR measurements of:
  - LV mass index
  - LVEF

- Septal and free wall thickness
- Left atrial volume index
- LVESV
- LVEDV

- Pharmacokinetic parameters through Week 24

Proportion of responders will be analyzed using CMH test stratified by randomization factors. Change from baseline in continuous echocardiography parameters will be analyzed using MMRM model with baseline as covariate, randomization stratification factors, visit, treatment group and treatment group by visit as interaction. An unstructured covariance matrix will be specified. Change from baseline in other CPET parameters will be analyzed using the same primary model for the primary endpoint. Change from baseline in parameters of CMR measurements and 5Q-5D-5L will be analyzed using an ANCOVA model with baseline as covariate, randomization stratification factors and treatment group as fixed effects. Median and median difference of NT-pro-BNP between treatment group and 95% confidence of the median difference will be presented. Log transformed NT-pro-BNP may be performed and analyzed using MMRM model with log baseline as covariate, visit, randomization stratification factors, treatment group as fixed effects and treatment group by visit interaction. The same model for KCCQ-CSS will be used to analyze the change from baseline in SAQ-7.

9.4.5. Safety Analysis

Safety analyses will be performed on the safety analysis set.

9.4.5.1. Adverse Events

A treatment-emergent AE is an AE with an onset after initiation of IP, or an AE present at initiation of IP dosing that worsens in severity during the treatment. AEs will be coded using MedDRA dictionary and summarized by preferred terms and system organ class. The version of the MedDRA dictionary will be specified in the clinical study report. AEs will be classified according to severity. The number and percentage of patients reporting AEs will be tabulated.

Only treatment-emergent AEs with an onset from the first dose until 4 weeks after last dose of IP will be summarized. All AEs will be included in patient listings.

Patient incidence of reported major adverse cardiac events (CV death, cardiac arrest, non-fatal stroke, non-fatal myocardial infarction, CV hospitalization) will be summarized by treatment group and event type. Patient incidence of new onset persistent atrial fibrillation, patient incidence of appropriate ICD discharges and aborted sudden cardiac death, patient incidence of LVEF <50% will be summarized by treatment group.

9.4.5.2. Serious Adverse Events

Summaries of SAEs (by preferred term and system organ class) and SAE severity will be presented.

The safety follow-up is defined as 4 weeks following the last dose of IP.

9.4.6. Pharmacokinetic Endpoints

Plasma concentrations of CK-3773274 and PK parameter C_{max} and C_{trough} will be summarized using descriptive statistics including mean, standard deviation, geometric mean, coefficient of variation, median, and range. Geometric mean concentrations over time will be graphically displayed.

9.4.7. Patient Disposition

The number of patients who are randomized, who complete the planned treatment, and who prematurely discontinue from the planned treatment and/or the study will be presented by treatment group and overall. Reasons for premature discontinuation as recorded on the End of Study page of the CRF will also be summarized.

9.4.8. Demographics and Other Baseline Characteristics

Patient demographics and other baseline characteristics will be summarized descriptively by treatment group.

9.4.9. Investigational Product Exposure

IP exposure will be summarized, including the total number of doses administered, total amount of drug administered, and the total duration of IP administration, defined as the date of the last dose minus the date of first dose + 1.

9.4.10. Concomitant Medications

Concomitant medications will be summarized and classified by drug class and preferred term using the World Health Organization (WHO) Drug Dictionary. The version of the WHO Drug Dictionary will be specified in the clinical study report.

9.4.11. Clinical Laboratory Parameters

Descriptive statistics for clinical laboratory values and changes from baseline at each protocol specified assessment time point will be presented.

9.4.12. Vital Signs

Descriptive statistics for vital signs and changes from baseline at each protocol specified assessment time point will be presented.

9.4.13. Electrocardiogram

Descriptive statistics for ECG parameters (eg, heart rate, PR interval, QRS interval, QT interval, and QTc interval [both Bazett’s and Fridericia’s corrections]) and changes from baseline at each protocol specified assessment time point will be presented. Select ECG parameters will be analyzed using a repeated measures analyses with dose and time points as factors and baseline ECG parameter as a covariate. Dose-response trend will be estimated.

9.5. Data Monitoring Committee

An unblinded DMC will regularly review the emerging data for safety monitoring purpose. The DMC or Cytokinetics can require an ad hoc DMC meeting at any time. No study activities will be suspended during the safety review. For details on the DMC, refer to Appendix 1 (Section 10.1.5).

10. SUPPORTING DOCUMENTATION AND OPERATIONAL CONSIDERATIONS

10.1. Appendix 1: Regulatory, Ethical, and Trial Oversight Considerations

10.1.1. Regulatory and Ethical Considerations

This trial will be conducted in accordance with the protocol and with the following:

- Consensus ethical principles derived from international guidelines including the Declaration of Helsinki and Council for International Organizations of Medical Sciences (CIOMS) International Ethical Guidelines
- Applicable ICH Good Clinical Practice (GCP) Guidelines
- Applicable laws and regulations

The protocol, protocol amendments, ICF, Investigator's Brochure, and other relevant documents (eg, advertisements) must be submitted to an IRB/IEC by the investigator and reviewed and approved by the IRB/IEC before the trial is initiated.

Any amendments to the protocol will require IRB/IEC approval before implementation of changes made to the trial design, except for changes necessary to eliminate an immediate hazard to trial patients.

The investigator will be responsible for the following:

- Providing summaries of the status of the trial to the IRB/IEC annually or more frequently in accordance with the requirements, policies, and procedures established by the IRB/IEC
- Notifying the IRB/IEC of SAEs or other significant safety findings as required by IRB/IEC procedures
- Providing oversight of the conduct of the trial at the site and adherence to requirements of 21 CFR, ICH guidelines, the IRB/IEC, European regulation 536/2014 for clinical studies (if applicable), and all other applicable local regulations

10.1.2. Financial Disclosure

Investigators and sub-investigators will provide Cytokinetics with sufficient, accurate financial information as requested to allow Cytokinetics to submit complete and accurate financial certification or disclosure statements to the appropriate regulatory authorities. Investigators are responsible for providing information on financial interests during the course of the trial and for 1 year after completion of the trial.

10.1.3. Informed Consent Process

The investigator or his/her representative will explain the nature of the trial to the patient or his/her legally authorized representative and answer all questions regarding the trial.

Patients must be informed that their participation is voluntary. Patients must be able to comprehend and be willing to sign a statement of informed consent that meets the requirements

of 21 CFR 50, local regulations, ICH guidelines, Health Insurance Portability and Accountability Act (HIPAA) requirements, where applicable, and the IRB/IEC or trial center.

The medical record must include a statement that informed consent was obtained before any trial-specific activities/procedures were performed and the date the consent was obtained. The authorized person obtaining the informed consent must also sign the ICF.

Patients must be re-consented to the most current version of the ICF(s) during their participation in the trial.

A copy of the ICF(s) must be provided to the patient or the patient’s legally authorized representative.

Patients are not required to sign a new ICF if they are retested during the initial screening window.

10.1.4. Data Protection

Patients will be assigned a unique identifier by Cytokinetics. Any patient records or datasets that are transferred to Cytokinetics will contain the identifier only; patient names or any information which would make the patient identifiable will not be transferred.

The patient must be informed that his/her personal trial-related data will be used by Cytokinetics in accordance with local data protection law. The level of disclosure must also be explained to the patient who will be required to give consent for their data to be used as described in the ICF.

The patient must be informed that his/her medical records may be examined by Clinical Quality Assurance auditors or other authorized personnel appointed by Cytokinetics, by appropriate IRB/IEC members, and by inspectors from regulatory authorities.

10.1.5. Committees Structure

The trial organization will include an Executive Committee (EC), Steering Committee (SC) and DMC.

The EC will contribute to trial design, implementation, data analysis, and communication of trial results and will consist of experts external to Cytokinetics who are qualified by their medical and scientific expertise and experience, one of the trial investigators, and a Cytokinetics representative. The responsibilities of the EC will be described in an EC charter.

The SC will contribute to implementation of the trial, data analysis, and communication of trial results. They will be HCM experts external to Cytokinetics and represent the different geographies the trial will be conducted in. The responsibilities of the SC will be described in a SC charter.

An independent DMC will be established for this trial to formally review the accumulating data periodically in order to assess risk to patients during the conduct of the trial. The DMC will include an external cardiologist with relevant expertise and other designated members with relevant expertise, eg, representing clinical science, clinical pharmacology, and biostatistics. The independent DMC membership will exclude the individuals from Cytokinetics or the contract research organization (CRO) trial team involved in trial conduct. The DMC members will have access to treatment assignments and patient level data from the clinical trial database. DMC

membership, responsibilities, relationship with Cytokinetics and the CRO, and the purpose and timing of the meetings will be further described in the DMC charter.

10.1.6. Data Quality Assurance

All patient data relating to the trial will be recorded on printed or electronic CRF unless transmitted to Cytokinetics or designee electronically (eg, laboratory data). The investigator is responsible for verifying that data entries are accurate and correct by physically or electronically signing the CRF.

The investigator must maintain accurate documentation (source data) that supports the information entered in the CRF.

The investigator must permit trial-related monitoring, audits, IRB/IEC review, and regulatory authority inspections and provide direct access to source data documents.

Monitoring details describing strategy (eg, risk-based initiatives in operations and quality such as Risk Management and Mitigation Strategies and Analytical Risk-Based Monitoring), methods, responsibilities and requirements, including handling of non-compliance issues and monitoring techniques (central, remote, or on-site monitoring) are provided in the Monitoring Plan.

Cytokinetics or designee is responsible for the data management of this trial including quality checking of the data.

Cytokinetics assumes accountability for actions delegated to other individuals (eg, CROs).

Trial monitors will perform ongoing source data verification to confirm that data entered into the CRF by authorized site personnel are accurate, complete, and verifiable from source documents; that the safety and rights of patients are being protected; and that the trial is being conducted in accordance with the currently approved protocol and any other trial agreements, ICH GCP, and all applicable regulatory requirements.

Records and documents, including signed ICFs, pertaining to the conduct of this trial, must be retained by the investigator in accordance with the strictest regulation applicable to this study and as obligated by the clinical trial agreement. No records may be destroyed during the retention period without the approval of Cytokinetics. No records may be transferred to another location or party without notification to Cytokinetics.

10.1.7. Source Documents

Source documents provide evidence for the existence of the patient and substantiate the integrity of the data collected. Source documents are filed at the investigator’s site.

Data reported in the CRF or entered in the CRF that are transcribed from source documents must be consistent with the source documents or the discrepancies must be explained. The investigator may need to request previous medical records or transfer records, depending on the trial. Also, current medical records must be available.

10.1.8. Trial and Site Start and Closure

The trial start date is the date on which the clinical trial will be open for recruitment of patients.

The first act of recruitment is the first site activated.

Cytokinetics or designee reserves the right to close the trial site or terminate the trial at any time for any reason at the sole discretion of Cytokinetics. Trial sites will be closed upon trial completion. A trial site is considered closed when all required documents and trial supplies have been collected and a site closure visit has been performed.

The investigator may initiate trial-site closure at any time, provided there is reasonable cause and sufficient notice is given in advance of the intended termination.

Reasons for the early closure of a trial site by Cytokinetics or investigator may include but are not limited to:

- Failure of the investigator to comply with the protocol, the requirements of the IRB/IEC or local regulatory authorities, Cytokinetics’s procedures, or GCP guidelines
- Inadequate recruitment of patients by the investigator
- Discontinuation of further IP development

If the trial is prematurely terminated or suspended, Cytokinetics shall promptly inform the investigators, the IECs/IRBs, the regulatory authorities, and any CRO(s) used in the trial of the reason for termination or suspension, as specified by the applicable regulatory requirements. The investigator shall promptly inform the patient and should assure appropriate patient therapy and/or follow-up.

10.2. Appendix 2: Clinical Laboratory Tests

The tests detailed in Table 9 will be performed by the central laboratory. If an issue arises with the central laboratory, a local laboratory can be used for eligibility after approval from the sponsor. If the results used for eligibility cannot be obtained from the central laboratory, a local laboratory may be used with the approval of the sponsor. If a local laboratory is used, duplicate samples must be drawn at the same time and sent to the central laboratory. Pregnancy testing for WOCBP at time points after screening may be performed locally.

Protocol-specific requirements for inclusion or exclusion of patients are detailed in Section 5 of the protocol.

Additional tests may be performed at any time during the trial as determined necessary by the investigator or required by local regulations.

Table 9: Protocol-Required Safety Laboratory Assessments

Chemistry		Urinalysis	Hematology	Other Assessments
Sodium	Total bilirubin	Specific gravity	Hemoglobin	CK-3773274 plasma concentration
Potassium	Direct bilirubin	pH	Hematocrit	
Chloride	CK	Blood	RBC	Pregnancy test[a]
Calcium	ALP	Protein	RDW	FSH[a]
Magnesium	LDH	Glucose	MCV	NT-proBNP[b]
Phosphorus	AST (SGOT)	Bilirubin	MCH	hs-cTnI
Urea	ALT (SGPT)		MCHC	Other biomarkers including: Galectin-3, PINP, TIMP-1, CITP and Soluble ST2
Creatinine	GGT		WBC	
Iron	TIBC		Platelets	
Glucose	Ferritin			PT-INR
Total protein	Bicarbonate			

ALP = alkaline phosphatase; ALT (SGPT) = alanine aminotransferase (serum glutamic-pyruvic transaminase); AST (SGOT) = aspartate aminotransferase (serum glutamic-oxaloacetic transaminase); CITP = collagen type 1; CK = creatine kinase; FSH = follicle-stimulating hormone; GGT = gamma-glutamyl transferase; hs-cTnI = high-sensitivity cardiac troponin I; LDH = lactic acid dehydrogenase; MCH = mean corpuscular hemoglobin; MCHC = mean corpuscular hemoglobin concentration; MCV = mean corpuscular volume; NT-proBNP = N-terminal pro-B-type natriuretic peptide; PINP = procollagen type 1 N-terminal propeptide; PT-INR = prothrombin time/international normalized ratio; RBC = red blood cell; RDW = red cell distributions width; SGOT = serum glutamic-oxaloacetic transaminase; SGPT = serum glutamic-pyruvic transaminase; Soluble ST2 = soluble suppression of tumorigenicity 2; TIBC= total iron binding protein; TIMP-1 = tissue inhibitor matrix metalloproteinase 1; WBC = white blood cell

[a] A pregnancy test is required for WOCBP; FSH only at screening if needed. If a urine pregnancy test is positive, a serum pregnancy test should be performed.

[b] NT-proBNP results will be masked throughout the study.

Investigators must document their review of each laboratory report.

Laboratory results that could unblind the trial will not be reported to investigative sites or other blinded personnel until the trial has been unblinded.

10.3. Appendix 3: Contraceptive Guidance

Definitions:

Woman of Childbearing Potential

A woman is considered fertile following menarche and until becoming post-menopausal unless permanently sterile (see below).

If fertility is unclear (eg, amenorrhea in adolescents or athletes) and a menstrual cycle cannot be confirmed before first dose of IP, additional evaluation should be considered.

Women in the following categories are not considered WOCBP:

1. Premenarchal
2. Premenopausal female with 1 of the following:
   - Documented hysterectomy
   - Documented bilateral salpingectomy
   - Documented bilateral oophorectomy

   For individuals with permanent infertility due to an alternate medical cause other than the above (eg, Mullerian agenesis, androgen insensitivity), investigator discretion should be applied to determining trial entry.

   Note: Documentation can come from the site personnel's review of the patient's medical records, medical examination, or medical history interview.
3. Postmenopausal female
   - A postmenopausal state is defined as no menses for 12 months without an alternative medical cause.
     – A high follicle stimulating hormone (FSH) level in the postmenopausal range may be used to confirm a postmenopausal state in women not using hormonal contraception or hormone replacement therapy. However, in the absence of 12 months of amenorrhea, confirmation with more than one FSH measurement (>40 IU/L or mIU/mL) is required.
   - Females on hormone replacement therapy and whose menopausal status is in doubt will be required to use one of the non-estrogen hormonal highly effective contraception methods if they wish to continue their hormone replacement therapy during the trial. Otherwise, they must discontinue hormone replacement therapy to allow confirmation of postmenopausal status before trial enrollment.

Highly Effective Method of Contraception

A highly effective method of contraception is one that has a failure rate of <1% per year when used consistently and correctly.

Examples of highly effective contraception that have low user dependency are:

- Implantable progestogen-only hormone contraception associated with inhibition of ovulation
- Intrauterine device (IUD)
- Intrauterine hormone-releasing system (IUS)
- Bilateral tubal occlusion
- Vasectomized partner, only when the absence of sperm has been confirmed and vasectomized partner is the sole sexual partner of the female patient

Examples of highly effective contraception that are user-dependent are:

- Combined hormonal methods of birth control include oral, intravaginal, transdermal, injectable, or implantable
- Oral or injectable progestogen-only hormone contraception associated with the inhibition of ovulation
- Sexual abstinence

  Note: Sexual abstinence is considered a highly effective method only if defined as refraining from heterosexual intercourse during the entire period of risk associated with the trial intervention. The reliability of sexual abstinence needs to be evaluated in relation to the duration of the trial and the preferred and usual lifestyle of the patient. Periodic abstinence (calendar, symptothermal, post-ovulation methods), withdrawal (coitus interruptus), spermicides only, and lactational amenorrhoea method (LAM) are not acceptable methods of contraception.

Contraception Guidance:

Women of Childbearing Potential

WOCBP must use at least one highly effective method of birth control. If any of the above highly effective methods of birth control are used, a male condom must also be used. Male condom and female condom should not be used together (due to risk of failure with friction).

If additional medications are given during treatment, the investigator is to review the prescribing information/summary of product characteristics for all concomitant therapy, as they may alter the contraceptive requirements. These additional medications may require an increase in the number of contraceptive methods and/or length of time that contraception is to be utilized after the last dose of protocol-required therapies. The investigator is to discuss these changes with the trial patient.

10.4. Appendix 4: Genetics

Use/Analysis of DNA

Genetic variation may impact a patient's response to IP, susceptibility to, and severity and progression of disease. Variable response to IP may be due to genetic determinants that impact drug absorption, distribution, metabolism, and excretion; mechanism of action of the drug; disease etiology; and/or molecular subtype of the disease being treated. Therefore, where local regulations and IRB/IEC allow, a blood sample will be collected for DNA analysis from patients consenting to provide the sample.

DNA samples will be used for research related to this trial may consist of the analysis of one or more candidate genes or the analysis of genetic markers throughout the genome (as appropriate).

The results of genetic analyses may be reported in the clinical study report or in a separate trial summary.

Cytokinetics will store the DNA samples in a secure storage space with adequate measures to protect confidentiality.

10.5. Appendix 5: Liver Safety: Actions and Follow-up Assessments

Drug-induced Liver Injury Reporting & Additional Assessments

To facilitate appropriate monitoring for signals of DILI, cases of concurrent aspartate aminotransferase (AST) or alanine aminotransferase (ALT) and total bilirubin (TBL) and/or international normalized ratio (INR) elevation require the following:

- The event is to be reported to Cytokinetics as an SAE within 24 hours of discovery or notification of the event (ie, before additional etiologic investigations have been concluded)
- The AE CRF that captures information necessary to facilitate the evaluation of treatment-emergent liver abnormalities is to be completed and sent to Cytokinetics.

Other events of hepatotoxicity and potential DILI are to be reported as SAEs if they meet the criteria for an SAE defined in Section 8.4.1.2.

Criteria for Permanent Discontinuation of Investigational Product and Other Protocol-required Therapies due to Potential Hepatotoxicity

CK-3773274 **must** be discontinued permanently and the patient should be followed according to the following recommendations for possible DILI, if ALL of the criteria below are met:

- TBL >2 × ULN or INR >1.5

AND

- increased AST or ALT from the relevant baseline value as specified below:

Baseline AST or ALT Value	AST or ALT Elevation
<ULN	>3 × ULN

AND

- no other cause for the combination of the above laboratory abnormalities is apparent; important alternative causes for elevated AST/ALT and TBL values include, but are not limited to:
  - hepatobiliary tract disease
  - viral hepatitis (eg, Hepatitis A/B/C/D/E, Epstein-Barr Virus, cytomegalovirus, Herpes Simplex Virus, Varicella, toxoplasmosis, and Parvovirus)
  - right sided heart failure, hypotension, or any cause of hypoxia to the liver causing ischemia
  - exposure to hepatotoxic agents/drugs or hepatotoxins, including herbal and dietary supplements, plants and mushrooms
  - heritable disorders causing impaired glucuronidation (eg, Gilbert's Syndrome, Crigler-Najjar syndrome) and drugs that inhibit bilirubin glucuronidation (eg, indinavir, atazanavir)

- alpha-one antitrypsin deficiency
- alcoholic hepatitis
- autoimmune hepatitis
- Wilson's disease and hemochromatosis
- nonalcoholic fatty liver disease including steatohepatitis
- nonhepatic causes (eg, rhabdomyolysis, hemolysis)

Criteria for Conditional Interruption of Investigational Product and Other Protocol-required Therapies due to Potential Hepatotoxicity

For patients who do not meet the criteria for permanent discontinuation of IP outlined above and have no underlying liver disease, the following rules are recommended for interruption of IP and other protocol required therapies:

- Elevation of either AST or ALT according to the following schedule:

Baseline AST or ALT Value	AST or ALT Elevation
Any	>8 × ULN at any time
Any	>5 × ULN but <8 × ULN for ≥2 weeks
Any	>5 × ULN but <8 × ULN and unable to adhere to enhanced monitoring schedule
Any	>3 × ULN with clinical signs or symptoms that are consistent with hepatitis (such as right upper quadrant pain/tenderness, fever, nausea, vomiting, jaundice)

OR

- TBL >3 × ULN at any time

IP and other protocol-required therapies, as appropriate must be withheld pending investigation into alternative causes of DILI. If IP is withheld, the patient is to be followed according to recommendations in this section for possible DILI. Rechallenge may be considered if an alternative cause for impaired liver tests (ALT, AST, ALP) and/or elevated TBL, is discovered and the laboratory abnormalities resolve to normal or baseline.

Rechallenge of Investigational Product and Other Protocol-required Therapies After Potential Hepatotoxicity

The decision to rechallenge the patient must be discussed and agreed upon unanimously by the patient, investigator, and Medical Monitor. Patients reinitiating IP after withholding for potential hepatotoxicity will restart IP, according to initial randomized allocation, on the same IP dose as established before the event and will not further titrate the dose.

If signs or symptoms recur with rechallenge, then IP must be permanently discontinued. Patients who clearly meet the criteria for permanent discontinuation must never be rechallenged.

Additional Clinical Assessments and Observation

All patients in whom IP(s) or protocol-required therapies is/are withheld (either permanently or conditionally) due to potential DILI or who experience AST or ALT elevations >3 × ULN are to undergo a repeat test and a period of "close observation" until abnormalities have stabilized, returned to normal, or returned to the patient's baseline levels. Recommended assessments and testing frequency that are to be performed during this period include:

- Repeat AST, ALT, ALP, bilirubin (total and direct), and INR within 48 hours of receiving results with repeat testing until stabilized
- In cases of TBL >2 × ULN or INR >1.5, retesting of liver tests, bilirubin (total and direct), and INR should be performed within 48 hours of receiving results with repeat testing 2-3 times per week until stabilized

Testing frequency of the above laboratory tests may decrease if the abnormalities stabilize or the IP(s) or protocol-required therapies has/have been discontinued AND the patient is asymptomatic.

- Initiate investigation of alternative causes for elevated AST or ALT and/or elevated TBL:
  - Obtain complete blood count (CBC) with differential to assess for eosinophilia
  - Obtain serum total immunoglobulin IgG, Anti-nuclear antibody (ANA), Anti Smooth Muscle Antibody, and Liver Kidney Microsomal antibody 1 (LKM1) to assess for autoimmune hepatitis
  - Obtain serum acetaminophen (paracetamol) levels
  - Obtain a more detailed history of:
    - Prior and/or concurrent diseases or illness
    - Exposure to environmental and/or industrial chemical agents
    - Symptoms (if applicable) including right upper quadrant pain, hypersensitivity type reactions, fatigue, nausea, vomiting and fever
    - Prior and/or concurrent use of alcohol, recreational drugs and special diets
    - Concomitant use of medications (including non-prescription medicines and herbal and dietary supplements), plants, and mushrooms
  - Obtain viral serologies
  - Obtain creatine phosphokinase, haptoglobin, lactate dehydrogenase, and peripheral blood smear
  - Perform appropriate liver imaging if clinically indicated
- Obtain appropriate blood sampling for PK analysis if this has not already been collected
- Obtain hepatology consult (liver biopsy may be considered in consultation with an hepatologist)

Follow the patient and the laboratory tests (ALT, AST, TBL, INR) until all laboratory abnormalities return to baseline or normal. The “close observation period” is to continue for a minimum of 4 weeks after discontinuation of all IP(s) and protocol required therapies.

The potential DILI event and additional information such as medical history, concomitant medications, and laboratory results must be captured in corresponding CRFs.

10.6. Appendix 6: Abbreviations

Table 10: List of Abbreviations

Abbreviation/Term	Explanation
AE	Adverse event
ALP	Alkaline phosphatase
ALT	Alanine aminotransferase
ANA	Anti-nuclear antibody
AST	Aspartate aminotransferase
CBC	Complete blood count
CGI	Clinical Global Impression scale
CI	Confidence interval
CIOMS	Council for International Organizations of Medical Sciences
C_{max}	Maximum plasma concentration observed
C_{trough}	Trough plasma concentration observed
CMH	Cochran–Mantel–Haenszel
CMR	Cardiac magnetic resonance
CPET	Cardiopulmonary exercise testing
CRF	Case report form
CRO	Contract research organization
CV	Cardiovascular
CYP	Cytochrome P450
DILI	Drug induced liver injury
DMC	Data monitoring committee
EC	Executive Committee
ECG	Electrocardiogra(m/phy)
ED	Early discontinuation
EOS	End of study
EOT	End of treatment
EQ-5D-5L	EuroQol 5-dimension 5-level instrument
FAS	Full analysis set
FSH	Follicle-stimulating hormone
FU	Follow up
GCP	Good Clinical Practice

Abbreviation/Term	Explanation
GLP	Good Laboratory Practice
HCM	Hypertrophic cardiomyopathy
HIPAA	Health Insurance Portability and Accountability Act
hs-cTnI	High sensitivity cardiac troponin I
IB	Investigator's Brochure
ICD	Implantable cardioverter defibrillators
ICF	Informed consent form
ICH	International Council for Harmonisation
IEC	Independent ethics committee
IgG	Immunoglobulin G
IMP	Investigational medicinal product
INR	International normalized ratio
IP	Investigational product
IRB	Institutional review board
IUD	Intrauterine device
IUS	Intrauterine hormone-releasing system
IWRS	Interactive web response system
KCCQ	Kansas City Cardiomyopathy Questionnaire
LAM	Lactational amenorrhoea method
LKM1	Liver Kidney Microsomal antibody 1
LSM	Least squares mean
LV	Left ventricle(ular)
LVEDV	Left ventricular end-diastolic volume
LVEF	Left ventricular ejection fraction
LVESV	Left ventricular end-systolic volume
LVFS	Left ventricular fractional shortening
LVOT	Left ventricular outflow tract
LVOT-G	Left ventricular outflow tract gradient
MedDRA	Medical Dictionary for Regulatory Activities
NIMP	Non-investigational medicinal product
NT-proBNP	n-terminal prohormone brain natriuretic peptide
NYHA	New York Heart Association

Abbreviation/Term	Explanation
oHCM	Obstructive hypertrophic cardiomyopathy
PD	Pharmacodynamics
PDS	Pharmacodynamics analysis set
PGI-C	Patient Global Impression of Change scale
PK	Pharmacokinetics
PKS	Pharmacokinetics analysis set
PRO	Patient-reported outcomes
pVO_2	Peak oxygen uptake
RBC	Red blood cell
RER	Respiratory exchange ratio
SAE	Serious adverse event
SAS	Safety analysis set
SAQ-7	Seattle Angina Questionnaire -7
SC	Steering Committee
SD	Standard deviation
SoA	Schedule of activities
SoC	Standard of care
TBL	Total bilirubin
ULN	Upper limit of normal
VAT	Ventilatory anaerobic threshold
WOCBP	Women of childbearing potential

10.7. Appendix 7: Protocol Amendment History

The Protocol Amendment Summary of Changes (Table 1) for the current amendment is located directly before the Table of Contents (TOC).

11. REFERENCES

Chuan, P., Sivaramakrishnan, S., Ashley, E. A. and Spudich, J. A. (2012). "Cell-intrinsic functional effects of the α-cardiac myosin arg-403-gln mutation in familial hypertrophic cardiomyopathy." Biophysical journal 102(12): 2782-2790.

Criteria Committee of the New York Heart, A. (1994). Nomenclature and criteria for diagnosis of diseases of the heart and great vessels. Boston: Little, Brown & Co.

Elliott, P. M., Anastasakis, A., Borger, M. A., Borggrefe, M., Cecchi, F., Charron, P., et al. (2014). "2014 esc guidelines on diagnosis and management of hypertrophic cardiomyopathy: The task force for the diagnosis and management of hypertrophic cardiomyopathy of the european society of cardiology (esc)." European Heart Journal 35(39): 2733-2779.

Gersh, B. J., Maron, B. J., Bonow, R. O., Dearani, J. A., Fifer, M. A., Link, M. S., et al. (2011). "2011 accf/aha guideline for the diagnosis and treatment of hypertrophic cardiomyopathy." Journal of the American College of Cardiology 58(25): e212-e260.

Husser, D., Ueberham, L., Jacob, J., Heuer, D., Riedel-Heller, S., Walker, J., et al. (2018). "Prevalence of clinically apparent hypertrophic cardiomyopathy in germany-an analysis of over 5 million patients." PLOS ONE 13(5): e0196612-e0196612.

Kristensen, S. D., Knuuti, J., Saraste, A., Anker, S., BÃ¸tker, H. E., Hert, S. D., et al. (2014). "2014 esc/esa guidelines on non-cardiac surgery: Cardiovascular assessment and management: The joint task force on non-cardiac surgery: Cardiovascular assessment and management of the european society of cardiology (esc) and the european society of anaesthesiology (esa)." European Heart Journal 35(35): 2383-2431.

Magnusson, P., Palm, A., Branden, E. and Morner, S. (2017). "Misclassification of hypertrophic cardiomyopathy: Validation of diagnostic codes." Clinical epidemiology 9: 403-410.

Marian, A. J. and Braunwald, E. (2017). "Hypertrophic cardiomyopathy: Genetics, pathogenesis, clinical manifestations, diagnosis, and therapy." Circulation research 121(7): 749-770.

Maron, B. J. (2018). "Clinical course and management of hypertrophic cardiomyopathy." New England Journal of Medicine 379(20): 655-668.

Maron, M. S., Iacopo, O., Andrey, G. Z., Mark, S. L., Natesa, G. P., Jeffery, T. K., et al. (2006). "Hypertrophic cardiomyopathy is predominantly a disease of left ventricular outflow tract obstruction." Circulation 114(21): 2232-2239.

Maron, M. S., Rowin, E. J., Olivotto, I., Casey, S. A., Arretini, A., Tomberli, B., et al. (2016). "Contemporary natural history and management of nonobstructive hypertrophic cardiomyopathy." Journal of the American College of Cardiology 67(12): 1399-1409.

Ommen, S. R., Mital, S., Burke, M. A., Day, S. M., Deswal, A., Elliott, P., et al. (2020). "2020 aha/acc guideline for the diagnosis and treatment of patients with hypertrophic cardiomyopathy." Circulation 142(25): e558-e631.

Ponikowski, P., Voors, A. A., Anker, S. D., Bueno, H., Cleland, J. G. F., Coats, A. J. S., et al. (2016). "2016 esc guidelines for the diagnosis and treatment of acute and chronic heart failure: The task force for the diagnosis and treatment of acute and chronic heart failure of the european society of cardiology (esc)developed with the special contribution of the heart failure association (hfa) of the esc." European Heart Journal 37(27): 2129-2200.

Pujades-Rodriguez, M., Guttmann, O. P., Gonzalez-Izquierdo, A., Duyx, B., O'Mahony, C., Elliott, P., et al. (2018). "Identifying unmet clinical need in hypertrophic cardiomyopathy using national electronic health records." PLOS ONE 13(1): e0191214-e0191214.

Sommese, R. F., Sung, J., Nag, S., Sutton, S., Deacon, J. C., Choe, E., et al. (2013). "Molecular consequences of the r453c hypertrophic cardiomyopathy mutation on human β-cardiac myosin motor function." Proceedings of the National Academy of Sciences 110(31): 12607-12612.

Spudich, J. A., Aksel, T., Bartholomew, S. R., Nag, S., Kawana, M., Yu, E. C., et al. (2016). "Effects of hypertrophic and dilated cardiomyopathy mutations on power output by human β-cardiac myosin." The Journal of Experimental Biology 219(2): 161-167.

Toepfer, C. N., Wakimoto, H., Garfinkel, A. C., McDonough, B., Liao, D., Jiang, J., et al. (2019). "Hypertrophic cardiomyopathy mutations in mybpc3 dysregulate myosin." Science Translational Medicine 11(476): eaat1199.

Wilson, W. S., Criley, J. M. and Ross, R. S. (1967). "Dynamics of left ventricular emptying in hypertrophic subaortic stenosis: A cineangiographic and hemodynamic study." American Heart Journal 73(1): 4-16.

Summary of changes

in the protocol

PROTOCOL AMENDMENT SUMMARY OF CHANGES

DOCUMENT HISTORY	
**Document**	**Date**
Amendment 01	17 August 2021
Original Protocol	26 July 2021

Amendment 01 (17 August 2021)

The purpose of this amendment is to revise Exclusion Criterion 214 to correctly state that patients with an oxygen saturation less than 90% are excluded from the trial

Table 1: Protocol Amendment Summary of Changes

Section # and Name	Description of Change	Brief Rationale
5.1 Inclusion Criteria	Revise Inclusion Criterion 104 to include patients with a wall thickness ≥13 mm in one or more wall segments and a known-disease-causing gene mutation or positive family history of HCM	To be consistent with the current diagnostic criteria for HCM
5.2 Exclusion Criteria	Correct Exclusion Criterion 214 to state that patients with an oxygen saturation < 90% are not eligible for this study	To correct an error in the original protocol.
5.2 Exclusion Criteria	Clarify that the criteria for the CMR sub-study are exclusion criteria	To clarify CMR sub-study exclusion criteria
Protocol Amendment Summary of Changes and 10.7 Appendix 8: Protocol Amendment History	New sections	These sections are applicable now that the protocol has been amended.
Global	Update of protocol version date.<br>Editorial (formatting, typographical, and grammatical) corrections throughout the protocol.	Administrative changes.

PROTOCOL AMENDMENT SUMMARY OF CHANGES

DOCUMENT HISTORY	
**Document**	**Date**
Amendment 02	10 December 2021
Amendment 01	17 August 2021
Original Protocol	26 July 2021

Amendment 02 (10 December 2021)

The purpose of this amendment is to allow enrollment of patients treated with disopyramide as a background therapy. Changes were also made to the schedule of activities. Editorial changes (formatting, grammatical, etc.) were made throughout the protocol.

While new data were added to the Benefit/Risk Assessment (Section 2.3), there is no change to the overall benefit-risk profile for CK-3773274.

Table 1: Protocol Amendment 02 Summary of Changes

Section # and Name	Description of Change	Brief Rationale
1.1 Synopsis	Update to exploratory endpoints	To be consistent with Section 3 Objectives and Endpoints
1.2 Schema	Update to Figure 2 trial schema	To correctly reflect entry requirements
1.3 Schedule of Activities	Removal of PGI-C at Day 1 and Week 28 (EOS)	To correctly capture patient's response compared to before treatment
1.3 Schedule of Activities	Removal of CGI at Day 1	To correctly capture response compared to before treatment
1.3 Schedule of Activities	Addition of EQ-5D-5L at Weeks 2, 4, 6, 8, 12, 16, 20, and 28 (EOS)	To align timepoints of KCCQ and NYHA
1.3 Schedule of Activities	Removal of Medical/Surgical history at Day 1	To avoid duplication of data entered at screening
1.3 Schedule of Activities	Addition of pregnancy test at Weeks 2 and 6	To test for pregnancy more frequently
1.3 Schedule of Activities and 8.10 Serum Collection for Future Analysis	Addition of plasma collection for future analysis	To collect and store plasma samples for future testing of biomarkers
1.3 Schedule of Activities and 8.1.4 Week 24: End of Treatment Visit	Added option to split week 24 visit over 2 days	To help with scheduling logistics at the site level
2.3.1 Risk Assessment	Update to Phase II study results	To update with new information

Table 1: Protocol Amendment 02 Summary of Changes (Continued)

Section # and Name	Description of Change	Brief Rationale
4.1.1 Number of Sites	Increase the number of sites to approximately 105	Updated to reflect expected site count
4.1 Overall Design	Addition of enrollment cap on patients treated with disopyramide	To avoid over-representation of subjects that have intrinsic baseline differences
4.1.5 CMR Imaging Sub-Study and 1.3 Schedule of Activities	Increase the number of patients in CMR sub-study	To ensure that the CMR sub-study is appropriately powered
5.1 Inclusion Criteria	Revised inclusion criterion 110	To clarify inclusion criteria for patients taking disopyramide
5.3 Lifestyle Considerations and 8.3.1 Cardiopulmonary Exercise Testing	Addition of guidelines for patient lifestyle restrictions prior to CPET	To state requirements for patients prior to CPET
5.4 Screen Failures and 8.3.3 Cardiac Magnetic Resonance	Addition of guideline for performing CMR for rescreened patients	To clarify CMR requirements
8.2.4 Week 24: End of Treatment Visit	Increase the CMR window at Week 24 (EOT)	To clarify allowable window for CMR at EOT
10.1.6 Data Quality Assurance	Update to document retention period	To extend retention period in alignment with ICH GCP requirements
10.2 Appendix 2: Clinical Laboratory Tests	Deletion of language regarding use of local laboratories	No local laboratories will be used for this study

PROTOCOL AMENDMENT SUMMARY OF CHANGES

DOCUMENT HISTORY	
**Document**	**Date**
Amendment 03	03 January 2023
Amendment 02	10 December 2021
Amendment 01	17 August 2021
Original Protocol	26 July 2021

Amendment 03 (03 January 2023)

The main purpose of this amendment is as follows:

- To add endpoints to evaluate duration of time that participants are eligible for septal reduction therapy.
- To update pVO_2 criterion to allow for patients with higher predicted pVO_2
- To add the option to increase the sample size based on pVO_2 variability and missing data rate
- To clarify that designated site staff who are unmasked to echocardiogram results remain blinded to patient treatment assignments.
- Editorial changes (formatting, grammatical, etc.) were made throughout the protocol.

Table 1: Protocol Amendment 03 Summary of Changes

Section # and Name	Description of Change	Brief Rationale
Title Page<br>Protocol Approval Page	Updated sponsor address	To reflect change in sponsor address
Protocol Approval Page<br>1.4 Key Contacts	Updated medical monitor contact information	To reflect change in medical monitor
1.1 Synopsis<br>2.3.1 Risk Assessment<br>2.3.2 CK-3773274 Benefit Assessment	Added CY 6021 Cohort 3 information	To provide up to date information
1.1 Synopsis<br>Table 2: Trial Objectives and Endpoints<br>9.4.3 Secondary Endpoint(s)<br>9.4.4 Exploratory Endpoint(s)	Updated objectives and endpoints to include effect on SRT eligibility	To assess effect of treatment on SRT eligibility and to define SRT eligible
1.1 Synopsis<br>5.1 Inclusion Criteria	Updated pVO_2 criterion	To allow for patients with higher predicted pVO_2

Table 1: Protocol Amendment 03 Summary of Changes (Continued)

Section # and Name	Description of Change	Brief Rationale
1.1 Synopsis<br>5.2 Exclusion Criteria	Added new language regarding atrial fibrillation criteria	To clarify patients with atrial fibrillation are only excluded from the study if they meet the listed exclusion criteria
1.1 Synopsis<br>4.1 Overall Design<br>6.5 Concomitant Therapy	Added language to clarify background therapy should be individually optimized	To clarify that background therapy should be individually optimized
1.1 Synopsis<br>4.1.1 Number of Sites	Increased number of sites	To allow for additional sites in the study
1.1 Synopsis<br>9.2 Sample Size Determination	Added option to increase sample size based on the missing data rate and blinded monitoring of variability (standard deviation) of the primary endpoint (change in pVO_2)	To maintain the intended study power
1.1 Synopsis<br>9.4.1.1 Multiplicity Adjustment<br>9.4.3 Secondary Endpoints	Updated to include the secondary endpoint of total duration SRT eligibility during the 24 Weeks and the closed testing procedure	To include the new secondary endpoint of total duration of SRT eligibility during the 24-week treatment period and update the testing hierarchy to include this endpoint
1.3 Schedule of Activities	Updated PT-INR timepoints	PT-INR was previously collected at screening, Day 1, and UNS DILI visits. This change clarifies PT-INR is not needed at Day 1 and is needed at screening and UNS DILI visits.
1.3 Schedule of Activities	Updated footnote for Week 28	To change timepoint for Week 28/EOS to 4 weeks after last dose of IP
1.3 Schedule of Activities	Updated footnote for ED visit	To align with section 8.1.5 of the protocol

Table 1: Protocol Amendment 03 Summary of Changes (Continued)

Section # and Name	Description of Change	Brief Rationale
1.3 Schedule of Activities<br>8.1 Visit Schedule<br>8.2.3 Cardiac Magnetic Resonance	Added clarification regarding order of CPET and CMR	To clarify order of procedures
1.3 Schedule of Activities<br>8.1.1 Screening Visit	Updated screening CPET window	To allow for more flexibility
1.3 Schedule of Activities<br>Table 9: Protocol-Required Safety Laboratory Assessments	Added language for pregnancy testing	To add serum pregnancy testing in the event of a positive urine pregnancy test
5.1 Inclusion Criteria	Updated timeframe for refraining from sperm donation<br>Updated timeframe for contraception use when the woman is of childbearing potential	To increase timeframe for refraining from sperm donation<br>To clarify timeframe contraception measures needed for both partners for male participants with WOCBP partners
5.2 Exclusion Criteria<br>8.1.5 Week 28: End of Study Visit	Updated exclusion criterion 219	To allow screening assessments for an open-label extension study of aficamten to be completed at the CY 6031 EOS visit
5.2 Exclusion Criteria	Added exclusion criterion for CMR sub-study	To clarify that patients who do not consent to the CMR sub-study will be excluded
5.2 Exclusion Criteria	Updated language for exclusion criterion 212	To clarify exclusion criterion for septal reduction therapy
5.4 Screen Failures	Added language regarding echocardiogram and CPET retesting	To clarify screening echocardiograms and CPETs failed due to technical insufficiencies can be repeated
6.2 Preparation/Handling/Storage/Accountability	Added language for number of tablets to take	To clarify patient dosing instructions

Table 1: Protocol Amendment 03 Summary of Changes (Continued)

Section # and Name	Description of Change	Brief Rationale
6.3 Measures to Minimize Bias: Randomization and Blinding<br>6.6.3 LVEF Safety Threshold<br>8.2.2 Echocardiography	Updated wording from unblinded to unmasked<br>Added language to provide guidelines for unmasked staff	To clarify unmasked site staff will not have access to patient treatment assignments<br>To give sites the option to have an unmasked designee<br>To clarify unmasked staff should not reveal echocardiogram results to blinded staff except in the event of a critical safety issue
6.5.2 Rescue Medication	Updated wording	To clarify the medication example
6.7 Access to Investigational Product after the End of the Study	Added clarification on open-label extension study	To provide clarification on open-label extension study
8.2.1 Cardiopulmonary Exercise Testing	Added details regarding weight collection	To specify when weight should be collected
8.4.1.10 Pregnancy and Breastfeeding	Updated timeline for collection of breastfeeding information<br>Added language regarding collection of infant data	To clarify safety follow up timelines
8.6 Pharmacokinetics	Updated number of samples	To correct an error in the protocol
8.7 Optional Genetics	Added additional language regarding testing	To clarify process for optional genetics testing and to clarify that certified results may be released to the investigator
8.9 Optional Serum and Plasma Collection for Future Analysis	Updated to add storage of genetic samples and to make storage of plasma, serum, and genetic samples optional	To give patients the option to consent to storage of samples for future research

Table 1: Protocol Amendment 03 Summary of Changes (Continued)

Section # and Name	Description of Change	Brief Rationale
Table 9: Protocol-Required Safety Laboratory Assessments	Removed procollagen 3 N-terminal peptide (PIIINP) testing<br>Added (prothrombin time/international normalized ratio) PT-INR	To remove unnecessary testing<br>To correct an error in the protocol
10.2 Appendix 2: Clinical Laboratory Tests	Added the option for using a local laboratory at screening	To give sites an alternative option if there are issues with the central laboratory
10.3 Appendix 3: Contraceptive Guidance	Corrected language regarding condom use	To be consistent with the inclusion criterion
Figure 1: Statistical Testing Hierarchy for Trial Endpoints	Updated figure	To ensure consistency with protocol updates

PROTOCOL AMENDMENT SUMMARY OF CHANGES

DOCUMENT HISTORY	
**Document**	**Date**
Amendment 04	08 December 2023
Amendment 03	03 January 2023
Amendment 02	10 December 2021
Amendment 01	17 August 2021
Original Protocol	26 July 2021

Amendment 04 (08 December 2023)

The main purpose of this amendment is as follows:

- To add a safety endpoint that contextualizes observations of LVEF <50% with clinically relevant associated findings.
- To update the definition of full analysis set.
- To update the testing hierarchy to use a closed testing procedure with pre-specified testing order to test the secondary endpoints once the primary endpoint achieves statistical significance.

Table 1: Protocol Amendment 04 Summary of Changes

Section # and Name	Description of Change	Brief Rationale
1.1 Synopsis<br>3. Objectives and Endpoints	Added the safety endpoint of incidence of LVEF <50% with signs and symptoms of heart failure (concomitant adverse event of heart failure or dyspnea) and/or increase in NT-proBNP from baseline	This additional safety endpoint contextualizes an observation of LVEF <50% with clinically relevant associated findings
1.1 Synopsis<br>9.3 Populations For Analyses	Updated full analysis set definition to include all randomized patients	Per FDA's recommendation

Table 1: Protocol Amendment 04 Summary of Changes (Continued)

Section # and Name	Description of Change	Brief Rationale
1.1 Synopsis<br>9.4.1.1 Multiplicity Adjustment	Revised testing hierarchy; and made editorial updates	The testing hierarchy was simplified from a parallel gatekeeping method to a closed testing procedure. This adjustment allows for the examination of secondary endpoints at two-sided alpha level of 0.05 once the primary endpoint achieves statistical significance at the prespecified sequential testing order

Data S2. Statistical analysis plan

Original statistical analysis plan

STATISTICAL ANALYSIS PLAN

VERSION: 1 Final

DATE OF PLAN:

April 03, 2023

STUDY DRUG:

CK-3773274 (aficamten)

PROTOCOL NUMBER:

CY 6031

STUDY TITLE:

A Phase 3, Multi-Center, Randomized, Double-blind, Placebo- controlled Trial to Evaluate the Efficacy and Safety of CK-3773274 in Adults with Symptomatic Hypertrophic Cardiomyopathy and Left Ventricular Outflow Tract Obstruction

BASED ON:

Protocol Amendment 3, 03 January 2023

SPONSOR:

Cytokinetics, Inc.

350 Oyster Point Blvd., South San Francisco, CA 94080

650-624-3000

This study is being conducted in compliance with good clinical practice, including the archiving of essential documents.

SIGNATURE PAGE

This document has been prepared and/or reviewed by*:

Amy Wohltman, ME, Director, Biostatistics Cytokinetics, Inc.	Signature	Date

This document has been reviewed and accepted by*:

Lisa Meng, PhD, VP, Clinical Biometrics, Cytokinetics, Inc.	Signature	Date
Steve Heiner, MD, VP, Clinical Research and Therapeutic Area Lead Cardiovascular, Cytokinetics, Inc.	Signature	Date
Stuart Kupfer, MD, SVP, Chief Medical Officer, Cytokinetics, Inc.	Signature	Date
Bonnie Charpentier, SVP, Regulatory and Compliance, Cytokinetics, Inc.	Signature	Date

*See electronic signatures at the end of the document.

TECHNICAL SUMMARY REPORT (TSR)

**Name of Sponsor/Company**<br>Cytokinetics, Inc.	**Individual Study Table Referring to Part of the Dossier:**<br>**Volume:**	***(For National Authority Use Only)*:**
**Name of Finished Product:**<br>No generic or trade name assigned	**Page:**	
**Name of Active Ingredient:**<br>Aficamten (CK-2773274)		
**Title of Study:** A Phase 3, Multi-Center, Randomized, Double-blind, Placebo- controlled Trial to Evaluate the Efficacy and Safety of CK-3773274 in Adults with Symptomatic Hypertrophic Cardiomyopathy and Left Ventricular Outflow Tract Obstruction		
**Investigators**:<br>Study Center(s): Patients will be enrolled from approximately 105 sites worldwide.		
**Studied period (years):** 2022 to 2023	**Phase of development:** Phase 3	

Objectives and Endpoints:

Objectives	***Endpoint(s)***
Primary	
• To evaluate the effect of CK-3773274 on exercise capacity in patients with symptomatic oHCM	• Change in peak oxygen uptake (pVO2) by cardiopulmonary exercise testing (CPET) from baseline to Week 24
Secondary	
• To evaluate the effect of CK-3773274 on patient health status	• Change in Kansas City Cardiomyopathy Questionnaire – Clinical Summary Score (KCCQ-CSS) from baseline to Week 12 and Week 24
• To evaluate the effect of CK-3773274 on New York Heart Association (NYHA) Functional Classification	• Proportion of patients with ≥1 class improvement in NYHA Functional Class from baseline to Week 12 and Week 24
• To evaluate the effect of CK-3773274 on post-Valsalva left ventricular outflow tract gradients (LVOT-G)	• Change in post-Valsalva LVOT-G from baseline to Week 12 and Week 24<br>• Proportion of patients with post-Valsalva LVOT-G <30 mmHg at Week 12 and Week 24
• To evaluate the effect of CK-3773274 on exercise capacity	• Change in total workload during CPET from baseline to Week 24
• To evaluate the effect of CK-3773274 on duration of eligibility for septal reduction therapy	• Total duration of septal reduction therapy (SRT) eligible during the 24 Week treatment period in patients who were SRT eligible at baseline

Methodology:

This is a Phase 3 randomized, placebo-controlled, double-blind, multi-center trial in patients with symptomatic oHCM. Approximately 270 eligible patients will be randomized in a 1:1 ratio to receive CK-3773274 or placebo. Doses of 5, 10, 15, or 20 mg or matching placebo will be administered in an escalating manner using echocardiography to guide dose titration. Randomization will be stratified by use of beta-blockers and CPET exercise modality.

The trial will comprise three periods. The screening period will be up to 6 weeks in duration. The double-blind placebo-controlled treatment period will last 24 weeks. Following the final dose of investigational product (IP), there will be a 4-week safety follow-up period. IP will be administered orally once daily. During the initial six weeks of the treatment period, IP doses will be individually titrated at Weeks 2, 4, and 6 using echocardiography. Dose escalation at the Weeks 2, 4, and 6 visits will occur only if a patient has a post-Valsalva LVOT-G ≥30 mmHg and a biplane LVEF ≥55%. An echocardiogram will be performed at each subsequent visit during the trial and the dose down-titrated if necessary. The primary endpoint of pVO2 will be measured by CPET at screening and at end of treatment (Week 24).

Number of Subjects (planned and analyzed): Approximately 270 patients will be randomized to CK-3773274 or placebo at 1:1 ratio.

Diagnosis and main criteria for inclusion:

The key inclusion criteria are below. A full listing of eligibility criteria can be found in protocol Section 5.

- Males and females between 18 and 85 years of age, inclusive, at screening.
- Body mass index <35 kg/m^2.
- Diagnosed with HCM per the following criteria:
  − Has LV hypertrophy and non-dilated LV chamber in the absence of other cardiac disease and
  − Has an end-diastolic LV wall thickness as measured by the echocardiography core laboratory of:
    - ≥15 mm in one or more myocardial segments OR
    - ≥13 mm in one or more wall segments and a known-disease-causing gene mutation or positive family history of HCM
- Has resting LVOT-G ≥30 mmHg and post-Valsalva LVOT-G ≥50 mmHg during screening as determined by the echocardiography core laboratory.
- LVEF ≥60% at screening as determined by the echocardiography core laboratory.
- NYHA Functional Class II or III at screening.
- Hemoglobin ≥10g/dL at screening.
- Respiratory exchange ratio (RER) ≥1.05 and pVO_2 ≤90% predicted on the screening CPET per the core laboratory.
- Patients on beta-blockers, verapamil, diltiazem, or disopyramide should have been on stable doses for >6 weeks prior to randomization and anticipate remaining on the same medication regimen during the trial. Patients treated with disopyramide must also be concomitantly treated with a beta blocker and/or calcium channel blocker

Test product, dose and mode of administration:

Aficamten will be administered orally once daily with or without food. Patients receiving CK-3773274 start at a dose of 5 mg once daily and may escalate through doses of 10, 15, and 20 mg once daily during the initial six weeks of treatment if they continue to meet the escalation criteria (post-Valsalva LVOT-G ≥30 mmHg and a biplane LVEF ≥55%) or will stop at their current dose when escalation criteria are not met.

Duration of treatment:

After signing the informed consent form, patients will complete assessments to determine trial eligibility during a screening period of up to 6 weeks in duration. The double-blind placebo-controlled treatment period will last 24 weeks. Following the final dose of IP, there will be a 4-week safety follow-up period.

Reference therapy, dose and mode of administration:

Doses of 5, 10, 15, or 20 mg or matching placebo will be administered in an escalating manner using echocardiography to guide dose titration.

Criteria for evaluation (see protocol Section 3):

Efficacy:

The primary efficacy endpoint is change in peak oxygen uptake (pVO_2) by cardiopulmonary exercise testing (CPET) from baseline to Week 24.

The secondary endpoints are as follows:

- Change in Kansas City Cardiomyopathy Questionnaire – Clinical Summary Score (KCCQ-CSS) from baseline to Week 12 and Week 24
- Proportion of patients with ≥1 class improvement in NYHA Functional Class from baseline to Week 12 and Week 24
- Change in post-Valsalva LVOT-G from baseline to Week 12 and Week 24
- Proportion of patients with post-Valsalva LVOT-G <30 mmHg at Weeks 12 and 24
- Change in total workload during CPET from baseline to Week 24
- Total duration of SRT eligible during the 24 Week treatment period in patients who were SRT eligible at baseline

Safety:

- Incidence of reported major adverse cardiac events (cardiovascular [CV] death, cardiac arrest, non-fatal stroke, non-fatal myocardial infarction, CV hospitalization)
- Incidence of new onset persistent atrial fibrillation
- Incidence of appropriate implantable cardiac defibrillator (ICD) discharges and aborted sudden cardiac death
- Incidence of left ventricular ejection fraction (LVEF) <50%
- Incidence of treatment emergent adverse events

Statistical methods:

Unless specified otherwise, efficacy analyses will be performed on the full analysis set (FAS), which includes all randomized patients who receive at least one dose of IP and have at least one post-baseline efficacy assessment. The primary analysis will test the null hypothesis that there is no treatment difference in the primary endpoint between patients randomized to placebo and those randomized to CK-3773274 in the FAS. Change from baseline in pVO2 will be analyzed using an ANCOVA model with treatment group, randomization stratification factors, baseline pVO2 and baseline weight as covariates.

For preservation of the overall type I error rate at two-sided 0.05 for the primary and secondary endpoints, the primary endpoint is tested first at two-sided 0.05. If the primary endpoint achieves statistical significance at two-sided $p<0.05$, then a parallel gatekeeper method with two-sided 0.025 separately allocated to Week 12 and to Week 24 is applied for the first four secondary endpoints, with their testing being in the sequential order of KCCQ-CSS change from baseline, proportion of patients with >1 NYHA functional class improvement, post-Valsalva LVOT-G change from baseline and proportion of patients with post-Valsalva LVOT-G < 30 mm Hg. If all four of the first four secondary endpoints at Week 12 (or at Week 24) have two-sided $p\leq0.025$, then there is recycling of the 0.025 for Week 12 (or Week 24) to Week 24 (or Week 12) so that 0.05 is applicable to the corresponding testing of the first four secondary endpoints. If $p\leq0.025$ for the first four secondary endpoints at Week 12 (or at Week 24) and if $p\leq0.05$ for the first four secondary endpoints at Week 24 (or at Week 12), then the fifth secondary endpoint is tested at two-sided 0.05. If $p\leq0.05$ for the fifth secondary endpoint at Week 24, the sixth secondary endpoint will be tested at two-sided 0.05. SRT eligibility is defined as resting or provoked LVOT-G $\geq$ 50 mmHg AND NYHA Functional Class $\geq$ 3. A final determination of the order of fifth and sixth secondary endpoints will be made once the trial is fully enrolled. The total duration of SRT eligibility over the 24-week of treatment in patients who were SRT eligible at baseline might be brought up to the 5th secondary endpoint if the proportion of patients with baseline NYHA III is determined to be approximately 30% or greater. The final decision will be made before the database lock and documented in the SAP.

The multiple testing procedure is illustrated in Figure 1 below.

Figure 1: Statistical Testing Hierarchy for Primary and Secondary Endpoints

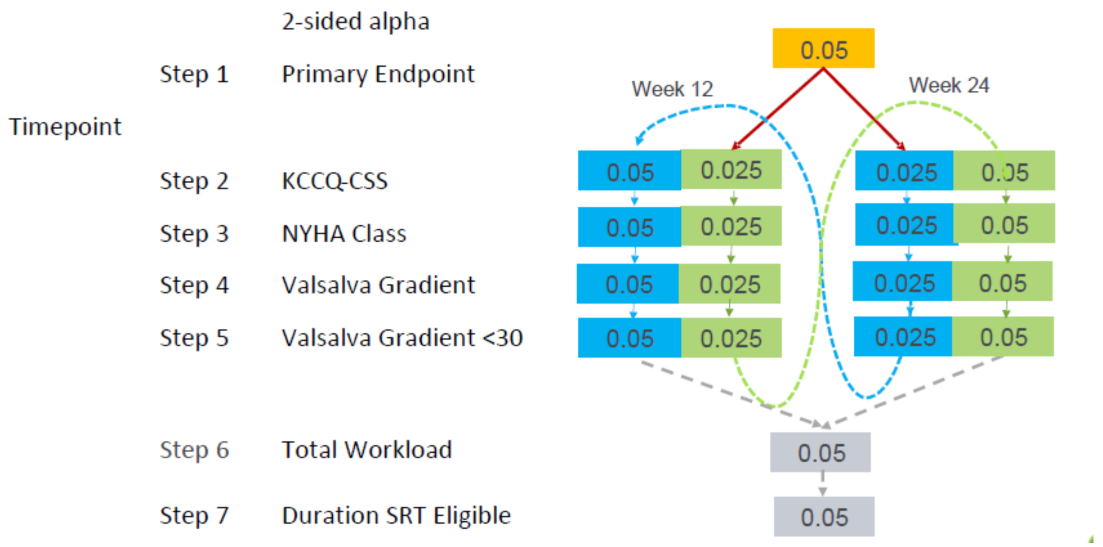

Multiplicity will be addressed using a parallel gatekeeping method detailed in Section 7.6.

The proportion of responders in various exploratory endpoints in FAS will be analyzed using Cochran–Mantel–Haenszel (CMH) test stratified by randomization factors. The p-value and 95% confidence interval (CI) will be obtained using exact method. Other change from baseline endpoints will be analyzed using mixed measures repeated model with treatment, visit, randomization stratification factors, treatment by visit, baseline by visit interaction as fixed effect and baseline assessment as covariate. Total of SRT eligible will be analyzed using an ANCOVA model with treatment group and randomization stratification factor beta blocker use/no use as fixed effects adjusting for significant baseline characteristics.

Safety analyses will be performed on the safety analysis set (SAS) which includes all patients who received at least one dose of IP. The pharmacokinetics analysis set (PKS) will consist of patients who have at least one evaluable plasma concentration of CK-3773274.

The number and percentage of patients reporting any treatment-emergent AEs will be coded using the MedDRA dictionary and be tabulated by system organ class and preferred term.

TABLE OF CONTENTS

SIGNATURE PAGE 2
TECHNICAL SUMMARY REPORT (TSR)....................3
TABLE OF CONTENTS....................8
LIST OF TABLES....................11
LIST OF FIGURES 11
LIST OF ABBREVIATIONS....................12
SAP VERSION HISTORY 14
1. INTRODUCTION....................15
2. STUDY OBJECTIVES AND ENDPOINTS....................16
2.1. Study Objectives....................16
2.1.1. Primary Objective....................16
2.1.2. Secondary Objective....................16
2.2. Study Endpoints....................16
2.2.1. Primary Endpoints 16
2.2.2. Secondary Endpoints 16
2.2.3. Exploratory Endpoints....................16
2.2.4. Safety Endpoints....................18
2.2.5. PK Parameters 19
3. STUDY DESIGN 20
3.1. Summary of Study Design....................20
3.2. Definition of Study Drugs 20
3.3. Sample Size Considerations 20
3.3.1. Sample Size Justifications 20
3.4. Randomization....................21
3.5. Clinical Assessments 21
3.5.1. Efficacy Assessments 21
3.5.1.1. Echocardiography....................21
3.5.1.2. Patient-Reported Outcomes....................23
3.5.1.3. CMR Assessments....................24
3.5.1.4. CPET Assessments....................25
3.5.2. Safety Assessments....................26

3.5.3. Pharmacokinetics Assessments 26
4. PLANNED ANALYSES 27
4.1. Interim Analyses 27
4.2. Final Analyses 27
5. GENERAL CONSIDERATIONS FOR DATA ANALYSES AND HANDLING 28
5.1. General Summary Table and Individual Subject Data Listing Considerations 28
5.2. General Post Text Summary Table and Individual Subject Data Listing Format Considerations 28
5.3. Data Management 28
5.4. Data Presentation Conventions 28
5.5. Analysis Populations 29
5.5.1. All Screened Patients 29
5.5.2. All Randomized Set 29
5.5.3. Safety Analysis Set 29
5.5.4. Full Analysis Set 29
5.5.5. Pharmacokinetics Analysis Set 30
5.6. Baseline Definition 30
5.7. Derived and Transformed Data 30
5.7.1. Baseline Age 30
5.7.2. Body Measurements Variable Derivation 30
5.7.3. Study Day 30
5.7.4. Change from Baseline 30
5.7.5. Summary Scores for Patient Reported Outcomes (PRO) 30
5.7.6. Analysis Windows 31
5.7.7. Multiple Assessments 31
5.7.8. Other Study Related Definitions 31
5.7.9. Derived Echocardiographic parameters 32
5.8. Handling of Missing Data 32
5.8.1. Missing Efficacy Endpoints 32
5.8.2. Missing Start and Stop Dates for Prior and Concomitant Medication 32
5.8.3. Missing Start and Stop Dates for Adverse Events 33
6. STUDY POPULATION 34

6.1. Subjects Disposition 34
6.2. Screen Failures....34
6.3. Protocol Deviations 34
6.4. Demographic and Baseline Characteristics 34
6.5. Listing of Subject Inclusion and Exclusion Criteria....35
6.6. Medical History 35
6.7. Baseline Medications Use....35
7. EFFICACY 36
7.1. General Considerations....36
7.2. Testing Statistical Assumptions Including Comparability at Baseline....36
7.3. Statement of the Null and Alternate Hypotheses....36
7.4. Planned Covariates 36
7.5. Subgroup Analyses 36
7.6. Multiple Comparisons and Multiplicity....37
7.7. Analysis of the Primary Efficacy Endpoint 40
7.7.1. Primary Efficacy Analysis....40
7.7.2. Sensitivity Analyses of the Primary Efficacy Endpoint 42
7.7.3. Subgroup Analyses for the Primary Endpoint....42
7.7.4. Supportive Analyses for the Primary Endpoint....42
7.8. Analysis of the Secondary Efficacy Endpoints 43
7.8.1. Analysis of the Secondary Efficacy Endpoints 43
7.9. Analysis of the Exploratory Efficacy Endpoints 51
7.9.1. Analysis of the Exploratory Efficacy Endpoint....51
8. SAFETY AND TOLERABILITY....53
8.1. Overall Summary of Tolerability....53
8.2. Adverse Event Preferred Term and Body/Organ System Summary Tables....53
8.2.1. Summaries of Adverse Event Incidence Rates for All Subjects....53
8.2.2. Summaries of Adverse Events of Special Interest....54
8.3. Total Duration of Therapy, Final Daily Dose of Study Medication, and Compliance....54
8.3.1. Summary of IP Exposure and Overall Compliance....54
8.3.2. Summary of Dose Titration 54
8.4. Concomitant and Other Medications....55

8.5. Routine Laboratory Data 55
8.6. Vital Signs 55
8.7. Electrocardiogram....56
9. PHARMACOKINETICS 58
10. REFERENCES 59
11. APPENDIX....60
11.1. Patient-reported Outcome Scoring Algorithm....60
11.1.1. KCCQ 60
11.1.2. SAQ-7 63
11.1.3. EQ-5D-5L 65
11.2. Table of Contents for Data Display Specifications 66
11.3. Data Display Specifications....66
11.4. Analysis Windows 67
11.5. Sample SAS Codes 67

LIST OF TABLES

Table 1: Investigational Products....20
Table 2: Echocardiographic Variables and Names 21
Table 3: CMR Parameters....24
Table 4: Testing Steps....38
Table 5: Estimands for Primary Endpoint 40
Table 6: Endpoint Summary Table 47
Table 7: EQ-5D-5L Value Set 65
Table 8: Analysis Windows for Measurements 67

LIST OF FIGURES

Figure 1: Statistical Testing Hierarchy for Primary and Secondary Endpoints 6

LIST OF ABBREVIATIONS

Abbreviation/Term	Explanation
AE	Adverse event
ALP	Alkaline phosphatase
ALT	Alanine aminotransferase
ANCOVA	Analysis of Covariance
AST	Aspartate aminotransferase
BSA	Baseline body surface area
CGI	Clinical Global Impression scale
CI	Confidence interval
CMH	Cochran–Mantel–Haenszel
CMR	Cardiac magnetic resonance
CPET	Cardiopulmonary exercise testing
CRF	Case report form
CSR	Clinical Study Report
CSS	Clinical Summary Score
cTTO	composite time trade-off
CV	Cardiovascular
CV%	Coefficient of Variation
ECG	Electrocardiogram
eCRF	Electronic Case Report Form
EQ-5D	EuroQol 5-dimension instrument
EQ-5D-5L	EuroQol 5-dimension 5-level instrument
EQ-VAS	EuroQol - Visual Analogue Scale
FAS	Full analysis set
HCM	Hypertrophic cardiomyopathy
hs-cTnI	High sensitivity cardiac troponin I
ICD	Implantable cardioverter defibrillators
ICF	Informed consent form
ICH	International Council for Harmonisation
ID	Identifier
IP	Investigational product
IWRS	Interactive web response system
KCCQ	Kansas City Cardiomyopathy Questionnaire
LLN	Lower Limit of Normal
LSM	Least Squares Mean
LV	Left ventricle(ular)

Abbreviation/Term	Explanation
LVEDV	Left ventricular end-diastolic volume
LVEF	Left ventricular ejection fraction
LVESV	Left ventricular end-systolic volume
LVOT	Left ventricular outflow tract
LVOT-G	Left ventricular outflow tract gradient
MAR	Missing At Random
MedDRA	Medical Dictionary for Regulatory Activities Terminology
MMRM	Mixed Model for Repeated Measures
MNAR	Missing Not At Random
NT-proBNP	n-terminal prohormone brain natriuretic peptide
NYHA	New York Heart Association
oHCM	Obstructive hypertrophic cardiomyopathy
PDCC	Protocol deviation classification committee
PGI-C	Patient Global Impression of Change scale
PK	Pharmacokinetics
PKS	Pharmacokinetics analysis set
PRO	Patient reported outcomes
PT	Preferred Term
pVO2	Peak oxygen uptake
QTcF	Fridericia corrected QT
RER	Respiratory exchange ratio
SAE	Serious Adverse Event
SAP	Statistical Analysis Plan
SAQ-7	Seattle Angina Questionnaire -7
SAS	Statistical Analysis System (SAS®)
SD	Standard deviation
SOC	System Organ Class
SRT	Septal Reduction Therapy
TEAE	Treatment-Emergent Adverse Event
TESAE	Treatment-Emergent Serious Adverse Event
TSS	Total Symptom Score
ULN	Upper Limit of Normal
VAS	Visual Analogue Scale
VAT	Ventilatory anaerobic threshold
WHO	World Health Organization

SAP VERSION HISTORY

Version and Date	Revision	Rationale
Final 1.0	Not applicable: original version	Not applicable

1. INTRODUCTION

The purpose of this statistical analysis plan (SAP) is to provide a technical elaboration of the planned analyses and detailed data displays to be included in the Clinical Study Report (CSR) for CY 6031 study.

This SAP was developed in accordance with International Council for Harmonisation (ICH) E9 and ICH E9 (R1) guideline. All decisions regarding final analysis, as defined in this SAP document, will be made prior to the study database lock. Further study information can be found in the protocol.

2. STUDY OBJECTIVES AND ENDPOINTS

2.1. Study Objectives

2.1.1. Primary Objective

To evaluate the effect of CK-3773274 on exercise capacity in patients with symptomatic obstructive hypertrophic cardiomyopathy (oHCM).

2.1.2. Secondary Objective

The secondary objectives of the study are listed as follows:

- To evaluate the effect of CK-3773274 on patient health status
- To evaluate the effect of CK-3773274 on New York Heart Association (NYHA) Functional Classification
- To evaluate the effect of CK-3773274 on post-Valsalva left ventricular outflow tract gradients (LVOT-G)
- To evaluate the effect of CK-3773274 on exercise capacity
- To evaluate the effect of CK-3773274 on duration of eligibility for SRT

2.2. Study Endpoints

2.2.1. Primary Endpoints

The primary endpoint of the study is Change in peak oxygen uptake (pVO2) by cardiopulmonary exercise testing (CPET) from baseline to Week 24.

2.2.2. Secondary Endpoints

- Change in Kansas City Cardiomyopathy Questionnaire – Clinical Summary Score (KCCQ-CSS) from baseline to Week 12 and Week 24
- Proportion of patients with ≥1 class improvement in NYHA Functional Class from baseline to Week 12 and Week 24
- Change in post-Valsalva LVOT-G from baseline to Week 12 and Week
- Proportion of patients with post-Valsalva LVOT-G <30 mmHg at Week 12 and Week 24
- Change in total workload during CPET from baseline to Week 24
- Total duration of SRT eligibility during the 24-Week treatment period in patients who were SRT eligible at baseline

2.2.3. Exploratory Endpoints

- Compared with baseline, number of patients at Week 24 achieving either:

  − Change from baseline of ≥1.5 mL/kg/min in pVO2 AND ≥1 class improvement in NYHA Functional Class

OR

− Change from baseline of ≥3.0 mL/kg/min in pVO2 AND no worsening of NYHA Functional Class

- Proportion of patients with improvement of >5, 10, 15 and 20 points in KCCQ-CSS and KCCQ- Total Symptom Score (TSS) at Weeks 12 and 24
- Proportion of patients with resting LVOT-G <30 mmHg, post-Valsalva LVOT-G <50 mmHg, and NYHA Functional Class I at Week 12 and Week 24
- Proportion of patients with resting LVOT-G <30 mmHg, post-Valsalva LVOT-G <50 mmHg, and ≥1 class improvement in NYHA Functional Class at Week 12 and Week 24
- Change from baseline to Week 24 in CPET parameters of:
    - Ventilatory efficiency (VE/VCO2 slope)
    - Circulatory power (VO2 × systolic BP)
    - Ventilatory anaerobic threshold (VAT)
- Proportion of patients who remain SRT eligible at Week 24 in patients who were eligible for SRT at baseline. Proportion of patients who remain SRT eligible will also be evaluated at other scheduled visit weeks
- Time to first SRT ineligibility status in patients who were SRT eligible at baseline
- Change from baseline to Week 24 in individual responses to the EuroQol 5-dimension 5-level instrument (EQ-5D-5L)
- Change from baseline to Week 24 in total score and domain scores for the Seattle Angina Questionnaire -7(SAQ-7)
- Change from baseline to Week 24 in echocardiographic measurements of cardiac structure and of systolic function including:
    - Left ventricular ejection fraction (LVEF)
    - Left ventricular global longitudinal strain (LV GLS)
    - Left ventricular end-systolic and end-diastolic volumes (LVESV and LVEDV)
    - Left atrial volume index
    - Left ventricular mass index
    - Maximal wall thickness
- Change from baseline values in n-terminal prohormone brain natriuretic peptide (NT-proBNP), high sensitivity cardiac troponin I (hs-cTnI) and other biomarkers through Week 24; proportional change (post randomization/baseline) will also be calculated and used in the analysis
- Change from baseline to Week 24 in patients enrolled in Cardiac magnetic resonance (CMR) substudy in CMR measurements of:

- Left ventricular (LV) mass index
- LVEF
- Septal, free wall and maximal wall thickness
- Left atrial volume index
- LVESV
- LVEDV
- Extracellular volume proportion
- Late gadolinium enhancement proportion
- Mitral regurgitation severity

- Time to Maximal ST Segment Depression on CPET ECG
- Time to 1 mm ST depression below Baseline on CPET ECG
- Maximal ST segment depression on resting ECG in mm at Weeks 12 and 24
- Number of patients with new or worsening ST depression during exercise at Weeks 12 and 24
- Change from baseline values in all other summary KCCQ scores (Physical Limitation, Symptom Stability, Symptom Frequency, Symptom Burden, Total Symptom Score, Self-efficacy, Quality of Life, Social Limitation, Overall Summary Score) at Weeks 12 and 24.
- Proportion of patients with LVH with strain pattern (typical+ atypical) on Electrocardiogram (ECG) at Weeks 12 and 24
- Proportion of patients with all LVH (with or without strain) on ECG at Weeks 12 and 24

2.2.4. Safety Endpoints

- Incidence of reported major adverse cardiac events (cardiovascular [CV] death, cardiac arrest, non-fatal stroke, non-fatal myocardial infarction, CV hospitalization)
- Incidence of new onset persistent atrial fibrillation
- Incidence of appropriate implantable cardiac defibrillator (ICD) discharges and aborted sudden cardiac death
- Incidence of LVEF <50% with at least one of the following:
  - Signs and symptoms of heart failure (concomitant adverse event of heart failure or dyspnea)

    AND/OR

  - Increase in NT-proBNP from baseline

  Signs and symptoms referring to AEs with onset date within ± 7 days relative to the date when LVEF <50%.
- Incidence of LVEF <40%

- Incidence of LVEF <50%

Incidence of LVEF below 40% and 50% will be summarized for site read, core lab read and both.

- Incidence of treatment emergent adverse events (TEAEs)

2.2.5. PK Parameters

- $C_{post\ dose}$ and $C_{pre\text{-}dose}$

3. STUDY DESIGN

3.1. Summary of Study Design

This is a Phase 3, randomized, placebo-controlled, double-blind, multi-center trial in patients with symptomatic oHCM. Approximately 270 eligible patients will be randomized in a 1:1 ratio to receive CK-3773274 or placebo. Randomization will be stratified by use of beta-blockers (yes or no) and CPET exercise modality (treadmill or bicycle) and implemented in the Interactive Web Response System (IWRS). A cap on the number of patients taking beta-blockers and will not exceed approximately 70% of total enrollment. The number of patients taking disopyramide will be capped at approximately 10% of total enrollment. The number of patients with persistent atrial fibrillation at screening will also be capped at approximately 15%, and the number of patients using the bicycle CPET exercise modality will be capped at approximately 50% as well.

Investigational product (IP) will be administered orally once daily with or without food. During the initial six weeks of the treatment period, IP doses will be individually titrated at Weeks 2, 4, and 6 using echocardiography. Dose escalation at Weeks 2, 4, and 6 will occur only if a patient has a post-Valsalva LVOT-G ≥30 mmHg and a biplane LVEF ≥55%. Echocardiograms will be performed at each subsequent visit during the trial and the dose down titrated if necessary. The primary endpoint of pVO2 will be measured by CPET at screening and at end of treatment (Week 24). If applicable, patients will continue taking background HCM medications consistent with regional clinical practice guidelines during the trial.

3.2. Definition of Study Drugs

Table 1 describes any study drug: IP (ie, aficamten) or placebo intended to be administered to a trial patient according to the protocol.

Table 1: Investigational Products

	Active	Placebo
**IP/Product Name**	CK-3773274	Placebo
**Type**	Drug	Drug
**Dose Formulation**	Tablet	Tablet
**Unit Dose Strength(s)**	5 mg	Matching placebo
**Dosage Level(s)**	5mg, 10mg, 15mg, 20 mg	
**Route of Administration**	Oral	Oral
**Use**	Experimental	Placebo
**IMP and NIMP**	IMP	IMP
**Packaging and Labeling**	IP will be provided in blister packs which will be labeled as required per country requirement	IP will be provided in blister packs which will be labeled as required per country requirement

3.3. Sample Size Considerations

3.3.1. Sample Size Justifications

Assuming a difference in change from baseline in pVO2 of 1.5 mL/kg/min for CK-3773274 compared to placebo, a standard deviation (SD) of 3.5 mL/kg/min, accounting for limiting beta-

blocker use (less than ~70%), limiting exercise modality of bicycle (less than ~50%) and 10% of patients missing change from baseline data of the primary endpoint, a sample size of 270 patients at randomization ratio of 1:1 (approximately 135 randomized to CK-3773274 and 135 randomized to placebo) provides more than 90% power to detect the difference in pVO2 change from baseline to Week 24 with a 2-sided type I error of 0.05.

During the study, Cytokinetics will periodically assess in a blinded fashion the aggregate pooled missing data rate and overall pooled SD for the change from baseline in pVO2 at Week 24. If the pooled SD is larger than expected, Cytokinetics may consider increasing the sample size once in order to maintain the intended power.

3.4. Randomization

All eligible patients will be centrally assigned to randomized IP using the IWRS. Randomization will be stratified by use of beta-blockers (yes or no) and CPET exercise modality (treadmill or bicycle) and implemented in the IWRS.

3.5. Clinical Assessments

3.5.1. Efficacy Assessments

Efficacy assessments include CPET, echocardiography, NYHA classification, patient-reported outcomes (KCCQ, EQ-5D-5L), Patient Global Impression of Change scale (PGI-C) and SAQ-7, and clinical global impression scale (CGI). CMR measurements will be assessed in patients included in CMR sub-study.

3.5.1.1. Echocardiography

Echocardiography will be done during screening, prior to dosing on Day 1, and 2 hours after dosing in the clinic on Weeks 2, 4, 6, 8, 12, 16, 20, 24, and 28.

Site read echocardiographic assessments include LVEF, resting and Valsalva LVOT at each visit. Unless otherwise specified, echocardiographic variables will be based on the core echocardiography laboratory assessments. Table 2 below lists the echocardiography parameters from core lab. A full list of echocardiography parameters is specified in the data transfer agreement from core lab.

Table 2: Echocardiographic Variables and Names

**Endpoint Names**
**Variables Describing LV Structure**
Left ventricular end diastolic diameter
Left ventricular end diastolic volume Index
Left Ventricular End Systolic Volume Index
left ventricular end systolic diameter
Left Ventricular Posterior Wall Thickness, End-diastole
LV Mass indexed

Table 2: Echocardiographic Variables and Names (Continued)

**Endpoint Names**
Interventricular Septum Thickness, End-Diastole
Left ventricular Maximal wall thickness
Left ventricular relative wall thickness
**Variable Describing LV Systolic Function**
Left ventricular ejection fraction
Left ventricular fractional shortening
Left ventricular stroke volume Index
Left ventricular cardiac output Index
Left Ventricular Isovolumetric Contraction Time
Left Ventricular Isovolumetric Relaxation Time
Left Ventricular Ejection Time
Left Ventricular Myocardial Performance Index
Left Ventricular Outflow Tract Velocity Time Integral
Left Ventricular Global Longitudinal Strain
Left Ventricular Global Circumferential Strain
Variables Describing LV Diastolic Function
Peak E Wave Velocity
Peak A Wave Velocity
Mitral Lateral Annular Early Diastolic Velocity
Mitral Septal Annular Early Diastolic Velocity
Mitral E/A Wave Velocity Ratio
Mitral E Wave to Lateral Annular Early Diastolic Velocity Ratio
Mitral E Wave to Septal Annular Early Diastolic Velocity Ratio
**LVOT Dynamic Gradient Assessment Variables**
Peak Left Ventricular Outflow Tract Pressure Gradient at Rest
Peak Left Ventricular Outflow Tract Pressure Gradient during Valsalva Maneuver
**LV Cavity Acceleration Assessment Variables**
Time from Left Ventricular Ejection Onset to 40 cm/sec Velocity
Time from Left Ventricular Ejection Onset to 60 cm/sec Velocity
Left Ventricular Acceleration Time
Left Ventricular Acceleration Time/Ejection Time Ratio

Table 2: Echocardiographic Variables and Names (Continued)

**Endpoint Names**
Variables Describing LA Size and Function
Left Atrial Width
Left Atrial Volume Index
**Variables Describing RV Size and Function**
Right Ventricular Outflow Tract Velocity Time Integral
Right Ventricular Myocardial Performance Index
Tricuspid annular plane systolic excursion
**Valvular Assessment Variables**
Presence of Mitral Regurgitation
Mitral Regurgitation Jet Area to Left Atrial Area Ratio
Presence of Mitral Systolic Anterior Motion

3.5.1.2. Patient-Reported Outcomes

KCCQ and EQ-5D-5L will be assessed at Day 1, Weeks 2, 4, 6, 8, 12, 16, 20, 24 and 28 (4 weeks after last dose at end of the study). SAQ-7 will be assessed at Day 1, Weeks 4, 8, 12, 16, 20, 24 and 28. PGI-C will be assessed at the Week 24. Algorithms to derive the scores KCCQ, EQ-5D-5L and SAQ-7 are in Section 11.1.

EQ-5D-5L and EQ-VAS

The instrument EQ-5D is a standardized measure of health status for clinical and economic appraisal (EuroQol Group 1990), which consists of two parts: a short descriptive system questionnaire (EQ-5D-3L) and a visual analogue scale (EQ-VAS).

EQ-5D-5L is the 5-level version of EQ-5D introduced to improve the instrument's sensitivity and to reduce ceiling effects (EuroQol Group 2009). The descriptive system comprises 5 dimensions: mobility, self-care, usual activities, pain/discomfort and anxiety/depression. Each dimension has 5 levels indicating no problems, slight problems, moderate problems, severe problems or extreme problems. Five responses with a response from each of the 5 dimensions form a 5-digit number that defines a patient's health state profile. A health state can potentially be assigned a summary index score based on societal preference weights (societal perspective) for the health state. The health state preferences often represent national or regional values and can therefore differ between countries/regions. The health state index scores will be calculated using the composite time trade-off (cTTO) method based on the United States valuation of EQ-5D-5L (Pickard 2019) for patients from the United States and for the FAS. The health state index score ranges from less than 0 to 1 with higher scores indicating higher health utility; a score 0 represents death, negative values represent worse than death, and 1 represents full health.

EQ-VAS rates a patient's perceived heath on a vertical visual analogue scale from 0 to 100, where 0 represents the worst imaginable health and 100 represents the best imaginable health.

The VAS can be used as a quantitative measure of health outcome that reflects the patient's own judgement.

3.5.1.3. CMR Assessments

A CMR imaging sub-study will assess the effects of administration of CK-3773274 dosing on cardiac morphology, function, and fibrosis in approximately 100 oHCM patients who are eligible and consent to participate. CMR will be performed during screening period and Week 24. CMR parameters are listed below in Table 3 and a final list of CMR parameters is specified in the data transfer agreement from core lab.

Table 3: CMR Parameters

**LV Parameters**
LVM - Left Ventricular Mass
LVMI Left Ventricular Mass Index
LVEDV - Left Ventricular End Diastolic Volume
LVEDVI - Left Ventricular End Diastolic Volume Index
LVESV - Left Ventricular End Systolic Volume
LVESVI - Left Ventricular End Systolic Volume Index
LVSV– Left Ventricular Stroke Volume=LVEDV-LVESV
LVSVI = Left Ventricular Stroke Volume Index=LVSV/Body Surface Area (mm2)
LVEF - Left ventricular Ejection Fraction
MR – Mitral Regurgitation
CO - Cardiac Output = Heart Rate x Stroke Volume
CI - Cardiac Index = Heart Rate x Stroke volume/Body Surface Area (m2)
LV maximal septal wall thickness
LV maximal lateral wall thickness
LV maximal wall thickness (each of 16 segments)
Overall LV maximal wall thickness (highest across all 16 segments)
LGE mass (g) (Global mass of LGE 6SD)
LGE mass % (as % of LV mass) (Global percent of LGE 6SD)
Global average ECVF
LV Segmental assessment of ECVF
Global Native T1
Extracellular volume (ECV)
Global ECV mass
Global ECV mass index

Table 3: CMR Parameters (Continued)

**LV Parameters**
ECVF normalized for height
HCM morphology (isolated basal septal hypertrophy, reverse septal curvature, apical, midcavity obstruction without apical aneurysm, midcavity obstruction with apical aneurysm, concentric, other
**RV Parameters**
RV end-diastolic volume (RVEDV)
RV end-diastolic volume index (RVEDVI)
RV end-systolic volume (RVESV)
RV end-systolic volume index (RVESVI)
RV stroke volume (RVSV)
RV stroke volume index (RVSVI)
RV ejection fraction (RVEF)
**LA parameters**
LA volume maximum
LA volume maximum index
LA reservoir amount
LA reservoir percent
LA contractile amount
LA contractile percent
LA total amount
LA total percent
LA global longitudinal strain
**Mitral Valve Parameters**
Exploratory Mitral Valve Regurgitation Measurements
Mitral regurgitation volume
Mitral regurgitation, regurgitation fraction

3.5.1.4. CPET Assessments

All patients will undergo CPET with gas-exchange analysis and the methodology will be standardized across all sites as specified in the CPET manual. Patients must use the same testing modality for all exercise tests during the trial. CPET are done at baseline and Week 24 post randomization. CPET parameters are listed below. A full list of CPET parameters is specified in the data transfer agreement from core lab.

- Workload

- Exercise Duration
- % of Predicted Oxygen Uptake
- Circulatory Power
- Predicted Oxygen Uptake
- Peak Oxygen Uptake per Kilogram
- Peak RER
- Oxygen Uptake Efficiency Slope
- Ventilatory Efficiency
- Anaerobic Threshold
- Aerobic Efficiency

3.5.2. Safety Assessments

Safety assessments include adverse events and serious adverse events (SAEs), ICD discharge, LVEF < 50% and <40%, electrocardiograms, laboratory assessments, physical examinations, and vital signs.

3.5.3. Pharmacokinetics Assessments

Blood samples will be collected to evaluate plasma concentrations of CK-3773274 at pre-dose and 2 hours post-dose at Day 1, Weeks 2, 4, 6, 8, 12, 16, 20 and 24.

4. PLANNED ANALYSES

4.1. Interim Analyses

No interim analysis is planned for this study.

4.2. Final Analyses

The final analysis will occur after all patients randomized in the study have completed the study including the 4-week safety follow up, all data has been entered into the clinical database, verified, and locked. Unblinding for the final analysis will occur after the database lock.

5. GENERAL CONSIDERATIONS FOR DATA ANALYSES AND HANDLING

5.1. General Summary Table and Individual Subject Data Listing Considerations

Descriptive statistics to be presented in a table include number of observations, mean, median, standard deviation, 1st and 3rd quartiles, minimum and maximum for continuous variables, and count of patients and the percentage for categorical variables. For variables that lognormal distribution assumptions may be appropriate geometric mean, and geometric coefficient of variation (CV%) will also be displayed. Geometric CV (%) will be derived as $100\% \times \text{sqrt}(\exp(s^2)-1)$, where s is the standard deviation of the natural logarithm (ln) transformed data.

For model-based analysis, least squares means (LSM), difference of least squares means between treatments, their standard errors and 95% confidence intervals (CI), and two-sided p-values for the statistical inferences will be presented.

Selected listings may be generated to include patient identifier (ID), demographics, randomized treatment group and other relevant items, and sorted by randomized treatment group, patient ID and date of assessment.

5.2. General Post Text Summary Table and Individual Subject Data Listing Format Considerations

Post text tables and individual subject data listings are prepared according to ICH Guideline E3. In general, summary and analysis tables will be presented by treatment groups and highest dose level administered.

5.3. Data Management

Data will be entered into the clinical database with programmed edit checks and manual data review to ensure integrity. The data will be reviewed and cleaned according to a data management plan. Clinical safety laboratory, ECG, Pharmacokinetics (PK) data, CMR, CPET and echocardiography will be provided per the pre-specified data transfer agreement from external laboratories.

5.4. Data Presentation Conventions

The following conventions will be applied to data presentations:

- For continuous variables, mean and median values are formatted to one more decimal place than the measured value. Standard deviation values are formatted to two more decimal places than the measured value. Minimum and maximum values are presented with the same number of decimal places as the measured value. For the statistical analyses results that are on the same scale as a measured value (e.g., change from baseline or treatment difference estimates), the LSM estimates and LSM estimate 95% CI boundary values will be formatted to one more decimal place than the measured value; Standard error of the mean (SEM) estimates will be formatted to two more decimal

places than the measured values. GLSM estimates for the proportional change from baseline, proportional change treatment ratios, odds ratios, and the corresponding 95% CIs will be presented with two decimal places.

- For categorical variables, the count and percentage of responses are presented in the form XX (XX.X%) where the percentage is in the parentheses.
- Date variables are formatted as YYYY-MM-DD for presentation. Time is formatted in military time as HH:MM for presentation.
- P-values, if applicable, will be presented to 3 decimal places. If the p-value is less than 0.0001 then it will be presented as <0.0001. If the rounded result is a value of 1.000, it will be displayed as >0.999.
- Unless otherwise stated, any statistical tests performed will use 2-sided tests at the 0.05 significance level.

The table and listing shells and table of contents provide the expected layout and titles of the tables, listings and figures. Any changes to format, layout, titles, numbering, or any other minor deviation will not necessitate a revision to the SAP, nor will it be considered a deviation from planned analyses. Only substantial deviation in the analysis methods will require an SAP revision or a change to planned analysis documented in the CSR.

5.5. Analysis Populations

5.5.1. All Screened Patients

All patients who signed the informed consent form (ICF) are included in the All Screened Patients Set. Patients who gave informed consent but are not randomized are considered screen failures. The following reasons for screen failures are collected: inclusion/exclusion criteria (including specific criteria not met), principal investigator decision, subject decision, lost to follow up, other. For patients who are screen failures, the reasons for failing will be summarized.

5.5.2. All Randomized Set

All Randomized Set includes patients who are randomized to receive CK-3773274 or placebo.

5.5.3. Safety Analysis Set

Safety analyses will be performed on the safety analysis set (SAF), which includes all randomized patients who receive at least one dose of IP, CK-3773274 or placebo. Unless otherwise specified, for safety analyses, subjects will be grouped according to their randomized treatment group assignment with the following exception: if a subject receives treatment throughout the study that is different than the randomized treatment group assignment, then the subject will be grouped by the actual treatment group.

5.5.4. Full Analysis Set

Efficacy analyses will be performed on the full analysis set (FAS), which includes all randomized patients who receive at least one dose of IP and have at least one post-baseline

efficacy measurement. Patients will be analyzed according to their randomized treatment group assignments.

5.5.5. Pharmacokinetics Analysis Set

All randomized patients who have at least one evaluable plasma concentration of CK-3773274, provided they have no major protocol deviations that could affect the PK of CK-3773274.

5.6. Baseline Definition

Unless otherwise specified, baseline is defined as the last available measurement taken prior to administration of the first dose of study drug. Baseline for KCCQ, EQ-5D-5L and SAQ-7 are assessments performed on Day 1. The assessments collected on the same date as the first dose of the IP that do not have assessment time are considered to have occurred prior to the first dose.

5.7. Derived and Transformed Data

5.7.1. Baseline Age

Age will be calculated as follows:

Age (years) = year of screening date – year of birth,

Patient age will be categorized as < 65 years or ≥ 65 years.

5.7.2. Body Measurements Variable Derivation

Baseline body surface area (BSA) will be calculated using the weight and height at screening using the DuBois and DuBois formula and rounded to two decimal points for the presentation of results:

BSA (m^2) = 0.007184 * (weight (kg) $^{0.425}$ * height (cm) $^{0.725}$).

5.7.3. Study Day

If the date of interest occurs on or after the first dose date, then study day will be calculated as (date of interest – date of first dose) + 1. If the date of interest occurs prior to the first dose date, then study day will be calculated as (date of interest – date of first dose). There is no study day 0.

5.7.4. Change from Baseline

Change from baseline is calculated as (post baseline value – baseline value).

Percent change from baseline is calculated as (change from baseline / baseline value) x 100%.

Proportional change from baseline is calculated as (post-baseline result/baseline value).

If either the baseline or the post-baseline value is missing, the change from baseline and percentage change from baseline will be set to missing.

5.7.5. Summary Scores for Patient Reported Outcomes (PRO)

Calculations of summary scores for KCCQ, SAQ-7 and EQ-5D-5L are specified in Appendix Section 11.1.

5.7.6. Analysis Windows

Since study visits do not always take place exactly as scheduled per protocol, it is necessary to assign the actual observation dates to analysis windows for analysis purposes.

For data collected at a scheduled post baseline visit, the analysis window will be assigned based on the scheduled study day of the nominal visit as collected on the eCRF.

For unscheduled or early discontinuation post baseline visits, measurements taken on or after the first dose of study drug will be assigned to an analysis window using defined lower and upper bounds for each analysis window. Measurements assigned in an analysis window will have study day greater than or equal to the lower bound but no greater than the upper bound of the analysis window. The lower bound and the upper bound for the analysis windows are defined as the midpoints between the scheduled visits for all assessments (see Section 11.4).

Visits are identified as the nominal visits according to the eCRFs. Each visit will be identified with the visit descriptor (eg, “Week 24”).

5.7.7. Multiple Assessments

Once analysis windows are assigned, a patient’s individual analysis window could potentially contain more than one visit. Records from all visits, including scheduled, unscheduled and early discontinuation visits could be flagged as the “analyzed record” within the analysis window, although the records from scheduled visit will take priority.

In the event of multiple visits falling within an analysis window, the following rules will be used in sequence to determine the “analyzed record” for the analysis window:

- If a scheduled visit occurred during the analysis window, then the measurement taken from the scheduled visit will be used.
- If no scheduled visit occurred during the analysis window, the measurement taken closest to the scheduled day will be used as the “analyzed record.”
- If no scheduled visit occurred during the analysis window and there is a tie between unscheduled visits in the number of days before and after the scheduled day, measurements from the later visit will be used as the “analyzed record.”

For analyses by visit, only the “analyzed record” within each analysis window and the visit will be summarized in a table. Only protocol specified visits will be presented in the summary table. If there are other visit records within the analysis window, they will only be included in data listings.

5.7.8. Other Study Related Definitions

<u>Actual Dose Group</u>

Patients in the aficamten actual treatment group will be identified as 5 mg, 10 mg, 15 mg, 20mg or discontinuing IP prior to dose adjustment based on the dose assigned at Week 8. If a patient discontinues IP prior to the start of Week 8 (IWRS Week 8 dispensation), then the subject will be identified as discontinued IP prior to achieve stable dose. The actual dose group may be used in selected displays.

Investigational Product Exposure Period
For subjects dosed with IP:
[(Last IP administration date – date of Study Day 1) + 1]/7 (in weeks)
Interruptions recorded in the eCRF page will be excluded from the expected dose calculation.

Treatment-emergent Adverse Event

For the purpose of reporting, an investigator-reported event starting on or after first dose of IP and up to and including 28 days after the last dose date of IP will be labeled as a treatment-emergent AE.

Last Titrated Dose

Last titrated dose is defined as the last titrated dose assigned to the patient during the 24 Week treatment period.

5.7.9. Derived Echocardiographic parameters

The following BSA-indexed variables will be derived using the baseline BSA defined in Section 5.7.2:

- LVEDV-I (mL/m^2) = LVEDV/BSA
- LVESV-I (mL/m^2) = LVESV/BSA
- LVSVI (mL/m^2) = LVSV/BSA
- LAV-I (mL/m^2) = LAV/BSA
- LVCO-I (mL/min/m^2) = LVCO/BSA
- LVmass-I(g/m^2) = LVmas/BSA

For the presentation of results, the BSA-indexed variables will be rounded to the same number of decimal places as the corresponding non-indexed variables provided by the echocardiography core laboratory.

5.8. Handling of Missing Data

5.8.1. Missing Efficacy Endpoints

For the primary endpoint, missing data will be imputed under missing at random (MAR) assumption and all observed and imputed missing pVO_2 assessments will be included in the primary analysis of the primary endpoint. Missing secondary CPET endpoints will be handled the same as for the primary endpoint. Missing response for patient reported outcomes will be handled as described in Section 11.1.

5.8.2. Missing Start and Stop Dates for Prior and Concomitant Medication

To classify medications as baseline use or concomitant, missing start and stop dates of medications will be imputed as follows:

- If the medication start date day is missing, it will be imputed with the first of the month,

- If the medication start date day and month are missing, they will be imputed with 01 January,
- If the medication stop date day is missing, it will be imputed with the last day of the month or the date of the last contact with the patient, with the imputed date doesn't exceed the date of last contact,
- If the medication stop date day and month are missing, they will be imputed with 31 December or the date of the last contact with the patient, with the imputed date doesn't exceed the date of last contact,
- For the ongoing medications, the stop date will not be imputed.

5.8.3. Missing Start and Stop Dates for Adverse Events

For AEs with incomplete date information recorded in the eCRF, the imputation will follow the following algorithm:

For missing AE onset Date

- If an AE onset Day is missing and the Month of AE onset is known, then the first day of the month of AE onset will be imputed as the AE onset date. If the month and year of AE onset are the same as month and year of the first dosing, the missing day will be imputed as the first dosing date.
- If AE onset information is not available, then the first dosing date will be imputed as the AE onset date.
- If AE onset day and month are both missing, the missing month and day will be imputed as 01 January. If the year of AE onset is the same as the first dosing date, the AE onset will be imputed as the first dosing date.

For missing AE end Date:

- If the AE end Day is missing and it will be imputed with the last day of the month or the date of the last contact with the patient, with the imputed date doesn't exceed the date of last contact.
- If the AE end date day and month are missing, they will be imputed with 31 December or the date of the last contact with the patient, with the imputed date doesn't exceed the date of last contact.
- For the ongoing AEs, the stop date will not be imputed.

6. STUDY POPULATION

6.1. Subjects Disposition

Patient disposition will be summarized based on all randomized patients. The following will be summarized:

- The number and percentage of patients who completed the study and the number of patients who discontinued from the study early,
- For the patients who discontinued from the study early, reasons for early discontinuation,
- The number and percentage of patients who received at least one dose of the IP,
- For the patients who received at least one dose of the IP, the number and percentage of patients who completed study treatment and the number of patients who discontinued the study treatment early,
- For the patients who discontinued the study treatment early, reasons for early discontinuation.

The number and percentage of randomized patients included in each analysis set will be summarized. Reasons for exclusion from analysis sets will be listed.

6.2. Screen Failures

Screen failures will be listed and summarized by reasons of screening failure.

6.3. Protocol Deviations

Major protocol deviation are reviewed and confirmed by the protocol deviation classification committee (PDCC) during the protocol deviation reviews throughout the study prior to database lock. Major protocol deviations are a subset of protocol deviations that may significantly impact the completeness, accuracy, and/or reliability of the study data or that may significantly affect a subject's rights, safety, or well-being. E.g., of major protocol deviations are

- patients who entered the study even though they did not satisfy the entry criteria.
- patients who developed withdrawal criteria during the study but were not withdrawn.
- patients who received the wrong treatment or incorrect dose.
- patients who received an excluded concomitant treatment.

Number of patients with reported important protocol deviation will be summarized by treatment group for all randomized subjects. A summary of protocol deviations due to COVID-19 will be provided separately.

6.4. Demographic and Baseline Characteristics

Demographic and baseline characteristics, including age, age group [< 65, ≥ 65], sex, race, ethnicity, height, weight, BMI, BSA, randomization stratification variables and baseline disease characteristics will be summarized by randomized treatment group for the FAS using descriptive

statistics. Summary may be repeated for all randomized patients. All randomized patients will be included in the listing of demographic and baseline characteristics.

6.5. Listing of Subject Inclusion and Exclusion Criteria

A listing of randomized patients who did not meet the inclusion and exclusion criteria will be provided.

6.6. Medical History

Medical history will be summarized by treatment received for the Full Analysis Set. HCM-related medical history will be summarized, including time since initial diagnosis and number and percentage of patients meeting the oHCM criteria.

Select cardiovascular medical history and other medical history will be summarized by the Medical Dictionary for Regulatory Activities Terminology (MedDRA) system organ class (SOC) and preferred term (PT).

6.7. Baseline Medications Use

Medications will be coded using World Health Organization (WHO) Drug Dictionary. Baseline medication use is defined medications that starts before the first dose of IP and ends after the first dose of IP or ongoing. The count and percentage of patients with each medication history item will be presented by therapeutic class (Anatomical Therapeutic Chemical [ATC] Class 3) and preferred name. If ATC Class 3 is not available, ATC Class 2 will be used in the summary.

7. EFFICACY

7.1. General Considerations

Efficacy analyses will be performed in the FAS by the randomized treatment group. Unless otherwise specified, all hypothesis tests will be reported as 2-sided p-values. Exploratory endpoints and subgroup analyses will be assessed using a nominal alpha level of 0.05 and will not have multiplicity adjustments. In order to preserve an overall type I error rate for the primary and secondary endpoints testing of the primary and secondary endpoints will follow the testing procedures specified in Section 7.6 .

7.2. Testing Statistical Assumptions Including Comparability at Baseline

The primary endpoint will be analyzed using an analysis of covariance (ANCOVA) model. Model assumption will be assessed by graphical examination of residuals. If assumptions are substantially violated, rank based analysis will be performed. See Section 7.7.4 for more details.

7.3. Statement of the Null and Alternate Hypotheses

The null hypothesis for the primary endpoint is that the treatment difference (aficamten – placebo) of mean change from baseline of pVO_2 at Week 24 is 0 and the alternative hypothesis is that the treatment difference is > 0 (favors aficamten). The tests will be reported with two-sided p-values, but only the direction favoring aficamten direction will be considered success.

7.4. Planned Covariates

Baseline covariates include but are not limited to the stratification factors and baseline measurements. For CPET related endpoints, covariates age, sex, baseline weight will also be evaluated. See Section 7.7.4 for details evaluating covariates effect in the primary endpoint.

7.5. Subgroup Analyses

Subgroup analyses with relatively moderate sample size will be performed to examine the consistency of the observed treatment effect and to gain insight into the effectiveness of aficamten in subpopulations. Analyses of the primary endpoint will be conducted for the following subgroups:

- Sex (male, female)
- Age group (< 65, ≥ 65 years old)
- Baseline body mass index (<30 vs. ≥ 30)
- Baseline NYHA Class (II, III)
- Baseline KCCQ CSS (≤ median and > median)
- Baseline LVEF (≤ median and > median)
- Baseline N-terminal prohormone brain natriuretic (NT-proBNP) (≤ median and > median)
- CPET modality (treadmill, bicycle)

- Baseline pVO_2 (≤ median, > median)
- Beta Blocker (use, no use)
- Baseline resting LVOT (≤ median and > median)
- Sarcomeric gene mutation status (pathogenic or variant of uncertain significance, and non-disease causing or none)

Subgroup analysis will be performed by including the subgroup effect and subgroup by treatment interaction to the model. The subgroup analysis will be performed based on the imputed dataset generated for the primary analysis of the endpoint if the primary analysis is based on multiple imputation.

In addition, subgroup of IND site status (IND sites vs. non-IND sites) analysis will be performed for the primary estimand only.

Analyses on the primary and secondary endpoints in patients who were SRT eligible at baseline may be explored.

7.6. Multiple Comparisons and Multiplicity

The null hypothesis for the primary and secondary efficacy variables in the FAS will be tested in the pre-specified order using a closed testing procedure.

For preservation of the overall type I error rate at two-sided 0.05 for the primary and secondary endpoints, the primary endpoint is tested first at two-sided 0.05. If the primary endpoint achieves statistical significance at two-sided alpha level of 0.05, then a parallel gatekeeper method with two-sided 0.025 separately allocated to Week 12 and to Week 24 is applied for the first four secondary endpoints, with their testing being in the sequential order of KCCQ-CSS change from baseline, proportion of patients with >1 NYHA functional class improvement, post-Valsalva LVOT-G change from baseline and proportion of patients with post-Valsalva LVOT-G < 30 mm Hg. If all four of the first four secondary endpoints at Week 12 (or at Week 24) have two-sided p≤0.025, then there is recycling of the 0.025 for Week 12 (or Week 24) to Week 24 (or Week 12) so that 0.05 is applicable to the corresponding testing of the first four secondary endpoints. If p≤0.025 for the first four secondary endpoints at Week 12 (or at Week 24) and if p≤0.05 for the first four secondary endpoints at Week 24 (or at Week 12), then the fifth secondary endpoint is tested at two-sided p≤0.05. If the fifth secondary endpoint achieves statistical significance at two-sided of alpha 0.05 in FAS then the last secondary endpoint will be tested at two-sided alpha level of 0.05.A final determination of the order of fifth and sixth secondary endpoints will be made once the trial is fully enrolled. The total duration of SRT eligibility over the 24-week of treatment in patients who were SRT eligible at baseline might be brought up to the 5th secondary endpoint if the proportion of patients with baseline NYHA III is determined to be approximately 30% or greater. The final decision will be made before the database lock and documented in the SAP. The multiple testing procedure is illustrated in Figure 1. The testing steps are described as follows in Table 4:

Table 4: Testing Steps

Step1	The null hypothesis for the primary endpoint is that there is no treatment difference in the change from baseline to Week 24 in pVO_2 between patients randomized to placebo and those randomized to aficamten in the FAS. The hypothesis will be tested at the two-sided significance level of 0.05. If this hypothesis is rejected, testing will proceed to Step 2; otherwise testing will stop.	
Step 2	The null hypothesis for the first secondary endpoint is that there is no treatment difference in the change from baseline to Week 12 in KCCQ-CSS in the FAS. The hypotheses will be tested at the two-sided significance level of 0.025. If this hypothesis is rejected, testing will proceed to Step 3; otherwise testing will stop	The null hypothesis for the first secondary endpoint is that there is no treatment difference in the change from baseline to Week 24 in KCCQ-CSS in the FAS. The hypotheses will be tested at the two-sided significance level of 0.025. If this hypothesis is rejected, testing will proceed to Step 3; otherwise testing will stop
Step 3	The null hypothesis for the second secondary endpoint is that there is no treatment difference in proportion of patients with >1 NYHA functional class improvement at Week 12 in the FAS. The hypotheses will be tested at the two-sided significance level of 0.025. If this hypothesis is rejected, testing will proceed to Step 4; otherwise testing will stop.	The null hypothesis for the second secondary endpoint is that there is no treatment difference in proportion of patients with >1 NYHA functional class improvement at Week 24 in the FAS. The hypotheses will be tested at the two-sided significance level of 0.025. If this hypothesis is rejected, testing will proceed to Step 4; otherwise testing will stop
Step 4	The null hypothesis for the third secondary endpoint is that there is no treatment difference in change from baseline to Week 12 in post-Valsalva LVOT-G in the FAS. The hypotheses will be tested at the two-sided significance level of 0.025. If this hypothesis is rejected, testing will proceed to Step 5; otherwise testing will stop	The null hypothesis for the third secondary endpoint is that there is no treatment difference in change from baseline to Week 24 in post-Valsalva LVOT-G in the FAS. The hypotheses will be tested at the two-sided significance level of 0.025. If this hypothesis is rejected, testing will proceed to Step 5; otherwise testing will stop

Table 4: Testing Steps (Continued)

Step 5	The null hypothesis for the fourth secondary endpoint is that there is no treatment difference in proportion of patients with post-Valsalva LVOT-G < 30 mm Hg at Week 12 in the FAS. The hypotheses will be tested at the two-sided significance level of 0.025.<br>If this hypothesis is rejected, testing of Step 2 to Step 5 at Week 24 can be performed at two-sided alpha level of 0.05.	The null hypothesis for the fourth secondary endpoint is that there is no treatment difference in proportion of patients with post-Valsalva LVOT-G < 30 mm Hg at Week 24 in the FAS. The hypotheses will be tested at the two-sided significance level of 0.025. If this hypothesis is rejected, testing of Step 2 to Step 5 at Week 12 can be performed at two-sided alpha level of 0.05.
Step 6[1]	Only when all testing at Step 2 – Step 5 are rejected can testing of Step 6 be performed.<br>The null hypothesis for the fifth secondary endpoint is that there is no treatment difference in change from baseline to Week 24 in total workload in the FAS. The hypothesis will be tested at the two-sided significance level of 0.05.	
Step 7	Only when null hypothesis at Step 6 is rejected can testing of Step 7 be performed. The null hypothesis is that there is no treatment difference in total duration for SRT eligible during the 24-Week treatment period in the FAS and patients who were SRT eligible at baseline. The hypothesis will be tested at the two-sided significance level of 0.05.	

[1] A final determination of the order of fifth and sixth secondary endpoints will be made once the trial is fully enrolled. The total duration of SRT eligibility over the 24-week of treatment in patients who were SRT eligible at baseline might be brought up to the 5th secondary endpoint if the proportion of patients with baseline NYHA III is determined to be approximately 30% or greater.

7.7. Analysis of the Primary Efficacy Endpoint

7.7.1. Primary Efficacy Analysis

The primary endpoint is change in pVO_2 from baseline to Week 24. The **primary analysis** will be performed using an ANCOVA model that includes terms of treatment, randomization stratification factors (beta-blocker use status and CPET modality), baseline pVO_2 and baseline body weight as covariates in the FAS. Table 5 below displays details of the two estimands for the primary endpoint.

Table 5: Estimands for Primary Endpoint

Attributes	Primary Estimand	Secondary Estimand
Population	FAS, target population of potentially treatable aficamten subjects. Subjects without any dose of IP excluded.	Hypothetical target population of potentially treatable aficamten subjects continue with treatment and are capable of completing the Week 24 assessment. Subjects with missing Week 24 pVO_2 due to intercurrent events or discontinuing treatment prior to Week 24 will be excluded.
Variable	Change from baseline to Week 24 in pVO_2. Data to be analyzed include all observed Week 24 pVO_2 values from subjects who complete 24 weeks of treatment, or from subjects who early terminate from the treatment but remain in the study and have Week 24 pVO_2 and imputed pVO_2 for subjects who don't have Week 24 pVO_2. Imputation details are provided below.	Change from baseline to Week 24 in pVO_2. Data to be analyzed include observed pVO_2 values from subjects who complete at least 24 weeks of treatment.
Measure of intervention effect	Mean treatment difference regardless of completing 24 weeks of treatment and experiencing intercurrent events.	Mean treatment difference among all subjects who remained on their randomized treatment for 24 weeks.

Subjects will be followed per the schedule of assessments from randomization through their final visit irrespective of whether the subject is continuing to receive study treatment. Reasons for not completing Week 24 CPET will be recorded on eCRF; categories of reasons include adverse events, early termination, equipment failure, investigator decision, subject decision and other. The percentage of missing CPET data at Week 24 and the reasons for the missing data will be tabulated in the FAS. The following type of intercurrent events could preclude CPET at Week 24.

- Death
- Hospitalization
- CV AEs
- non-CV AEs (e.g., orthopedic injury)

- COVID-19 related intercurrent events e.g., subjects' decision to early terminate from the study due to the COVID-19 precautions, site closures, hospitalization due to COVID-19, or COVID-19 symptoms preventing subjects from coming to the Week 24 visit.

CPET data deemed to be invalid by the CPET core lab will be treated as missing CPET data in the analysis. CPET core lab flags the CPET results as invalid when there are:

- Onset of a non-cardiac issue with the subject that precluded conduct of the exercise study (as defined by inability to turn the pedals or walk on a treadmill during the warm-up period for at least 3 min)
- Technical - technical equipment failure during CPET (i.e., air leak/lack of proper equipment utilization with missing nose clip or malfunction of the ergometer or metabolic cart leading to inability to adequately capture gas exchange data)
- CPET MOP (Manual of Operating Procedures) - major CPET process deviation, particularly when deviations result in significant differences in the way tests were conducted at baseline and end-of study evaluations.

Missing data as a result of an invalid assessment by the CPET core lab cab will be considered as missing at random.

Missing pVO2 at Week 24 regardless of type of intercurrent events will be imputed using multiple imputation methodology under the MAR assumption for the primary analysis of the primary estimand. Missing Week 24 CPET is expected to be low. Patients will be followed according to the schedule of activities in the protocol from randomization through the date of final visit irrespective of whether the patient is continuing to receive IP unless the patient has discontinued prematurely from the study or withdrawn consent. The protocol allows up to 4 weeks extension of Week 24 in the event that the subject is temporarily unable to exercise due to an AE e.g., ankle sprain, upper respiratory infection etc. or due to equipment malfunction to ensure post randomization CPET data collection. Missing Week 24 CPET due to intercurrent events i.e., non-CV AEs or COVID-19 related intercurrent events can be considered as MAR. Death event is expected to be rare given the patient population and the duration of the treatment in this study. Assume patients' risk of death event or hospitalization due to HCM symptom and other CV AEs is proportional between treatment groups, missing data due to this type of intercurrent events for the primary estimand will be imputed as MAR.

The imputation model will use regression multiple imputation which includes treatment group, randomization stratification factors, baseline pVO_2, sex, age, baseline hemoglobin, baseline body weight, baseline KCCQ CSS, and baseline NYHA class and the last available post randomization NYHA functional class, resting and Valsalva LVOT. Categorical variables, i.e., treatment group, baseline NYHA functional class, and sex will be specified in the CLASS statement. Fifty (50) imputed datasets will be generated. Change from baseline in pVO_2 will be calculated based on the observed and imputed data. Each of the imputed dataset will be analyzed using the primary analysis ANCOVA model. LSM estimate of treatment difference and the standard error will be combined using Rubin's rules (Rubin 1987) to produce a LSM estimate of the treatment difference, its 95% confidence interval, and p-value for the test of null hypothesis of no treatment effect. LSM, LSM difference and the corresponding standard error, 95% CI and p-value will be presented.

7.7.2. Sensitivity Analyses of the Primary Efficacy Endpoint

To evaluate the robustness of the primary analysis approach, sensitivity analyses, e.g., placebo-based imputation and tipping point analysis will be performed. In placebo-based imputation, missing pVO2 from subjects who discontinued from aficamten treatment or missing pVO2 from subjects from the placebo arm will be imputed based on the model that is constructed using observed pVO2 data from the placebo arm. Missing pVO2 from subjects who remained on aficamten treatment will be imputed based on the model that is constructed using observed pVO2 data from aficamten arm. Tipping point analysis will be performed by applying a range of negative shift to adjust the imputed value of missing pVO2 in aficamten group. If there are 10% or more missing data and/or 5% or more subjects missing data due to reason related to IP, the primary analysis will have the missing data imputed using placebo-based imputation.

The tipping point can be identified while the result is no longer statistically significant. Clinical judgment will be applied to evaluate the plausibility of the assumptions underlying this tipping point.

In addition, sensitivity analysis to evaluate COVID-19 impact will be performed by repeating the primary analysis after setting the Week 24 CPET to missing from subjects who are impacted by COVID pandemic. If there are more than 20% of patients from a region that are impacted by COVID pandemic another sensitivity analysis will be performed by repeating the primary analysis after removing the patients from the impacted region. Subjects included in this analysis is the Full Analysis set excluding all the randomized subjects from that region. This sensitivity analysis will also be performed for the secondary endpoints.

Another sensitivity analysis is to fit a repeated measures mixed model to pVO_2 baseline and Week 24 data. The model includes stratification factors, visit, stratification by visit, and a numeric covariate which equals 0 for both treatment groups at baseline and equals 0 for placebo at Week 24 and equals 1 for aficamten group at Week 24. The primary treatment comparison is for the numeric covariate for treatments which corresponds to the treatment difference at week 24 in a specification for which there is no treatment difference at baseline.

7.7.3. Subgroup Analyses for the Primary Endpoint

Subgroup analyses will be performed by including the subgroup effect and subgroup by treatment interaction terms to the primary ANCOVA model for the primary endpoint. For subgroup analysis for the primary estimand, missing data imputed in the primary analysis will be used in the subgroup analyses. Only summary statistics will be presented for the subgroup level when the number of subjects in either treatment arm is ≤ 15 at this level. LSM estimate of the treatment difference, 95% confidence intervals for the mean treatment difference and nominal p-values will be provided for each subgroup level.

7.7.4. Supportive Analyses for the Primary Endpoint

To explain heterogeneity or identify treatment effect modifiers from the baseline characteristics, covariates used to define pre-specified subgroups and these covariates by treatment interaction terms will be included in the ANCOVA model as supportive analysis. Covariates measured as continuous will be introduced to the model as continuous variable. Global test of covariates by treatment interactions will be performed.

Stepwise model selection method will be used based on the default stay or entry level of 0.05 to evaluate significant baseline covariates impact on the primary endpoint. ANCOVA model will be repeated by adjusting for the significant baseline covariates. These analyses will be based on observed data.

The assumptions of the ANCOVA model will be investigated graphically. The scaled residuals will be examined. The analysis will be repeated after transforming pVO_2 data into ranks if severe deviation from the normal assumption is observed. The primary analysis will report the rank-based p-value and provide the treatment effect using the estimate from the primary analysis based on the original scale. The decision to use report rank based p value for the primary analysis will be specified and document prior to the database lock. Ranks will be applied to all changes from baseline data after the imputation step. Baseline data will be ranked separately.

The secondary estimand will be analyzed using the same primary analysis ANCOVA model for the primary estimand. Subgroup analysis for the secondary estimand will be performed using the same ANCOVA model for the subgroup analysis for the primary estimand.

7.8. Analysis of the Secondary Efficacy Endpoints

The secondary endpoint(s) of the trial are:

- Change in KCCQ-CSS from baseline to Week 12 and Week 24
- Proportion of patients with ≥1 class improvement in NYHA Functional Class from baseline to Week 12 and Week 24
- Change in post-Valsalva LVOT-G from baseline to Week 12 and Week 24
- Proportion of patients with post-Valsalva LVOT-G <30 mmHg at Weeks 12 and 24
- Change in total workload during CPET from baseline to Week 24
- Total duration of SRT eligibility during the 24-Week treatment period in patients who were SRT eligible at baseline

7.8.1. Analysis of the Secondary Efficacy Endpoints

1. Change in KCCQ-CSS from baseline to Week 12 and Week 24

The primary analysis for change in KCCQ-CSS from baseline to Week 12 and 24 will be performed using a MMRM model with baseline as covariate, randomization stratification factors, visit, treatment group, and interaction terms of treatment by visit and baseline by visit. An unstructured covariance matrix will be specified. All data observed up to Week 24 post randomization will be included in the model. Estimates for endpoints at Week 12 and Week 24 will be obtained from the LS Means estimate at visit of Week 12 and 24 from the model.

If there are >10% difference in baseline KCCQ CSS across regions, treatment by visit and region, baseline by visit and region will be included in the model. Treatment effect at each visit week will be estimated from the interaction terms of treatment by visit by region, with coefficients for each region determined by proportion of patients evaluated in each region.

Sensitivity analysis based on multiple imputation with fifty invocations will be performed. First the intermittent missing data will be imputed using the Markov Chain Monte Carlo (MCMC)

method under MAR assumption. The imputation will be performed separately for each randomized treatment group and will include the following terms in the imputation model: endpoint observations from baseline up to Week 24. The monotone missing data will be imputed using the imputation model built from placebo group. 50 complete data sets will be generated and analyzed using an ANCOVA model with baseline as covariate, randomization stratification factors and treatment as fixed effects for Week 12 and 24, separately. The results from the 50 complete data sets will be combined using Rubin's combination rule for the inference.

Subgroup analysis will be performed using the primary analysis model MMRM model including additional effects of subgroup, subgroup by treatment and subgroup by treatment by visit interaction the model.

COVID-19 related sensitivity will be performed to first censor the data point(s) subsequent to being impacted by COVID pandemic to missing and impute the missing data using the same method specified above.

2. Change from baseline in post-Valsalva LVOT-G from baseline to Week 12 and Week 24

The analysis of this endpoint will follow the same specified above for change in KCCQ-CSS change from baseline to Week 12 and 24.

3. Proportion of patients with ≥1 class improvement in NYHA Functional Class at Weeks 12 and Week 24

For the proportion of patients with ≥1 class improvement in NYHA class at Week 12, the later available Week 8 and 16 NYHA class will be used if Week 12 NYHA class is not available. Patient will be considered as not achieving ≥1 improvement in NYHA class at Week 12 if no NYHA at Weeks 8, 12 and 16 is available. Similarly, Week 20 NYHA class will be used if Week 24 NYHA is not available. Patient will be considered as not achieving ≥1 improvement in NYHA class at Week 24 if Week 20 and Week 24 NYHA are not available. Proportion of patients with ≥1 class improvement in NYHA class will be analyzed using Cochran– Mantel–Haenszel (CMH) test stratified by randomization factors. The p-value and 95% CI will be obtained using exact method.

Subgroup analysis will be done by repeating the CMH test for each subgroup level without specifying stratifying by randomization stratification factors.

4. COVID-19 related sensitivity analysis will be performed by first censoring the data point(s) subsequent to being impacted by COVID pandemic to missing. Missing NYHA class will be imputed using multiple imputation Fully Conditional Specification (FCS) logistic regression method. The complete data will be analyzed using repeated logistic regression model with baseline as covariate, randomization of beta-blocker use status, visit, treatment group, interaction terms of treatment group by visit as fixed effect. An unstructured covariance matrix will be specified. Compound symmetry covariance matrix will be specified if there are computational issue. Proportion of patients with post-Valsalva LVOT-G <30 mmHg at Weeks 12 and 24

For the proportion of patients with post-Valsalva LVOT < 30 mmHg at Week 12, Week 16 post-Valsalva LVOT will be used if Week 12 visit is performed but post Valsalva LVOT is not available. Week 8 post-Valsalva LVOT will be used if Week 16 is not available. Similarly,

Week 20 post Valsalva LVOT will be used if Week 24 is performed but post Valsalva LVOT is not available.

Proportion of patients with post-Valsalva LVOT-G <30 mmHg at Weeks 12 and 24 will be analyzed using Cochran– Mantel–Haenszel (CMH) test stratified by randomization factors. The p-value and 95% CI will be obtained using exact method.

Subgroup analysis will be done by repeating the CMH test for each subgroup level without specifying stratifying by randomization stratification factors.

COVID-19 related sensitivity analysis will be performed by first censoring the data point(s) subsequent to being impacted by COVID pandemic to missing. Missing LVOT-G will be imputed the same way as specified in the sensitivity analysis of change in LVOT-G from baseline to Week 12 and 24. Responders will be defined based on the complete data and analyzed using a repeated logistic regression model with baseline as covariate, randomization of beta-blocker use status, visit, treatment group, interaction terms of treatment group by visit as fixed effect. An unstructured covariance matrix will be specified. Compound symmetry covariance matrix will be specified if there are computational issue.

5. Change in total workload during CPET from baseline to Week 24

The primary and sensitivity analysis for the total workload will follow the same analysis approach for the primary estimand of the primary endpoint.

Subgroup analysis will be performed similarly as specified for the primary endpoint.

COVID-19 related sensitivity for total workload from baseline to Week 24 follows the same approach specified for the primary endpoint.

6. Total duration of SRT eligibility during the 24-Week treatment period in patients who were SRT eligible at baseline

Patient SRT eligibility will be assigned after data handling in case there are missing NYHA class or LOVT assessments. Patient will be treated as SRT eligible if SRT eligibility can’t be determined due to the missing NYHA class or LVOT assessments or clinical visits not performed after patient early terminates from the study.

Total duration will be calculated as the number of days patients are SRT eligible, from the date of the randomization until Week 24. Total duration will be 24 weeks if a patient remains SRT eligible at all visits from until Week 24 (regardless of whether a patient is SRT eligible at Week 24 or not). Because the SRT eligibility status of a patient may change during the study conduct, only intervals during which the patient is SRT eligible, will be summed. The start of the interval is the time of randomization, and the end of the interval is the date prior to the visit when the patient becomes SRT not eligible.

SRT (Y/N) / Case #	Week 2	Week 4	Week 6	Week 8	Week 12	Week 16	Week 20	Week 24	Duration SRT Eligible (Weeks)
1	N	Y	Y	Y	N	N	Y	Y	12
2	Y	Y	N	N	Y	N	N	Y	10
3	Y	Y	N	N	N	N	N	N	16
4	Y	Y	N	N	Y	N	Y	N	14
5	Y	Y	N	N	Y	N	Y	Y	14

Total duration of SRT eligibility during the 24 Week of treatment period will be analyzed using an ANCOVA model includes treatment and randomization stratification factor beta blocker use/non-use as fixed effects and significant baseline characteristics as covariates. Stepwise model selection method will be used based on the default stay or entry level of 0.05 to evaluate significant baseline covariates. Sensitivity analysis of the SRT eligible endpoints includes repeating the same ANCOV analysis the area under the curve calculated using the numeric value assigned to the SRT eligibility at each visit and treatment duration until Week 24, where SRT eligibility is assigned to value of 1 and SRT not eligible is assigned to value of 0.

Subgroup analysis for the responder type of endpoints will be done by repeating the CMH test for each subgroup level without specifying stratifying by randomization stratification factors.

COVID-19 related sensitivity analysis will be performed by first censoring the data point(s) subsequent to being impacted by COVID pandemic to missing. Missing NYHA class will be imputed using multiple imputation Fully Conditional Specification (FCS) logistic regression method. Sensitivity analysis for total duration of SRT eligible will be performed after the SRT eligibility being re-derived based on the imputed NYHA class and LVOT-G values. Table 6 summarizes the primary and secondary efficacy endpoints and planned analysis method.

Table 6: Endpoint Summary Table

Endpoint	Primary Analysis Method	Sensitivity/Subgroup Analysis	COVID-19 Related Analyses[1]
Primary Endpoint: Change in pVO2 on CPET from baseline to Week 24			
Primary estimand[2]	Missing data will be imputed using multiple imputation method (Section 7.7.1). Complete dataset will be analyzed using an ANCOVA model with fixed effects of treatment, randomization stratification factors baseline pVO_2 value and baseline body weight	Sensitivity analysis<br>• ANCOVA model will be repeated with missing data from subjects who discontinued aficamten treatment as if the aficamten subjects were in the placebo arm<br>• Tipping point analysis<br>Supportive analysis<br>• Mixed model with numeric covariate as 0 for baseline and placebo group at Week 24 and 1 for aficamten group at Week 24, visit, stratification factors and stratification factors by visit as fixed term. unscheduled covariance structure will be specified.<br>• Multivariate ANCOVA model to evaluate treatment by covariates interaction; ANCOVA model with significant covariates per model selection<br>Subgroup analyses for variables in Section 7.5	• Repeat the primary analysis after setting Week 24 pVO_2 to missing from subjects who were impacted by COVID pandemic<br>• Summarize the number of subjects infected (positive COVID-19 test with or without symptoms) prior to Week 24 CPET

Table 6: Endpoint Summary Table (Continued)

Endpoint	Primary Analysis Method	Sensitivity/Subgroup Analysis	COVID-19 Related Analyses[3]
Secondary estimand	ANCOVA model with fixed effects of treatment, randomization stratification factors baseline pVO_2 value and baseline body weight.	Subgroup analyses for variables in Section 7.5	• Repeat the primary analysis by excluding subjects whose Week 24 pVO_2 are obtained from subjects who were impacted by COVID pandemic.
Secondary Endpoints			
Change in KCCQ-CSS from baseline to Week 12 and Week 24	MMRM model with baseline as covariate, randomization stratification factors, visit, treatment group, and interaction terms of treatment by visit and baseline by visit. An unstructured covariance matrix will be specified.	Sensitivity analysis:<br>• Intermittent missing data be imputed using multiple imputation MCMC first and the monotone missing values will be imputed using the imputation model built from the placebo group. Complete data will be analyzed using ANCOVA model for each week separately.<br>Subgroup analyses for variables in Section 7.5	• Repeat the primary analysis (MMRM) model after setting datapoint subsequent to being impacted by COVID pandemic missing.
Change in post-Valsalva LVOT-G from baseline to Week 12 and Week 24	MMRM model with baseline as covariate, randomization stratification factors, visit, treatment group, and interaction terms of treatment by visit and baseline by visit. An unstructured covariance matrix will be specified.	Sensitivity analysis:<br>• Intermittent missing data be imputed using multiple imputation MCMC first and the monotone missing values will be imputed using the imputation model built from the placebo group. Complete data will be analyzed using ANCOVA model for each week separately,<br>Subgroup analyses for variables in Section 7.5	• Repeat the primary analysis (MMRM) model after setting datapoint subsequent to being impacted by COVID pandemic to missing.

Table 6: Endpoint Summary Table (Continued)

Endpoint	Primary Analysis Method	Sensitivity/Subgroup Analysis	COVID-19 Related Analyses[4]
Proportion of patients with ≥1 class improvement in NYHA Functional Class from baseline to Week 12 and Week 24	CMH test stratified by randomization factors	Subgroup analyses for variables in Section 7.5	Re-derive responder after setting datapoint subsequent to being impacted by COVID pandemic to to missing. Missing NYHA will be imputed using multiple imputation using Fully Conditional Specification (FCS) logistic regression method.
Proportion of patients with post-Valsalva LVOT-G <30 mmHg	CMH test stratified by randomization factors	Subgroup analyses for variables in Section 7.5	Re-derive responder after setting datapoint subsequent to being impacted by COVID pandemic to missing.
Change in total workload during CPET from baseline to Week 24	Missing data will be imputed using multiple imputation method (Section 7.7.1). Complete dataset will be analyzed using an ANCOVA model with fixed effects of treatment, randomization stratification factors baseline total workload value and baseline body weight.	Sensitivity analysis<br>• ANCOVA model will be repeated with missing data from subjects who discontinued aficamten treatment as if the aficamten subjects were in the placebo arm.<br>• Tipping point analysis<br>Subgroup analyses for variables in Section 7.5	Repeat the primary analysis after censoring Week 24 pVO_2 to missing from subjects who were impacted by COVID pandemic

Table 6: Endpoint Summary Table (Continued)

Endpoint	Primary Analysis Method	Sensitivity/Subgroup Analysis	COVID-19 Related Analyses[5]
Total duration of SRT eligible during the 24-week of treatment period	Total duration of SRT eligible during the 24-week of treatment period will be analyzed using an ANCOVA model includes treatment and randomization stratification factor beta blocker use/non-use as fixed effects and significant baseline characteristics as covariates. Stepwise model selection method will be used based on the default stay or entry level of 0.05 to evaluate significant baseline covariates.	SRT eligible will be assigned value of 1 and SRT not eligible will be assigned value of 0. Area under the curve using the numeric value assigned and treatment duration until Week 24 will be calculated. AUC will be analyzed using the same ANCOVA model.<br>Same ANCOVA model will be used to analyze the total time for SRT eligiblility.	Re-derive SRT eligibility after setting datapoint subsequent to being impacted by COVID pandemic to missing.

[1] Another sensitivity analyses will be performed for the primary and secondary endpoints by repeating the primary analysis method for the endpoints after removing the randomized patients from the region where more than 20% of patients are impacted by COVID pandemic in that region.
[2] See Table 5 for details on the two estimands for the primary endpoint.

7.9. Analysis of the Exploratory Efficacy Endpoints

Exploratory endpoints are specified in Section 2.2.3. Other echocardiography parameters and CPET parameters not listed as secondary endpoints or in Section 2.2.3 may also be analyzed as exploratory endpoints. All 10 summary scores will be derived for KCCQ. Change from baseline in each summary scores (except CSS) to Weeks 12 and 24 will be analyzed as exploratory endpoints. Patients with >5, 10 and 20 points improvement in KCCQ summary scores at Weeks 12 and 24 will also be summarized and analyzed. Domain scores and summary score of SAQ-7 will be derived. Health state for EQ-5D-5L and index value using US value set will be calculated. Analysis of change from baseline in index score and VAS score of EQ-5D-5L be performed. Proportional change in Valsalva and resting LVOT-G will be derived and analyzed as exploratory endpoints.

7.9.1. Analysis of the Exploratory Efficacy Endpoint

The same ANCOVA model specified for the primary endpoint will be used to analyze the endpoints from CPET and continuous CMR measurements that are only assessed once post randomization. Body weight will not be included in the model to analyze CMR parameters. The same MMRM model will be used to analyze the continuous endpoints measured at multiple visits post randomization. CMH test stratified by randomization factors will be used analyze endpoints evaluation proportion of responders or binary outcomes.

Proportion of responders will at each week will be summarized. Responders that are defined based on NYHA classification or LVOTs will be assigned after data handling in case there are missing NYHA class or LOVT assessments. Missing NYHA classification or LVOT value will be imputed the same way as that for the secondary endpoints that are based on NYHA classification or LVOT value. Patient will be treated as non-responder if patient's response status can't be determined due to the missing NYHA classification or LVOT assessments (after the imputation above) or clinical visits not performed after the patient early terminates from the study. Proportion of responders will be analyzed using Cochran– Mantel–Haenszel (CMH) test stratified by randomization factors. The p-value and 95% CI will be obtained using exact method. Proportion of patients remaining SRT eligible at Week 24 will be analyzed using logistic model regression model stratified by beta blocker use/non-use adjusting for significant baseline characteristics. The model will include treatment as fixed effect and baseline characteristics as covariates. Covariates measured as continuous will be introduced to the model as continuous variable. Stepwise model selection method will be used based on the default stay or entry level of 0.05 to evaluate significant baseline covariates. Supportive analysis using CMH stratified by randomization factor (beta-blocker use/non-use) will be performed. Another supportive analysis of proportion of patients SRT eligible at each visit will be provided using a repeated measure logistic regression model with treatment, visit, treatment by visit and significant baseline characteristics from the model above. Time to first SRT eligible in patients who are SRT eligible at baseline will be analyzed using Kaplan-Meier method.

For NT-proBNP and hs-cardiac-TnI, the log transformed proportional change will be analyzed using a MMRM model with log baseline as covariate, treatment group, randomization stratification factors, visit, log baseline by visit and treatment by visit interaction as fixed effects. Log transformed proportional change in Valsalva and resting LVOT will be analyzed using the

same model. Geometric LS Means estimate and ratio of proportional change in NT-proBNP and hs-cardiac-TnI between aficamten vs. placebo, 95% CI of ratio and p-value will be presented. Median and median difference of NT-pro-BNP and hs-cardiac- Tnl between treatment group and 95% confidence of the median difference will be presented at Week 12 and 24. Time to 1 mm ST depression at Week 24 will be analyzed using Cox regression model with treatment, randomization stratification factors as fixed effect. Patients didn’t experience 1mm ST depression will be censored at the end of CPET exercise. Logistic regression model will be fit to LVH strain pattern on ECG for Week 12 and 24, separately. The model will include baseline LVH pattern, stratification factors and treatment. Difference in proportion of patients with no LVH will be estimated and 95% CI for odds ratio (aficamten vs. placebo) and its corresponding p value will be obtained.

8. SAFETY AND TOLERABILITY

Safety and tolerability analyses will be based on the Safety Analysis Set. Safety data will be analyzed descriptively and tabulated by treatment groups.

8.1. Overall Summary of Tolerability

Overall summary of tolerability will include the following:

- Number of patients treated
- Number of patients with TEAEs
- Number of patients with treatment-emergent serious adverse events (TESAEs)
- Number of patients with TEAEs leading to premature treatment discontinuation
- Number of patients with at least one TEAE related to the study drug
- Patients with at least one moderate or severe TEAE
- Patients with at least one severe TEAE
- Number of Deaths

Summary of number and percent of patients with each AE category will be provided by treatment group and dose level at AE onset. Summaries of the number of events will also be provided.

8.2. Adverse Event Preferred Term and Body/Organ System Summary Tables

8.2.1. Summaries of Adverse Event Incidence Rates for All Subjects

All AE terms will be coded using the MedDRA. TEAEs and TESAEs will be summarized by primary SOC and PT, and also by severity (mild, moderate and severe) and relationship to study drug (related and not related). For a TEAE reported more than once from a patient, the TEAE will be counted only once in the SOC or PT category using the most severe occurrence or closer relationship to the study drug. All AEs will be listed.

The following subsets of TEAEs will be summarized by SOC and PT:

- All TEAEs
- TEAEs related to study drug
- TEAEs leading to early discontinuation of study drug
- TESAEs

A summary of all TEAEs by PT will be provided. AE summaries will be sorted by descending order of SOC in aficamtem group and descending order of preferred term within the SOC. TEAE and TESAE summary of ≥5% and ≥2%, respectively will be provided based on incidence rate in either aficamtem or placebo group. Summary of TEAEs by maximum severity will display number and percentage of AEs with maximum severity being mild, moderate, or severe within

each SOC and PT. All TEAE summary will also be provided by dose level. summary of number of events will be provided for all TEAE summary.

8.2.2. Summaries of Adverse Events of Special Interest

The following events are considered adverse events of special interest:

- Incidence of reported major adverse cardiac events (CV death, cardiac arrest, non-fatal stroke, non-fatal myocardial infarction, CV hospitalization)
- Incidence of new onset persistent atrial fibrillation
- Incidence of ventricular arrhythmias requiring treatment

Summary of patients counts and percentage by each event type will be provided by treatment group.

In addition, incidence of appropriate ICD discharges will be summarized for baseline and also post randomization. Incidence of aborted sudden cardiac death will be provided. Number of patients and number of incidences of LVEF <40% and 50% will be provided by treatment group. Number of patients and number of incidences of LVEF <50% and with signs and symptoms of heart failure (concomitant adverse event of heart failure or dyspnea) or experienced increase in NT-proBNP from baseline will be provided by treatment group. Signs and symptoms and NT-proBNP increase referring to AEs with onset date or NT-proBNP assessment date within ± 7 days relative to the date when LVEF <50%.

8.3. Total Duration of Therapy, Final Daily Dose of Study Medication, and Compliance

8.3.1. Summary of IP Exposure and Overall Compliance

Total duration of treatment and total exposure of study drug will be summarized. IP compliance will be derived as:

> IP compliance =100% * (number of tablets dispensed – number of tablets returned)/expected number of tablets administered.

Number of tablets dispensed and returned will be collected on the study drug accountability eCRF. For the IP kits not returned, the number of tablets returned will be set to 0 in this derivation, assuming all tablets were taken. Expected number of tablets administered will be derived as the number of daily tablets times the days in an IP dosing period, summed over all dosing periods. Days of dosing interruption will be excluded from the expected number of tablets calculation.

8.3.2. Summary of Dose Titration

IWRS-guided dose titration will be summarized showing the number and percentage of patients at each dose level by visit. Number and percentage of patients by last titrated dose will be provided.

8.4. Concomitant and Other Medications

Concomitant medications reported on the eCRF will be summarized. Medications with a start date that is 28 days after the last dose of the study drug will be excluded from the summary. The WHO Drug Dictionary will be used to classify medications by therapeutic class (ATC Class 3) and preferred name. If ATC Class 3 is not available, ATC Class 2 will be used in the summary. Coding will be performed using WHO Drug Dictionary.

8.5. Routine Laboratory Data

Clinical chemistry, hematology and urinalysis laboratory measurements and value changes from baseline at each laboratory blood sample collection time point will be summarized. Values below or above the quantifiable limits will be treated as equal to the limits in the summary. The count and percentage of patients who had normal or missing laboratory values at baseline and abnormal laboratory values post baseline will be presented. The lower limit of normal (LLN) and upper limit of normal (ULN) provided by the laboratories will be used as the criteria to determine abnormality. For each parameter, the denominator of the percentage will include patients with normal or missing assessments at baseline, and with at least one assessment post baseline. The numerator of the percentage will include patients who had at least one abnormal assessment post baseline among the patients that were counted in the denominator. Assessment collected at unscheduled visits, or the Follow-up Visit will be included in the summary.

Shift of clinical laboratory results from baseline severity to the maximum post baseline severity will be presented for selected laboratory parameters.

Liver function test results will be summarized as count and percentage of patients with normal baseline and abnormal post-baseline values in Alanine Aminotransferase (ALT), Aspartate Aminotransferase (AST), Alkaline Phosphatase (ALP) and bilirubin, with the following categories:

- ALT > 3xULN, > 5xULN, > 8xULN
- AST > 3xULN, > 5xULN, > 8xULN
- ALT and/or AST > 3xULN, > 5xULN, > 8xULN
- ALT and/or AST > 3xULN and total bilirubin > 2xULN and ALP < 2xULN
- ALT and/or AST > 3xULN and total bilirubin > 2xULN
- Bilirubin (total, indirect or direct) > 2xULN, >3xULN
- ALT or AST > 3 x ULN with symptoms including nausea, vomiting, anorexia, abdominal pain, fatigue, rash, dark-colored urine, light-colored bowel movements, jaundice, or fever

8.6. Vital Signs

Vital signs and changes from baseline will be summarized descriptively by treatment group over time. The changes from baseline at each post-baseline on-treatment visit will be additionally summarized by dose level at visit.

Patients will also be categorized into the following groups for each of the vital sign parameters if a post baseline value falls into a specific group. Unscheduled assessments will be included in the determination. The number of subjects in each group will be summarized for each dosing group.

Diastolic Blood Pressure

- ≤ 50 mmHg
- ≥ 100 mmHg

Systolic Blood Pressure

- ≤ 80 mmHg
- ≥ 160 mmHg

Heart Rate

- ≤ 50 beats/min
- ≥ 120 beats/min

Respiratory Rate

- > 18 breaths/min

8.7. Electrocardiogram

The baseline ECG is defined as the mean of all pre-dose assessments. PR, RR, QRS, QT, and Fridericia corrected QT (QTcF) intervals and their change from baseline will be summarized by treatment group and scheduled assessment. patients will be categorized into the following groups per their maximum change from baseline in QTcF. Unscheduled assessments will be included in the determination of the maximum change. The number and percentage of subjects in each group will be summarized.

- ≤30 msec
- >30 – 60 msec
- >60 msec

Patients will also be categorized into the following groups per their maximum post baseline QTcF. Unscheduled assessments will be included in the determination of the maximum post baseline value. The number of subjects in each group will be summarized for each dosing group.

- ≤450 msec
- >450 – 480 msec
- >480 – 500 msec
- >500 msec

ECG morphology analyses will be performed. New onset findings will be presented as the percentage of subjects with ‘new’ finding (ECG finding that were not present at any baseline ECG and became present on at least 1 ECG during the treatment) for the following variables

2:1 AV Block, AV Mobitz I,II, Complete heart block, first degree AV block, left atrial abnormality, left ventricular hypertrophy, right ventricular hypertrophy, incomplete left bundle branch block, incomplete right bundle branch block, intraventricular conduction defect, left anterior hemiblock, left bundle branch block, left posterior hemiblock, right bundle branch block, Wolff-Parkinson-White, Artificial pacemaker, atrial pacing, sinus bradycardia, sinus pauses, sinus tachycardia, atrial fibrillation, atrial flutter, atrial tachycardia, supraventricular tachycardia, prolonged QTC, ST depressed, ST elevated, T wave inverted, non-sustained ventricular tachycardia, ventricular fibrillation.

9. PHARMACOKINETICS

Plasma concentrations of CK-3773274 and its metabolites and PK parameters $C_{post\ dose}$ and $C_{pre\text{-}dose}$ will be summarized using descriptive statistics including arithmetic mean, standard deviation, coefficient of variation, geometric mean, geometric coefficient of variation, median, and range. Geometric mean concentrations over time will be graphically displayed.

10. REFERENCES

EuroQol Group. (1990). "Eq-5d-3l." EuroQol Research Foundation, from https://euroqol.org/publications/user-guides, Updated Date Accessed Date.

EuroQol Group. (2009). "Eq-5d-5l." EuroQol Research Foundation, from https://euroqol.org/publications/user-guides, Updated Date Accessed Date.

Pickard, A. S., Law, E. H., Jiang, R., Pullenayegum, E., Shaw, J. W., Xie, F., et al. (2019). "United states valuation of eq-5d-5l health states using an international protocol." Value in Health 22(8): 931-941.

Rubin, D. B. (1987). Multiple imputation for nonresponse in surveys. New York, John Wiley & Sons, Inc.

11. APPENDIX

11.1. Patient-reported Outcome Scoring Algorithm

11.1.1. KCCQ

There are 10 summary scores within the KCCQ, which are calculated as follows:

1. Physical Limitation

- Code responses to each of Questions 1a-f as follows:

  Extremely limited = 1
  Quite a bit limited = 2
  Moderately limited = 3
  Slightly limited = 4
  Not at all limited = 5
  Limited for other reasons or did not do = <missing value>

- If at least three of Questions 1a-f are not missing, then compute
  Physical Limitation Score = 100*[(mean of Questions 1a-f actually answered) - 1]/4
  (see footnote at end of this appendix for explanation of meaning of "actually answered")

2. Symptom Stability

- Code the response to Question 2 as follows:

  Much worse = 1
  Slightly worse = 2
  Not changed = 3
  Slightly better = 4
  Much better = 5
  I've had no symptoms over the last 2 weeks = 3

- If Question 2 is not missing, then compute

  Symptom Stability Score = 100*[(Question 2) – 1]/4

3. Symptom Frequency

- Code responses to Questions 3, 5, 7 and 9 as follows:

  Question 3
  Every morning = 1
  3 or more times a week but not every day = 2
  1-2 times a week = 3
  Less than once a week = 4
  Never over the past 2 weeks = 5

  Questions 5 and 7
  All of the time = 1
  Several times a day = 2
  At least once a day = 3

3 or more times a week but not every day = 4
1-2 times a week = 5
Less than once a week = 6
Never over the past 2 weeks = 7

Question 9
Every night = 1
3 or more times a week but not every day = 2
1-2 times a week = 3
Less than once a week = 4
Never over the past 2 weeks = 5

- If at least two of Questions 3, 5, 7 and 9 are not missing, then compute:

S3 = [(Question 3) – 1]/4
S5 = [(Question 5) – 1]/6
S7 = [(Question 7) – 1]/6
S9 = [(Question 9) – 1]/4
Symptom Frequency Score = 100*(mean of S3, S5, S7 and S9)

4. Symptom Burden

- Code responses to each of Questions 4, 6 and 8 as follows:

Extremely bothersome = 1
Quite a bit bothersome = 2
Moderately bothersome = 3
Slightly bothersome = 4
Not at all bothersome = 5
I've had no swelling/fatigue/shortness of breath = 5

- If at least one of Questions 4, 6 and 8 is not missing, then compute

Symptom Burden Score = 100*[(mean of Questions 4, 6 and 8 actually answered) – 1]/4

5. Total Symptom Score

= mean of the following available summary scores:
Symptom Frequency Score
Symptom Burden Score

6. Self-efficacy

- Code responses to Questions 10 and 11 as follows:

Question 10
Not at all sure = 1
Not very sure = 2
Somewhat sure = 3
Mostly sure = 4
Completely sure = 5

Question 11
Do not understand at all = 1

Do not understand very well = 2
Somewhat understand = 3
Mostly understand = 4
Completely understand = 5

- If at least one of Questions 10 and 11 is not missing, then compute

  Self-Efficacy Score = 100*[(mean of Questions 10 and 11 actually answered) – 1]/4

7. Quality of Life

- Code responses to Questions 12, 13 and 14 as follows:

<u>Question 12</u>
It has extremely limited my enjoyment of life = 1
It has limited my enjoyment of life quite a bit = 2
It has moderately limited my enjoyment of life = 3
It has slightly limited my enjoyment of life = 4
It has not limited my enjoyment of life at all = 5

<u>Question 13</u>
Not at all satisfied = 1
Mostly dissatisfied = 2
Somewhat satisfied = 3
Mostly satisfied = 4
Completely satisfied = 5

<u>Question 14</u>
I felt that way all of the time = 1
I felt that way most of the time = 2
I occasionally felt that way = 3
I rarely felt that way = 4
I never felt that way = 5

- If at least one of Questions 12, 13 and 14 is not missing, then compute

  Quality of Life Score = 100*[(mean of Questions 12, 13 and 14 actually answered) – 1]/4

8. Social Limitation

- Code responses to each of Questions 15a-d as follows:

Severely limited = 1
Limited quite a bit = 2
Moderately limited = 3
Slightly limited = 4
Did not limit at all = 5
Does not apply or did not do for other reasons = <missing value>

- If at least two of Questions 15a-d are not missing, then compute

  Social Limitation Score = 100*[(mean of Questions 15a-d actually answered) – 1]/4

9. Overall Summary Score

= mean of the following available summary scores:
Physical Limitation Score
Total Symptom Score
Quality of Life Score
Social Limitation Score

10. Clinical Summary Score

= mean of the following available summary scores:
Physical Limitation Score
Total Symptom Score

Note: references to **"means of questions actually answered"** imply the following.

- If there are n questions in a scale, and the subject must answer m to score the scale, but the subject answers only n-i, where n-i >= m, calculate the **mean of those questions** as

  (sum of the responses to those n-i questions) / (n-i)
  not

(sum of the responses to those n-i questions) / n

11.1.2. SAQ-7

Three domain scores and one summary score are generated from the SAQ-7:

Physical Limitation Score (SAQ7-PL)

Angina Frequency Score (SAQ7-AF)

Quality of Life Score (SAQ7-QL)

Summary Score (SAQ7)

Scores are scaled 0-100, where 0 denotes the lowest reportable health status and 100 the highest.

<u>Physical limitation Score</u>

The physical Limitation score corresponds to Questions 1a, 1b and 1c. Responses are coded as follows:

Extremely limited	1
Quite a bit limited	2
Moderately limited	3
Slightly limited	4
Not at all limited	5
Limited for other reasons or did not do the activity	6

A response of 6 is treated as missing value for the purpose of scoring. If responses to two or more questions are missing, no score is computed. If the response to Question 1a or Question 1c

is missing, it is assigned the responses from Question 1b. If the response to Question 1b is missing, it is assigned the average of responses to Questions 1a and 1c. The score is then calculated by taking the average of the three responses and rescaling to 0 – 100, as follows:

SAQ7-PL = 100*[(average of Questions 1a, 1b and 1c)-1]/4

<u>Angina Frequency Score</u>

The Angina Frequency score corresponds to Questions 2 and 3. Responses are coded as follows:

4 or more times per day	1
1 – 3 times per day	2
3 or more times per week but not every day	3
1 -2 times per week	4
Less than once a week	5
None over the past 4 weeks	6

If responses to both questions are missing, no score is computed. Otherwise, the score is calculated by taking the average of non-missing responses and rescale to 0-100 as follows:

SAQ7-AF = 100 *[(average of Questions 2 and 3) -1]/5

<u>Quality of Life Score</u>

The quality of life score corresponds to Questions 4 and 5. Responses are coded as follows:

Question 4	
It has extremely limited my enjoyment of life	1
It has limited my enjoyment of life quite a bit	2
It has moderately limited my enjoyment of life	3
It has slightly limited my enjoyment of life	4
It has not limited my enjoyment of life at all	5
Question 5	
Not satisfied at all	1
Mostly dissatisfied	2
Somewhat satisfied	3
Mostly satisfied	4
Completely satisfied	5

If responses to both questions are missing, no score is computed. Otherwise, the score is calculated by taking the average of the non-missing response and rescaling to 0 -100, as follows:

SAQ7-QL= 100*[(average of Question 4 and 5)-1]/4

<u>Summary Score</u>

The summary score represents an integration of the patients' physical limitation, angina symptom and quality of life. If all three domain scores are missing, no summary score is computed. Otherwise, the score is calculated as the average of the non-missing domain scores:

SAQ7 = average of SAQ- PL, SAQ- AF, and SAQ – QL

11.1.3. EQ-5D-5L

Five dimensions of the EQ-5D-5L include 'mobility', 'selfcare', 'activity', 'pain', and 'anxiety'. The US Pickard value set will be used to compute the EQ-5D-5L index values. The value set will be denoted as disut_mo for 'mobility', disut_sc for 'selfcare', disut_ua for 'activity', disut_pd for 'pain', and disut_ad for 'anxiety' in Table 7 below:

Table 7: EQ-5D-5L Value Set

		US value set
MOBILITY		disut_mo
I have no problems in walking about	1	0
I have slight problems in walking about	2	0.096
I have moderate problems in walking about	3	0.122
I have severe problems in walking about	4	0.237
I am unable to walk about	5	0.322
SELF-CARE		disut_sc
I have no problems washing or dressing myself	1	0
I have slight problems washing or dressing myself	2	0.089
I have moderate problems washing or dressing myself	3	0.107
I have severe problems washing or dressing myself	4	0.220
I am unable to wash or dress myself	5	0.261
USUAL ACTIVITIES		disut_ua
I have no problems doing my usual activities	1	0
I have slight problems doing my usual activities	2	0.068
I have moderate problems doing my usual activities	3	0.101
I have severe problems doing my usual activities	4	0.255
I am unable to do my usual activities	5	0.255
PAIN / DISCOMFORT		disut_pd
I have no pain or discomfort	1	0
I have slight pain or discomfort	2	0.060
I have moderate pain or discomfort	3	0.098
I have severe pain or discomfort	4	0.318
I have extreme pain or discomfort	5	0.414
ANXIETY / DEPRESSION		disut_ad
I am not anxious or depressed	1	0
I am slightly anxious or depressed	2	0.057
I am moderately anxious or depressed	3	0.123
I am severely anxious or depressed	4	0.299
I am extremely anxious or depressed	5	0.321
We would like to know how good or bad your health is TODAY	0 to 100	

disut_total=disut_mo+disut_sc+disut_ua+disut_pd+disut_ad;

The EQ-5D-5L index value (EQindex) =1-disut_total

The SAS code will be provided in Appendix Section 11.5

11.2. Table of Contents for Data Display Specifications

Table of contents for data display specifications will be provided in a separate document.

11.3. Data Display Specifications

Data display specifications will be provided in a separate document.

11.4. Analysis Windows

Measurements collected during the 24-week double-blind placebo-controlled period will be included only in the analysis windows up to Week 24. For data collected at a scheduled post randomization, the analysis visit will be the nominal visit as collected and visit window will not be applied.

For unscheduled or early discontinuation post randomization, analysis visit will be used according to Table 8 below when the scheduled visit is not available.

Table 8: Analysis Windows for Measurements

Visit	Scheduled Day	Lower Bound	Upper Bound
Screening	<1	<1	<1
Day 1	1	1	1
Week 2	15	2	21
Week 4	29	22	35
Week 6	43	36	49
Week 8	57	50	70
Week 12	85	71	98
Week 16	113	99	126
Week 20	141	127	154
Week 24	169	155	196
Week 28/Follow-up	last dose + 28 days	-	-

Note: Week 28/Follow-up should occur 4 weeks after last dose. The analysis visit of week 28/Follow-up will include the nominal visit End of Study (Week 28) and any unscheduled visits occurred after the week 24 window.

11.5. Sample SAS Codes

ANCOVA model for primary endpoint

```
proc mixed data=work;
        class <treatment arm (ref='0')> <Beta Blocker use> <Exercise Modality>;
        model chg=<base pvo2 > <base weight> <treatment arm> <beta blocker use> <Exercise
        Modality> /solution s Influence(EFFECT=usubjid) outp=out vciry;
        lsmeans <treatment arm>/pdiff cl;
        ods output solutionf=mixparms covB=mixcovb;
run;
```

This code assumes that the analysis involves 2 levels in treatment arm (e.g., placebo group is coded as 0 and aficamten is coded as1).

MMRM model

```
proc mixed data=work;
```

```
    class <Subject> <treatment arm (ref=’0’)> <Beta Blocker use> <Exercise Modality> <visit>;
model <chg> = <base> <treatment arm> <visit> <visit>*<treatment arm> <Beta Blocker use> <Exercise
Modality> <visit>*<base>/ddfm=kenwardroger;
repeated <visit> / type=un subject=<Subject>;
lsmeans <visit>*<treatment arm>/cl pdiff;
run;
```

This code assumes 2 level in treatment arm with placebo group is coded as 0. visit has level of nominal visit week where continuous measurements are assessed up to Week 24.

Imputation model for primary endpoint

```
proc mi data=work seed=&seed out=miout NIMPUTE=50;
        class <treatment arm> <Beta Blocker use> <Exercise Modality> <base NYHA> <randomization
        factors>;
        var <treatment arm> <randomization stratifications> <sex> <age> <base pVO2> <base
        hemoglobin> <base KCCQ CSS> <baseline NYHA> <last available post rand NYHA> <last
        available post rand resting LVOT> <last available post rand Valsalva LVOT> <W24 pVO2> ;
        monotone reg(W24 pVO2);
  run;
```

Placebo-based imputation for sensitivity analysis of the primary endpoint

Step 1:
generate input dataset for subjects in placebo group or subject who didn’t complete treatment, and datasets for the rest of subjects in aficamten group.
Step2: impute missing data with separately with each input dataset using the imputation model for the primary endpoint.
Step 3. Combined the complete data from the imputations above as final imputed dataset.

Tipping point imputation for primary endpoint

```
proc mi data=work seed=&seed out=miout NIMPUTE=50;
        class <treatment arm> <Beta Blocker use> <Exercise Modality> <base NYHA> <>;
        var <treatment arm> <sex> <age> <base pVO2> <base hemoglobin> <base KCCQ CSS>
        <baseline NYHA> <last available post rand NYHA> <last available post rand resting LVOT>
        <last available post rand Valsalva LVOT> <W24 pVO2> ;
        monotone reg(W24 pVO2);
        mnar adjust (W24 pVO2/shift=&shift adjustobs=(Trt='1'));
run;
```

shift will be set so that the imputed value for aficamten group is 1 ml/min/kg worse as starting point with increment of 1 ml/min/kg worse each time until the result is no longer significant.

Multiple Imputation for sensitivity analysis for repeated measures endpoint

Step1:

```
proc mi data=work1 seed=&seed1 out=miout1 NIMPUTE=50;
        by <treatment arm>;
        mcmc IMPUTE=monotone ;
        var <base> <var week 2> <var week4> <var week6> <var week8> <var week12> <var week16>
<var week20> <var week24>;

run;
```

Step 2:

```
proc mi data=miout1 seed=&seed NIMPUTE=1 OUT=miout2;
 class <treatment arm>;
 by _IMPUTATION_;
 var <treatment arm> <base> <var week 2> <var week4> <var week6> <var week8> <var week12> <var
week16> <var week20> <var week24>;
 monotone reg (<base> <var week 2> <var week4> <var week6> <var week8> <var week12> <var
week16> <var week20>);
 mnar model (<base> <var week 2> <var week4> <var week6> <var week8> <var week12> <var
week16> <var week20> / modelobs = (trt01pn = ‘0’));
run;
```

Multiple Imputation for categorical endpoint

```
proc mi data=work seed=&seed out=outwork NIMPUTE=50;
        class <var at week8> < var at week 12> < var at week 16> < var at week 20> < var at week 24>
<treatment arm>;
   var <treatment arm> <var at week8> < var at week 12> < var at week 16> < var at week 20> < var at
week 24> ;
fcs logistic (<var at week8> < var at week 12> < var at week 16> < var at week 20> < var at week 24> =
<treatment arm> /link=glogit);
run;
```

SAS codes using proc minalyze to combine results from imputed datasets

The pooled estimates from the 50 imputed datasets are obtained from the following codes.

```
proc mianalyze data=est;
  modeleffects estimate;
  stderr stderr;
run;
```

Mixed model for CPET data

```
proc mixed data=work;
    class <subject ID> <visit> <stratification factors> ;
    model <chg in CPET>=<trtid> <stratification factors> <stratification factors>*<visit> /s;
    estimate 'active at Week 24’ int 1 <trtid> 1 <stratification factors> &c1 &c2 stratification
    factors>*<visit> 0 0 &c1 & / e;
    estimate 'placebo at Week 24’ int 1 <trtid> 0 <stratification factors> &c1 &c2 stratification
    factors>*<visit> 0 0 &c1 & / e;
    estimate 'active vs PBO at Week 24' trt 1;
    repeated <visit>/subject=<subject id> type=un;
run;
```

where trtid is assigned as 0 at baseline, 0 for placebo group at Week 24 and 1 for active group at Week 24. &c1 and &c2 are the proportion of patients evaluated in each stratification level among all the patients.

Subgroup analysis for the primary endpoint

```
proc mianalyze parms=mixparms covb(effectvar=rowcol)=mixcovb;
    class <treatment arm> <beta blocker use> <exercise modality> <subgroup> ;
```

```
=<base pvo2 > <base weight> <treatment arm> <beta blocker use> <Exercise modality>
modeleffects Intercept <base pvo2 > <base weight> <treatment arm> <beta blocker use>
<Exercise Modality> <subgroup>*<treatment arm>;
run;
```

SAS codes to evaluate normality assumption for the primary endpoint

The normality assumptions for the ANCOVA analysis will be assessed by residual illustration. The outpred option in the above code stores residuals which are used to test the assumption of normality. Examination of residuals can be done using the following codes.

```
proc univariate data=work normal;
var ScaledResid; QQPLOT ScaledResid;
ods output QQPlot=qqplot;
run;
```

SAS codes to perform CMH test

```
proc freq data=work;
 tables <Beta blocker use>*<CPET modality>*<treatment arm>*<response Y/N>/CMH
 exactcmh;
run;
```

SAS codes to rank observation

```
proc rank data=work out=rwork nplus1;
Var chg;
Ranks rchg;
Run;
```

SAS code for proportional hazard Cox regression model

```
Proc phreg data=work;
        Class <treatment arm>(ref=first) ;
        Model <time>*censor (1)= ;
        Strata <beta blocker use> <CPET modality> ;
Run;
```

SAS code for stratified logistics regression model

```
Proc phreg data=work;
        Class <treatment arm>;
        Model <Response> (event='Y')= <treatment arm> <baseline covariates>/selection=stepwise
details;
        Strata <beta blocker use> ;
Run;
```

SAS code for EQ-5D-5L Index Value

```
*****************************************************************
*SAS syntax code for the computation of index*
```

values with the US TTO value set
****

```
data WORK.CAT;
set WORK.CAT;

if mobility eq 1 then disut_mo=0;
else if mobility eq 2 then disut_mo=0.096;
else if mobility eq 3 then disut_mo=0.122;
else if mobility eq 4 then disut_mo=0.237;
else if mobility eq 5 then disut_mo=0.322;

if selfcare eq 1 then disut_sc=0;
else if selfcare eq 2 then disut_sc=0.089;
else if selfcare eq 3 then disut_sc=0.107;
else if selfcare eq 4 then disut_sc=0.220;
else if selfcare eq 5 then disut_sc=0.261;

if activity eq 1 then disut_ua=0;
else if activity eq 2 then disut_ua=0.068;
else if activity eq 3 then disut_ua=0.101;
else if activity eq 4 then disut_ua=0.255;
else if activity eq 5 then disut_ua=0.255;

if pain eq 1 then disut_pd=0;
else if pain eq 2 then disut_pd=0.060;
else if pain eq 3 then disut_pd=0.098;
else if pain eq 4 then disut_pd=0.318;
else if pain eq 5 then disut_pd=0.414;

if anxiety eq 1 then disut_ad=0;
else if anxiety eq 2 then disut_ad=0.057;
else if anxiety eq 3 then disut_ad=0.123;
else if anxiety eq 4 then disut_ad=0.299;
else if anxiety eq 5 then disut_ad=0.321;

disut_total=disut_mo+disut_sc+disut_ua+disut_pd+disut_ad;
EQindex=1-disut_total;
run;
```

Final version of

statistical analysis plan

STATISTICAL ANALYSIS PLAN

VERSION: 2 Final

DATE OF PLAN:

October 2, 2023

STUDY DRUG:

CK-3773274 (aficamten)

PROTOCOL NUMBER:

CY 6031

STUDY TITLE:

A Phase 3, Multi-Center, Randomized, Double-blind, Placebo- controlled Trial to Evaluate the Efficacy and Safety of CK-3773274 in Adults with Symptomatic Hypertrophic Cardiomyopathy and Left Ventricular Outflow Tract Obstruction

BASED ON:

Protocol Amendment 3, 03 January 2023

SPONSOR:

Cytokinetics, Inc.
350 Oyster Point Blvd., South San Francisco, CA 94080
650-624-3000

This study is being conducted in compliance with good clinical practice, including the archiving of essential documents.

SIGNATURE PAGE

This document has been prepared and/or reviewed by*:

Amy Wohltman, ME, Director, Biostatistics Cytokinetics, Inc.	Signature	Date

This document has been reviewed and accepted by*:

Lisa Meng, PhD, VP, Clinical Biometrics, Cytokinetics, Inc.	Signature	Date
Steve Heitner, MD, VP, Clinical Research and Therapeutic Area Lead Cardiovascular, Cytokinetics, Inc.	Signature	Date
Stuart Kupfer, MD, SVP, Chief Medical Officer, Cytokinetics, Inc.	Signature	Date
Christine Murray, SVP, Regulatory and Quality, Cytokinetics, Inc.	Signature	Date

*See electronic signatures at the end of the document.

TECHNICAL SUMMARY REPORT (TSR)

**Name of Sponsor/Company**<br>Cytokinetics, Inc.	**Individual Study Table Referring to Part of the Dossier:**<br>**Volume:**	***(For National Authority Use Only)*:**
**Name of Finished Product:**<br>No generic or trade name assigned	**Page:**	
**Name of Active Ingredient:**<br>Aficamten (CK-3773274)		
**Title of Study:** A Phase 3, Multi-Center, Randomized, Double-blind, Placebo- controlled Trial to Evaluate the Efficacy and Safety of CK-3773274 in Adults with Symptomatic Hypertrophic Cardiomyopathy and Left Ventricular Outflow Tract Obstruction		
**Investigators**:<br>Study Center(s): Patients will be enrolled from approximately 105 sites worldwide.		
**Studied period (years):** 2022 to 2023	**Phase of development:** Phase 3	

Objectives and Endpoints:

Objectives	***Endpoint(s)***
Primary	
• To evaluate the effect of CK-3773274 on exercise capacity in patients with symptomatic oHCM	• Change in peak oxygen uptake (pVO2) by cardiopulmonary exercise testing (CPET) from baseline to Week 24
Secondary	
• To evaluate the effect of CK-3773274 on patient health status	• Change in Kansas City Cardiomyopathy Questionnaire – Clinical Summary Score (KCCQ-CSS) from baseline to Week 12 and Week 24
• To evaluate the effect of CK-3773274 on New York Heart Association (NYHA) Functional Classification	• Proportion of patients with ≥1 class improvement in NYHA Functional Class from baseline to Week 12 and Week 24
• To evaluate the effect of CK-3773274 on post-Valsalva left ventricular outflow tract gradients (LVOT-G)	• Change in post-Valsalva LVOT-G from baseline to Week 12 and Week 24<br>• Proportion of patients with post-Valsalva LVOT-G <30 mmHg at Week 12 and Week 24
• To evaluate the effect of CK-3773274 on duration of eligibility for septal reduction therapy	• Total duration of septal reduction therapy (SRT) eligible during the 24 Week treatment period in patients who were SRT eligible at baseline
• To evaluate the effect of CK-3773274 on exercise capacity	• Change in total workload during CPET from baseline to Week 24

Methodology:

This is a Phase 3 randomized, placebo-controlled, double-blind, multi-center trial in patients with symptomatic oHCM. Approximately 270 eligible patients will be randomized in a 1:1 ratio to receive CK-3773274 or placebo. Doses of 5, 10, 15, or 20 mg or matching placebo will be administered in an escalating manner using echocardiography to guide dose titration. Randomization will be stratified by use of beta-blockers and CPET exercise modality.

The trial will comprise three periods. The screening period will be up to 6 weeks in duration. The double-blind placebo-controlled treatment period will last 24 weeks. Following the final dose of investigational product (IP), there will be a 4-week safety follow-up period. IP will be administered orally once daily. During the initial six weeks of the treatment period, IP doses will be individually titrated at Weeks 2, 4, and 6 using echocardiography. Dose escalation at the Weeks 2, 4, and 6 visits will occur only if a patient has a post-Valsalva LVOT-G ≥30 mmHg and a biplane LVEF ≥55%. An echocardiogram will be performed at each subsequent visit during the trial and the dose down-titrated if necessary. The primary endpoint of pVO2 will be measured by CPET at screening and at end of treatment (Week 24).

Number of Subjects (planned and analyzed): Approximately 270 patients will be randomized to CK-3773274 or placebo at 1:1 ratio.

Diagnosis and main criteria for inclusion:

The key inclusion criteria are below. A full listing of eligibility criteria can be found in protocol Section 5.

- Males and females between 18 and 85 years of age, inclusive, at screening.
- Body mass index <35 kg/m^2.
- Diagnosed with HCM per the following criteria:
  − Has LV hypertrophy and non-dilated LV chamber in the absence of other cardiac disease and
  − Has an end-diastolic LV wall thickness as measured by the echocardiography core laboratory of:
    - ≥15 mm in one or more myocardial segments OR
    - ≥13 mm in one or more wall segments and a known-disease-causing gene mutation or positive family history of HCM
- Has resting LVOT-G ≥30 mmHg and post-Valsalva LVOT-G ≥50 mmHg during screening as determined by the echocardiography core laboratory.
- LVEF ≥60% at screening as determined by the echocardiography core laboratory.
- NYHA Functional Class II or III at screening.
- Hemoglobin ≥10g/dL at screening.
- Respiratory exchange ratio (RER) ≥1.05 and pVO_2 ≤90% predicted on the screening CPET per the core laboratory.
- Patients on beta-blockers, verapamil, diltiazem, or disopyramide should have been on stable doses for >6 weeks prior to randomization and anticipate remaining on the same medication regimen during the trial. Patients treated with disopyramide must also be concomitantly treated with a beta blocker and/or calcium channel blocker

Test product, dose and mode of administration:

Aficamten will be administered orally once daily with or without food. Patients receiving CK-3773274 start at a dose of 5 mg once daily and may escalate through doses of 10, 15, and 20 mg once daily during the initial six weeks of treatment if they continue to meet the escalation criteria (post-Valsalva LVOT-G ≥30 mmHg and a biplane LVEF ≥55%) or will stop at their current dose when escalation criteria are not met.

Duration of treatment:

After signing the informed consent form, patients will complete assessments to determine trial eligibility during a screening period of up to 6 weeks in duration. The double-blind placebo-controlled treatment period will last 24 weeks. Following the final dose of IP, there will be a 4-week safety follow-up period.

Reference therapy, dose and mode of administration:

Doses of 5, 10, 15, or 20 mg or matching placebo will be administered in an escalating manner using echocardiography to guide dose titration.

Criteria for evaluation (see protocol Section 3):

Efficacy:

The primary efficacy endpoint is change in peak oxygen uptake (pVO_2) by cardiopulmonary exercise testing (CPET) from baseline to Week 24.

The secondary endpoints are as follows:

- Change in Kansas City Cardiomyopathy Questionnaire – Clinical Summary Score (KCCQ-CSS) from baseline to Week 12 and Week 24
- Proportion of patients with ≥1 class improvement in NYHA Functional Class from baseline to Week 12 and Week 24
- Change in post-Valsalva LVOT-G from baseline to Week 12 and Week 24
- Proportion of patients with post-Valsalva LVOT-G <30 mmHg at Weeks 12 and 24
- Change in total workload during CPET from baseline to Week 24
- Total duration of SRT eligible during the 24 Week treatment period in patients who were SRT eligible at baseline

Safety:

- Incidence of reported major adverse cardiac events (cardiovascular [CV] death, cardiac arrest, non-fatal stroke, non-fatal myocardial infarction, CV hospitalization)
- Incidence of new onset persistent atrial fibrillation
- Incidence of appropriate implantable cardiac defibrillator (ICD) discharges and aborted sudden cardiac death
- Incidence of left ventricular ejection fraction (LVEF) <50%
- Incidence of treatment emergent adverse events

Statistical methods:

Unless specified otherwise, efficacy analyses will be performed on the full analysis set (FAS), which includes all randomized patients. The primary analysis will test the null hypothesis that there is no treatment difference in the primary endpoint between patients randomized to placebo and those randomized to CK-3773274 in the FAS. Change from baseline in pVO2 will be analyzed using an ANCOVA model with treatment group, randomization stratification factors, baseline pVO2 and baseline weight as covariates.

For preservation of the overall type I error rate at two-sided 0.05 for the primary and secondary endpoints, the primary and secondary endpoints will be tested in the following specified order using a closed testing procedure. The primary endpoint is tested first at two-sided 0.05. If the primary endpoint achieves statistical significance at two-sided p<0.05, then the secondary endpoints will be tested at two-sided 0.05, with their testing being in the sequential order of KCCQ-CSS change from baseline, proportion of patients with ≥1 NYHA functional class improvement, post-Valsalva LVOT-G change from baseline, and proportion of patients with post-Valsalva LVOT-G < 30 mmHg; duration of SRT eligibility for participants who are SRT eligible at baseline, for each after 24 weeks of treatment; then KCCQ-CSS change from baseline, proportion of patients with ≥1 NYHA functional class improvement, post-Valsalva LVOT-G change from baseline, and proportion of patients with post-Valsalva LVOT-G < 30 mmHg, for each after 12 weeks of treatment; and lastly change from baseline to Week 24 in total workload. SRT eligibility is defined as resting or post-Valsalva LVOT-G ≥ 50 mmHg AND NYHA Functional Class ≥ 3. See below table for illustration of the testing order.

Primary Endpoint		Significance Level
Step 1	pVO_2	0.05
Secondary Endpoints		↓
Step 2	KCCQ-CSS (24 wk)	0.05
		↓
Step 3	NYHA Class (24 wk)	0.05
		↓
Step 4	Valsalva Gradient (24 wk)	0.05
		↓
Step 5	%Valsalva Gradient (24 wk)	0.05
		↓
Step 6	Duration SRT Eligible (24 wk)	0.05
		↓
Step 7	KCCQ-CSS (12 wk)	0.05
		↓
Step 8	NYHA Class (12 wk)	0.05
		↓
Step 9	Valsalva Gradient (12 wk)	0.05
		↓
Step 10	%Valsalva Gradient (12 wk)	0.05
		↓
Step 11	Total workload (24 wk)	0.05

Multiplicity will be detailed in Section 7.6.

The proportion of responders in various exploratory endpoints in FAS will be analyzed using Cochran–Mantel–Haenszel (CMH) test stratified by randomization factors. The p-value and 95% confidence interval (CI) will be obtained using exact method. Other change from baseline endpoints will be

analyzed using mixed measures repeated model with treatment, visit, randomization stratification factors, treatment by visit, baseline by visit interaction as fixed effect and baseline assessment as covariate. Total of SRT eligible will be analyzed using an ANCOVA model with treatment group and randomization stratification factor beta blocker use/no use as fixed effects adjusting for significant baseline characteristics.

Safety analyses will be performed on the safety analysis set (SAS) which includes all patients who received at least one dose of IP. The pharmacokinetics analysis set (PKS) will consist of patients who have at least one evaluable plasma concentration of CK-3773274.

The number and percentage of patients reporting any treatment-emergent AEs will be coded using the MedDRA dictionary and be tabulated by system organ class and preferred term.

TABLE OF CONTENTS

SIGNATURE PAGE 2
TECHNICAL SUMMARY REPORT (TSR).....3
TABLE OF CONTENTS.....8
LIST OF TABLES.....11
LIST OF ABBREVIATIONS.....12
SAP VERSION HISTORY 14
1. INTRODUCTION 16
2. STUDY OBJECTIVES AND ENDPOINTS.....17
2.1. Study Objectives.....17
2.1.1. Primary Objective.....17
2.1.2. Secondary Objective.....17
2.2. Study Endpoints.....17
2.2.1. Primary Endpoints 17
2.2.2. Secondary Endpoints 17
2.2.3. Exploratory Endpoints.....17
2.2.4. Safety Endpoints.....19
2.2.5. PK Parameters 20
3. STUDY DESIGN 21
3.1. Summary of Study Design.....21
3.2. Definition of Study Drugs 21
3.3. Sample Size Considerations 22
3.3.1. Sample Size Justifications 22
3.4. Randomization.....22
3.5. Clinical Assessments 22
3.5.1. Efficacy Assessments 22
3.5.1.1. Echocardiography.....22
3.5.1.2. Patient-Reported Outcomes.....24
3.5.1.3. CMR Assessments.....25
3.5.1.4. CPET Assessments.....27
3.5.2. Safety Assessments.....27
3.5.3. Pharmacokinetics Assessments 27

4. PLANNED ANALYSES 28
4.1. Interim Analyses 28
4.2. Final Analyses 28
5. GENERAL CONSIDERATIONS FOR DATA ANALYSES AND HANDLING 29
5.1. General Summary Table and Individual Subject Data Listing Considerations 29
5.2. General Post Text Summary Table and Individual Subject Data Listing Format Considerations 29
5.3. Data Management 29
5.4. Data Presentation Conventions 29
5.5. Analysis Populations 30
5.5.1. All Screened Patients 30
5.5.2. All Randomized Set 30
5.5.3. Safety Analysis Set 30
5.5.4. Full Analysis Set 31
5.5.5. Pharmacokinetics Analysis Set 31
5.6. Baseline Definition 31
5.7. Derived and Transformed Data 31
5.7.1. Baseline Age 31
5.7.2. Body Measurements Variable Derivation 31
5.7.3. Study Day 31
5.7.4. Change from Baseline 31
5.7.5. Summary Scores for Patient Reported Outcomes (PRO) 32
5.7.6. Analysis Windows 32
5.7.7. Multiple Assessments 32
5.7.8. Other Study Related Definitions 32
5.7.9. Derived Echocardiographic Parameters 33
5.8. Handling of Missing Data 33
5.8.1. Missing Efficacy Endpoints 33
5.8.2. Missing Start and Stop Dates for Prior and Concomitant Medication 34
5.8.3. Missing Start and Stop Dates for Adverse Events 34
6. STUDY POPULATION 35
6.1. Subjects Disposition 35

6.2. Screen Failures....35
6.3. Protocol Deviations....35
6.4. Demographic and Baseline Characteristics....35
6.5. Listing of Subject Inclusion and Exclusion Criteria....36
6.6. Medical History....36
6.7. Baseline Medications Use....36
7. EFFICACY....37
7.1. General Considerations....37
7.2. Testing Statistical Assumptions Including Comparability at Baseline....37
7.3. Statement of the Null and Alternate Hypotheses....37
7.4. Planned Covariates....37
7.5. Subgroup Analyses....37
7.6. Multiple Comparisons and Multiplicity....38
7.7. Analysis of the Primary Efficacy Endpoint....40
7.7.1. Primary Efficacy Analysis....40
7.7.2. Sensitivity Analyses of the Primary Efficacy Endpoint....42
7.7.3. Subgroup Analyses for the Primary Endpoint....42
7.7.4. Supportive Analyses for the Primary Endpoint....42
7.8. Analysis of the Secondary Efficacy Endpoints....43
7.8.1. Analysis of the Secondary Efficacy Endpoints....43
7.9. Analysis of the Exploratory Efficacy Endpoints....49
7.9.1. Analysis of the Exploratory Efficacy Endpoint....49
8. SAFETY AND TOLERABILITY....51
8.1. Overall Summary of Tolerability....51
8.2. Adverse Event Preferred Term and Body/Organ System Summary Tables....51
8.2.1. Summaries of Adverse Event Incidence Rates for All Subjects....51
8.2.2. Summaries of Adverse Events of Special Interest....52
8.3. Total Duration of Therapy, Final Daily Dose of Study Medication, and Compliance....52
8.3.1. Summary of IP Exposure and Overall Compliance....52
8.3.2. Summary of Dose Titration....52
8.4. Concomitant and Other Medications....53
8.5. Routine Laboratory Data....53

8.6. Vital Signs 53
8.7. Electrocardiogram 54
9. PHARMACOKINETICS 56
10. REFERENCES 57
11. APPENDIX 58
11.1. Patient-reported Outcome Scoring Algorithm 58
11.1.1. KCCQ 58
11.1.2. SAQ-7 61
11.1.3. EQ-5D-5L 63
11.2. Table of Contents for Data Display Specifications 64
11.3. Data Display Specifications 64
11.4. Analysis Windows 64
11.5. Sample SAS Codes 64

LIST OF TABLES

Table 1: Investigational Products 21
Table 2: Echocardiographic Variables and Names 22
Table 3: CMR Parameters 25
Table 4: Testing Steps 39
Table 5: Estimands for Primary Endpoint 40
Table 6: Endpoint Summary Table 46
Table 7: EQ-5D-5L Value Set 63
Table 8: Analysis Windows for Measurements 64

LIST OF ABBREVIATIONS

Abbreviation/Term	Explanation
AE	Adverse event
ALP	Alkaline phosphatase
ALT	Alanine aminotransferase
ANCOVA	Analysis of Covariance
AST	Aspartate aminotransferase
BSA	Baseline body surface area
CGI	Clinical Global Impression scale
CI	Confidence interval
CMH	Cochran–Mantel–Haenszel
CMR	Cardiac magnetic resonance
CPET	Cardiopulmonary exercise testing
CRF	Case report form
CSR	Clinical Study Report
CSS	Clinical Summary Score
cTTO	composite time trade-off
CV	Cardiovascular
CV%	Coefficient of Variation
ECG	Electrocardiogram
eCRF	Electronic Case Report Form
EQ-5D	EuroQol 5-dimension instrument
EQ-5D-5L	EuroQol 5-dimension 5-level instrument
EQ-VAS	EuroQol - Visual Analogue Scale
FAS	Full analysis set
HCM	Hypertrophic cardiomyopathy
hs-cTnI	High sensitivity cardiac troponin I
ICD	Implantable cardioverter defibrillators
ICF	Informed consent form
ICH	International Council for Harmonisation
ID	Identifier
IP	Investigational product
IWRS	Interactive web response system
KCCQ	Kansas City Cardiomyopathy Questionnaire
LLN	Lower Limit of Normal
LSM	Least Squares Mean
LV	Left ventricle(ular)
LVEDV	Left ventricular end-diastolic volume

**Abbreviation/Term**	**Explanation**
LVEF	Left ventricular ejection fraction
LVESV	Left ventricular end-systolic volume
LVOT	Left ventricular outflow tract
LVOT-G	Left ventricular outflow tract gradient
MAR	Missing At Random
MedDRA	Medical Dictionary for Regulatory Activities Terminology
MMRM	Mixed Model for Repeated Measures
MNAR	Missing Not At Random
NT-proBNP	n-terminal prohormone brain natriuretic peptide
NYHA	New York Heart Association
oHCM	Obstructive hypertrophic cardiomyopathy
PDCC	Protocol deviation classification committee
PGI-C	Patient Global Impression of Change scale
PK	Pharmacokinetics
PKS	Pharmacokinetics analysis set
PRO	Patient reported outcomes
PT	Preferred Term
pVO2	Peak oxygen uptake
QTcF	Fridericia corrected QT
RER	Respiratory exchange ratio
SAE	Serious Adverse Event
SAP	Statistical Analysis Plan
SAQ-7	Seattle Angina Questionnaire -7
SAS	Statistical Analysis System (SAS®)
SD	Standard deviation
SOC	System Organ Class
SRT	Septal Reduction Therapy
TEAE	Treatment-Emergent Adverse Event
TESAE	Treatment-Emergent Serious Adverse Event
TSS	Total Symptom Score
ULN	Upper Limit of Normal
VAS	Visual Analogue Scale
VAT	Ventilatory anaerobic threshold
WHO	World Health Organization

SAP VERSION HISTORY

Version and Date	Revision	Rationale
Final 1.0	Not applicable: original version	Not applicable
Version 2.0	Synopsis and Section 7.6: updated testing hierarchy and removed the statement related to determination of the testing order of the duration of SRT eligibility and total workload.	The testing hierarchy was simplified from parallel gate keeping method to a closed testing procedure to allow the testing of the secondary endpoints including SRT eligibility at Week 24 at two-sided alpha level of 0.05 once the primary endpoint reaches statistical significance at a specified sequential testing order.
	Section 2.2.3: corrected typo in exploratory endpoints:<br>number of patients with new or worsening ST depression during exercise at Week 12 and 24 by removing time point Week 12<br><br>added 1 exploratory efficacy endpoint	CPET is only performed at Week 24.
	Section 2.2.4: updated the definition of the endpoint of incidence of LVEF <50% with signs and symptoms of heart failure (concomitant adverse event of heart failure or dyspnea) and/or increase in NT-proBNP from baseline	Update was made to provide greater specificity to the increase in NT-proBNP (≥30% increase) in relation to worsening HF.
	Section 5.5.4: modified FAS definition by removing condition of requiring at least one post baseline efficacy measurement	Update was made to address FDA comments to the SAP.
	Section 7.2: added statement that if model assumptions are substantially violated, rank based analysis will be performed as supportive analysis.	Updates were made to address FDA comments to the SAP.
	Section 7.5: added additional analyses by IND Sites status	Updates were made to add additional analyses to have consistent scope of by IND sites status analyses
	Section 7.8: Remove COVID-19 sensitivity analyses from the secondary endpoints	The impact of COVID-19 reported on the study has been minimal.

Version and Date	Revision	Rationale
	Section 7.7.1: updated the reasons of invalid CPET	To use the same statements provided by the CPET laboratory used in the data transfer specification
	Section 7.7.4: updated to include criteria of condition when rank-based analysis will be performed and clarified that, if performed, rank-based analysis will be supportive analysis for the primary endpoint	Updates were made to address FDA comments to the SAP and provide specific criteria for the rank-based analysis to be used.
	Section 7.8.1: update the number of imputed datasets from 50 to 100	Updates were made per FDA comments to the SAP
	Section 7.8.1: clarified imputing missing Week 12 NYHA.<br>Added sensitivity analysis for proportion of responders at Week 12 and 24 after treating patients with missing data as non-responder. Added statement to impute intermittent missing data use adjacent visits results in determining patients' SRT eligibility.	Updates were made per FDA comments to the SAP; added clarification statements on imputing intermittent missing data except Week 12 and Week 24 in determining patient's SRT eligibility
	Editorial updates made where applicable	To correct editorial issues

1. INTRODUCTION

The purpose of this statistical analysis plan (SAP) is to provide a technical elaboration of the planned analyses and detailed data displays to be included in the Clinical Study Report (CSR) for CY 6031 study.

This SAP was developed in accordance with International Council for Harmonisation (ICH) E9 and ICH E9 (R1) guideline. All decisions regarding final analysis, as defined in this SAP document, will be made prior to the study database lock. Further study information can be found in the protocol.

2. STUDY OBJECTIVES AND ENDPOINTS

2.1. Study Objectives

2.1.1. Primary Objective

To evaluate the effect of CK-3773274 on exercise capacity in patients with symptomatic obstructive hypertrophic cardiomyopathy (oHCM).

2.1.2. Secondary Objective

The secondary objectives of the study are listed as follows:

- To evaluate the effect of CK-3773274 on patient health status
- To evaluate the effect of CK-3773274 on New York Heart Association (NYHA) Functional Classification
- To evaluate the effect of CK-3773274 on post-Valsalva left ventricular outflow tract gradients (LVOT-G)
- To evaluate the effect of CK-3773274 on exercise capacity
- To evaluate the effect of CK-3773274 on duration of eligibility for SRT

2.2. Study Endpoints

2.2.1. Primary Endpoints

The primary endpoint of the study is Change in peak oxygen uptake (pVO2) by cardiopulmonary exercise testing (CPET) from baseline to Week 24.

2.2.2. Secondary Endpoints

- Change in Kansas City Cardiomyopathy Questionnaire – Clinical Summary Score (KCCQ-CSS) from baseline to Week 12 and Week 24
- Proportion of patients with ≥1 class improvement in NYHA Functional Class from baseline to Week 12 and Week 24
- Change in post-Valsalva LVOT-G from baseline to Week 12 and Week 24
- Proportion of patients with post-Valsalva LVOT-G <30 mmHg at Week 12 and Week 24
- Total duration of SRT eligibility during the 24-Week treatment period in patients who were SRT eligible at baseline
- Change in total workload during CPET from baseline to Week 24

2.2.3. Exploratory Endpoints

- Compared with baseline, proportion of patients at Week 24 achieving either:

  − Change from baseline of ≥1.5 mL/kg/min in pVO2 AND ≥1 class improvement in NYHA Functional Class

OR

− Change from baseline of ≥3.0 mL/kg/min in pVO2 AND no worsening of NYHA Functional Class

- Proportion of patients with >3 mL/kg/min improvement in pVO2 and ≥1 class improvement in NYHA Functional Class
- Proportion of patients with improvement of ≥5, 10, 15 and 20 points in KCCQ-CSS and KCCQ- Total Symptom Score (TSS) at Weeks 12 and 24
- Proportion of patients with resting LVOT-G <30 mmHg, post-Valsalva LVOT-G <50 mmHg, and NYHA Functional Class I at Week 12 and Week 24
- Proportion of patients with resting LVOT-G <30 mmHg, post-Valsalva LVOT-G <50 mmHg, and ≥1 class improvement in NYHA Functional Class at Week 12 and Week 24
- Change from baseline to Week 24 in CPET parameters of:
  - Ventilatory efficiency (VE/VCO2 slope)
  - Circulatory power (VO2 × systolic BP)
  - Ventilatory anaerobic threshold (VAT)
- Proportion of patients who remain SRT eligible at Week 24 in patients who were eligible for SRT at baseline. Proportion of patients who remain SRT eligible will also be evaluated at other scheduled visit weeks
- Time to first SRT ineligibility status in patients who were SRT eligible at baseline
- Change from baseline to Week 24 in individual responses to the EuroQol 5-dimension 5-level instrument (EQ-5D-5L)
- Change from baseline to Week 24 in total score and domain scores for the Seattle Angina Questionnaire -7 (SAQ-7)
- Change from baseline to Week 24 in echocardiographic measurements of cardiac structure and of systolic function including:
  - Left ventricular ejection fraction (LVEF)
  - Left ventricular global longitudinal strain (LV GLS)
  - Left ventricular end-systolic and end-diastolic volumes (LVESV and LVEDV)
  - Left atrial volume index
  - Left ventricular mass index
  - Maximal wall thickness
- Change from baseline values in n-terminal prohormone brain natriuretic peptide (NT-proBNP), high sensitivity cardiac troponin I (hs-cTnI) and other biomarkers through Week 24; proportional change (post randomization/baseline) will also be calculated and used in the analysis

- Change from baseline to Week 24 in patients enrolled in Cardiac magnetic resonance (CMR) substudy in CMR measurements of:
    - Left ventricular (LV) mass index
    - LVEF
    - Septal, free wall and maximal wall thickness
    - Left atrial volume index
    - LVESV
    - LVEDV
    - Extracellular volume proportion
    - Late gadolinium enhancement proportion
    - Mitral regurgitation severity
- Time to Maximal ST Segment Depression on CPET ECG
- Time to 1 mm ST depression below Baseline on CPET ECG
- Maximal ST segment depression on resting ECG in mm at Weeks 12 and 24
- Number of patients with new or worsening ST depression during exercise at Week 24
- Change from baseline values in all other summary KCCQ scores (Physical Limitation, Symptom Stability, Symptom Frequency, Symptom Burden, Total Symptom Score, Self-efficacy, Quality of Life, Social Limitation, Overall Summary Score) at Weeks 12 and 24.
- Proportion of patients with LVH with strain pattern (typical+ atypical) on Electrocardiogram (ECG) at Weeks 12 and 24
- Proportion of patients with all LVH (with or without strain) on ECG at Weeks 12 and 24

2.2.4. Safety Endpoints

- Incidence of reported major adverse cardiac events (cardiovascular [CV] death, cardiac arrest, non-fatal stroke, non-fatal myocardial infarction, CV hospitalization)
- Incidence of new onset persistent atrial fibrillation
- Incidence of appropriate implantable cardiac defibrillator (ICD) discharges and aborted sudden cardiac death
- Incidence of LVEF <50% with at least one of the following:
    - Signs and symptoms of heart failure (concomitant adverse event of heart failure or dyspnea)

      AND/OR
    - Increase in NT-proBNP (≥30% increase), relative to results from the most recent previous visit and above the upper limit of normal, at the time of LVEF assessment <50%

Note signs and symptoms refer to AEs with onset date within ± 7 days relative to the date when LVEF <50%.

- Incidence of LVEF <40%
- Incidence of LVEF <50%

Incidence of LVEF below 40% and 50% will be summarized for site read, core lab read and both.

- Incidence of treatment emergent adverse events (TEAEs)

2.2.5. PK Parameters

- $C_{post\ dose}$ and $C_{pre\text{-}dose}$

3. STUDY DESIGN

3.1. Summary of Study Design

This is a Phase 3, randomized, placebo-controlled, double-blind, multi-center trial in patients with symptomatic oHCM. Approximately 270 eligible patients will be randomized in a 1:1 ratio to receive CK-3773274 or placebo. Randomization will be stratified by use of beta-blockers (yes or no) and CPET exercise modality (treadmill or bicycle) and implemented in the Interactive Web Response System (IWRS). A cap on the number of patients taking beta-blockers and will not exceed approximately 70% of total enrollment. The number of patients taking disopyramide will be capped at approximately 10% of total enrollment. The number of patients with persistent atrial fibrillation at screening will also be capped at approximately 15%, and the number of patients using the bicycle CPET exercise modality will be capped at approximately 50% as well.

Investigational product (IP) will be administered orally once daily with or without food. During the initial six weeks of the treatment period, IP doses will be individually titrated at Weeks 2, 4, and 6 using echocardiography. Dose escalation at Weeks 2, 4, and 6 will occur only if a patient has a post-Valsalva LVOT-G ≥30 mmHg and a biplane LVEF ≥55%. Echocardiograms will be performed at each subsequent visit during the trial and the dose down titrated if necessary. The primary endpoint of pVO2 will be measured by CPET at screening and at end of treatment (Week 24). If applicable, patients will continue taking background HCM medications consistent with regional clinical practice guidelines during the trial.

3.2. Definition of Study Drugs

Table 1 describes any study drug: IP (ie, aficamten) or placebo intended to be administered to a trial patient according to the protocol.

Table 1: Investigational Products

	Active	Placebo
**IP/Product Name**	CK-3773274	Placebo
**Type**	Drug	Drug
**Dose Formulation**	Tablet	Tablet
**Unit Dose Strength(s)**	5 mg	Matching placebo
**Dosage Level(s)**	5mg, 10mg, 15mg, 20 mg	
**Route of Administration**	Oral	Oral
**Use**	Experimental	Placebo
**IMP and NIMP**	IMP	IMP
**Packaging and Labeling**	IP will be provided in blister packs which will be labeled as required per country requirement	IP will be provided in blister packs which will be labeled as required per country requirement

3.3. Sample Size Considerations

3.3.1. Sample Size Justifications

Assuming a difference in change from baseline in pVO2 of 1.5 mL/kg/min for CK-3773274 compared to placebo, a standard deviation (SD) of 3.5 mL/kg/min, accounting for limiting beta-blocker use (less than ~70%), limiting exercise modality of bicycle (less than ~50%) and 10% of patients missing change from baseline data of the primary endpoint, a sample size of 270 patients at randomization ratio of 1:1 (approximately 135 randomized to CK-3773274 and 135 randomized to placebo) provides more than 90% power to detect the difference in pVO2 change from baseline to Week 24 with a 2-sided type I error of 0.05.

During the study, Cytokinetics will periodically assess in a blinded fashion the aggregate pooled missing data rate and overall pooled SD for the change from baseline in pVO2 at Week 24. If the pooled SD is larger than expected, Cytokinetics may consider increasing the sample size once in order to maintain the intended power.

3.4. Randomization

All eligible patients will be centrally assigned to randomized IP using the IWRS. Randomization will be stratified by use of beta-blockers (yes or no) and CPET exercise modality (treadmill or bicycle) and implemented in the IWRS.

3.5. Clinical Assessments

3.5.1. Efficacy Assessments

Efficacy assessments include CPET, echocardiography, NYHA classification, patient-reported outcomes (KCCQ, EQ-5D-5L), Patient Global Impression of Change scale (PGI-C) and SAQ-7, and clinical global impression scale (CGI). CMR measurements will be assessed in patients included in CMR sub-study.

3.5.1.1. Echocardiography

Echocardiography will be done during screening, prior to dosing on Day 1, and 2 hours after dosing in the clinic on Weeks 2, 4, 6, 8, 12, 16, 20, 24, and 28.

Site read echocardiographic assessments include LVEF, resting and Valsalva LVOT at each visit. Unless otherwise specified, echocardiographic variables will be based on the core echocardiography laboratory assessments. Table 2 below lists the echocardiography parameters from core lab. A full list of echocardiography parameters is specified in the data transfer agreement from core lab.

Table 2: Echocardiographic Variables and Names

**Endpoint Names**
**Variables Describing LV Structure**
Left ventricular end diastolic diameter
Left ventricular end diastolic volume Index

Table 2: Echocardiographic Variables and Names (Continued)

**Endpoint Names**
Left Ventricular End Systolic Volume Index
Left ventricular end systolic diameter
Left Ventricular Posterior Wall Thickness, End-diastole
LV Mass indexed
Interventricular Septum Thickness, End-Diastole
Left ventricular Maximal wall thickness
Left ventricular relative wall thickness
**Variable Describing LV Systolic Function**
Left ventricular ejection fraction
Left ventricular fractional shortening
Left ventricular stroke volume Index
Left ventricular cardiac output Index
Left Ventricular Isovolumetric Contraction Time
Left Ventricular Isovolumetric Relaxation Time
Left Ventricular Ejection Time
Left Ventricular Myocardial Performance Index
Left Ventricular Outflow Tract Velocity Time Integral
Left Ventricular Global Longitudinal Strain
Left Ventricular Global Circumferential Strain
Variables Describing LV Diastolic Function
Peak E Wave Velocity
Peak A Wave Velocity
Mitral Lateral Annular Early Diastolic Velocity
Mitral Septal Annular Early Diastolic Velocity
Mitral E/A Wave Velocity Ratio
Mitral E Wave to Lateral Annular Early Diastolic Velocity Ratio
Mitral E Wave to Septal Annular Early Diastolic Velocity Ratio
**LVOT Dynamic Gradient Assessment Variables**
Peak Left Ventricular Outflow Tract Pressure Gradient at Rest
Peak Left Ventricular Outflow Tract Pressure Gradient during Valsalva Maneuver

Table 2: Echocardiographic Variables and Names (Continued)

Endpoint Names
**Variables Describing LA Size and Function**
Left Atrial Width
Left Atrial Volume Index
**Variables Describing RV Size and Function**
Right Ventricular Outflow Tract Velocity Time Integral
Right Ventricular Myocardial Performance Index
Tricuspid annular plane systolic excursion
**Valvular Assessment Variables**
Presence of Mitral Regurgitation
Mitral Regurgitation Jet Area to Left Atrial Area Ratio
Presence of Mitral Systolic Anterior Motion

3.5.1.2. Patient-Reported Outcomes

KCCQ and EQ-5D-5L will be assessed at Day 1, Weeks 2, 4, 6, 8, 12, 16, 20, 24 and 28 (4 weeks after last dose at end of the study). SAQ-7 will be assessed at Day 1, Weeks 4, 8, 12, 16, 20, 24 and 28. PGI-C will be assessed at the Week 24. Algorithms to derive the scores KCCQ, EQ-5D-5L and SAQ-7 are in Section 11.1.

EQ-5D-5L and EQ-VAS

The instrument EQ-5D is a standardized measure of health status for clinical and economic appraisal (EuroQol Group 1990), which consists of two parts: a short descriptive system questionnaire (EQ-5D-3L) and a visual analogue scale (EQ-VAS).

EQ-5D-5L is the 5-level version of EQ-5D introduced to improve the instrument's sensitivity and to reduce ceiling effects (EuroQol Group 2009). The descriptive system comprises 5 dimensions: mobility, self-care, usual activities, pain/discomfort and anxiety/depression. Each dimension has 5 levels indicating no problems, slight problems, moderate problems, severe problems or extreme problems. Five responses with a response from each of the 5 dimensions form a 5-digit number that defines a patient's health state profile. A health state can potentially be assigned a summary index score based on societal preference weights (societal perspective) for the health state. The health state preferences often represent national or regional values and can therefore differ between countries/regions. The health state index scores will be calculated using the composite time trade-off (cTTO) method based on the United States valuation of EQ-5D-5L (Pickard 2019) for patients from the United States and for the FAS. The health state index score ranges from less than 0 to 1 with higher scores indicating higher health utility; a score 0 represents death, negative values represent worse than death, and 1 represents full health.

EQ-VAS rates a patient's perceived health on a vertical visual analogue scale from 0 to 100, where 0 represents the worst imaginable health and 100 represents the best imaginable health.

The VAS can be used as a quantitative measure of health outcome that reflects the patient's own judgement.

3.5.1.3. CMR Assessments

A CMR imaging sub-study will assess the effects of administration of CK-3773274 dosing on cardiac morphology, function, and fibrosis in approximately 100 oHCM patients who are eligible and consent to participate. CMR will be performed during screening period and Week 24. CMR parameters are listed below in Table 3 and a final list of CMR parameters is specified in the data transfer agreement from core lab.

Table 3: CMR Parameters

**LV Parameters**
LVM - Left Ventricular Mass
LVMI Left Ventricular Mass Index
LVEDV - Left Ventricular End Diastolic Volume
LVEDVI - Left Ventricular End Diastolic Volume Index
LVESV - Left Ventricular End Systolic Volume
LVESVI - Left Ventricular End Systolic Volume Index
LVSV– Left Ventricular Stroke Volume=LVEDV-LVESV
LVSVI = Left Ventricular Stroke Volume Index=LVSV/Body Surface Area (mm2)
LVEF - Left ventricular Ejection Fraction
CO - Cardiac Output = Heart Rate x Stroke Volume
CI - Cardiac Index = Heart Rate x Stroke volume/Body Surface Area (m2)
LV maximal septal wall thickness
LV maximal lateral wall thickness
LV maximal wall thickness (each of 16 segments)
Overall LV maximal wall thickness (highest across all 16 segments)
LGE mass (g) (Global mass of LGE 6SD)
LGE mass % (as % of LV mass) (Global percent of LGE 6SD)
Global average ECVF

Table 3: CMR Parameters (Continued)

**LV Parameters**
LV Segmental assessment of ECVF
Global Native T1
Extracellular volume (ECV)
Global ECV mass
Global ECV mass index
ECVF normalized for height
HCM morphology (isolated basal septal hypertrophy, reverse septal curvature, apical, midcavity obstruction without apical aneurysm, midcavity obstruction with apical aneurysm, concentric, other
**RV Parameters**
RV end-diastolic volume (RVEDV)
RV end-diastolic volume index (RVEDVI)
RV end-systolic volume (RVESV)
RV end-systolic volume index (RVESVI)
RV stroke volume (RVSV)
RV stroke volume index (RVSVI)
RV ejection fraction (RVEF)
**LA parameters**
LA volume maximum
LA volume maximum index
LA reservoir amount
LA reservoir percent
LA contractile amount
LA contractile percent
LA total amount
LA total percent
LA global longitudinal strain
**Mitral Valve Parameters**
Exploratory Mitral Valve Regurgitation Measurements
Mitral regurgitation volume
Mitral regurgitation, regurgitation fraction

3.5.1.4. CPET Assessments

All patients will undergo CPET with gas-exchange analysis and the methodology will be standardized across all sites as specified in the CPET manual. Patients must use the same testing modality for all exercise tests during the trial. CPET are done at baseline and Week 24 post randomization. CPET parameters are listed below. A full list of CPET parameters is specified in the data transfer agreement from core lab.

- Workload
- Exercise Duration
- % of Predicted Oxygen Uptake
- Circulatory Power
- Predicted Oxygen Uptake
- Peak Oxygen Uptake per Kilogram
- Peak RER
- Oxygen Uptake Efficiency Slope
- Ventilatory Efficiency
- Anaerobic Threshold
- Aerobic Efficiency

3.5.2. Safety Assessments

Safety assessments include adverse events and serious adverse events (SAEs), ICD discharge, LVEF < 50% and <40%, electrocardiograms, laboratory assessments, physical examinations, and vital signs.

3.5.3. Pharmacokinetics Assessments

Blood samples will be collected to evaluate plasma concentrations of CK-3773274 at pre-dose and 2 hours post-dose at Day 1, Weeks 2, 4, 6, 8, 12, 16, 20 and 24.

4. PLANNED ANALYSES

4.1. Interim Analyses

No interim analysis is planned for this study.

4.2. Final Analyses

The final analysis will occur after all patients randomized in the study have completed the study including the 4-week safety follow up, all data has been entered into the clinical database, verified, and locked. Unblinding for the final analysis will occur after the database lock.

5. GENERAL CONSIDERATIONS FOR DATA ANALYSES AND HANDLING

5.1. General Summary Table and Individual Subject Data Listing Considerations

Descriptive statistics to be presented in a table include number of observations, mean, median, standard deviation, 1st and 3rd quartiles, minimum and maximum for continuous variables, and count of patients and the percentage for categorical variables. For variables that lognormal distribution assumptions may be appropriate geometric mean, and geometric coefficient of variation (CV%) will also be displayed. Geometric CV (%) will be derived as $100\% \times \text{sqrt}(\exp(s^2)–1)$, where s is the standard deviation of the natural logarithm (ln) transformed data.

For model-based analysis, least squares means (LSM), difference of least squares means between treatments, their standard errors and 95% confidence intervals (CI), and two-sided p-values for the statistical inferences will be presented.

Selected listings may be generated to include patient identifier (ID), demographics, randomized treatment group and other relevant items, and sorted by randomized treatment group, patient ID and date of assessment.

5.2. General Post Text Summary Table and Individual Subject Data Listing Format Considerations

Post text tables and individual subject data listings are prepared according to ICH Guideline E3. In general, summary and analysis tables will be presented by treatment groups and highest dose level administered.

5.3. Data Management

Data will be entered into the clinical database with programmed edit checks and manual data review to ensure integrity. The data will be reviewed and cleaned according to a data management plan. Clinical safety laboratory, ECG, Pharmacokinetics (PK) data, CMR, CPET and echocardiography will be provided per the pre-specified data transfer agreement from external laboratories.

5.4. Data Presentation Conventions

The following conventions will be applied to data presentations:

- For continuous variables, mean and median values are formatted to one more decimal place than the measured value. Standard deviation values are formatted to two more decimal places than the measured value. Minimum and maximum values are presented with the same number of decimal places as the measured value. For the statistical analyses results that are on the same scale as a measured value (e.g., change from baseline or treatment difference estimates), the LSM estimates and LSM estimate 95% CI boundary values will be formatted to one more decimal place than the measured value; Standard error of the mean (SEM) estimates will be formatted to two more decimal

places than the measured values. GLSM estimates for the proportional change from baseline, proportional change treatment ratios, odds ratios, and the corresponding 95% CIs will be presented with two decimal places.

- For categorical variables, the count and percentage of responses are presented in the form XX (XX.X%) where the percentage is in the parentheses.
- Date variables are formatted as YYYY-MM-DD for presentation. Time is formatted in military time as HH:MM for presentation.
- P-values, if applicable, will be presented to 3 decimal places. If the p-value is less than 0.0001 then it will be presented as <0.0001. If the rounded result is a value of 1.000, it will be displayed as >0.999.
- Unless otherwise stated, any statistical tests performed will use 2-sided tests at the 0.05 significance level.

The table and listing shells and table of contents provide the expected layout and titles of the tables, listings and figures. Any changes to format, layout, titles, numbering, or any other minor deviation will not necessitate a revision to the SAP, nor will it be considered a deviation from planned analyses. Only substantial deviation in the analysis methods will require an SAP revision or a change to planned analysis documented in the CSR.

5.5. Analysis Populations

5.5.1. All Screened Patients

All patients who signed the informed consent form (ICF) are included in the All Screened Patients Set. Patients who gave informed consent but are not randomized are considered screen failures. The following reasons for screen failures are collected: inclusion/exclusion criteria (including specific criteria not met), principal investigator decision, subject decision, lost to follow up, and other. For patients who are screen failures, the reasons for failing will be summarized.

5.5.2. All Randomized Set

All Randomized Set includes patients who are randomized to receive CK-3773274 or placebo.

5.5.3. Safety Analysis Set

Safety analyses will be performed on the safety analysis set (SAF), which includes all randomized patients who receive at least one dose of IP, CK-3773274 or placebo. Unless otherwise specified, for safety analyses, subjects will be grouped according to their randomized treatment group assignment with the following exception: if a subject receives treatment throughout the study that is different than the randomized treatment group assignment, then the subject will be grouped by the actual treatment group.

5.5.4. Full Analysis Set

Efficacy analyses will be performed on the full analysis set (FAS), which includes all randomized patients. Patients will be analyzed according to their randomized treatment group assignments.

5.5.5. Pharmacokinetics Analysis Set

All randomized patients who have at least one evaluable plasma concentration of CK-3773274, provided they have no major protocol deviations that could affect the PK of CK-3773274.

5.6. Baseline Definition

Unless otherwise specified, baseline is defined as the last available measurement taken prior to administration of the first dose of study drug. Baseline for KCCQ, EQ-5D-5L and SAQ-7 are assessments performed on Day 1. The assessments collected on the same date as the first dose of the IP that do not have assessment time are considered to have occurred prior to the first dose.

5.7. Derived and Transformed Data

5.7.1. Baseline Age

Age will be calculated as follows:

Age (years) = year of screening date – year of birth,

Patient age will be categorized as < 65 years or ≥ 65 years.

5.7.2. Body Measurements Variable Derivation

Baseline body surface area (BSA) will be calculated using the weight and height at screening using the DuBois and DuBois formula and rounded to two decimal points for the presentation of results:

BSA (m^2) = 0.007184 * (weight (kg) $^{0.425}$ * height (cm) $^{0.725}$).

5.7.3. Study Day

If the date of interest occurs on or after the first dose date, then study day will be calculated as (date of interest – date of first dose) + 1. If the date of interest occurs prior to the first dose date, then study day will be calculated as (date of interest – date of first dose). There is no study day 0.

5.7.4. Change from Baseline

Change from baseline is calculated as (post baseline value – baseline value).

Percent change from baseline is calculated as (change from baseline / baseline value) x 100%.

Proportional change from baseline is calculated as (post-baseline result/baseline value).

If either the baseline or the post-baseline value is missing, the change from baseline and percentage change from baseline will be set to missing.

5.7.5. Summary Scores for Patient Reported Outcomes (PRO)

Calculations of summary scores for KCCQ, SAQ-7 and EQ-5D-5L are specified in Section 11.1.

5.7.6. Analysis Windows

Since study visits do not always take place exactly as scheduled per protocol, it is necessary to assign the actual observation dates to analysis windows for analysis purposes.

For data collected at a scheduled post baseline visit, the analysis visit will be assigned based on the scheduled nominal visit as collected on the eCRF.

For unscheduled or early discontinuation post baseline visits, measurements taken on or after the first dose of study drug will be assigned to an analysis window using defined lower and upper bounds for each analysis window. Measurements assigned in an analysis window will have study day greater than or equal to the lower bound but no greater than the upper bound of the analysis window. The lower bound and the upper bound for the analysis windows are defined as the midpoints between the scheduled visits for all assessments (see Section 11.4).

Visits are identified as the nominal visits according to the eCRFs. Each visit will be identified with the visit descriptor (eg, “Week 24”).

5.7.7. Multiple Assessments

Once analysis windows are assigned, a patient’s individual analysis window could potentially contain more than one visit. Records from all visits, including scheduled, unscheduled and early discontinuation visits could be flagged as the “analyzed record” within the analysis window, although the records from scheduled visit will take priority.

In the event of multiple visits falling within an analysis window, the following rules will be used in sequence to determine the “analyzed record” for the analysis window:

- If a scheduled visit occurred during the analysis window, then the measurement taken from the scheduled visit will be used.
- If no scheduled visit occurred during the analysis window, the measurement taken closest to the scheduled day will be used as the “analyzed record.”
- If no scheduled visit occurred during the analysis window and there is a tie between unscheduled visits in the number of days before and after the scheduled day, measurements from the later visit will be used as the “analyzed record.”

For analyses by visit, only the “analyzed record” within each analysis window and the visit will be summarized in a table. Only protocol specified visits will be presented in the summary table. If there are other visit records within the analysis window, they will only be included in data listings.

5.7.8. Other Study Related Definitions

<u>Actual Dose Group</u>

Patients in the aficamten actual treatment group will be identified as 5 mg, 10 mg, 15 mg, 20mg or discontinuing IP prior to dose adjustment based on the dose assigned at Week 8. If a patient discontinues IP prior to the start of Week 8 (IWRS Week 8 dispensation), then the subject will be

identified as discontinued IP prior to achieve stable dose. The actual dose group may be used in selected displays.

Investigational Product Exposure Period

For subjects dosed with IP:
[(Last IP administration date – date of Study Day 1) + 1]/7 (in weeks)
Interruptions recorded in the eCRF page will be excluded from the expected dose calculation.

Treatment-emergent Adverse Event

For the purpose of reporting, an investigator-reported event starting on or after first dose of IP and up to and including 28 days after the last dose date of IP will be labeled as a treatment-emergent AE.

Last Titrated Dose

Last titrated dose is defined as the last titrated dose assigned to the patient during the 24 Week treatment period.

5.7.9. Derived Echocardiographic Parameters

The following BSA-indexed variables will be derived using the baseline BSA defined in Section 5.7.2:

- LVEDV-I (mL/m^2) = LVEDV/BSA
- LVESV-I (mL/m^2) = LVESV/BSA
- LVSVI (mL/m^2) = LVSV/BSA
- LAV-I (mL/m^2) = LAV/BSA
- LVCO-I (mL/min/m^2) = LVCO/BSA
- LVmass-I(g/m^2) = LVmas/BSA

For the presentation of results, the BSA-indexed variables will be rounded to the same number of decimal places as the corresponding non-indexed variables provided by the echocardiography core laboratory.

5.8. Handling of Missing Data

5.8.1. Missing Efficacy Endpoints

For the primary endpoint, missing data will be imputed under missing at random (MAR) assumption and all observed and imputed missing pVO_2 assessments will be included in the primary analysis of the primary endpoint. Missing secondary CPET endpoints will be handled the same as for the primary endpoint. Missing response for patient reported outcomes will be handled as described in Section 11.1.

5.8.2. Missing Start and Stop Dates for Prior and Concomitant Medication

To classify medications as baseline use or concomitant, missing start and stop dates of medications will be imputed as follows:

- If the medication start date day is missing, it will be imputed with the first of the month,
- If the medication start date day and month are missing, they will be imputed with 01 January,
- If the medication stop date day is missing, it will be imputed with the last day of the month or the date of the last contact with the patient, with the imputed date doesn't exceed the date of last contact,
- If the medication stop date day and month are missing, they will be imputed with 31 December or the date of the last contact with the patient, with the imputed date doesn't exceed the date of last contact,
- For the ongoing medications, the stop date will not be imputed.

5.8.3. Missing Start and Stop Dates for Adverse Events

For AEs with incomplete date information recorded in the eCRF, the imputation will follow the following algorithm:

For missing AE onset Date

- If an AE onset Day is missing and the Month of AE onset is known, then the first day of the month of AE onset will be imputed as the AE onset date. If the month and year of AE onset are the same as month and year of the first dosing, the missing day will be imputed as the first dosing date.
- If AE onset information is not available, then the first dosing date will be imputed as the AE onset date.
- If AE onset day and month are both missing, the missing month and day will be imputed as 01 January. If the year of AE onset is the same as the first dosing date, the AE onset will be imputed as the first dosing date.

For missing AE end Date:

- If the AE end Day is missing and it will be imputed with the last day of the month or the date of the last contact with the patient, with the imputed date doesn't exceed the date of last contact.
- If the AE end date day and month are missing, they will be imputed with 31 December or the date of the last contact with the patient, with the imputed date doesn't exceed the date of last contact.
- For the ongoing AEs, the stop date will not be imputed.

6. STUDY POPULATION

6.1. Subjects Disposition

Patient disposition will be summarized based on all randomized patients. The following will be summarized:

- The number and percentage of patients who completed the study and the number of patients who discontinued from the study early,
- For the patients who discontinued from the study early, reasons for early discontinuation,
- The number and percentage of patients who received at least one dose of the IP,
- For the patients who received at least one dose of the IP, the number and percentage of patients who completed study treatment and the number of patients who discontinued the study treatment early,
- For the patients who discontinued the study treatment early, reasons for early discontinuation.

The number and percentage of randomized patients included in each analysis set will be summarized. Reasons for exclusion from analysis sets will be listed.

6.2. Screen Failures

Screen failures will be listed and summarized by reasons of screening failure.

6.3. Protocol Deviations

Major protocol deviations are reviewed and confirmed by the protocol deviation classification committee (PDCC) during the protocol deviation reviews throughout the study prior to database lock. Major protocol deviations are a subset of protocol deviations that may significantly impact the completeness, accuracy, and/or reliability of the study data or that may significantly affect a subject's rights, safety, or well-being. E.g., of major protocol deviations are

- patients who entered the study even though they did not satisfy the entry criteria.
- patients who developed withdrawal criteria during the study but were not withdrawn.
- patients who received the wrong treatment or incorrect dose.
- patients who received an excluded concomitant treatment.

Number of patients with reported major protocol deviation will be summarized by treatment group for all randomized subjects. A summary of protocol deviations due to COVID-19 will be provided separately.

6.4. Demographic and Baseline Characteristics

Demographic and baseline characteristics, including age, age group [< 65, ≥ 65], sex, race, ethnicity, height, weight, BMI, BSA, randomization stratification variables and baseline disease characteristics will be summarized by randomized treatment group for the FAS using descriptive

statistics. Summary may be repeated for all randomized patients. All randomized patients will be included in the listing of demographic and baseline characteristics.

6.5. Listing of Subject Inclusion and Exclusion Criteria

A listing of randomized patients who did not meet the inclusion and exclusion criteria will be provided.

6.6. Medical History

Medical history will be summarized by treatment received for the Full Analysis Set. HCM-related medical history will be summarized, including time since initial diagnosis and number and percentage of patients meeting the oHCM criteria.

Select cardiovascular medical history and other medical history will be summarized by the Medical Dictionary for Regulatory Activities Terminology (MedDRA) system organ class (SOC) and preferred term (PT).

6.7. Baseline Medications Use

Medications will be coded using World Health Organization (WHO) Drug Dictionary. Baseline medication use is defined as medications that start before the first dose of IP and ends after the first dose of IP or ongoing. The count and percentage of patients with each medication history item will be presented by therapeutic class (Anatomical Therapeutic Chemical [ATC] Class 3) and preferred name. If ATC Class 3 is not available, ATC Class 2 will be used in the summary.

7. EFFICACY

7.1. General Considerations

Efficacy analyses will be performed in the FAS by the randomized treatment group. Unless otherwise specified, all hypothesis tests will be reported as 2-sided p-values. Exploratory endpoints and subgroup analyses will be assessed using a nominal alpha level of 0.05 and will not have multiplicity adjustments. In order to preserve an overall type I error rate for the primary and secondary endpoints testing of the primary and secondary endpoints will follow the testing procedures specified in Section 7.6.

7.2. Testing Statistical Assumptions Including Comparability at Baseline

The primary endpoint will be analyzed using an analysis of covariance (ANCOVA) model. Model assumption will be assessed by graphical examination of residuals. If assumptions are substantially violated, rank based analysis will be performed as supportive analysis. See Section 7.7.4 for more details.

7.3. Statement of the Null and Alternate Hypotheses

The null hypothesis for the primary endpoint is that the treatment difference (aficamten – placebo) of mean change from baseline of pVO_2 at Week 24 is 0 and the alternative hypothesis is that the treatment difference is > 0 (favors aficamten). The tests will be reported with two-sided p-values, but only the direction favoring aficamten direction will be considered success.

7.4. Planned Covariates

Baseline covariates include but are not limited to the stratification factors and baseline measurements. For CPET related endpoints, covariates age, sex, baseline weight will also be evaluated. See Section 7.7.4 for details evaluating covariates effect in the primary endpoint.

7.5. Subgroup Analyses

Subgroup analyses with relatively moderate sample size will be performed to examine the consistency of the observed treatment effect and to gain insight into the effectiveness of aficamten in subpopulations. Analyses of the primary endpoint will be conducted for the following subgroups:

- Sex (male, female)
- Age group (< 65, ≥ 65 years old)
- Baseline body mass index (<30 vs. ≥ 30)
- Baseline NYHA Class (II, III)
- Baseline KCCQ CSS (≤ median and > median)
- Baseline LVEF (≤ median and > median)
- Baseline N-terminal prohormone brain natriuretic (NT-proBNP) (≤ median and > median)

- CPET modality (treadmill, bicycle)
- Baseline pVO_2 (≤ median, > median)
- Beta Blocker (use, no use)
- Baseline resting LVOT (≤ median and > median)
- Sarcomeric gene mutation status (pathogenic or variant of uncertain significance, and non-disease causing or none)

Subgroup analysis will be performed by including the subgroup effect and subgroup by treatment interaction to the model. The subgroup analysis will be performed based on the imputed dataset generated for the primary analysis of the endpoint if the primary analysis is based on multiple imputation.

In addition, subgroup of IND site status (IND sites vs. non-IND sites) analysis will be performed for the primary estimand, proportion of patients with >1 NYHA functional class improvement, KCCQ CSS change from baseline, post-Valsalva LVOT-G change from baseline at Week 24 and overall adverse events summary to demonstrate compliance with relevant aspects of 21 CFR 312.120.

Analyses on the primary and secondary endpoints in patients who were SRT eligible at baseline may be explored.

7.6. Multiple Comparisons and Multiplicity

The null hypothesis for the primary and secondary efficacy variables in the FAS will be tested in the pre-specified order using a closed testing procedure.

For preservation of the overall type I error rate at two-sided 0.05 for the primary and secondary endpoints, the closed testing procedure will be used to address multiplicity. The primary endpoint is tested first at two-sided 0.05. If the primary endpoint achieves statistical significance at two-sided alpha level of 0.05, then the secondary endpoints will be tested at two-sided 0.05, with their testing being in the sequential order of KCCQ-CSS change from baseline, proportion of patients with ≥1 NYHA functional class improvement, post-Valsalva LVOT-G change from baseline, and proportion of patients with post-Valsalva LVOT-G < 30 mmHg in FAS at Week 24; duration of SRT eligibility up to 24 weeks of treatment for those who were SRT eligible at baseline; KCCQ-CSS change from baseline, proportion of patients with ≥1 NYHA functional class improvement, post-Valsalva LVOT-G change from baseline, and proportion of patients with post-Valsalva LVOT-G < 30 mmHg in FAS at Week 12; and change from baseline to Week 24 in total workload. The testing steps are described as follows in Table 4:

Table 4: Testing Steps

Step1	The null hypothesis for the primary endpoint is that there is no treatment difference in the change from baseline to Week 24 in pVO_2 between patients randomized to placebo and those randomized to aficamten in the FAS. The hypothesis will be tested at the two-sided significance level of 0.05. If this hypothesis is rejected, testing will proceed to Step 2; otherwise testing will stop.
Step 2	The null hypothesis for the first secondary endpoint is that there is no treatment difference in the change from baseline to Week 24 in KCCQ-CSS in the FAS. The hypotheses will be tested at the two-sided significance level of 0.05. If this hypothesis is rejected, testing will proceed to Step 3; otherwise testing will stop.
Step 3	The null hypothesis for the second secondary endpoint is that there is no treatment difference in proportion of patients with ≥1 NYHA functional class improvement at Week 24 in the FAS. The hypotheses will be tested at the two-sided significance level of 0.05. If this hypothesis is rejected, testing will proceed to Step 4; otherwise testing will stop.
Step 4	The null hypothesis for the third secondary endpoint is that there is no treatment difference in change from baseline to Week 24 in post-Valsalva LVOT-G in the FAS. The hypotheses will be tested at the two-sided significance level of 0.05. If this hypothesis is rejected, testing will proceed to Step 5; otherwise testing will stop.
Step 5	The null hypothesis for the fourth secondary endpoint is that there is no treatment difference in the proportion of patients with post-Valsalva LVOT-G < 30 mmHg at Week 24 in the FAS. The hypotheses will be tested at the two-sided significance level of 0.05. If this hypothesis is rejected, testing will proceed to Step 6; otherwise testing will stop.
Step 6	The null hypothesis for the fifth secondary endpoint is that there is no treatment difference in duration for SRT eligible during the 24-Week treatment period in patients who were SRT eligible at baseline in the FAS. The hypothesis will be tested at the two-sided significance level of 0.05. If this hypothesis is rejected, testing will proceed to Step 7; otherwise testing will stop.
Step 7	The null hypothesis for the sixth secondary endpoint is that there is no treatment difference in the change from baseline to Week 12 in KCCQ-CSS in the FAS. The hypothesis will be tested at the two-sided significance level of 0.05. If this hypothesis is rejected, testing will proceed to Step 8; otherwise testing will stop.
Step 8	The null hypothesis for the seventh secondary endpoint is that there is no treatment difference in proportion of patients with ≥1 NYHA functional class improvement at Week 12 in the FAS. The hypotheses will be tested at the two-sided significance level of 0.05. If this hypothesis is rejected, testing will proceed to Step 9; otherwise testing will stop.
Step 9	The null hypothesis for the eighth secondary endpoint is that there is no treatment difference in change from baseline to Week 12 in post-Valsalva LVOT-G in the FAS. The hypotheses will be tested at the two-sided significance level of 0.05. If this hypothesis is rejected, testing will proceed to Step 10; otherwise testing will stop.

Step 10	The null hypothesis for the ninth secondary endpoint is that there is no treatment difference in the proportion of patients with post-Valsalva LVOT-G < 30 mmHg at Week 12 in the FAS. The hypotheses will be tested at the two-sided significance level of 0.05. If this hypothesis is rejected, testing will proceed to Step 11; otherwise testing will stop.
Step 11	The null hypothesis for the tenth secondary endpoint is that there is no treatment difference in change from baseline to Week 24 in total workload in the FAS. The hypotheses will be tested at the two-sided significance level of 0.05.

7.7. Analysis of the Primary Efficacy Endpoint

7.7.1. Primary Efficacy Analysis

The primary endpoint is change in pVO_2 from baseline to Week 24. The **primary analysis** will be performed using an ANCOVA model that includes terms of treatment, randomization stratification factors (beta-blocker use status and CPET modality), baseline pVO_2 and baseline body weight as covariates in the FAS. Table 5 below displays details of the two estimands for the primary endpoint.

Table 5: Estimands for Primary Endpoint

Attributes	Primary Estimand	Secondary Estimand
Population	FAS, target population of potentially treatable aficamten subjects.	Hypothetical target population of potentially treatable aficamten subjects continue with treatment and are capable of completing the Week 24 assessment. Subjects with missing Week 24 pVO_2 due to intercurrent events or discontinuing treatment prior to Week 24 will be excluded.
Variable	Change from baseline to Week 24 in pVO_2. Data to be analyzed include all observed Week 24 pVO_2 values from subjects who complete 24 weeks of treatment, or from subjects who early terminate from the treatment but remain in the study and have Week 24 pVO_2 and imputed pVO_2 for subjects who don't have Week 24 pVO_2. Imputation details are provided below.	Change from baseline to Week 24 in pVO_2. Data to be analyzed include observed pVO_2 values from subjects who complete at least 24 weeks of treatment.
Measure of intervention effect	Mean treatment difference regardless of completing 24 weeks of treatment and experiencing intercurrent events.	Mean treatment difference among all subjects who remained on their randomized treatment for 24 weeks.

Subjects will be followed per the schedule of assessments from randomization through their final visit irrespective of whether the subject is continuing to receive study treatment. Reasons for not completing Week 24 CPET will be recorded on eCRF; categories of reasons include adverse

events, early termination, equipment failure, investigator decision, subject decision and other. The percentage of missing CPET data at Week 24 and the reasons for the missing data will be tabulated in the FAS. The following type of intercurrent events could preclude CPET at Week 24.

- Death
- Hospitalization
- CV AEs
- non-CV AEs (e.g., orthopedic injury)
- COVID-19 related intercurrent events e.g., subjects' decision to early terminate from the study due to the COVID-19 precautions, site closures, hospitalization due to COVID-19, or COVID-19 symptoms preventing subjects from coming to the Week 24 visit.

CPET data deemed to be invalid by the CPET core lab will be treated as missing CPET data in the analysis. CPET core lab flags the CPET results as invalid when there are:

- Technical equipment failure during CPET (i.e., air leak/lack of proper equipment utilization such as missing nose clip leading to lack of capture of gas exchange data)
- Transient non-cardiac issues that precluded conduct of the exercise study (as defined by inability to turn the pedals during the warm-up period for at least 3 min)
- CPET MOP-major CPET process deviation can impact one or more CPET variables.

Missing data as a result of an invalid assessment by the CPET core lab cab will be considered as missing at random.

Missing pVO2 at Week 24 regardless of type of intercurrent events will be imputed using multiple imputation methodology under the MAR assumption for the primary analysis of the primary estimand. Missing Week 24 CPET is expected to be low. Patients will be followed according to the schedule of activities in the protocol from randomization through the date of final visit irrespective of whether the patient is continuing to receive IP unless the patient has discontinued prematurely from the study or withdrawn consent. The protocol allows up to 4 weeks extension of Week 24 in the event that the subject is temporarily unable to exercise due to an AE e.g., ankle sprain, upper respiratory infection etc. or due to equipment malfunction to ensure post randomization CPET data collection. Missing Week 24 CPET due to intercurrent events i.e., non-CV AEs or COVID-19 related intercurrent events can be considered as MAR. Death event is expected to be very rare given the patient population and the duration of the treatment in this study. Assume patients' risk of death event or hospitalization due to HCM symptom and other CV AEs is balanced between treatment groups, missing data due to this type of intercurrent events for the primary estimand will be imputed as MAR.

The imputation model will use regression multiple imputation which includes treatment group, randomization stratification factors, baseline pVO_2, sex, age, baseline hemoglobin, baseline body weight, baseline KCCQ CSS, and baseline NYHA class and the last available post randomization NYHA functional class, resting and Valsalva LVOT. Categorical variables, i.e., treatment group, baseline NYHA functional class, and sex will be specified in the CLASS statement. One hundred

(100) imputed datasets will be generated. Change from baseline in pVO_2 will be calculated based on the observed and imputed data. Each of the imputed dataset will be analyzed using the primary analysis ANCOVA model. LSM estimate of treatment difference and the standard error will be combined using Rubin's rules (Rubin 1987) to produce a LSM estimate of the treatment difference, its 95% confidence interval, and p-value for the test of null hypothesis of no treatment effect. LSM, LSM difference and the corresponding standard error, 95% CI and p-value will be presented.

7.7.2. Sensitivity Analyses of the Primary Efficacy Endpoint

To evaluate the robustness of the primary analysis approach, sensitivity analyses, e.g., placebo-based imputation and tipping point analysis will be performed. In placebo-based imputation, missing pVO2 from subjects who discontinued from aficamten treatment or missing pVO2 from subjects from the placebo arm will be imputed based on the model that is constructed using observed pVO2 data from the placebo arm. Missing pVO2 from subjects who remained on aficamten treatment will be imputed based on the model that is constructed using observed pVO2 data from aficamten arm. Tipping point analysis will be performed by applying a range of negative shift to adjust the imputed value of missing pVO2 in aficamten group. If there are 10% or more missing data and/or 5% or more subjects missing data due to reason related to IP, the primary analysis will have the missing data imputed using placebo-based imputation.

The tipping point can be identified while the result is no longer statistically significant. Clinical judgment will be applied to evaluate the plausibility of the assumptions underlying this tipping point.

In addition, sensitivity analysis to evaluate COVID-19 impact will be performed by repeating the primary analysis after setting the Week 24 CPET to missing from subjects who are impacted by COVID pandemic.

Another sensitivity analysis is to fit a repeated measures mixed model to pVO_2 baseline and Week 24 data. The model includes stratification factors, visit, stratification by visit, and a numeric covariate which equals 0 for both treatment groups at baseline and equals 0 for placebo at Week 24 and equals 1 for aficamten group at Week 24. The primary treatment comparison is for the numeric covariate for treatments which corresponds to the treatment difference at week 24 in a specification for which there is no treatment difference at baseline.

7.7.3. Subgroup Analyses for the Primary Endpoint

Subgroup analyses will be performed by including the subgroup effect and subgroup by treatment interaction terms to the primary ANCOVA model for the primary endpoint. For subgroup analysis for the primary estimand, missing data imputed in the primary analysis will be used in the subgroup analyses. Only summary statistics will be presented for the subgroup level when the number of subjects in either treatment arm is ≤ 15 at this level. LSM estimate of the treatment difference, 95% confidence intervals for the mean treatment difference and nominal p-values will be provided for each subgroup level.

7.7.4. Supportive Analyses for the Primary Endpoint

To explain heterogeneity or identify treatment effect modifiers from the baseline characteristics, covariates used to define pre-specified subgroups and these covariates by treatment interaction

terms will be included in the ANCOVA model as supportive analysis. Global test of covariates by treatment interactions will be performed.

Stepwise model selection method will be used based on the stay or entry level of 0.05 to evaluate significant baseline covariates impact on the primary endpoint. ANCOVA model will be repeated by adjusting for the significant baseline covariates. Covariates measured as continuous will be introduced to the model as continuous variable. These analyses will be based on observed data.

The normality assumptions of the ANCOVA model will be investigated graphically. The scaled residuals will be examined. A supportive analysis will be performed after transforming pVO_2 data into ranks if greater than 5% of patients have an extreme change from baseline value at Week 24. Extreme values in the pooled data are defined as observations outside of Tukey's outer fences, i.e. observations that are less than the 25% quartile – 3 times the inter quartile range or greater than 75% quartile + 3 times the inter quartile range. Ranks will be applied to all changes from baseline data after the imputation step. Baseline data will be ranked separately.

The secondary estimand will be analyzed using the same primary analysis ANCOVA model for the primary estimand. Subgroup analysis for the secondary estimand will be performed using the same ANCOVA model for the subgroup analysis for the primary estimand.

7.8. Analysis of the Secondary Efficacy Endpoints

The secondary endpoint(s) of the trial are:

- Change in KCCQ-CSS from baseline to Week 12 and Week 24
- Proportion of patients with ≥1 class improvement in NYHA Functional Class from baseline to Week 12 and Week 24
- Change in post-Valsalva LVOT-G from baseline to Week 12 and Week 24
- Proportion of patients with post-Valsalva LVOT-G <30 mmHg at Weeks 12 and 24
- Total duration of SRT eligibility during the 24-Week treatment period in patients who were SRT eligible at baseline
- Change in total workload during CPET from baseline to Week 24

7.8.1. Analysis of the Secondary Efficacy Endpoints

1. Change in KCCQ-CSS from baseline to Week 12 and Week 24

The primary analysis for change in KCCQ-CSS from baseline to Week 12 and 24 will be performed using a MMRM model with baseline as covariate, randomization stratification factors, visit, treatment group, and interaction terms of treatment by visit and baseline by visit. An unstructured covariance matrix will be specified. All data observed up to Week 24 post randomization will be included in the model. Estimates for endpoints at Week 12 and Week 24 will be obtained from the LS Means estimate at visit of Week 12 and 24 from the model.

If there are >10% difference in baseline KCCQ CSS across regions, treatment by visit and region, baseline by visit and region will be included in the model. Treatment effect at each visit

week will be estimated from the interaction terms of treatment by visit by region, with coefficients for each region determined by proportion of patients evaluated in each region.

Sensitivity analysis based on multiple imputation with one hundred invocations will be performed. First the intermittent missing data will be imputed using the Markov Chain Monte Carlo (MCMC) method under MAR assumption. The imputation will be performed separately for each randomized treatment group and will include the following terms in the imputation model: region (for KCCQ only), endpoint observations from baseline up to Week 24. The monotone missing data will be imputed using the imputation model built from placebo group. 100 complete data sets will be generated and analyzed using the same model for the primary analysis of the change from baseline endpoint. The results from the 100 complete data sets will be combined using Rubin's combination rule for the inference.

Subgroup analysis will be performed using the primary analysis model MMRM model including additional effects of subgroup, subgroup by treatment and subgroup by treatment by visit interaction the model.

2. Change from baseline in post-Valsalva LVOT-G from baseline to Week 12 and Week 24

The analysis of this endpoint will follow the same specified above for change in KCCQ-CSS change from baseline to Week 12 and 24.

3. Proportion of patients with ≥1 class improvement in NYHA Functional Class at Weeks 12 and Week 24

For the proportion of patients with ≥1 class improvement in NYHA class at Week 12, Week 16 NYHA class will be used if Week 12 NYHA class is not available. Week 8 NYHA class will be used if Week 16 NYHA is not available. Patient will be considered as not achieving ≥1 improvement in NYHA class at Week 12 if no NYHA at Weeks 8, 12 and 16 is available. Similarly, Week 20 NYHA class will be used if Week 24 NYHA is not available. Patient will be considered as not achieving ≥1 improvement in NYHA class at Week 24 if Week 20 and Week 24 NYHA are not available. A sensitivity analysis will be performed by repeating Cochran–Mantel–Haenszel (CMH) test by assigning missing NYHA class at Weeks 12 and 24 as non-responders. Proportion of patients with ≥1 class improvement in NYHA class will be analyzed using CMH test stratified by randomization factors. The p-value and 95% CI will be obtained using exact method.

Subgroup analysis will be done by repeating the CMH test for each subgroup level without specifying stratifying by randomization stratification factors.

4. Proportion of patients with post-Valsalva LVOT-G <30 mmHg at Weeks 12 and 24

For the proportion of patients with post-Valsalva LVOT < 30 mmHg at Week 12, Week 16 post-Valsalva LVOT will be used if Week 12 visit is performed but post Valsalva LVOT is not available. Week 8 post-Valsalva LVOT will be used if Week 16 is not available. Similarly, Week 20 post Valsalva LVOT will be used if Week 24 is performed but post Valsalva LVOT is not available.

Proportion of patients with post-Valsalva LVOT-G <30 mmHg at Weeks 12 and 24 will be analyzed using Cochran– Mantel–Haenszel (CMH) test stratified by randomization factors. The p-value and 95% CI will be obtained using exact method.

Subgroup analysis will be done by repeating the CMH test for each subgroup level without specifying stratifying by randomization stratification factors.

5. Total duration of SRT eligibility during the 24-Week treatment period in patients who were SRT eligible at baseline

Patient SRT eligibility will be assigned after data handling in case there are missing NYHA class or LVOT assessments. Intermittent missing NYHA or LVOT will be imputed follows the same imputing method for missing Week 12 or Week 24; e.g., impute Week 16 NYHA if Week 20 NYHA is available or use Week 12 NYHA if Week 20 NYHA is not available. Patient will be treated as SRT eligible if SRT eligibility can't be determined due to the missing NYHA class or LVOT assessments or clinical visits not performed after patient early terminates from the study.

Total duration will be calculated as the number of days patients are SRT eligible, from the date of the randomization until Week 24. Total duration will be 24 weeks if a patient remains SRT eligible at all visits from baseline until Week 24 (regardless of whether a patient is SRT eligible at Week 24 or not). Because the SRT eligibility status of a patient may change during the study conduct, only intervals during which the patient is SRT eligible, will be summed. The start of the interval is the time of randomization (or resumption of SRT eligible status), and the end of the interval is the date prior to the visit when the patient becomes SRT not eligible.

SRT (Y/N) / Case #	Week 2	Week 4	Week 6	Week 8	Week 12	Week 16	Week 20	Week 24	Duration SRT Eligible (Weeks)
1	N	Y	Y	Y	N	N	Y	Y	12
2	Y	Y	N	N	Y	N	N	Y	10
3	Y	Y	N	N	N	N	N	N	6
4	Y	Y	N	N	Y	N	Y	N	14
5	Y	Y	N	N	Y	N	Y	Y	14

Total duration of SRT eligibility during the 24 Week of treatment period will be analyzed using an ANCOVA model includes treatment and randomization stratification factor beta blocker use/non-use as fixed effects and significant baseline characteristics as covariates. Stepwise model selection method will be used based on the default stay or entry level of 0.05 to evaluate significant baseline covariates. Sensitivity analysis of the SRT eligible endpoints includes repeating the same ANCOVA analysis the area under the curve calculated using the numeric value assigned to the SRT eligibility at each visit and treatment duration until Week 24, where SRT eligibility is assigned to value of 1 and SRT not eligible is assigned to value of 0.

6. Change in total workload during CPET from baseline to Week 24

The primary and sensitivity analysis for the total workload will follow the same analysis approach for the primary estimand of the primary endpoint.

Subgroup analysis will be performed similarly as specified for the primary endpoint.

Table 6 summarizes the primary and secondary efficacy endpoints and planned analysis method.

Table 6: Endpoint Summary Table

Endpoint	Primary Analysis Method	Sensitivity/Subgroup Analysis	COVID-19 Related Analyses
Primary Endpoint: Change in pVO2 on CPET from baseline to Week 24			
Primary estimand[1]	Missing data will be imputed using multiple imputation method (Section 7.7.1). Complete dataset will be analyzed using an ANCOVA model with fixed effects of treatment, randomization stratification factors baseline pVO_2 value and baseline body weight	Sensitivity analysis<br>• ANCOVA model will be repeated with missing data from subjects who discontinued aficamten treatment as if the aficamten subjects were in the placebo arm<br>• Tipping point analysis<br>Supportive analysis<br>• Mixed model with numeric covariate as 0 for baseline and placebo group at Week 24 and 1 for aficamten group at Week 24, visit, stratification factors and stratification factors by visit as fixed term. unscheduled covariance structure will be specified.<br>• Multivariate ANCOVA model to evaluate treatment by covariates interaction; ANCOVA model with significant covariates per model selection<br>Subgroup analyses for variables in Section 7.5	• Repeat the primary analysis after setting Week 24 pVO_2 to missing from subjects who were impacted by COVID pandemic<br>• Summarize the number of subjects infected (positive COVID-19 test with or without symptoms) prior to Week 24 CPET.
Secondary estimand	ANCOVA model with fixed effects of treatment, randomization stratification factors baseline pVO2 value and baseline body weight.	Subgroup analyses for variables in Section 7.5	

Table 6: Endpoint Summary Table (Continued)

Endpoint	Primary Analysis Method	Sensitivity/Subgroup Analysis	COVID-19 Related Analyses
Secondary Endpoints			
Change in KCCQ-CSS from baseline to Week 12 and Week 24	MMRM model with baseline as covariate, randomization stratification factors, visit, treatment group, and interaction terms of treatment by visit and baseline by visit. An unstructured covariance matrix will be specified.	Sensitivity analysis:<br>• Intermittent missing data be imputed using multiple imputation MCMC first and the monotone missing values will be imputed using the imputation model built from the placebo group. Complete data will be analyzed using ANCOVA model for each week separately.<br>Subgroup analyses for variables in Section 7.5	
Change in post-Valsalva LVOT-G from baseline to Week 12 and Week 24	MMRM model with baseline as covariate, randomization stratification factors, visit, treatment group, and interaction terms of treatment by visit and baseline by visit. An unstructured covariance matrix will be specified.	Sensitivity analysis:<br>• Intermittent missing data be imputed using multiple imputation MCMC first and the monotone missing values will be imputed using the imputation model built from the placebo group. Complete data will be analyzed using ANCOVA model for each week separately,<br>Subgroup analyses for variables in Section 7.5	
Proportion of patients with ≥1 class improvement in NYHA Functional Class from baseline to Week 12 and Week 24	CMH test stratified by randomization factors	Treating patient as non-responder if NYHA is not performed and repeat CMH test.<br>Subgroup analyses for variables in Section 7.5	

Table 6: Endpoint Summary Table (Continued)

Endpoint	Primary Analysis Method	Sensitivity/Subgroup Analysis	COVID-19 Related Analyses
Proportion of patients with post-Valsalva LVOT-G <30 mmHg	CMH test stratified by randomization factors	Subgroup analyses for variables in Section 7.5	
Total duration of SRT eligible during the 24-week of treatment period	Total duration of SRT eligible during the 24-week of treatment period will be analyzed using an ANCOVA model includes treatment and randomization stratification factor beta blocker use/non-use as fixed effects and significant baseline characteristics as covariates. Stepwise model selection method will be used based on the default stay or entry level of 0.05 to evaluate significant baseline covariates.	SRT eligible will be assigned value of 1 and SRT not eligible will be assigned value of 0. Area under the curve using the numeric value assigned and treatment duration until Week 24 will be calculated. AUC will be analyzed using the same ANCOVA model.<br>Same ANCOVA model will be used to analyze the total time for SRT eligibility.	
Change in total workload during CPET from baseline to Week 24	Missing data will be imputed using multiple imputation method (Section 7.7.1). Complete dataset will be analyzed using an ANCOVA model with fixed effects of treatment, randomization stratification factors baseline total workload value and baseline body weight.	Sensitivity analysis<br>• ANCOVA model will be repeated with missing data from subjects who discontinued aficamten treatment as if the aficamten subjects were in the placebo arm.<br>• Tipping point analysis<br>Subgroup analyses for variables in Section 7.5	

[1]See Table 5 for details on the two estimands for the primary endpoint.

7.9. Analysis of the Exploratory Efficacy Endpoints

Exploratory endpoints are specified in Section 2.2.3. Other echocardiography parameters and CPET parameters not listed as secondary endpoints or in Section 2.2.3 may also be analyzed as exploratory endpoints. All 10 summary scores will be derived for KCCQ. Change from baseline in each summary scores (except CSS) to Weeks 12 and 24 will be analyzed as exploratory endpoints. Patients with ≥5, 10 and 20 points improvement in KCCQ summary scores at Weeks 12 and 24 will also be summarized and analyzed. Domain scores and summary score of SAQ-7 will be derived. Health state for EQ-5D-5L and index value using US value set will be calculated. Analysis of change from baseline in index score and VAS score of EQ-5D-5L be performed. Proportional change in Valsalva and resting LVOT-G will be derived and analyzed as exploratory endpoints.

7.9.1. Analysis of the Exploratory Efficacy Endpoint

The same ANCOVA model specified for the primary endpoint will be used to analyze the endpoints from CPET and continuous CMR measurements that are only assessed once post randomization. Body weight will not be included in the model to analyze CMR parameters. The same MMRM model will be used to analyze the continuous endpoints measured at multiple visits post randomization. CMH test stratified by randomization factors will be used analyze endpoints evaluation proportion of responders or binary outcomes.

Proportion of responders at each week will be summarized. Responders that are defined based on NYHA classification or LVOTs will be assigned after data handling in case there are missing NYHA class or LVOT assessments. Missing NYHA classification or LVOT value will be imputed the same way as that for the secondary endpoints that are based on NYHA classification or LVOT value. Patient will be treated as non-responder if patient's response status can't be determined due to the missing NYHA classification or LVOT assessments (after the imputation above) or clinical visits not performed after the patient early terminates from the study. Proportion of responders will be analyzed using Cochran– Mantel–Haenszel (CMH) test stratified by randomization factors. The p-value and 95% CI will be obtained using exact method. Proportion of patients remaining SRT eligible at Week 24 will be analyzed using logistic model regression model stratified by beta blocker use/non-use adjusting for significant baseline characteristics. The model will include treatment as fixed effect and baseline characteristics as covariates. Covariates measured as continuous will be introduced to the model as continuous variable. Stepwise model selection method will be used based on the default stay or entry level of 0.05 to evaluate significant baseline covariates. Supportive analysis using CMH stratified by randomization factor (beta-blocker use/non-use) will be performed. Another supportive analysis of proportion of patients SRT eligible at each visit will be provided using a repeated measure logistic regression model with treatment, visit, treatment by visit and significant baseline characteristics from the model above. Time to first SRT ineligible in patients who are SRT eligible at baseline will be analyzed using Kaplan-Meier method.

For NT-proBNP and hs-cardiac-TnI, the log transformed proportional change will be analyzed using a MMRM model with log baseline as covariate, treatment group, randomization stratification factors, visit, log baseline by visit and treatment by visit interaction as fixed effects. Log transformed proportional change in Valsalva and resting LVOT will be analyzed using the

same model. Geometric LS Means estimate and ratio of proportional change in NT-proBNP and hs-cardiac-TnI between aficamten vs. placebo, 95% CI of ratio and p-value will be presented. Median and median difference of NT-pro-BNP and hs-cardiac- Tnl between treatment group and 95% confidence of the median difference will be presented at Week 12 and 24. Time to 1 mm ST depression at Week 24 will be analyzed using Cox regression model with treatment, randomization stratification factors as fixed effect. Patients didn't experience 1 mm ST depression will be censored at the end of CPET exercise. Logistic regression model will be fit to LVH strain pattern on ECG for Week 12 and 24, separately. The model will include baseline LVH pattern, stratification factors and treatment. Difference in proportion of patients with no LVH will be estimated and 95% CI for odds ratio (aficamten vs. placebo) and its corresponding p value will be obtained.

8. SAFETY AND TOLERABILITY

Safety and tolerability analyses will be based on the Safety Analysis Set. Safety data will be analyzed descriptively and tabulated by treatment groups.

8.1. Overall Summary of Tolerability

Overall summary of tolerability will include the following:

- Number of patients treated
- Number of patients with TEAEs
- Number of patients with treatment-emergent serious adverse events (TESAEs)
- Number of patients with TEAEs leading to premature treatment discontinuation
- Number of patients with at least one TEAE related to the study drug
- Patients with at least one moderate or severe TEAE
- Patients with at least one severe TEAE
- Number of Deaths

Summary of number and percent of patients with each AE category will be provided by treatment group and dose level at AE onset. Summaries of the number of events will also be provided.

8.2. Adverse Event Preferred Term and Body/Organ System Summary Tables

8.2.1. Summaries of Adverse Event Incidence Rates for All Subjects

All AE terms will be coded using MedDRA. TEAEs and TESAEs will be summarized by primary SOC and PT, and also by severity (mild, moderate and severe) and relationship to study drug (related and not related). For a TEAE reported more than once from a patient, the TEAE will be counted only once in the SOC or PT category using the most severe occurrence or closer relationship to the study drug. All AEs will be listed.

The following subsets of TEAEs will be summarized by SOC and PT:

- All TEAEs
- TEAEs related to study drug
- TEAEs leading to early discontinuation of study drug
- TESAEs

A summary of all TEAEs by PT will be provided. AE summaries will be sorted by descending order of SOC in aficamten group and descending order of preferred term within the SOC. TEAE and TESAE summary of ≥5% and ≥2%, respectively will be provided based on incidence rate in either aficamten or placebo group. Summary of TEAEs by maximum severity will display number and percentage of AEs with maximum severity being mild, moderate, or severe within

each SOC and PT. All TEAE summary will also be provided by dose level. Summary of number of events will be provided for all TEAE summary.

8.2.2. Summaries of Adverse Events of Special Interest

The following events are considered adverse events of special interest:

- Incidence of reported major adverse cardiac events (CV death, cardiac arrest, non-fatal stroke, non-fatal myocardial infarction, CV hospitalization)
- Incidence of new onset persistent atrial fibrillation
- Incidence of ventricular arrhythmias requiring treatment

Summary of patients counts and percentage by each event type will be provided by treatment group.

In addition, incidence of appropriate ICD discharges will be summarized for baseline and also post randomization. Incidence of aborted sudden cardiac death will be provided. Number of patients and number of incidences of LVEF <40% and 50% will be provided by treatment group. Number of patients and number of incidences of LVEF <50% and with signs and symptoms of heart failure (concomitant adverse event of heart failure or dyspnea) or experienced ≥30% increase in NT-proBNP, relative to results from the most recent previous visit and above the upper limit of normal, at the time of LVEF assessment will be provided by treatment group. Signs and symptoms and NT-proBNP increase referring to AEs with onset date or NT-proBNP assessment date within ± 7 days relative to the date when LVEF <50%.

8.3. Total Duration of Therapy, Final Daily Dose of Study Medication, and Compliance

8.3.1. Summary of IP Exposure and Overall Compliance

Total duration of treatment and total exposure of study drug will be summarized. IP compliance will be derived as:

IP compliance =100% * (number of tablets dispensed – number of tablets returned)/expected number of tablets administered.

Number of tablets dispensed and returned will be collected on the study drug accountability eCRF. For the IP kits not returned, the number of tablets returned will be set to 0 in this derivation, assuming all tablets were taken. Expected number of tablets administered will be derived as the number of daily tablets times the days in an IP dosing period, summed over all dosing periods. Days of dosing interruption will be excluded from the expected number of tablets calculation.

8.3.2. Summary of Dose Titration

IWRS-guided dose titration will be summarized showing the number and percentage of patients at each dose level by visit. Number and percentage of patients by last titrated dose will be provided.

8.4. Concomitant and Other Medications

Concomitant medications reported on the eCRF will be summarized. Medications with a start date that is 28 days after the last dose of the study drug will be excluded from the summary. The WHO Drug Dictionary will be used to classify medications by therapeutic class (ATC Class 3) and preferred name. If ATC Class 3 is not available, ATC Class 2 will be used in the summary. Coding will be performed using WHO Drug Dictionary.

8.5. Routine Laboratory Data

Clinical chemistry, hematology and urinalysis laboratory measurements and value changes from baseline at each laboratory blood sample collection time point will be summarized. Values below or above the quantifiable limits will be treated as equal to the limits in the summary. The count and percentage of patients who had normal or missing laboratory values at baseline and abnormal laboratory values post baseline will be presented. The lower limit of normal (LLN) and upper limit of normal (ULN) provided by the laboratories will be used as the criteria to determine abnormality. For each parameter, the denominator of the percentage will include patients with normal or missing assessments at baseline, and with at least one assessment post baseline. The numerator of the percentage will include patients who had at least one abnormal assessment post baseline among the patients that were counted in the denominator. Assessment collected at unscheduled visits, or the Follow-up Visit will be included in the summary.

Shift of clinical laboratory results from baseline severity to the maximum post baseline severity will be presented for selected laboratory parameters.

Liver function test results will be summarized as count and percentage of patients with normal baseline and abnormal post-baseline values in Alanine Aminotransferase (ALT), Aspartate Aminotransferase (AST), Alkaline Phosphatase (ALP) and bilirubin, with the following categories:

- ALT > 3xULN, > 5xULN, > 8xULN
- AST > 3xULN, > 5xULN, > 8xULN
- ALT and/or AST > 3xULN, > 5xULN, > 8xULN
- ALT and/or AST > 3xULN and total bilirubin > 2xULN and ALP < 2xULN
- ALT and/or AST > 3xULN and total bilirubin > 2xULN
- Bilirubin (total, indirect or direct) > 2xULN, >3xULN
- ALT or AST > 3 x ULN with symptoms including nausea, vomiting, anorexia, abdominal pain, fatigue, rash, dark-colored urine, light-colored bowel movements, jaundice, or fever

8.6. Vital Signs

Vital signs and changes from baseline will be summarized descriptively by treatment group over time. The changes from baseline at each post-baseline on-treatment visit will be additionally summarized by dose level at visit.

Patients will also be categorized into the following groups for each of the vital sign parameters if a post baseline value falls into a specific group. Unscheduled assessments will be included in the determination. The number of subjects in each group will be summarized for each dosing group.

Diastolic Blood Pressure

- ≤ 50 mmHg
- ≥ 100 mmHg

Systolic Blood Pressure

- ≤ 80 mmHg
- ≥ 160 mmHg

Heart Rate

- ≤ 50 beats/min
- ≥ 120 beats/min

Respiratory Rate

- > 18 breaths/min

8.7. Electrocardiogram

The baseline ECG is defined as the mean of all pre-dose assessments. PR, RR, QRS, QT, and Fridericia corrected QT (QTcF) intervals and their change from baseline will be summarized by treatment group and scheduled assessment. patients will be categorized into the following groups per their maximum change from baseline in QTcF. Unscheduled assessments will be included in the determination of the maximum change. The number and percentage of subjects in each group will be summarized.

- ≤30 msec
- >30 – 60 msec
- >60 msec

Patients will also be categorized into the following groups per their maximum post baseline QTcF. Unscheduled assessments will be included in the determination of the maximum post baseline value. The number of subjects in each group will be summarized for each dosing group.

- ≤450 msec
- >450 – 480 msec
- >480 – 500 msec
- >500 msec

ECG morphology analyses will be performed. New onset findings will be presented as the percentage of subjects with ‘new’ finding (ECG finding that were not present at any baseline ECG and became present on at least 1 ECG during the treatment) for the following variables

2:1 AV Block, AV Mobitz I,II, Complete heart block, first degree AV block, left atrial abnormality, left ventricular hypertrophy, right ventricular hypertrophy, incomplete left bundle branch block, incomplete right bundle branch block, intraventricular conduction defect, left anterior hemiblock, left bundle branch block, left posterior hemiblock, right bundle branch block, Wolff-Parkinson-White, Artificial pacemaker, atrial pacing, sinus bradycardia, sinus pauses, sinus tachycardia, atrial fibrillation, atrial flutter, atrial tachycardia, supraventricular tachycardia, prolonged QTC, ST depressed, ST elevated, T wave inverted, non-sustained ventricular tachycardia, ventricular fibrillation.

9. PHARMACOKINETICS

Plasma concentrations of CK-3773274 and its measured metabolites and PK parameters $C_{post\ dose}$ and $C_{pre\text{-}dose}$ will be summarized using descriptive statistics including arithmetic mean, standard deviation, coefficient of variation, geometric mean, geometric coefficient of variation, median, and range. Geometric mean concentrations over time will be graphically displayed.

10. REFERENCES

EuroQol Group. (1990). "Eq-5d-3l." EuroQol Research Foundation, from https://euroqol.org/publications/user-guides, Updated Date Accessed Date.

EuroQol Group. (2009). "Eq-5d-5l." EuroQol Research Foundation, from https://euroqol.org/publications/user-guides, Updated Date Accessed Date.

Pickard, A. S., Law, E. H., Jiang, R., Pullenayegum, E., Shaw, J. W., Xie, F., et al. (2019). "United states valuation of eq-5d-5l health states using an international protocol." Value in Health 22(8): 931-941.

Raghunathan, T., & Dong, Q. (2011). Analysis of variance from multiply imputed data sets. *Ann Arbor: University of Michigan*.

Rubin, D. B. (1987). Multiple imputation for nonresponse in surveys. New York, John Wiley & Sons, Inc.

11. APPENDIX

11.1. Patient-reported Outcome Scoring Algorithm

11.1.1. KCCQ

There are 10 summary scores within the KCCQ, which are calculated as follows:

1. Physical Limitation

- Code responses to each of Questions 1a-f as follows:

  Extremely limited = 1
  Quite a bit limited = 2
  Moderately limited = 3
  Slightly limited = 4
  Not at all limited = 5
  Limited for other reasons or did not do = <missing value>

- If at least three of Questions 1a-f are not missing, then compute
  Physical Limitation Score = 100*[(mean of Questions 1a-f actually answered) - 1]/4
  (see footnote at end of this appendix for explanation of meaning of "actually answered")

2. Symptom Stability

- Code the response to Question 2 as follows:

  Much worse = 1
  Slightly worse = 2
  Not changed = 3
  Slightly better = 4
  Much better = 5
  I've had no symptoms over the last 2 weeks = 3

- If Question 2 is not missing, then compute

  Symptom Stability Score = 100*[(Question 2) – 1]/4

3. Symptom Frequency

- Code responses to Questions 3, 5, 7 and 9 as follows:

  Question 3
  Every morning = 1
  3 or more times a week but not every day = 2
  1-2 times a week = 3
  Less than once a week = 4
  Never over the past 2 weeks = 5

  Questions 5 and 7
  All of the time = 1
  Several times a day = 2
  At least once a day = 3

3 or more times a week but not every day = 4
1-2 times a week = 5
Less than once a week = 6
Never over the past 2 weeks = 7

<u>Question 9</u>
Every night = 1
3 or more times a week but not every day = 2
1-2 times a week = 3
Less than once a week = 4
Never over the past 2 weeks = 5

- If at least two of Questions 3, 5, 7 and 9 are not missing, then compute:

S3 = [(Question 3) – 1]/4
S5 = [(Question 5) – 1]/6
S7 = [(Question 7) – 1]/6
S9 = [(Question 9) – 1]/4
Symptom Frequency Score = 100*(mean of S3, S5, S7 and S9)

4. Symptom Burden

- Code responses to each of Questions 4, 6 and 8 as follows:

Extremely bothersome = 1
Quite a bit bothersome = 2
Moderately bothersome = 3
Slightly bothersome = 4
Not at all bothersome = 5
I've had no swelling/fatigue/shortness of breath = 5

- If at least one of Questions 4, 6 and 8 is not missing, then compute

Symptom Burden Score = 100*[(mean of Questions 4, 6 and 8 actually answered) – 1]/4

5. Total Symptom Score

= mean of the following available summary scores:
Symptom Frequency Score
Symptom Burden Score

6. Self-efficacy

- Code responses to Questions 10 and 11 as follows:

<u>Question 10</u>
Not at all sure = 1
Not very sure = 2
Somewhat sure = 3
Mostly sure = 4
Completely sure = 5

Question 11
Do not understand at all = 1
Do not understand very well = 2
Somewhat understand = 3
Mostly understand = 4
Completely understand = 5

- If at least one of Questions 10 and 11 is not missing, then compute

  Self-Efficacy Score = 100*[(mean of Questions 10 and 11 actually answered) – 1]/4

7. Quality of Life

- Code responses to Questions 12, 13 and 14 as follows:

Question 12
It has extremely limited my enjoyment of life = 1
It has limited my enjoyment of life quite a bit = 2
It has moderately limited my enjoyment of life = 3
It has slightly limited my enjoyment of life = 4
It has not limited my enjoyment of life at all = 5

Question 13
Not at all satisfied = 1
Mostly dissatisfied = 2
Somewhat satisfied = 3
Mostly satisfied = 4
Completely satisfied = 5

Question 14
I felt that way all of the time = 1
I felt that way most of the time = 2
I occasionally felt that way = 3
I rarely felt that way = 4
I never felt that way = 5

- If at least one of Questions 12, 13 and 14 is not missing, then compute

  Quality of Life Score = 100*[(mean of Questions 12, 13 and 14 actually answered) – 1]/4

8. Social Limitation

- Code responses to each of Questions 15a-d as follows:

Severely limited = 1
Limited quite a bit = 2
Moderately limited = 3
Slightly limited = 4
Did not limit at all = 5
Does not apply or did not do for other reasons = <missing value>

- If at least two of Questions 15a-d are not missing, then compute

  Social Limitation Score = 100*[(mean of Questions 15a-d actually answered) – 1]/4

9. Overall Summary Score

= mean of the following available summary scores:
Physical Limitation Score
Total Symptom Score
Quality of Life Score
Social Limitation Score

10. Clinical Summary Score

= mean of the following available summary scores:
Physical Limitation Score
Total Symptom Score

Note: references to **“means of questions actually answered”** imply the following.

- If there are n questions in a scale, and the subject must answer m to score the scale, but the subject answers only n-i, where n-i >= m, calculate the **mean of those questions** as

  (sum of the responses to those n-i questions) / (n-i)
  not

(sum of the responses to those n-i questions) / n

11.1.2. SAQ-7

Three domain scores and one summary score are generated from the SAQ-7:

Physical Limitation Score (SAQ7-PL)

Angina Frequency Score (SAQ7-AF)

Quality of Life Score (SAQ7-QL)

Summary Score (SAQ7)

Scores are scaled 0-100, where 0 denotes the lowest reportable health status and 100 the highest.

<u>Physical limitation Score</u>

The physical Limitation score corresponds to Questions 1a, 1b and 1c. Responses are coded as follows:

Extremely limited	1
Quite a bit limited	2
Moderately limited	3
Slightly limited	4
Not at all limited	5
Limited for other reasons or did not do the activity	6

A response of 6 is treated as missing value for the purpose of scoring. If responses to two or more questions are missing, no score is computed. If the response to Question 1a or Question 1c is missing, it is assigned the responses from Question 1b. If the response to Question 1b is missing, it is assigned the average of responses to Questions 1a and 1c. The score is then calculated by taking the average of the three responses and rescaling to 0 – 100, as follows:

SAQ7-PL = 100*[(average of Questions 1a, 1b and 1c)-1]/4

Angina Frequency Score

The Angina Frequency score corresponds to Questions 2 and 3. Responses are coded as follows:

4 or more times per day	1
1 – 3 times per day	2
3 or more times per week but not every day	3
1 -2 times per week	4
Less than once a week	5
None over the past 4 weeks	6

If responses to both questions are missing, no score is computed. Otherwise, the score is calculated by taking the average of non-missing responses and rescale to 0-100 as follows:

SAQ7-AF = 100 *[(average of Questions 2 and 3) -1]/5

Quality of Life Score

The quality of life score corresponds to Questions 4 and 5. Responses are coded as follows:

Question 4	
It has extremely limited my enjoyment of life	1
It has limited my enjoyment of life quite a bit	2
It has moderately limited my enjoyment of life	3
It has slightly limited my enjoyment of life	4
It has not limited my enjoyment of life at all	5
Question 5	
Not satisfied at all	1
Mostly dissatisfied	2
Somewhat satisfied	3
Mostly satisfied	4
Completely satisfied	5

If responses to both questions are missing, no score is computed. Otherwise, the score is calculated by taking the average of the non-missing response and rescaling to 0 -100, as follows:

SAQ7-QL= 100*[(average of Question 4 and 5)-1]/4

Summary Score

The summary score represents an integration of the patients' physical limitation, angina symptom and quality of life. If all three domain scores are missing, no summary score is computed. Otherwise, the score is calculated as the average of the non-missing domain scores:

SAQ7 = average of SAQ- PL, SAQ- AF, and SAQ – QL

11.1.3. EQ-5D-5L

Five dimensions of the EQ-5D-5L include 'mobility', 'selfcare', 'activity', 'pain', and 'anxiety'. The US Pickard value set will be used to compute the EQ-5D-5L index values. The value set will be denoted as disut_mo for 'mobility', disut_sc for 'selfcare', disut_ua for 'activity', disut_pd for 'pain', and disut_ad for 'anxiety' in Table 7 below:

Table 7: EQ-5D-5L Value Set

		US value set
MOBILITY		disut_mo
I have no problems in walking about	1	0
I have slight problems in walking about	2	0.096
I have moderate problems in walking about	3	0.122
I have severe problems in walking about	4	0.237
I am unable to walk about	5	0.322
SELF-CARE		disut_sc
I have no problems washing or dressing myself	1	0
I have slight problems washing or dressing myself	2	0.089
I have moderate problems washing or dressing myself	3	0.107
I have severe problems washing or dressing myself	4	0.220
I am unable to wash or dress myself	5	0.261
USUAL ACTIVITIES		disut_ua
I have no problems doing my usual activities	1	0
I have slight problems doing my usual activities	2	0.068
I have moderate problems doing my usual activities	3	0.101
I have severe problems doing my usual activities	4	0.255
I am unable to do my usual activities	5	0.255
PAIN / DISCOMFORT		disut_pd
I have no pain or discomfort	1	0
I have slight pain or discomfort	2	0.060
I have moderate pain or discomfort	3	0.098
I have severe pain or discomfort	4	0.318
I have extreme pain or discomfort	5	0.414
ANXIETY / DEPRESSION		disut_ad
I am not anxious or depressed	1	0
I am slightly anxious or depressed	2	0.057
I am moderately anxious or depressed	3	0.123
I am severely anxious or depressed	4	0.299
I am extremely anxious or depressed	5	0.321
We would like to know how good or bad your health is TODAY	0 to 100	

disut_total=disut_mo+disut_sc+disut_ua+disut_pd+disut_ad;
The EQ-5D-5L index value (EQindex) =1-disut_total

The SAS code will be provided in Appendix Section 11.5

11.2. Table of Contents for Data Display Specifications

Table of contents for data display specifications will be provided in a separate document.

11.3. Data Display Specifications

Data display specifications will be provided in a separate document.

11.4. Analysis Windows

Measurements collected during the 24-week double-blind placebo-controlled period will be included only in the analysis windows up to Week 24. For data collected at a scheduled post randomization, the analysis visit will be the nominal visit as collected and visit window will not be applied.

For unscheduled or early discontinuation post randomization, analysis visit will be used according to Table 8 below when the scheduled visit is not available.

Table 8: Analysis Windows for Measurements

Visit	Scheduled Day	Lower Bound	Upper Bound
Screening	<1	<1	<1
Day 1	1	1	1
Week 2	15	2	21
Week 4	29	22	35
Week 6	43	36	49
Week 8	57	50	70
Week 12	85	71	98
Week 16	113	99	126
Week 20	141	127	154
Week 24	169	155	196
Week 28/Follow-up	last dose + 28 days	-	-

Note: Week 28/Follow-up should occur 4 weeks after last dose. The analysis visit of week 28/Follow-up will include the nominal visit End of Study (Week 28) and any unscheduled visits occurred after the week 24 window.

11.5. Sample SAS Codes

ANCOVA model for primary endpoint

```
proc mixed data=work;
        class <treatment arm (ref=’0’)> <Beta Blocker use> <Exercise Modality>;
        model chg=<base pvo2 > <base weight> <treatment arm> <beta blocker use> <Exercise
        Modality> /solution s Influence(EFFECT=usubjid) outp=out vciry;
        lsmeans <treatment arm>/pdiff cl;
        ods output solutionf=mixparms covB=mixcovb;
run;
```

This code assumes that the analysis involves 2 levels in treatment arm (e.g., placebo group is coded as 0 and aficamten is coded as1).

MMRM model

```
proc mixed data=work;
    class <Subject> <treatment arm (ref='0')> <Beta Blocker use> <Exercise Modality> <visit>;
model <chg> = <base> <treatment arm> <visit> <visit>*<treatment arm> <Beta Blocker use> <Exercise
Modality> <visit>*<base>/ddfm=kenwardroger;
repeated <visit> / type=un subject=<Subject>;
lsmeans <visit>*<treatment arm>/cl pdiff;
run;
```

This code assumes 2 level in treatment arm with placebo group is coded as 0. visit has level of nominal visit week where continuous measurements are assessed up to Week 24.

Imputation model for primary endpoint

```
proc mi data=work seed=&seed out=miout NIMPUTE=50;
        class <treatment arm> <Beta Blocker use> <Exercise Modality> <base NYHA> <randomization
        factors>;
        var <treatment arm> <randomization stratifications> <sex> <age> <base pVO2> <base
        hemoglobin> <base KCCQ CSS> <baseline NYHA> <last available post rand NYHA> <last
        available post rand resting LVOT> <last available post rand Valsalva LVOT> <W24 pVO2> ;
        monotone reg(W24 pVO2);
  run;
```

Placebo-based imputation for sensitivity analysis of the primary endpoint

Step 1:
generate input dataset for subjects in placebo group or subject who didn't complete treatment, and datasets for the rest of subjects in aficamten group.
Step2: impute missing data with separately with each input dataset using the imputation model for the primary endpoint.
Step 3. Combined the complete data from the imputations above as final imputed dataset.

Tipping point imputation for primary endpoint

```
proc mi data=work seed=&seed out=miout NIMPUTE=50;
        class <treatment arm> <Beta Blocker use> <Exercise Modality> <base NYHA> <>;
        var <treatment arm> <sex> <age> <base pVO2> <base hemoglobin> <base KCCQ CSS>
        <baseline NYHA> <last available post rand NYHA> <last available post rand resting LVOT>
        <last available post rand Valsalva LVOT> <W24 pVO2> ;
        monotone reg(W24 pVO2);
        mnar adjust (W24 pVO2/shift=&shift adjustobs=(Trt='1'));
run;
```

shift will be set so that the imputed value for aficamten group is 1 ml/min/kg worse as starting point with increment of 1 ml/min/kg worse each time until the result is no longer significant.

Multiple Imputation for sensitivity analysis for repeated measures endpoint

```
Step1:
proc mi data=work1 seed=&seed1 out=miout1 NIMPUTE=100;
        by <treatment arm>;
        mcmc IMPUTE=monotone ;
        var <base> <var week 2> <var week4> <var week6> <var week8> <var week12> <var week16>
<var week20> <var week24>;
run;

Step 2:

proc mi data=miout1 seed=&seed NIMPUTE=1 OUT=miout2;
 class <treatment arm>;
 by _IMPUTATION_;
 var <treatment arm> <base> <var week 2> <var week4> <var week6> <var week8> <var week12> <var
week16> <var week20> <var week24>;
 monotone reg (<base> <var week 2> <var week4> <var week6> <var week8> <var week12> <var
week16> <var week20>);
 mnar model (<base> <var week 2> <var week4> <var week6> <var week8> <var week12> <var
week16> <var week20> / modelobs = (trt01pn = '0'));
run;
```

Multiple Imputation for categorical endpoint

```
proc mi data=work seed=&seed out=outwork NIMPUTE=100;
         class <var at week8> < var at week 12> < var at week 16> < var at week 20> < var at week 24>
<treatment arm>;
  var <treatment arm> <var at week8> < var at week 12> < var at week 16> < var at week 20> < var at
week 24> ;
fcs logistic (<var at week8> < var at week 12> < var at week 16> < var at week 20> < var at week 24> =
<treatment arm> /link=glogit);
run;
```

SAS codes using proc minalyze to combine results from imputed datasets

The pooled estimates from the 50 imputed datasets are obtained from the following codes.

```
proc mianalyze data=est;
  modeleffects estimate;
  stderr stderr;
run;
```

Mixed model for CPET data

```
proc mixed data=work;
    class <subject ID> <visit> <stratification factors> ;
    model <chg in CPET>=<trtid> <stratification factors> <stratification factors>*<visit> /s;
    estimate 'active at Week 24' int 1 <trtid> 1 <stratification factors> &c1 &c2 stratification
    factors>*<visit> 0 0 &c1 & / e;
    estimate 'placebo at Week 24' int 1 <trtid> 0 <stratification factors> &c1 &c2 stratification
    factors>*<visit> 0 0 &c1 & / e;
    estimate 'active vs PBO at Week 24' trt 1;
    repeated <visit>/subject=<subject id> type=un;
run;
```

where trtid is assigned as 0 at baseline, 0 for placebo group at Week 24 and 1 for active group at Week 24. &c1 and &c2 are the proportion of patients evaluated in each stratification level among all the patients.

Subgroup analysis for the primary endpoint

```
proc mianalyze parms=mixparms covb(effectvar=rowcol)=mixcovb;
    class <treatment arm> <beta blocker use> <exercise modality> <subgroup> ;
    =<base pvo2 > <base weight> <treatment arm> <beta blocker use> <Exercise modality>
    modeleffects Intercept <base pvo2 > <base weight> <treatment arm> <beta blocker use>
    <Exercise Modality> <subgroup>*<treatment arm>;
    run;
```

SAS codes to evaluate normality assumption for the primary endpoint

The normality assumptions for the ANCOVA analysis will be assessed by residual illustration. The outpred option in the above code stores residuals which are used to test the assumption of normality. Examination of residuals can be done using the following codes.

```
proc univariate data=work normal;
var ScaledResid; QQPLOT ScaledResid;
ods output QQPlot=qqplot;
run;
```

SAS codes to perform CMH test

```
proc freq data=work;
 tables <Beta blocker use>*<CPET modality>*<treatment arm>*<response Y/N>/CMH
 exactcmh;
exact riskdiff (column=2) relrisk (column=2) commor.
Run;
where response is coded as Y=1, N=0.
```

SAS codes to rank observation

```
proc rank data=work out=rwork nplus1;
Var chg;
Ranks rchg;
Run;
```

SAS code for proportional hazard Cox regression model

```
Proc phreg data=work;
        Class <treatment arm>(ref=first) ;
        Model <time>*censor (1)= ;
        Strata <beta blocker use> <CPET modality> ;
Run;
```

SAS code for stratified logistics regression model

```
Proc phreg data=work;
        Class <treatment arm>;
        Model <Response> (event='Y')= <treatment arm> <baseline covariates>/selection=stepwise
details;
        Strata <beta blocker use> ;
Run;
```

SAS code for EQ-5D-5L Index Value

```
*******************************************************************
*SAS syntax code for the computation of index*
*values with the US TTO value set*
*******************************************************************

data WORK.CAT;
set WORK.CAT;

if mobility eq 1 then disut_mo=0;
else if mobility eq 2 then disut_mo=0.096;
else if mobility eq 3 then disut_mo=0.122;
else if mobility eq 4 then disut_mo=0.237;
else if mobility eq 5 then disut_mo=0.322;

if selfcare eq 1 then disut_sc=0;
else if selfcare eq 2 then disut_sc=0.089;
else if selfcare eq 3 then disut_sc=0.107;
else if selfcare eq 4 then disut_sc=0.220;
else if selfcare eq 5 then disut_sc=0.261;

if activity eq 1 then disut_ua=0;
else if activity eq 2 then disut_ua=0.068;
else if activity eq 3 then disut_ua=0.101;
else if activity eq 4 then disut_ua=0.255;
else if activity eq 5 then disut_ua=0.255;

if pain eq 1 then disut_pd=0;
else if pain eq 2 then disut_pd=0.060;
else if pain eq 3 then disut_pd=0.098;
else if pain eq 4 then disut_pd=0.318;
else if pain eq 5 then disut_pd=0.414;

if anxiety eq 1 then disut_ad=0;
else if anxiety eq 2 then disut_ad=0.057;
else if anxiety eq 3 then disut_ad=0.123;
else if anxiety eq 4 then disut_ad=0.299;
else if anxiety eq 5 then disut_ad=0.321;

disut_total=disut_mo+disut_sc+disut_ua+disut_pd+disut_ad;
EQindex=1-disut_total;
run;
```

Summary of changes in statistical analysis plan

SAP VERSION HISTORY

Version and Date	Revision	Rationale
Final 1.0	Not applicable: original version	Not applicable
Version 2.0	Synopsis and Section 7.6: updated testing hierarchy and removed the statement related to determination of the testing order of the duration of SRT eligibility and total workload.	The testing hierarchy was simplified from parallel gate keeping method to a closed testing procedure to allow the testing of the secondary endpoints including SRT eligibility at Week 24 at two-sided alpha level of 0.05 once the primary endpoint reaches statistical significance at a specified sequential testing order.
	Section 2.2.3: corrected typo in exploratory endpoints:<br>number of patients with new or worsening ST depression during exercise at Week 12 and 24 by removing time point Week 12<br><br>added 1 exploratory efficacy endpoint	CPET is only performed at Week 24.
	Section 2.2.4: updated the definition of the endpoint of incidence of LVEF <50% with signs and symptoms of heart failure (concomitant adverse event of heart failure or dyspnea) and/or increase in NT-proBNP from baseline	Update was made to provide greater specificity to the increase in NT-proBNP (≥30% increase) in relation to worsening HF.
	Section 5.5.4: modified FAS definition by removing condition of requiring at least one post baseline efficacy measurement	Update was made to address FDA comments to the SAP.
	Section 7.2: added statement that if model assumptions are substantially violated, rank based analysis will be performed as supportive analysis.	Updates were made to address FDA comments to the SAP.
	Section 7.5: added additional analyses by IND Sites status	Updates were made to add additional analyses to have consistent scope of by IND sites status analyses
	Section 7.8: Remove COVID-19 sensitivity analyses from the secondary endpoints	The impact of COVID-19 reported on the study has been minimal.

Version and Date	Revision	Rationale
	Section 7.7.1: updated the reasons of invalid CPET	To use the same statements provided by the CPET laboratory used in the data transfer specification
	Section 7.7.4: updated to include criteria of condition when rank-based analysis will be performed and clarified that, if performed, rank-based analysis will be supportive analysis for the primary endpoint	Updates were made to address FDA comments to the SAP and provide specific criteria for the rank-based analysis to be used.
	Section 7.8.1: update the number of imputed datasets from 50 to 100	Updates were made per FDA comments to the SAP
	Section 7.8.1: clarified imputing missing Week 12 NYHA.<br>Added sensitivity analysis for proportion of responders at Week 12 and 24 after treating patients with missing data as non-responder. Added statement to impute intermittent missing data use adjacent visits results in determining patients' SRT eligibility.	Updates were made per FDA comments to the SAP; added clarification statements on imputing intermittent missing data except Week 12 and Week 24 in determining patient's SRT eligibility
	Editorial updates made where applicable	To correct editorial issues

Table S1. Average Dose of Background Medications for all Patients and by Treatment Group

	Aficamten (n=142)	Placebo (n=140)	Overall (N=282)
**Beta-blockers**			
Atenolol (mg)			
n	7	3	10
Mean (SD)	107.1 (47.3)	166.7 (104.1)	125.0 (68.7)
Median	100	200	100
Q1, Q3	100, 150	50, 250	100, 175
Betaxolol (mg)			
n	1	0	1
Mean (SD)	60.0 (NA)	NA (NA)	60.0 (NA)
Median	60.0	NA	60.0
Q1, Q3	NA	NA	NA
Bisoprolol (mg)			
n	24	25	49
Mean (SD)	8.2 (5.8)	8.6 (9.3)	8.4 (7.7)
Median	5.6	7.5	6.3
Q1, Q3	5.0, 10.0	3.8, 10.0	5.0, 10.0
Carvedilol (mg)			
n	0	2	2
Mean (SD)	NA	28.1 (30.9)	28.1 (30.9)
Median	NA	28.1	28.1
Q1, Q3	NA	6.3, 50.0	6.3, 50.0
Metoprolol succinate (mg)			
n	34	33	67
Mean (SD)	84.1 (57.73)	87.0 (60.00)	85.5 (58.43)
Median	62.5	71.3	71.3
Q1, Q3	47.5, 100.0	47.5, 125.0	47.5, 125.0
Metoprolol tartrate (mg)			
n	8	7	15
Mean (SD)	118.8 (82.10)	90.1 (63.10)	105.4 (72.78)
Median	125.0	100.0	100.0
Q1, Q3	37.5, 200.0	25.0, 100.0	25.0, 200.0
Nadolol (mg)			
n	8	6	14
Mean (SD)	91.3 (96.1)	106.7 (32.7)	97.9 (73.8)
Median	80.0	100.0	80.0
Q1, Q3	40, 80	80, 120	80, 120
Nebivolol (mg)			
n	1	7	8
Mean (SD)	10.0 (NA)	7.5 (8.2)	7.8 (7.6)
Median	10	5	5

Q1, Q3	10.0, 10	2.5, 10	2.5, 10
Propranolol (mg)			
n	1	3	4
Mean (SD)	320.0 (NA)	160.0 (80.0)	200.0 (103.3)
Median	320	160	200
Q1, Q3	320, 320	80, 240	120, 280
Sotalol (mg)			
n	2	1	3
Mean (SD)	160.0 (0)	160.0 (NA)	160.0 (0)
Median	160	160	160
Q1, Q3	160, 160	160, 160	160, 160
**Non-dihydropyridine calcium channel blockers**			
Diltiazem (mg)			
n	24	19	43
Mean (SD)	198.8 (129.4)	111.1 (49.9)	160.0 (110.3)
Median	180	120	120
Q1, Q3	90, 270	90, 120	90, 180
Verapamil (mg)			
n	22	17	39
Mean (SD)	299.1 (153.4)	215.3 (76.7)	262.6 (131.3)
Median	240	240	240
Q1, Q3	180, 480	120, 240	120, 360
**Disopyramide**			
Disopyramide (mg)			
N	16	20	36
Mean (SD)	334 (188.3)	303 (93.9)	317 (131.1)
Median	300	300	300
Q1, Q3	250, 338	225, 350	250, 338

All doses are expressed as the cumulative daily dose in milligrams.

Table S2. Timing and Indication for Dose Adjustment During SEQUOIA-HCM Study Visit

Placebo

	**Biplane LVEF <50% (n=4)**	**Biplane LVEF ≥50%–55% (n=9)**	**Biplane LVEF ≥55% and Valsalva LVOT-G ≥30 mmHg (n=48)**	**Biplane LVEF ≥55% and Valsalva LVOT-G ≥30 mmHg (n=1059)**	
Analysis visit, week					p=0.85
2	0 (0.0%)	1 (11.1%)	5 (10.4%)	134 (12.7%)	
4	1 (25.0%)	1 (11.1%)	9 (18.8%)	129 (12.2%)	
6	1 (25.0%)	0 (0.0%)	8 (16.7%)	131 (12.4%)	
8	0 (0.0%)	1 (11.1%)	4 (8.3%)	135 (12.7%)	
12	1 (25.0%)	2 (22.2%)	7 (14.6%)	130 (12.3%)	
16	1 (25.0%)	1 (11.1%)	4 (8.3%)	134 (12.7%)	
20	0 (0.0%)	3 (33.3%)	6 (12.5%)	131 (12.4%)	
24	0 (0.0%)	0 (0.0%)	5 (10.4%)	135 (12.7%)	

Aficamten

	**Biplane LVEF <50% (n=8)**	**Biplane LVEF ≥50%–55% (n=40)**	**Biplane LVEF ≥55% and Valsalva LVOT-G ≥30 mmHg (n=441)**	**Biplane LVEF ≥55% and Valsalva LVOT-G ≥30 mmHg (n=647)**	
Analysis visit, week					p<0.001
2	0 (0.0%)	2 (5.0%)	22 (5.0%)	118 (18.2%)	
4	0 (0.0%)	2 (5.0%)	35 (7.9%)	105 (16.2%)	
6	0 (0.0%)	3 (7.5%)	54 (12.2%)	85 (13.1%)	
8	1 (12.5%)	5 (12.5%)	78 (17.7%)	58 (9.0%)	
12	1 (12.5%)	8 (20.0%)	66 (15.0%)	67 (10.4%)	
16	4 (50.0%)	7 (17.5%)	63 (14.3%)	68 (10.5%)	
20	1 (12.5%)	7 (17.5%)	57 (12.9%)	77 (11.9%)	
24	1 (12.5%)	6 (15.0%)	66 (15.0%)	69 (10.7%)	

The number (n) and percentage (%) of study visits is shown in each treatment arm.
LVEF indicates left ventricular ejection fraction; LVOT-G, left ventricular outflow tract gradient.

Table S3. Treatment-Emergent Adverse Events (≥2%) per Study Phase

Titration Phase

	**Randomized to placebo (n=140)**	**Randomized to aficamten* (n=142)**
Upper respiratory tract infection	4 (2.9%)	4 (2.8%)
Headache	7 (5.0%)	9 (6.3%)
COVID-19	2 (1.4%)	6 (4.2%)
Palpitations	1 (0.7%)	6 (4.2%)
Dyspnea	4 (2.9%)	2 (1.4%)
Angina pectoris	1 (0.7%)	1 (0.7%)
Chest pain	1 (0.7%)	1 (0.7%)
Hypertension	2 (1.4%)	4 (2.8%)
Nasopharyngitis	3 (2.1%)	2 (1.4%)
Fatigue	5 (3.6%)	1 (0.7%)
Hypertrophic cardiomyopathy	3 (2.1%)	0 (0.0%)
Atrial fibrillation	1 (0.7%)	2 (1.4%)
Chest discomfort	2 (1.4%)	1 (0.7%)
Nausea	3 (2.1%)	5 (3.5%)
Dizziness	2 (1.4%)	3 (2.1%)
Non-cardiac chest pain	2 (1.4%)	1 (0.7%)
Diarrhea	3 (2.1%)	2 (1.4%)
Urinary tract infection	0 (0.0%)	2 (1.4%)
Asthenia	0 (0.0%)	1 (0.7%)
Back pain	1 (0.7%)	2 (1.4%)
Cough	0 (0.0%)	1 (0.7%)

Maintenance Phase

	Randomized to placebo (n=140)	Randomized to aficamten (n=142)
Upper respiratory tract infection	10 (7.1%)	6 (4.2%)
Headache	5 (3.6%)	2 (1.4%)
COVID-19	3 (2.1%)	2 (1.4%)
Palpitations	2 (1.4%)	4 (2.8%)
Dyspnea	3 (2.1%)	2 (1.4%)
Angina pectoris	4 (2.9%)	1 (0.7%)
Chest pain	1 (0.7%)	3 (2.1%)
Hypertension	0 (0.0%)	5 (3.5%)
Nasopharyngitis	3 (2.1%)	3 (2.1%)
Fatigue	1 (0.7%)	1 (0.7%)
Hypertrophic cardiomyopathy	1 (0.7%)	0 (0.0%)
Atrial fibrillation	4 (2.9%)	2 (1.4%)
Chest discomfort	1 (0.7%)	2 (1.4%)
Nausea	1 (0.7%)	0 (0.0%)
Dizziness	0 (0.0%)	2 (1.4%)
Non-cardiac chest pain	1 (0.7%)	1 (0.7%)
Diarrhea	1 (0.7%)	0 (0.0%)
Urinary tract infection	1 (0.7%)	1 (0.7%)
Asthenia	0 (0.0%)	3 (2.1%)
Back pain	1 (0.7%)	1 (0.7%)
Cough	0 (0.0%)	4 (2.8%)

Washout Phase

	Randomized to placebo (n=140)	Randomized to aficamten (n=142)
Upper respiratory tract infection	1 (0.7%)	2 (1.4%)
Headache	1 (0.7%)	1 (0.7%)
COVID-19	3 (2.1%)	0 (0.0%)
Palpitations	2 (1.4%)	2 (1.4%)
Dyspnea	1 (0.7%)	4 (2.8%)
Angina pectoris	3 (2.1%)	1 (0.7%)
Chest pain	0 (0.0%)	2 (1.4%)
Hypertension	1 (0.7%)	0 (0.0%)
Nasopharyngitis	0 (0.0%)	0 (0.0%)
Fatigue	1 (0.7%)	1 (0.7%)
Hypertrophic cardiomyopathy	0 (0.0%)	7 (4.9%)
Atrial fibrillation	0 (0.0%)	0 (0.0%)
Chest discomfort	0 (0.0%)	4 (2.8%)
Nausea	0 (0.0%)	1 (0.7%)
Dizziness	0 (0.0%)	1 (0.7%)
Non-cardiac chest pain	0 (0.0%)	1 (0.7%)
Diarrhea	0 (0.0%)	1 (0.7%)
Urinary tract infection	1 (0.7%)	2 (1.4%)
Asthenia	0 (0.0%)	0 (0.0%)
Back pain	0 (0.0%)	1 (0.7%)
Cough	1 (0.7%)	0 (0.0%)

All data are n (%).
*2 patients withdrew from the aficamten arm prior to completing week 8.
COVID-19 indicates coronavirus disease 2019.

Figure S1. CONSORT diagram

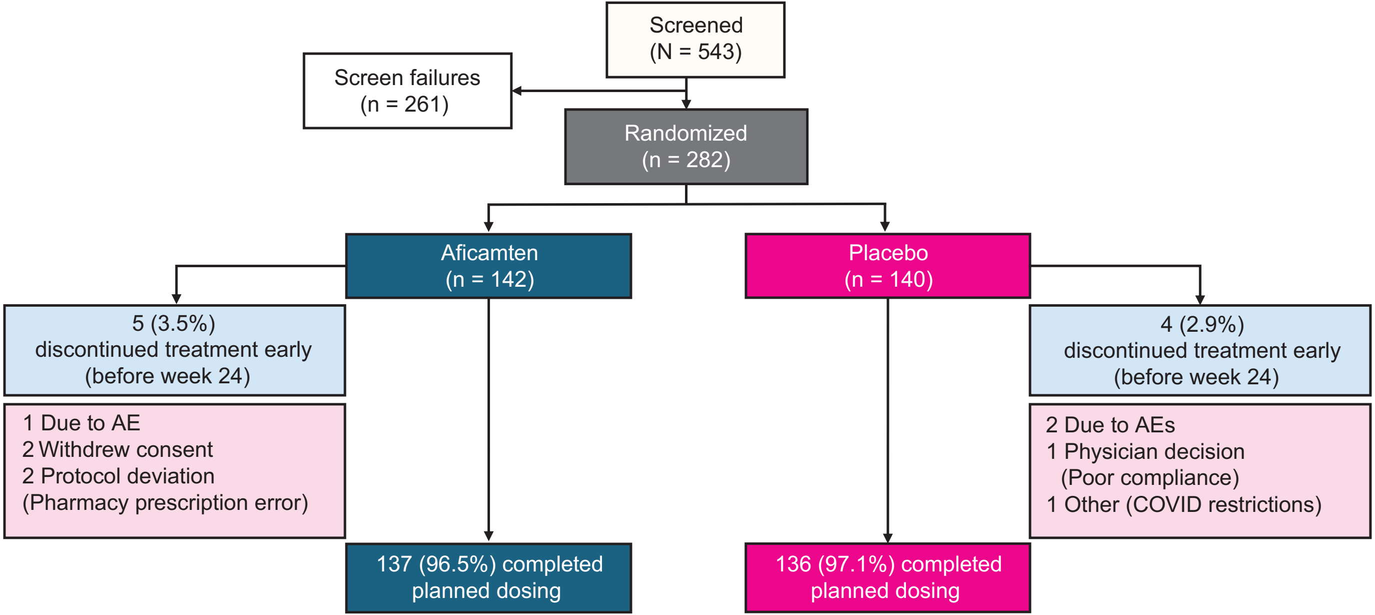

Figure S2. Spaghetti plots of individual patients with a core laboratory echocardiogram of LVEF <50%.

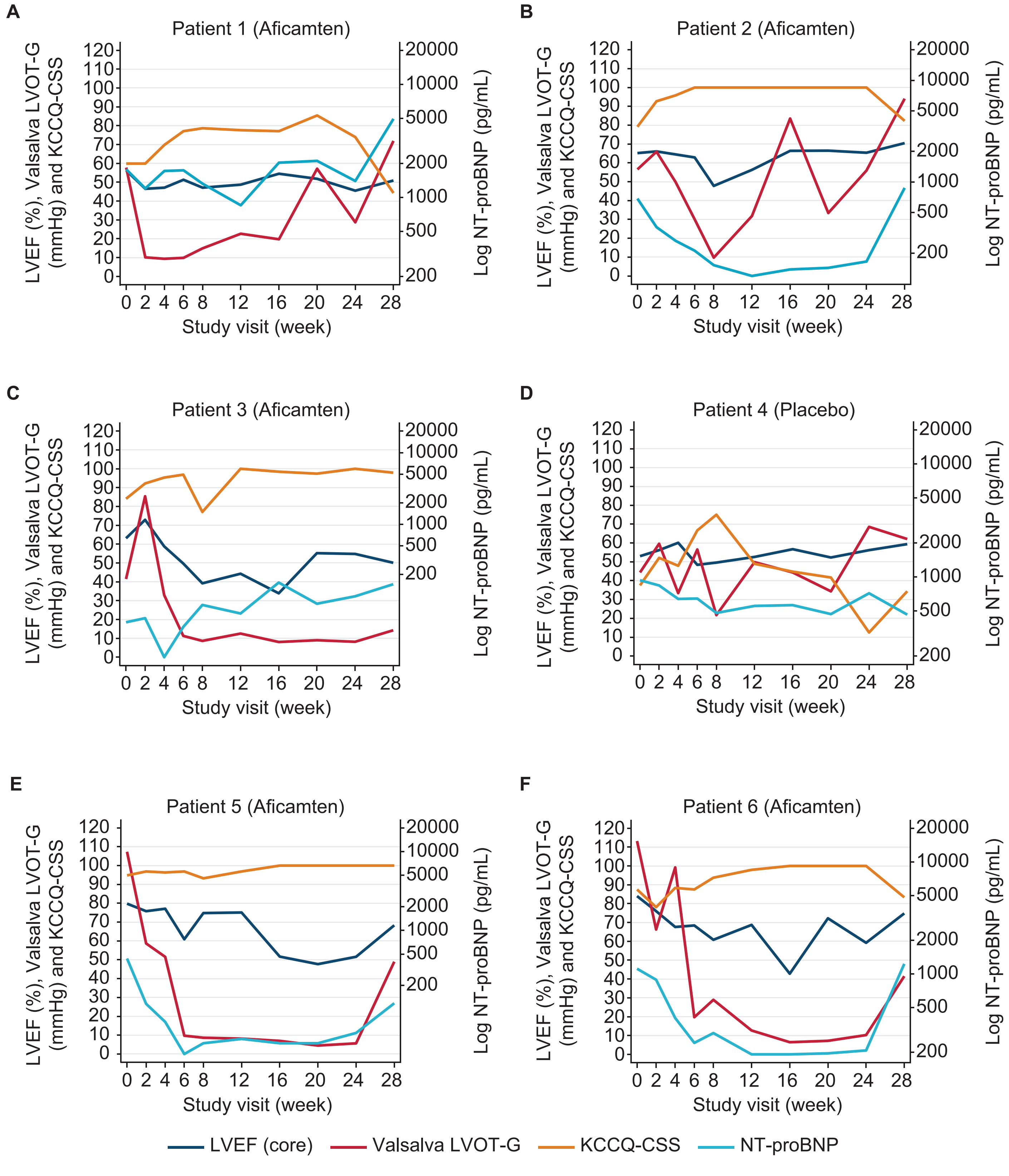

KCCQ-CSS indicates Kansas City Cardiomyopathy Questionnaire-Clinical Summary Score; LVEF, left ventricular ejection fraction; LVOT-G, left ventricular outflow tract gradient; NT-proBNP, N-terminal pro–B-type natriuretic peptide.

Figure S3. Correlation between core laboratory- and site read-measurements of echocardiographic parameters: (A) LVEF, (B) resting LVOT-G, and (C) Valsalva LVOT-G used for dosing.*

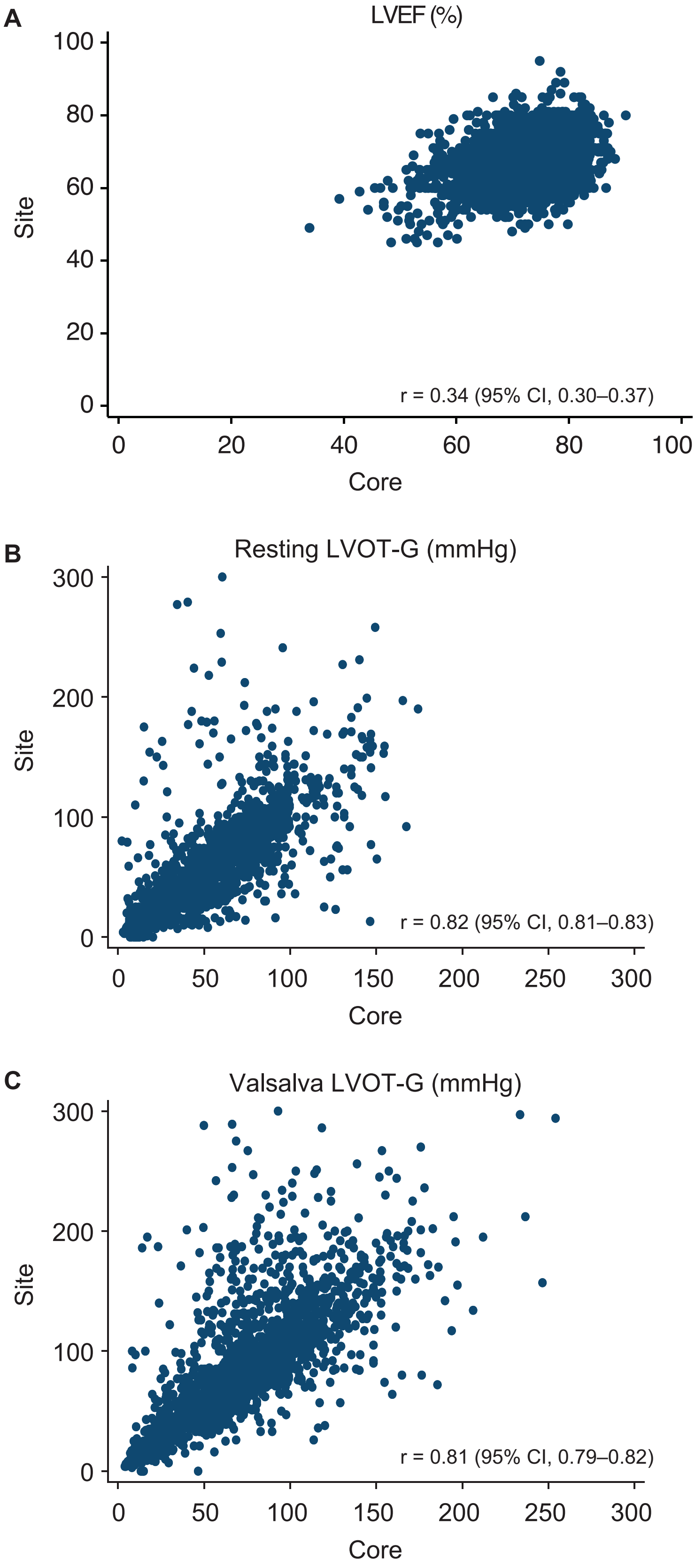

CI indicates confidence interval; LVEF, left ventricular ejection fraction; LVOT-G, left ventricular outflow tract gradient.
*Data were analyzed using correlation coefficients for site-read vs core laboratory measurements of echocardiographic parameters.
